# Genome-Wide Expression Difference of MicroRNAs in Basal Cell Carcinoma

**DOI:** 10.1155/2021/7223500

**Published:** 2021-08-04

**Authors:** Hai-Peng Wei, Song Zhan, Qing-An Zhu, Zhen-Juan Chen, Xian Feng, Jun-Yuan Chen, Qi-Lin Zhang, Jingjie Zhao, Lingzhang Meng

**Affiliations:** ^1^Department of Dermatology & STD, Beihai People's Hospital, Beihai City, Guangxi Province, China; ^2^State Key Laboratory of Pharmaceutical Biotechnology, School of Life Science, Nanjing University, Nanjing City, Guangxi Province, China; ^3^Faculty of Life Science and Technology, Kunming University of Science and Technology, Kunming City, Yunnan Province, China; ^4^Life Science and Clinical Research Center, Affiliated Hospital of Youjiang Medical University for Nationalities, Baise City, Guangxi Province, China; ^5^Center for Systemic Inflammation Research (CSIR), School of Preclinical Medicine, Youjiang Medical University for Nationalities, Baise City, Guangxi Province, China

## Abstract

Distinct expression of the miRNAs has rarely been explored in basal cell carcinoma (BCC) of skin, and the regulatory role of miRNAs in BCC development remains quite opaque. Here, we collected control tissues from adjacent noncancerous skin (*n* = 15; control group) and tissues at tumor centers from patients with cheek BCC (*n* = 15; BCC group) using punch biopsies. After six small RNA sequencing- (sRNA-seq-) based miRNA expression profiles were generated for both BCC and controls, including three biological replicates, we conducted comparative analysis on the sRNA-seq dataset, discovering 181 differentially expressed miRNAs (DEMs) out of the 1,873 miRNAs in BCCs. In order to validate the sRNA-seq data, expression of 15 randomly selected DEMs was measured using the TaqMan probe-based quantitative real-time PCR. Functional analysis of predicted target genes of DEMs in BCCs shows that these miRNAs are primarily involved in various types of cancers, immune response, epithelial growth, and morphogenesis, as well as energy production and metabolism, indicating that BCC development is caused, at least in part, by changes in miRNA regulation for biological and disease processes. In particular, the “basal cell carcinoma pathways” were found to be enriched by predicted DEM targets, and regulatory relationships between DEMs and their targeted genes in this pathway were further uncovered. These results revealed the association between BCCs and abundant miRNA molecules that regulate target genes, functional modules, and signaling pathways in carcinogenesis.

## 1. Introduction

Basal cell carcinoma (BCC) is the most ordinary human solid tumor in the skin, accounting for approximately 80% of nonmelanoma skin cancers [1, 2]. Most BCC patients usually exhibit translucent lesions in superficial and easily develop symptoms such as bleeding and crusting. In general, BCC primarily occurs in the scalp, cheek, forearm, thorax, popliteal, and preauricular regions, with the cheek being the most common region [3, 4]. From the first case described by Krompecher in 1900, several investigations have been conducted to uncover the molecular pathogenesis of BCC. For example, several tumor-promoting signal transduction pathways have been reported, such as the sonic hedgehog (Shh) and mitogen-activated protein kinase/extracellular signal-regulated kinase (Mapk/Erk) pathways [1, 2, 5]. Additionally, several genes/proteins have also been reported to be associated with the development of BCC. In particular, the patched (Ptch1) receptor binding to the Shh ligand casts impacts on the smoothened protein (Smo) on the cell membrane, which results in the suppression of the zinc finger transcription factor gene (*GLI1*) [2]. A mechanism as such counteracts cell-cycle arrest and leads to the development of skin hair follicle BCCs [6–8]. Moreover, functional loss of the Ptch1 receptor caused by gene mutations has been detected in approximately 70% of BCCs [2, 8]. In addition, gain-of-function mutations of SMOH have been found in approximately 15% of BCCs [2]. These data indicates that variations in dimensions of expression and sequence (including pathways) of genes are vital for the progress of BCCs.

MicroRNAs (miRNAs) are a rather novel type of short RNAs which are known to be involved in posttranscriptional gene expression [9]. As it has been evaluated that 30–60% of the entire human gene expression is controlled by miRNAs, a possible function of miRNAs in the molecular etiopathogenesis of BCC needs to be investigated [1]. As a class, miRNAs function across numerous processes of the skin physiologically and pathophysiologically, as well as across other human tissues [10, 11]. The miRNA maturing enzymes DICER and DROSHA, partial miRNA microprocessor complex, along with the miRNA effector RNA-caused silencing complex have been discovered to be dysregulated in epithelial skin cancers which includes BCC [12]. Currently, several studies have revealed miRNA expression profiles from BCC and nonlesional epithelial skin samples of clinical patients by using small RNA sequencing (sRNA-seq) and miRNA microarrays [1, 2]. These miRNA-based researches have been primarily focused on identifying the differentially expressed miRNAs (DEMs) in BCCs by comparing with control tissues, but the molecular etiopathogenesis of BCCs with regard to miRNAs has not been broadly investigated, especially on the regulatory functions (i.e., target gene function and signaling pathways) and relationships (i.e., networks of miRNA-targeted genes and miRNA-targeted genes-pathways) of miRNAs. Hence, it is essential to systematically recognize the miRNAs participating in BCCs and analyze the potential genes and pathways that are possibly regulated by them. Though numerous reports have emphasized the biological/pathological role of miRNAs played in the development of BCC, few studies have been found to illustrate the landscape of miRNA in BCC biopsies.

In this study, we identified miRNAs in BCCs using sRNA-seq and bioinformatics approaches, while discovering a panel of DEMs in BCC tissues by comparing genome-wide miRNA transcriptional profiles of BCC with control groups and by conducting TaqMan quantitative real-time PCR (qPCR). Subsequently, based on the reference gene sets of humans, the primary regulatory functions and relationships of these miRNAs were further elucidated. The regulatory roles of miRNAs have been unveiled by our research, and their fundamental biological role will facilitate the comprehension of the molecular pathogenesis of BCCs.

## 2. Materials and Methods

### 2.1. BCC Materials

Our research was granted by the Ethical Review Board of the Beihai People's Hospital Guangxi Province, China (Approval No. Beiyi (lunshen) 2020003). As we adopted the dermatological surgery section, the present research was conducted according to Ethical Principles for Medical Research Involving Human Sub in the light of the Declaration of Helsinki (version 2013). Every subject of this research has provided written informed consent.

The BCC and control samples in the cheeks of five patients were collected as per abovementioned methods [1, 2, 4]. To be specific, during the micrographic controlled surgery with cold steel by regional anaesthesia, 4 mm punch biopsies were both taken from cancer cores (BCC groups) and adjacent noncancerous epithelial skin (control groups). Each sample was conducted three times independently (*n* = 5 patients per collection; *n* = 15 patients across three times) with three biological replicates. Overall, 15 patients (*n* = 7 female and *n* = 8 male; mean age 67.4 years) with cheek BCC were included in our research (Table 1). The specimens were immediately put in RNAlater (Qiagen, Hilden, Germany) and reserved until further use at -80°C.

### 2.2. RNA Extraction, Library Construction, and Small RNA Sequencing

Entire RNA was extracted from each BCC and control specimens and treated with the TRIzol reagent (Invitrogen, CA, USA) as per the manual from the supplier. Residual genomic DNA was extracted via RNase-free DNase Set (Qiagen, Germany), as per the manual from the supplier. The purity was evaluated by using a NanoDrop ND-1000 spectrophotometer (Thermo Scientific, CA, USA), while the RNA structural completeness was validated using agarose gel electrophoresis at 1.5%. To eliminate biological variations by different levels of gene expression in each biological replicate, the entire RNA from each sample was diluted to 200 ng/*μ*L using RNase-free water. Afterwards, an equal volume of the RNA from all five BCC samples of each biological replicate was pooled together to develop a sRNA-seq library, and RNA from five control samples was collected likewise. According to the MGIEasy Small RNA Library Prep Kit V2.0 (Illumina, CA, USA) protocol, approximately 2 *μ*g of total pooled RNA was utilized to prepare a small RNA library. To generate three biological replicates, the experiments were independently repeated three times. Under rigorous quality control standards, we carried out single-end sequencing (45 bp) of six sRNA libraries on an BGISEQ-500 at the Beijing Genomics Institute (BGI, Shenzhen, China).

### 2.3. Preprocessing Analysis, Annotation of miRNAs, and Differentially Expressed miRNAs

The raw tags were filtered by eliminating defective sequences, sequences with poly-A tail and adaptors, while the remaining sequences were retained as clean tags. The length distributions of clean sequences were then summarized, followed by the removal of the highly repetitive clean tags via the RepeatMasker tool (http://www.repeatmasker.org/). In order to avoid nonhuman contamination, the clean tags were mapped to the human reference genome (NCBI version: GRCh38.p13) using the Bowtie2 [13] tool. Subsequently, these clean tags were searched within the GenBank and Rfam databases by using Bowtie2 to determine the annotation information of non-miRNA sRNAs (e.g., rRNAs, tRNAs, and snoRNAs). Eventually, the non-miRNA sequences were removed for the following analysis.

To identify known miRNAs, the obtained clean tags were then annotated using the miRBase 22.1 database (http://www.mirbase.org/), while the completely matched (no mismatch base) clean tags with mature miRNAs in the miRBase were recognized as miRNAs. To acquire dependable outcomes by strict standards, miRNAs that were shared by the 3 biological duplicates were reserved as the miRNA set for control and BCC samples, respectively. In order to calculate the mapped tags and quantify the miRNA expression in downstream analyses, all clean tags were aligned to the miRNA sequences using Bowtie2.

The expression of miRNAs across the entire 6 libraries was quantified as per the obtained count of mapped tags for each miRNA via the quantification method of transcripts per million kilobase (TPM) values [14]. Pearson's correlation (*r*) between two libraries was computed to determine the reproducibility within the 3 biological duplicates. Certain miRNAs (more than two mapped tags) were either not detected or only detected in BCC groups, which suggests that the expression of these miRNAs was affected by the BCC. In order to include these potential candidates for the BCC process, the union of recognized miRNAs in the control and BCC samples was utilized to identify DEMs. DESeq v1.36.0 [15], an R program suitable for the identification of differentially expressed genes from high-throughput sequencing data, helped identify DEMs between the control and BCC samples. The miRNAs with fold change (FC) ≥ 2 (∣Log_2_ ratio∣≥1) and *p* values (Wald test in DESeq) rectified by a false discovery ratio (FDR) < 0.001 were recognized as DEMs.

### 2.4. Prediction and Functional Enrichment Analysis of miRNA Target Genes

Available human gene sets downloaded from NCBI were utilized as the reference geneset to predict miRNA targets. Furthermore, gene functions were annotated by searching within the NCBI nonredundant protein (NR) databases by using a Blastx tool with default parameters. To determine the controlling role of DEMs in BCCs, the potential target genes of miRNAs were predicted by adopting TargetScan v7.2 [16] and miRanda both of which are appropriate for forecasting the target genes of mammalian miRNAs [17]. The forecast shared by both methods was reserved as dependable targets.

In order to elucidate the biological role of DEM-regulated target genes and signaling pathways, the functional analyses of miRNA target genes were implemented by using the Database for Annotation, Visualization, and Integrated Discovery (DAVID) v6.8 [18, 19]. By adopting the Functional Annotation Clustering tool, Gene Ontology (GO) terms, and Kyoto Encyclopedia of Genes and Genomes (KEGG) pathways, the annotated functional terms of DEMs were clustered, while each clustered set with an FDR-value < 0.05 was taken as remarkably enriched. Furthermore, the superfluous GO terms were thoroughly removed using GO Trimming [20].

### 2.5. Validation of sRNA-seq Data Using TaqMan qPCR

In order to validate the reliability of sRNA-seq results, relative quantification of the TaqMan qPCR approach was utilized to determine the expression of 15 casually picked DEMs, involving 8 upregulated and 7 downregulated DEMs, by utilizing the identical total RNA specimens which were employed in sRNA-seq. The TaqMan® Advanced miRNA cDNA Synthesis Kit (Applied Biosystems, Darmstadt, Germany) was adopted to conduct the poly(A) tailing reaction, adaptor ligation reaction, reverse transcription (RT) reaction, and miR-Amp reaction (miRNA preamplification) in order, as per the manufacturer's instructions. Afterwards, RNase-free water was added in order to dilute miR-Amp products into 10 ng/*μ*L. The TaqMan® Advanced miRNA Assays kits for qPCR reaction were made-to-order for each miRNA, including the 2x TaqMan Fast Advanced Master Mix, TaqMan probes, and PCR primers. The U6 snRNA was selected as a reference gene [1], while product information for each miRNA is listed in Supplementary Table 1. Each qPCR reaction system at an eventual reaction volume of 10 *μ*L was implemented as per the manufacturer's instructions of kits in an ABI 7500 Fast Real-Time PCR System (Applied Biosystems, Darmstadt, Germany). The following experimental instructions were adopted for qPCR: enzyme activation at 95°C for 20 s, subsequently 40 cycles of denaturation at 95°C for 3 s, and annealing and extension at 60°C for 30 s. A negative control without template was included, while 3 technical duplicates and 3 biological duplicates were conducted for the qPCR reaction of each miRNA.

Relative expression was computed by the 2^-*ΔΔ*CT^ approach [21] in the MedCalc v19.6.1 statistics software, the outcomes of which were described as the mean ± standard deviation (SD). By using significance, calculation of the Pearson correlation coefficient between sRNA-seq and qPCR results was also implemented via this statistical software.

## 3. Results

### 3.1. Overview of Sequencing Data

For the 6 sRNA-seq libraries, about 88 million and 87 million raw tags were attained by sequencing the control and BCC samples, separately. The percentage of Q20 tags in the raw data of each library was greater than 99%, while the clean tags accounted for more than 80% of the raw tags in every sRNA-seq library after data filtering. Afterwards, the obtained clean tags were uploaded onto the NCBI BioProject (accession No. PRJNA597543, weblink: https://www.ncbi.nlm.nih.gov/bioproject/?term=597543). The percentage (approximately 92-96%) of mapped clean tags to the reference genome was almost identical among the six sRNA-seq libraries, which indicates no bias in data generation among libraries. After the annotation of various sRNA types, most clean tags were confirmed to be miRNAs, accounting for 60-65% of all clean tags in controls and 78-83% of all the clean tags in BCCs. Detailed information regarding the miRNAs is generalized in Table 2, while the length distribution of the most abundant clean tags is primarily concentrated from 21-23 bp in each library (Supplementary Figure 1). These results are consistent with the known size of sRNAs [22], indicating the reliability of sRNA-seq.

**Figure 1:**
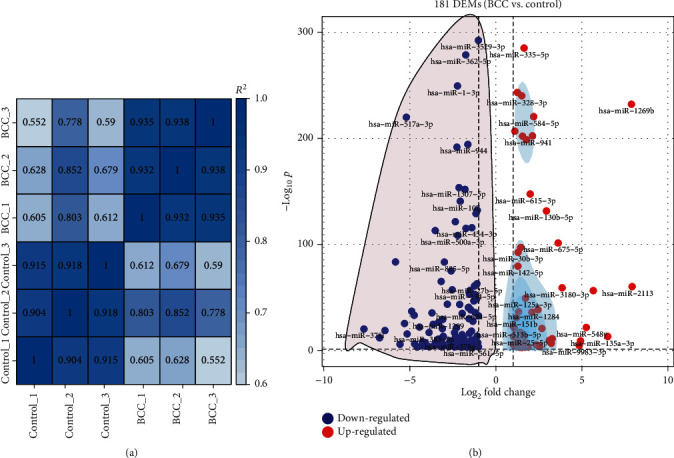
Overview of sRNA sequencing results. (a) Heatmap of Pearson correlation coefficients across diverse specimens. Greater values and darker-blue squares refer to higher similarity between the two sequencing libraries. BCC_1 to BCC_3 indicate sRNA-seq libraries of BCCs across three biological replicates. Control_1 to Control_3 indicate sRNA-seq libraries of controls across three biological replicates. (b) Volcano plot shows the distribution of 181 DEMs (BCC *vs.* control). The DEMs with ∣Log_2_FC | >1 and *p* < 0.05 were considered significant ones.

Based on the expression (TPM values) of each identified sRNA molecule, the Pearson correlation coefficient was more than 0.9 for in pair contrast of biological replicate libraries from the identical treatment (Figure 1(a)). The outcome suggests sampling effectiveness and reliable expression quantification within this study.

### 3.2. Identification of miRNAs

For the control groups, 1,997, 1,954, and 1,901 miRNAs were identified as known miRNAs across the three biological replicates. In total, 1,534 known miRNAs were shared across these 3 libraries. For the BCC groups, 1,982, 1,999, and 1,987 known miRNAs were also identified across the 3 biological duplicates, 1,656 known miRNAs of which were shared by the entire 3 libraries. Eventually, when we integrated the control and BBC samples, a total of 1,873 known miRNAs (Supplementary Table 2) were identified and used for the identification of additional DEMs.

### 3.3. Differentially Expressed miRNAs and TaqMan qPCR Validation

**Figure 2:**
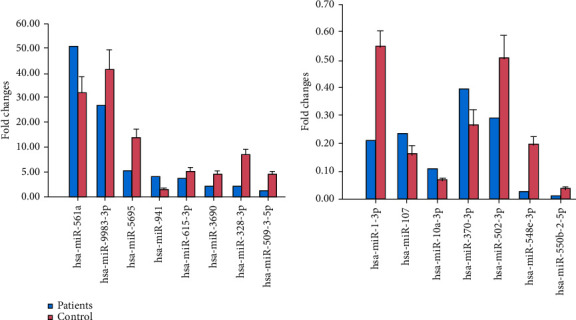
Validation of sRNA-seq expression profiles using TaqMan qPCR. Blue bars represent results from sRNA-seq, while pink bars represent results of qPCR. Results of qPCR analysis are shown as mean ± SD (*n* = 9). Data represents 3 independent experiments.

Overall, 181 miRNAs were identified to be DEMs (Figure 1(b)) in this study, indicating a systematic identification for DEMs in BCCs. These DEMs accounted for 9.66% of all miRNAs that were identified in this study and comprised 53 upregulated and 128 downregulated miRNAs (Supplementary Table 3). Meanwhile, The miRNAs with the top 10 different expression levels are listed in Table 3. Seven miRNAs (hsa-miR-2113, hsa-miR-1269b, hsa-miR-135a-3p, hsa-miR-551a, hsa-miR-548y, hsa-miR-548i, and hsa-miR-9983-3p) were shown to be downregulated by greater than 16-fold in BCCs, while all top 10 miRNAs were downregulated by greater than 16-fold. The TaqMan qPCR analysis for all 15 DEMs represents a close correlation (Pearson's correlation coefficients = 0.878, *p* < 0.01) in fold changes for DEMs between sRNA-seq and qPCR (Figure 2), which suggests the accuracy of sRNA-seq and DEM analyses. Among the upregulated DEMS, it is known that has-miR-9983-3p, has-miR-5695, and has-miR-941 contribute to the development of nasopharyngeal carcinoma, thyroid carcinoma, and laryngeal squamous cell carcinoma.

### 3.4. Function of Target Genes Regulated by Differentially Expressed miRNAs

In total, 1,356 DEM target genes that were shared by TargetScan and miRanda analyses were identified. Results from the GO enrichment method suggested that these target genes were significantly enriched within the 39 biological process terms (Supplementary Table 4). The “biological process” functions of DEM target genes were primarily involved in epidermal growth and morphogenesis, immune response, cell regulation and migration, metabolism processes, and skin development.

Moreover, 38 KEGG signaling pathways are remarkably enriched by target genes of DEMs (Table 4). Furthermore, 10 KEGG signaling pathways enriched by target genes of DEMs were identified as typical cancer-related pathways, including basal cell carcinoma (ko05217), breast cancer (ko05224), and hepatocellular carcinoma (ko05225). The remaining KEGG terms are primarily associated with ATP production (i.e., oxidative phosphorylation (ko00190) and citrate cycle (TCA cycle) (ko00020)) and metabolism (e.g., protein digestion and absorption (ko04974), histidine metabolism (ko00340), and mineral absorption (ko04978)).

### 3.5. Regulatory Roles of DEMs for Basal Cell Carcinoma Pathway

**Figure 3:**
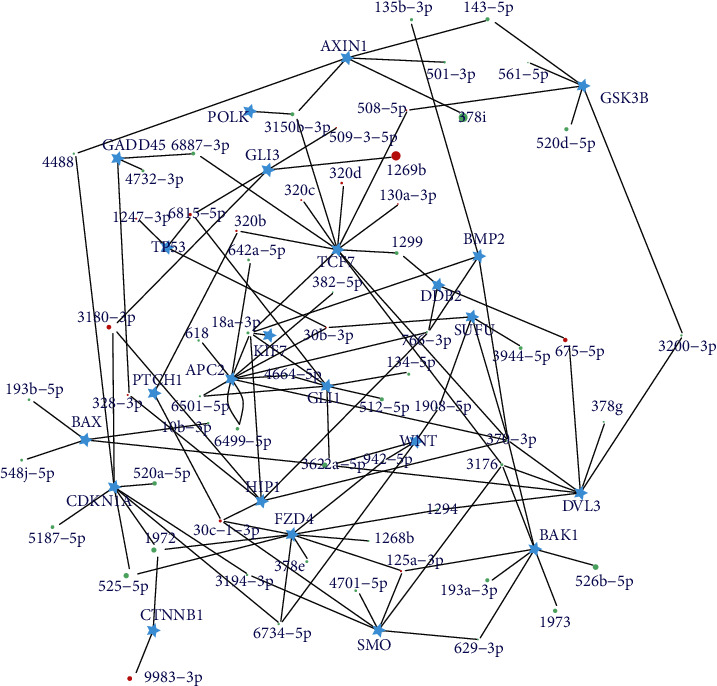
Regulatory networks of the miRNA-targeted gene members in the basal cell carcinoma (ko05217) pathway. Red solid circles represent upregulated miRNAs, while green solid circles indicate upregulated miRNAs. Larger size shows higher fold changes of miRNA expression between BCCs and controls. Blue stars indicate target genes.

“Basal cell carcinoma” (ko05217) enriched by predicted target genes of DEMs is a typical signaling pathway involved in the development of BCC, and therefore, its gene members have a key role. The DEMs-targeting gene members in this pathway are considered to be key miRNAs (KmiRNAs) for BCCs. In order to further explore the regulatory roles of these KmiRNAs in BCCs, their target gene members in the “basal cell carcinoma” KEGG pathway were further extracted (Supplementary Table 5), according to the miRNA-target relationships predicted above, and regulatory networks between the KmiRNAs and these pathways were constructed (Figure 3). In total, 49 DEMs were predicted to be KmiRNAs, and 24 gene members (marked in a red box in Supplementary Figure 2) in the “basal cell carcinoma” pathway were shown to be regulated by these KmiRNAs. Notably, the majority of KmiRNAs (e.g., hsa-miR-629-3p, hsa-miR-3176, hsa-miR-3194-3p, and hsa-miR-4701-5p) that regulated the smoothened homolog precursor (*SMO*), an oncogene, are significantly downregulated in BCCs. Conversely, there is a remarkable upregulated expression of most KmiRNAs (e.g., hsa-miR-320b, hsa-miR-30c-1-3p, hsa-miR-30b-3p, hsa-miR-6815-5p, and hsa-miR-1247-3p) that regulate genes encoding tumor suppressors (i.e., *PTCH1* and *TP53*) in BCCs.

In addition, six genes that are involved in tumorigenic processes, such as uncontrolled proliferation, increased survival of cancer cells, and genomic instability, have been predicted to be targets of KmiRNAs. These genes include cyclin-dependent kinase inhibitor 1A (*CDKN1A*), apoptosis regulator BAX (*BAX*), DNA damage-binding protein 2 (*DDB2*), growth arrest and DNA-damage-inducible protein (*GADD45*), bcl-2 homologous antagonist/killer 1 (*BAK1*), and DNA polymerase kappa (*POLK*). Furthermore, seven genes associated with cancer cell invasion were detected in the basal cell carcinoma pathway, including frizzled 4 (*FZD4*), segment polarity protein dishevelled 3 (*DVL3*), glycogen synthase kinase 3 beta (*GSK3B*), axin 1 (*AXIN1*), adenomatosis polyposis coli protein (*APC2*), catenin beta 1 (*CTNNB1*), and transcription factor 7 (*TCF7*). Moreover, seven genes that play a role in cancer cell proliferation were also found to be regulated by KmiRNAs, including suppressor of fused homolog (*SUFU*), zinc finger protein GLI3 (*GLI3*), bone morphogenetic protein 2 (*BMP2*), hedgehog interacting protein 1 (*HIP1*), *GLI1*, patched 2 (*PTCH2*), and wingless-type MMTV integration site family, member 9 (*WNT*). Notably, the majority of KmiRNAs that regulate these cancer/invasion/proliferation-promoting genes were found to be significantly downregulated in BCCs. Interestingly, numerous gene members in the basal cell carcinoma pathway were found to be regulated by multiple miRNAs, with the exception of *POLK* and *KIF7*; meanwhile, for many KmiRNAs, one of them (e.g., hsa-miR-320b, hsa-miR-18a-3p, and hsa-miR-370-3p) targets more than one gene members.

## 4. Discussion

For miRNAs, several studies have characterized DEMs in BCCs through the use of sRNA-seq, microarray, and RT-PCR miRNA profiling [1, 2]. However, to date, no studies have explored the regulatory function and relationships of miRNAs in BCCs. In the present research, we identified massive miRNAs in BCCs using sRNA-seq and bioinformatics. Furthermore, we also identified a group of DEMs, which is far more than the number of DEMs that were previously reported in BCCs (e.g., 26 DEMs [1] and 33 upregulated miRNAs [2]). Thus, it is clear that previous studies have largely underestimated the number and types of DEMs in BCCs. In addition, we found that most DEMs that were identified by previous studies were also detected in this study, including hsa-miR-223, hsa-miR-452-5p, and hsa-miR-941. This observation indicates that these miRNAs are common regulators in various types of BCCs and that they are basic miRNAs for the development of BCC. These common DEMs may represent a potential biomarker for early BCC detection.

Overall, we identified 181 miRNAs that were significantly differentially expressed in our cohort, indicating that these miRNAs participate in BCC processes. In particular, upregulated miRNAs were detected among the list of top 10 DEMs in BCC. The regulatory function of several of these DEMs has been reported in previous studies. For example, upregulated expression of hsa-miR-1269b has been found in hepatocellular carcinoma [23] and was considered a prognostic marker for hepatocellular carcinoma [24]. Deregulation of expression of hsa-miR-135a-3p was identified in urothelial carcinoma of the bladder, and this miRNA is a biomarker for predicting survival of patients [25]. Furthermore, hsa-miR-551a was found to significantly affect the prognosis of patients with pancreatic cancer and can be used as a biomarker of tumor prognosis [26]. Previous studies have shown that single nucleotide polymorphisms (SNPs) in the hsa-miR-548 family binding site at the glyceraldehyde-3-phosphate dehydrogenase alter susceptibility to breast cancer [27]. In combination with results from this current study, the hsa-miR-548 family (i.e., hsa-miR-548y and hsa-miR-548i) can regulate cancer processes at both the gene expression and SNP level. The survivin gene (baculoviral IAP repeat containing 5, *BIRC5*), a novel member of the inhibitor of apoptosis protein family, is only expressed in tumor and embryonic tissues and is tightly associated with the differentiation, proliferation, invasion, and metastasis of tumor cells. The expression of hsa-miR-542-3p was found to be negatively correlated to survivin levels in oral squamous cell carcinoma [28]. This study found upregulated expression of hsa-miR-542-3p in BCCs, which suggests that inhibition of survivin gene expression by increasing apoptosis likely promotes BCCs. For downregulated miRNAs listed in the list of top 15 DEMs in BCC, the expression of hsa-miR-516a-5p has been described as downregulated in the decidua and villus of recurrent miscarriage patients [29]. Previous studies have demonstrated reduced expression of hsa-miR-378i in stage II colon cancer, which affects cell proliferation, cell-cell interaction, and apoptosis [30]. Furthermore, it is worth noting that regulatory functions of several miRNAs (i.e., hsa-miR-2113, hsa-miR-9983-3p, hsa-miR-3619-3p, hsa-miR-550b-2-5p, and hsa-miR-523-3p) in the list of top 15 DEMs in BCC have not yet been explored, particularly in the context of cancer. Thus, despite these miRNAs being highly differentially expressed in BCCs in comparison with normal tissues, the regulatory role of these miRNAs has yet to be fully elucidated. The results presented here provide key miRNA candidates in the development of BCCs.

In order to further explore the global regulatory functions of miRNAs for BCC, functional enrichment analyses of potential mRNAs targets of DEMs are necessary for uncovering miRNA-mediated biological processes and pathways. In this current investigation, the predicted target genes of DEMs were found to be remarkably enriched according to various GO and KEGG terms. Many cases have reported that BBCs arise from abnormal epidermal cell differentiation levels/epidermal diseases (i.e., cyst, hyperplasia, and cell carcinomas) [31, 32]. These GO terms are primarily involved in skin development, epidermal growth, and morphogenesis, which can explain why BCCs are related to an abnormal epidermal epidermis. Furthermore, we identified GO terms that are primarily involved in the immune response and metabolism, which are expected as disturbances in the immune system and metabolism and have been widely demonstrated across tumors such as BCCs [33, 34]. Cell regulation and migration is a key step for BCC developments [35]. In this study, we found that BCCs were also enriched for GO terms that are associated with cell regulation and migration. These DEMs that target regulation and migration of BCC cells can represent potential therapeutic targets to control BCC invasion.

Several enriched KEGG pathways were identified in this study, and they were largely found to be immune-related. These outcomes unveiled partial vital immune-related pathways participating in BCCs. In particular, dysfunction of immune signaling pathways possibly has an impact on cancer risk and features of cancer progression [36]. In this study, we found miRNAs that target these immune signaling pathways, providing evidence for modulation of risk and progression of cancer caused by alterations in miRNA expression, with BCCs as an example. Furthermore, at least 10 significant pathways were primarily related to typical human cancers, such as breast cancer, gastric cancer, and small-cell lung cancer. Thus, the molecular pathogenesis of BCCs may be implicated in that of other cancers, and many cancer-related signaling pathways that are regulated by miRNAs are shared among various cancer types. Previous studies that are involved in the molecular pathogenesis of human breast cancer have also targeted genes of DEMs in tumor tissues that are enriched in pathways involved in many other cancers (i.e., prostate cancer, basal cell carcinoma, and acute myeloid leukemia) [37], further supporting the speculation. Overall, this study is the first to reveal signaling pathway candidates regulated by miRNAs and the relationship between these miRNAs and pathways in BCCs.

Notably, a typical BCC-related pathway “basal cell carcinoma” was enriched in this study, which led us to uncover miRNAs that regulate this pathway and construct regulatory networks of miRNA-gene members in this pathway. In the “basal cell carcinoma pathway” that was enriched in this study, almost all genes associated with these three primary BCC processes (i.e., tumorigenesis, proliferation of cancer cells, and invasion) can be detected as targets of KmiRNAs. These results indicate that miRNAs probably regulate all the key steps in BCC processes, similar to other cancer types including breast cancer [38], prostate cancer [39], and gastric cancer [40]. Interestingly, in general, the role of miRNAs seems to be gene regulation by targeting specific transcripts for the suppression of gene expression. This study has found downregulated expression in most KmiRNAs. Evidence as such suggests that relax/reduction of miRNA-mediated inhibition of genes may play a vital role in BCC development, which needs to be further studied by detecting expression changes in target gene members. The majority of KmiRNAs that have upregulated expression primarily target tumor suppressor genes in the BCC pathway. For example, mutation-based functional loss of PTCH1 receptor has been significantly detected in most BCCs [2, 8]. Upregulated expression of most KmiRNAs that regulate *PTCH1* in this study suggests that suppression of *PTCH1* also induces the development of BCCs and further demonstrates the function of PTCH1 as a tumor suppressor. In addition, the tumor suppressor gene *TP53* is a key regulator of cell division and/or apoptosis, and overexpression of the TP53 protein may suppress its antiapoptotic activity in BCC cells [41]. Therefore, upregulated expression of all KmiRNAs that target *TP53* indicates that miRNAs have an effect on BCC development by regulating *TP53*-mediated apoptosis. This study lacks the evidence from cell culture and/or animal experiments, to show that miRNAs could influence the biological features in BCC; however, these results provide strong correlations between miRNAs and typical BCC-related pathways. Certainly, regulatory relationships of multiple miRNAs-multiple target genes have been found in the basal cell carcinoma pathway, which shows extreme complexity in regulatory networks of miRNAs in BCCs.

## 5. Conclusions

For vital regulators of BCC molecular etiopathogenesis, the present research provides a dataset of DEMs/miRNA candidates which should be verified and examined functionally. Moreover, we are the first to explore the regulatory roles/functions of miRNAs and regulatory networks of miRNAs-target genes in BCC tissues by using sRNA-seq and bioinformatics. Thus, our research facilitates deeper comprehension of miRNA regulatory mechanisms which function in the BCC molecular etiopathogenesis. We found that certain DEMs (e.g., hsa-miR-2113, hsa-miR-9983-3p, and hsa-miR-3619-3p) cannot be directly linked to the BCC processes, as their functions were not previously reported in cancer and other human diseases, and only differences on expression levels do not completely verify their regulatory roles in BCCs, although those miRNAs indeed displayed a remarkable expression variation in this study. Hence, more researches using sRNA-seq and functional genomics are required to verify their roles.

## Figures and Tables

**Table 1:** Details of basal cell carcinoma (BCC) and control specimens.

Sample ID	Localization	Histology	Invasion depth (mm)
1_BCC	Cheek	Solid BCC	2.7
1_control		Nonlesional epithelial skin	n.a.
2_BCC	Cheek	Solid BCC	4.1
2_control		Nonlesional epithelial skin	n.a.
3_BCC	Cheek	Solid BCC	1.8
3_control		Nonlesional epithelial skin	n.a.
4_BCC	Cheek	Solid BCC	2.2
4_control		Nonlesional epithelial skin	n.a.
5_BCC	Cheek	Solid BCC	4.5
5_control		Nonlesional epithelial skin	n.a.
6_BCC	Cheek	Solid BCC	3.4
6_control		Nonlesional epithelial skin	n.a.
7_BCC	Cheek	Solid BCC	1.8
7_control		Nonlesional epithelial skin	n.a.
8_BCC	Cheek	Solid BCC	3.9
8_control		Nonlesional epithelial skin	n.a.
9_BCC	Cheek	Solid BCC	2.4
9_control		Nonlesional epithelial skin	n.a.
10_BCC	Cheek	Solid BCC	3.0
10_control		Nonlesional epithelial skin	n.a.
11_BCC	Cheek	Solid BCC	4.0
11_control		Nonlesional epithelial skin	n.a.
12_BCC	Cheek	Solid BCC	3.7
12_control		Nonlesional epithelial skin	n.a.
13_BCC	Cheek	Solid BCC	2.2
13_control		Nonlesional epithelial skin	n.a.
14_BCC	Cheek	Solid BCC	5.3
14_control		Nonlesional epithelial skin	n.a.
15_BCC	Cheek	Solid BCC	3.8
15_control		Nonlesional epithelial skin	n.a.

**Table 2:** Summary of deep sequencing information for each sRNA sequencing library.

Category	Control groups			Basal cell carcinoma (BCC) groups		
	Control_1	Control_2	Control_3	BBC_1	BBC_2	BBC_3
Total raw tags	28,888,581	29,268,292	29,541,601	27,833,324	29,268,292	30,001,854
Q20 percentage (%)	99.2	99.4	99.2	99.4	99.4	99.4
Total clean tags	25,648,341	23,931,822	25,876,318	23,720,918	23,886,431	25,250,916
Percentage of clean tag (%)	88.78	81.77	87.59	85.22	81.61	84.16
Percentage of miRNA (%)	60.3	64.9	61.8	82.2	78.6	82.9
Total mapped tags to genome (%)	91.87	95.01	95.10	95.96	96.07	95.92
Identified miRNAs	1,997	1,954	1,901	1,982	1,999	1,987
Total miRNAs	1873					

**Table 3:** Top 10 differentially expressed miRNAs (DEMs) in the basal cell carcinoma (BCC) of the skin compared to adjacent noncancerous skin (controls).

miRNA ID	Log_2_fold change	Expression pattern	FDR
hsa-miR-2113	7.92	Up	6.31*E* − 61
hsa-miR-1269b	7.88	Up	6.94*E* − 233
hsa-miR-135a-3p	6.51	Up	6.28*E* − 14
hsa-miR-551a	5.66	Up	3.71*E* − 57
hsa-miR-548y	5.26	Up	1.91*E* − 22
hsa-miR-548i	4.92	Up	9.51*E* − 10
hsa-miR-9983-3p	4.85	Up	1.87*E* − 05
hsa-miR-3180-3p	3.85	Up	8.58*E* − 60
hsa-miR-675-5p	3.62	Up	4.43*E* − 102
hsa-miR-542-3p	3.28	Up	8.15*E* − 12
hsa-miR-378i	-7.67	Down	5.64*E* − 21
hsa-miR-523-3p	-6.76	Down	1.33*E* − 12
hsa-miR-550b-2-5p	-6.45	Down	1.58*E* − 19
hsa-miR-516a-5p	-5.83	Down	3.31*E* − 84
hsa-miR-3619-3p	-5.57	Down	1.48*E* − 06
hsa-miR-1323	-5.31	Down	3.75*E* − 26
hsa-miR-517a-3p	-5.20	Down	1.43*E* − 220
hsa-miR-203a-5p	-5.16	Down	2.77*E* − 16
hsa-miR-520h	-4.89	Down	1.10*E* − 37
hsa-miR-526b-5p	-4.75	Down	6.72*E* − 34

**Table 4:** List of KEGG pathways of target genes of differentially expressed miRNAs (DEMs) in the basal cell carcinoma (BCC) of the skin compared to controls. ^∗^The pathways related to cancer.

Pathway	Pathway ID	FDR
Protein digestion and absorption	ko04974	1.42*E* − 11
Focal adhesion	ko04510	3.80*E* − 06
Notch signaling pathway	ko04330	3.72*E* − 05
Fc gamma R-mediated phagocytosis	ko04666	1.76*E* − 04
RNA transport	ko03013	2.16*E* − 04
Basal cell carcinoma^∗^	ko05217	2.61*E* − 04
Chemokine signaling pathway	ko04062	3.05*E* − 04
Histidine metabolism	ko00340	3.28*E* − 04
Breast cancer^∗^	ko05224	3.63*E* − 04
Hepatocellular carcinoma^∗^	ko05225	4.86*E* − 04
Beta-alanine metabolism	ko00410	5.00*E* − 04
IL-17 signaling pathway	ko04657	5.38*E* − 04
Gastric cancer^∗^	ko05226	5.83*E* − 04
Natural killer cell-mediated cytotoxicity	ko04650	6.15*E* − 04
Mineral absorption	ko04978	6.54*E* − 04
PI3K-Akt signaling pathway	ko04151	6.93*E* − 04
Chronic myeloid leukemia^∗^	ko05220	6.99*E* − 04
Mucin type O-glycan biosynthesis	ko00512	7.41*E* − 04
Small-cell lung cancer^∗^	ko05222	8.06*E* − 04
Th1 and Th2 cell differentiation	ko04658	8.18*E* − 04
Cytokine-cytokine receptor interaction	ko04060	1.33*E* − 03
Complement and coagulation cascades	ko04610	1.63*E* − 03
B cell receptor signaling pathway	ko04662	2.14*E* − 03
MicroRNAs in cancer^∗^	ko05206	3.07*E* − 03
Th17 cell differentiation	ko04659	3.20*E* − 03
TNF signaling pathway	ko04668	5.74*E* − 03
Prostate cancer^∗^	ko05215	7.70*E* − 03
Bladder cancer^∗^	ko05219	7.76*E* − 03
T cell receptor signaling pathway	ko04660	8.26*E* − 03
Citrate cycle (TCA cycle)	ko00020	8.80*E* − 03
NF-kappa B signaling pathway	ko04064	8.85*E* − 03
C-type lectin receptor signaling pathway	ko04625	9.03*E* − 03
Leukocyte transendothelial migration	ko04670	9.06*E* − 03
Pancreatic cancer^∗^	ko05212	9.07*E* − 03
Oxidative phosphorylation	ko00190	9.10*E* − 03
NOD-like receptor signaling pathway	ko04621	9.12*E* − 03
Toll-like receptor signaling pathway	ko04620	9.27*E* − 03
Apoptosis	ko04210	9.70*E* − 03

## Data Availability

The data used to support the findings of this study is available from the corresponding authors upon reasonable request.

## Abbreviations

BCC: Basal cell carcinoma
DEMs: Differentially expressed miRNAs
sRNA-seq: Small RNA sequencing
qPCR: TaqMan quantitative real-time PCR
TPM: Transcripts per million kilobase
NR: NCBI nonredundant protein
DAVID: Database for Annotation, Visualization, and Integrated Discovery
GO: Gene Ontology
KEGG: Kyoto Encyclopedia of Genes and Genomes
SNPs: Single nucleotide polymorphisms.

## Supplementary Materials

Supplementary Materials Supplementary Figure 1: length distribution of sRNA tags in six sequencing libraries. Supplementary Figure 2: KEGG pathway of basal cell carcinoma and 24 miRNA-regulated gene members marked in a red box. Supplementary Table 1: information of TaqMan probes used in quantitative PCR. Supplementary Table 2: list of miRNAs identified in this study. Supplementary Table 3: differentially expressed miRNAs (DEMs) in the BCCs when compared with control. Supplementary Table 4: list of the enriched “biological process” GO terms of targeted genes of DEMs between the control and BCC groups. Supplementary Table 5: miRNA-targeted gene analysis in the basal cell carcinoma (ko05217) pathway. u: upregulation; d: downregulation.
