# Supplementary material for: Genome-Wide Expression Difference of MicroRNAs in Basal Cell Carcinoma
Source: J Immunol Res. 2021 Aug 4;2021:7223500. doi: 10.1155/2021/7223500 (PMC8357504; doi:10.1155/2021/7223500)
Supplement: Supplementary Materials — Supplementary Figure 1: length distribution of sRNA tags in six sequencing libraries. Supplementary Figure 2: KEGG pathway of basal cell carcinoma and 24 miRNA-regulated gene members marked in a red box. Supplementary Table 1: information of TaqMan probes used in quantitative PCR. Supplementary Table 2: list of miRNAs identified in this study. Supplementary Table 3: differentially expressed miRNAs (DEMs) in the BCCs when compared with control. Supplementary Table 4: list of the enriched “biological process” GO terms of targeted genes of DEMs between the control and BCC groups. Supplementary Table 5: miRNA-targeted gene analysis in the basal cell carcinoma (ko05217) pathway. u: upregulation; d: downregulation. [file 7223500.f1.zip › 7223500.f3.docx]

| **Table S1. Information of TaqMan probes used in quantitative PCR.** | | | |
| --- | --- | --- | --- |
| **miRNAs ID** | **Sequence** | **ID** | **Website** |
| hsa-miR-1-3p | UGGAAUGUAAAGAAGUAUGUAU | 477820_mir | https://www.thermofisher.com/order/genome-database/details/microrna/477820_mir |
| hsa-miR-107 | AGCAGCAUUGUACAGGGCUAUCA | 478254_mir | https://www.thermofisher.com/order/genome-database/details/microrna/478254_mir |
| hsa-miR-10a-3p | CAAAUUCGUAUCUAGGGGAAUA | 478624_mir | https://www.thermofisher.com/order/genome-database/details/microrna/478624_mir |
| hsa-miR-551a | GCGACCCACUCUUGGUUUCCA | 478158_mir | https://www.thermofisher.com/order/genome-database/details/microrna/478158_mir |
| hsa-miR-9983-3p | UUUUUUGCUGGAACAUUUCUGG | 483116_mir | https://www.thermofisher.com/order/genome-database/details/microrna/483116_mir |
| hsa-miR-5695 | ACUCCAAGAAGAAUCUAGACAG | 480154_mir | https://www.thermofisher.com/order/genome-database/details/microrna/480154_mir |
| hsa-miR-941 | CACCCGGCUGTGUGCACAUGUGC | 479217_mir | https://www.thermofisher.com/order/genome-database/details/microrna/479217_mir |
| hsa-miR-615-3p | UCCGAGCCUGGGUCUCCCTCUU | 478175_mir | https://www.thermofisher.com/order/genome-database/details/microrna/478175_mir |
| hsa-miR-3690 | ACCUGGACCCAGCGUAGACAAAG | 462093_mat | https://www.thermofisher.com/order/genome-database/details/microrna/462093_mat |
| hsa-miR-328-3p | CUGGCCCUCUCUGCCCUUCCGU | 000543_mir | https://www.thermofisher.com/order/genome-database/details/microrna/000543_mir |
| hsa-miR-370-3p | GCCUGCUGGGGUGGAACCUGGU | 002275_mir | https://www.thermofisher.com/order/genome-database/details/microrna/002275_mir |
| hsa-miR-502-3p | AAUGCACCUGGGCAAGGAUUCA | 002083_mir | https://www.thermofisher.com/order/genome-database/details/microrna/002083_mir |
| hsa-miR-509-3-5p | UACUGCAGACGUGGCAAUCAUG | 478963_mir | https://www.thermofisher.com/order/genome-database/details/microrna/478964_mir |
| hsa-miR-548e-3p | AAAAACUGAGACUACUUUUGCA | 478362_mir | https://www.thermofisher.com/order/genome-database/details/microrna/478362_mir |
| hsa-miR-550b-2-5p | AUGUGCCUGAGGGAGUAAGACA | 479033_mir | https://www.thermofisher.com/order/genome-database/details/microrna/479033_mir |
| internal cotrol gene U6 | GTGCTCGCTTCGGCAGCACATATACTAAAATTGGAACGATACAGAGAAGATTAGCATGGCCCCTGCGCAAGGATGACACGCAAATTCGTGAAGCGTTCCATATTTT | 001973 | https://www.thermofisher.com/order/genome-database/details/mirna/001973 |
