# Supplementary material for: Genome-Wide Expression Difference of MicroRNAs in Basal Cell Carcinoma
Source: J Immunol Res. 2021 Aug 4;2021:7223500. doi: 10.1155/2021/7223500 (PMC8357504; doi:10.1155/2021/7223500)
Supplement: Supplementary Materials — Supplementary Figure 1: length distribution of sRNA tags in six sequencing libraries. Supplementary Figure 2: KEGG pathway of basal cell carcinoma and 24 miRNA-regulated gene members marked in a red box. Supplementary Table 1: information of TaqMan probes used in quantitative PCR. Supplementary Table 2: list of miRNAs identified in this study. Supplementary Table 3: differentially expressed miRNAs (DEMs) in the BCCs when compared with control. Supplementary Table 4: list of the enriched “biological process” GO terms of targeted genes of DEMs between the control and BCC groups. Supplementary Table 5: miRNA-targeted gene analysis in the basal cell carcinoma (ko05217) pathway. u: upregulation; d: downregulation. [file 7223500.f1.zip › 7223500.f4.docx]

| **Table S2. List of miRNAs identified in this study.** | | |
| --- | --- | --- |
| **miRNA id** | **mature** | **hairpin** |
| hsa-miR-576-3p | AAGATGTGGAAAAATTGGAATC | TACAATCCAACGAGGATTCTAATTTCTCCACGTCTTTGGTAATAAGGTTTGGCAAAGATGTGGAAAAATTGGAATCCTCATTCGATTGGTTATAACCA |
| hsa-miR-140-5p | CAGTGGTTTTACCCTATGGTAG | TGTGTCTCTCTCTGTGTCCTGCCAGTGGTTTTACCCTATGGTAGGTTACGTCATGCTGTTCTACCACAGGGTAGAACCACGGACAGGATACCGGGGCACC |
| hsa-miR-519c-5p | CTCTAGAGGGAAGCGCTTTCTG | TCTCAGGCTGTGTCCCTCTAGAGGGAAGCGCTTTCTGTTGTCTGAAAGAAAAGAAAATGGTTCCCTTTAGAGTGTTACGCTTTGAGA |
| hsa-miR-1298-5p | TTCATTCGGCTGTCCAGATGTA | AGACGAGGAGTTAAGAGTTCATTCGGCTGTCCAGATGTATCCAAGTACCCTGTGTTATTTGGCAATAAATACATCTGGGCAACTGACTGAACTTTTCACTTTTCATGACTCA |
| hsa-miR-133a-3p | TTTGGTCCCCTTCAACCAGCTG | ACAATGCTTTGCTAGAGCTGGTAAAATGGAACCAAATCGCCTCTTCAATGGATTTGGTCCCCTTCAACCAGCTGTAGCTATGCATTGA |
| hsa-miR-4743-3p | TTTCTGTCTTTTCTGGTCCAG | GCTGGCCGGATGGGACAGGAGGCATGAATGAGCCATCTTTCCAATGCCTTTCTGTCTTTTCTGGTCCAG |
| hsa-miR-548ao-3p | AAAGACCGTGACTACTTTTGCA | AACTATTCTTAGGTTGATGCAGAAGTAACTACGGTTTTTGCAGTTGAAAGTAATGGCAAAGACCGTGACTACTTTTGCAACAGCCTAATAGTTTCT |
| hsa-miR-5088-5p | CAGGGCTCAGGGATTGGATGGAGG | CCCATCAGGGCTCAGGGATTGGATGGAGGTGATGGGGGCAGGGGATGGGTCTCACCCTCCCTTCTTCCTGGGCCCTCAG |
| hsa-miR-4649-5p | TGGGCGAGGGGTGGGCTCTCAGAG | TCTGGGCGAGGGGTGGGCTCTCAGAGGGGCTGGCAGTACTGCTCTGAGGCCTGCCTCTCCCCAG |
| hsa-miR-665 | ACCAGGAGGCTGAGGCCCCT | TCTCCTCGAGGGGTCTCTGCCTCTACCCAGGACTCTTTCATGACCAGGAGGCTGAGGCCCCTCACAGGCGGC |
| hsa-miR-3622b-3p | TCACCTGAGCTCCCGTGCCTG | AGTGATATAATAGAGGGTGCACAGGCATGGGAGGTCAGGTGAGCTCAGCTCCCTGCCTCACCTGAGCTCCCGTGCCTGTGCACCCTCTATTGGCT |
| hsa-miR-1250-5p | ACGGTGCTGGATGTGGCCTTT | CTGTCCCGCTGGCCTGGCAGGTGACGGTGCTGGATGTGGCCTTTTTGCCTTTTCTAAAGGCCACATTTTCCAGCCCATTCAACCTTCCAGAGCCCTCTGAAGTGGCCACAGGC |
| hsa-miR-3622a-5p | CAGGCACGGGAGCTCAGGTGAG | AATAGAGGGTGCACAGGCACGGGAGCTCAGGTGAGGCAGGGAGCTGAGCTCACCTGACCTCCCATGCCTGTGCACCCTCTATT |
| hsa-miR-381-3p | TATACAAGGGCAAGCTCTCTGT | TACTTAAAGCGAGGTTGCCCTTTGTATATTCGGTTTATTGACATGGAATATACAAGGGCAAGCTCTCTGTGAGTA |
| hsa-miR-4709-3p | TTGAAGAGGAGGTGCTCTGTAGC | CTGCTTCAACAACAGTGACTTGCTCTCCAATGGTATCCAGTGATTCGTTGAAGAGGAGGTGCTCTGTAGCAG |
| hsa-miR-6738-5p | CGAGGGGTAGAAGAGCACAGGGG | GAAGGCGAGGGGTAGAAGAGCACAGGGGTTCTGATAAACCCTTCTGCCTGCATTCTACTCCCAG |
| hsa-miR-374b-3p | CTTAGCAGGTTGTATTATCATT | ACTCGGATGGATATAATACAACCTGCTAAGTGTCCTAGCACTTAGCAGGTTGTATTATCATTGTCCGTGTCT |
| hsa-miR-6874-3p | CAGTTCTGCTGTTCTGACTCTAG | GCCACATGGAGCTGGAACCAGATCAGGCTTTAATGTTTGAAGTAATGTCAGTTCTGCTGTTCTGACTCTAG |
| hsa-miR-448 | TTGCATATGTAGGATGTCCCAT | GCCGGGAGGTTGAACATCCTGCATAGTGCTGCCAGGAAATCCCTATTTCATATAAGAGGGGGCTGGCTGGTTGCATATGTAGGATGTCCCATCTCCCAGCCCACTTCGTCA |
| hsa-miR-548s | ATGGCCAAAACTGCAGTTATTTT | TTGCTGCAAAAATAATTGCAGTTTTTGCCATTATTTTTAATAATTATAATAATGGCCAAAACTGCAGTTATTTTTGCACCAA |
| hsa-miR-515-5p | TTCTCCAAAAGAAAGCACTTTCTG | TCTCATGCAGTCATTCTCCAAAAGAAAGCACTTTCTGTTGTCTGAAAGCAGAGTGCCTTCTTTTGGAGCGTTACTGTTTGAGA |
| hsa-miR-6891-5p | TAAGGAGGGGGATGAGGGG | GTAAGGAGGGGGATGAGGGGTCATATCTCTTCTCAGGGAAAGCAGGAGCCCTTCAGCAGGGTCAGGGCCCCTCATCTTCCCCTCCTTTCCCAG |
| hsa-miR-431-3p | CAGGTCGTCTTGCAGGGCTTCT | TCCTGCTTGTCCTGCGAGGTGTCTTGCAGGCCGTCATGCAGGCCACACTGACGGTAACGTTGCAGGTCGTCTTGCAGGGCTTCTCGCAAGACGACATCCTCATCACCAACGACG |
| hsa-miR-6832-5p | AGTAGAGAGGAAAAGTTAGGGTC | GGTGGAGTAGAGAGGAAAAGTTAGGGTCAGTGGCAGAGCCAGGCAGATGCTGACCCTTTTTCTCTTTCCCAG |
| hsa-miR-103a-2-5p | AGCTTCTTTACAGTGCTGCCTTG | TTGTGCTTTCAGCTTCTTTACAGTGCTGCCTTGTAGCATTCAGGTCAAGCAGCATTGTACAGGGCTATGAAAGAACCA |
| hsa-miR-6877-5p | AGGGCCGAAGGGTGGAAGCTGC | AGTTCAGGGCCGAAGGGTGGAAGCTGCTGGTGCTCATCTCAGCCTCTGCCCTTGGCCTCCCCAG |
| hsa-miR-1298-3p | CATCTGGGCAACTGACTGAAC | AGACGAGGAGTTAAGAGTTCATTCGGCTGTCCAGATGTATCCAAGTACCCTGTGTTATTTGGCAATAAATACATCTGGGCAACTGACTGAACTTTTCACTTTTCATGACTCA |
| hsa-miR-3682-5p | CTACTTCTACCTGTGTTATCAT | TAAGTTATATATGTCTACTTCTACCTGTGTTATCATAATAAAGGTGTCATGATGATACAGGTGGAGGTAGAAATATATAACTTA |
| hsa-miR-3155a | CCAGGCTCTGCAGTGGGAACT | TCCGGGCATCACCTCCCACTGCAGAGCCTGGGGAGCCGGACAGCTCCCTTCCCAGGCTCTGCAGTGGGAACTGATGCCTGGA |
| hsa-miR-5089-5p | GTGGGATTTCTGAGTAGCATC | AAGGACTTCAGTGGGATTTCTGAGTAGCATCCTTGGAATCTGCACTCAAGGGATGCTACTCGGAAATCCCACTGAAGTCCTTTT |
| hsa-miR-6740-5p | AGTTTGGGATGGAGAGAGGAGA | GAAAGAGTTTGGGATGGAGAGAGGAGAAACTTGAGGTCTCTGGGAGTTGCTTAAACCAGTTGACCGTAACCTGGCCAGAGAATTCTGATAGTGTCTTCTCTCCTCCCAAACAG |
| hsa-miR-450a-2-3p | ATTGGGGACATTTTGCATTCAT | CCAAAGAAAGATGCTAAACTATTTTTGCGATGTGTTCCTAATATGTAATATAAATGTATTGGGGACATTTTGCATTCATAGTTTTGTATCAATAATATGG |
| hsa-miR-6793-3p | TCCCCAACCCCTGCCCGCAG | GTCACTGTGGGTTCTGGGTTGGGGTGATACACAAGCCTGACCCTCCCCAACCCCTGCCCGCAG |
| hsa-miR-3622a-3p | TCACCTGACCTCCCATGCCTGT | AATAGAGGGTGCACAGGCACGGGAGCTCAGGTGAGGCAGGGAGCTGAGCTCACCTGACCTCCCATGCCTGTGCACCCTCTATT |
| hsa-miR-541-5p | AAAGGATTCTGCTGTCGGTCCCACT | ACGTCAGGGAAAGGATTCTGCTGTCGGTCCCACTCCAAAGTTCACAGAATGGGTGGTGGGCACAGAATCTGGACTCTGCTTGTG |
| hsa-miR-6515-3p | TCTCTTCATCTACCCCCCAG | CATTGGAGGGTGTGGAAGACATCTGGGCCAACTCTGATCTCTTCATCTACCCCCCAG |
| hsa-miR-1276 | TAAAGAGCCCTGTGGAGACA | CCCCAGCTAGGTAAAGAGCCCTGTGGAGACACCTGGATTCAGAGAACATGTCTCCACTGAGCACTTGGGCCTTGATGGCGGCT |
| hsa-miR-6499-3p | AGCAGTGTTTGTTTTGCCCACA | TCAGTCGGGCGCAAGAGCACTGCAGTTCTGTTGGGTGACAGCAGTGTTTGTTTTGCCCACAG |
| hsa-miR-2276-3p | TCTGCAAGTGTCAGAGGCGAGG | GTGTTCTTCCAGTCCGCCCTCTGTCACCTTGCAGACGGCTTTCTCTCCGAATGTCTGCAAGTGTCAGAGGCGAGGAGTGGCAGCTGCAT |
| hsa-miR-3120-3p | CACAGCAAGTGTAGACAGGCA | GTCATGTGACTGCCTGTCTGTGCCTGCTGTACAGGTGAGCGGATGTTCTGCACAGCAAGTGTAGACAGGCAGACACATGAC |
| hsa-miR-6780b-5p | TGGGGAAGGCTTGGCAGGGAAGA | CAGCCTGGGGAAGGCTTGGCAGGGAAGACACATGAGCAGTGCCTCCACTTCACGCCTCTCCCTTGTCTCCTTTCCCTAG |
| hsa-miR-1306-5p | CCACCTCCCCTGCAAACGTCCA | GTGAGCAGTCTCCACCACCTCCCCTGCAAACGTCCAGTGGTGCAGAGGTAATGGACGTTGGCTCTGGTGGTGATGGACAGTCCGA |
| hsa-miR-519b-3p | AAAGTGCATCCTTTTAGAGGTT | CATGCTGTGACCCTCTAGAGGGAAGCGCTTTCTGTTGTCTGAAAGAAAAGAAAGTGCATCCTTTTAGAGGTTTACTGTTTG |
| hsa-miR-489-3p | GTGACATCACATATACGGCAGC | GTGGCAGCTTGGTGGTCGTATGTGTGACGCCATTTACTTGAACCTTTAGGAGTGACATCACATATACGGCAGCTAAACTGCTAC |
| hsa-miR-23a-3p | ATCACATTGCCAGGGATTTCC | GGCCGGCTGGGGTTCCTGGGGATGGGATTTGCTTCCTGTCACAAATCACATTGCCAGGGATTTCCAACCGACC |
| hsa-miR-6796-5p | TTGTGGGGTTGGAGAGCTGGCTG | TTACCTTGTGGGGTTGGAGAGCTGGCTGGTCCAGCCCCTCAGAAGCTCTCCCCTCCCCGCAG |
| hsa-miR-675-3p | CTGTATGCCCTCACCGCTCA | CCCAGGGTCTGGTGCGGAGAGGGCCCACAGTGGACTTGGTGACGCTGTATGCCCTCACCGCTCAGCCCCTGGG |
| hsa-miR-2110 | TTGGGGAAACGGCCGCTGAGTG | CAGGGGTTTGGGGAAACGGCCGCTGAGTGAGGCGTCGGCTGTGTTTCTCACCGCGGTCTTTTCCTCCCACTCTTG |
| hsa-miR-1269a | CTGGACTGAGCCGTGCTACTGG | TGGATTGCCTAGACCAGGGAAGCCAGTTGGCATGGCTCAGTCCAAGTCTGACCACCTGAGGAATGCCTGGACTGAGCCGTGCTACTGGCTTCCCTGGTCTCCAGC |
| hsa-miR-483-3p | TCACTCCTCTCCTCCCGTCTT | GAGGGGGAAGACGGGAGGAAAGAAGGGAGTGGTTCCATCACGCCTCCTCACTCCTCTCCTCCCGTCTTCTCCTCTC |
| hsa-miR-4659a-3p | TTTCTTCTTAGACATGGCAACG | GAAACTGCTGAAGCTGCCATGTCTAAGAAGAAAACTTTGGAGAAAAATTTTCTTCTTAGACATGGCAACGTCAACAGTTTC |
| hsa-miR-1912-3p | TACCCAGAGCATGCAGTGTGAA | CTCTAGGATGTGCTCATTGCATGGGCTGTGTATAGTATTATTCAATACCCAGAGCATGCAGTGTGAACATAATAGAGATT |
| hsa-miR-146a-5p | TGAGAACTGAATTCCATGGGTT | CCGATGTGTATCCTCAGCTTTGAGAACTGAATTCCATGGGTTGTGTCAGTGTCAGACCTCTGAAATTCAGTTCTTCAGCTGGGATATCTCTGTCATCGT |
| hsa-miR-1289 | TGGAGTCCAGGAATCTGCATTTT | TTCTCAATTTTTAGTAGGAATTAAAAACAAAACTGGTAAATGCAGACTCTTGGTTTCCACCCCCAGAGAATCCCTAAACCGGGGGTGGAGTCCAGGAATCTGCATTTTAGAAAGTACCCAGGGTGATTCTGATAATTGGGAACA |
| hsa-miR-8485 | CACACACACACACACACGTAT | TCTGTGATATACGTGTGTGTGTGTGTGTATATAGCATATGTGTATACATACACACACACACACACACACACACACACACACACACACGTAT |
| hsa-miR-425-3p | ATCGGGAATGTCGTGTCCGCCC | GAAAGCGCTTTGGAATGACACGATCACTCCCGTTGAGTGGGCACCCGAGAAGCCATCGGGAATGTCGTGTCCGCCCAGTGCTCTTTC |
| hsa-miR-4474-3p | TTGTGGCTGGTCATGAGGCTAA | TTGCCTACCTTGTTAGTCTCATGATCAGACACAAATATGGCTCTTTGTGGCTGGTCATGAGGCTAACAAGGTAGGCAC |
| hsa-miR-5002-5p | AATTTGGTTTCTGAGGCACTTAGT | TCTTCCTCTCTGTCCTCTGGAATTTGGTTTCTGAGGCACTTAGTAGGTGATAGCATGACTGACTGCCTCACTGACCACTTCCAGATGAGGGTTACTC |
| hsa-let-7a-3p | CTATACAATCTACTGTCTTTC | TGGGATGAGGTAGTAGGTTGTATAGTTTTAGGGTCACACCCACCACTGGGAGATAACTATACAATCTACTGTCTTTCCTA |
| hsa-miR-190b-3p | ACTAAATGTCAAACATATTCT | TGCTTCTGTGTGATATGTTTGATATTGGGTTGTTTAATTAGGAACCAACTAAATGTCAAACATATTCTTACAGCAGCAG |
| hsa-miR-10527-5p | AAAGCAAATGTTGGGTGAACGGC | AAAGCAAATGTTGGGTGAACGGCTGTTTCCTCTTATTCAAGCCATGCACCTTACTCTTGCTGGTA |
| hsa-miR-4488 | AGGGGGCGGGCTCCGGCG | GGTAGGGGGCGGGCTCCGGCGCTGGGACCCCACTAGGGTGGCGCCTTGGCCCCGCCCCGCCC |
| hsa-miR-3691-5p | AGTGGATGATGGAGACTCGGTAC | TTGAGGCACTGGGTAGTGGATGATGGAGACTCGGTACCCACTGCTGAGGGTGGGGACCAAGTCTGCGTCATCCTCTCCTCAGTGCCTCAA |
| hsa-miR-3688-5p | AGTGGCAAAGTCTTTCCATAT | TCTTCACTTTCAAGAGTGGCAAAGTCTTTCCATATGTATGTATGTATGTCTGTTACACATATGGAAAGACTTTGCCACTCTTTAAAGTGAAGA |
| hsa-miR-532-3p | CCTCCCACACCCAAGGCTTGCA | CGACTTGCTTTCTCTCCTCCATGCCTTGAGTGTAGGACCGTTGGCATCTTAATTACCCTCCCACACCCAAGGCTTGCAGAAGAGCGAGCCT |
| hsa-miR-4747-5p | AGGGAAGGAGGCTTGGTCTTAG | AGGGAAGGAGGCTTGGTCTTAGCACGGGGTCTAAGGCCCGGGCTTTCCTCCCAG |
| hsa-miR-6813-3p | AACCTTGGCCCCTCTCCCCAG | GTAGGCAGGGGCTGGGGTTTCAGGTTCTCAGTCAGAACCTTGGCCCCTCTCCCCAG |
| hsa-miR-3150b-3p | TGAGGAGATCGTCGAGGTTGG | GAGGGAAAGCAGGCCAACCTCGAGGATCTCCCCAGCCTTGGCGTTCAGGTGCTGAGGAGATCGTCGAGGTTGGCCTGCTTCCCCTC |
| hsa-miR-4797-3p | TCTCAGTAAGTGGCACTCTGT | GACTCAGAAGACAGAGTGCCACTTACTGAAAGGTTTTTTCTCTCAGTAAGTGGCACTCTGTCTTCTGAGTT |
| hsa-miR-4684-5p | CTCTCTACTGACTTGCAACATA | GCACCAGGGGTACCTCTCTACTGACTTGCAACATACATTTGTCTTGGTGTGTTGCAAGTCGGTGGAGACGTACCCTTGGTGC |
| hsa-miR-4638-5p | ACTCGGCTGCGGTGGACAAGT | GACTCGGCTGCGGTGGACAAGTCCGGCTCCAGAACCTGGACACCGCTCAGCCGGCCGCGGCAGGGGTC |
| hsa-miR-6868-3p | TTCCTTCTGTTGTCTGTGCAG | CCAGGACTGGCAGAACACTGAAGCAGCAGGCACCTGCTTCCTTCTGTTGTCTGTGCAG |
| hsa-miR-101-5p | CAGTTATCACAGTGCTGATGCT | TGCCCTGGCTCAGTTATCACAGTGCTGATGCTGTCTATTCTAAAGGTACAGTACTGTGATAACTGAAGGATGGCA |
| hsa-miR-548bc | AAAAACTGTGATTACTTTTGC | TCTTTTGTTACAGGTTGGTGAGAAGTAATCGCGGTTTTTGCCATTACTGCTCATGGCAAAAACTGTGATTACTTTTGCACCAACCTAATATAATATTTGGTCTTCAATTA |
| hsa-miR-6771-3p | CAAACCCCTGTCTACCCGCAG | GGTGCCTCGGGAGGGCATGGGCCAGGCCACATAATGAGCCAAACCCCTGTCTACCCGCAG |
| hsa-miR-6748-3p | TCCTGTCCCTGTCTCCTACAG | TGGTGTGTGGGTGGGAAGGACTGGATTTGAAATGGTCCCACTCCTGACGATCCTGTCCCTGTCTCCTACAG |
| hsa-miR-1248 | ACCTTCTTGTATAAGCACTGTGCTAAA | TTTACCTTCTTGTATAAGCACTGTGCTAAAATTGCAGACACTAGGACCATGTCTTGGTTTTTGCAATAATGCTAGCAGAGTACACACAAGAAGAAAAGTAACAGCA |
| hsa-miR-1306-3p | ACGTTGGCTCTGGTGGTG | GTGAGCAGTCTCCACCACCTCCCCTGCAAACGTCCAGTGGTGCAGAGGTAATGGACGTTGGCTCTGGTGGTGATGGACAGTCCGA |
| hsa-miR-579-5p | TCGCGGTTTGTGCCAGATGACG | CATATTAGGTTAATGCAAAAGTAATCGCGGTTTGTGCCAGATGACGATTTGAATTAATAAATTCATTTGGTATAAACCGCGATTATTTTTGCATCAAC |
| hsa-miR-3917 | GCTCGGACTGAGCAGGTGGG | GGCGCTTTTGTGCGCGCCCGGGTCTGTTGGTGCTCAGAGTGTGGTCAGGCGGCTCGGACTGAGCAGGTGGGTGCGGGGCTCGGAGGAGGCGGC |
| hsa-miR-496 | TGAGTATTACATGGCCAATCTC | CCCAAGTCAGGTACTCGAATGGAGGTTGTCCATGGTGTGTTCATTTTATTTATGATGAGTATTACATGGCCAATCTCCTTTCGGTACTCAATTCTTCTTGGG |
| hsa-miR-320c | AAAAGCTGGGTTGAGAGGGT | AAAAATGAGGCCTTCTCTTCCCAGTTCTTCCCAGAGTCAGGAAAAGCTGGGTTGAGAGGGTAGAAAAAAAAT |
| hsa-miR-6818-3p | TTGTCTCTTGTTCCTCACACAG | CTATTTTGTGTGAGTACAGAGAGCATCTGAATGGGTACAGTTGTTGTCTCTTGTTCCTCACACAG |
| hsa-miR-3162-3p | TCCCTACCCCTCCACTCCCCA | CTGACTTTTTTAGGGAGTAGAAGGGTGGGGAGCATGAACAATGTTTCTCACTCCCTACCCCTCCACTCCCCAAAAAAGTCAG |
| hsa-miR-5705 | TGTTTCGGGGCTCATGGCCTGTG | TCCCCATTTACACAGGCCATGAGCCCCGAAACACCCATCCCAGGATTGCTGATGGGTGTTTCGGGGCTCATGGCCTGTGTAAATGGGGA |
| hsa-miR-6767-3p | CCACGTGCTTCTCTTTCCGCAG | TGAAATCGCAGACAGGGACACATGGAGAACGCCCCCACCAGTTCCCACGTGCTTCTCTTTCCGCAG |
| hsa-miR-4477a | CTATTAAGGACATTTGTGATTC | TCCTCCTCCCATCAATCACAAATGTCCTTAATGGCATTTAAGGATTGCTATTAAGGACATTTGTGATTCACGGGAGGAGGT |
| hsa-miR-424-3p | CAAAACGTGAGGCGCTGCTAT | CGAGGGGATACAGCAGCAATTCATGTTTTGAAGTGTTCTAAATGGTTCAAAACGTGAGGCGCTGCTATACCCCCTCGTGGGGAAGGTAGAAGGTGGGG |
| hsa-miR-1238-5p | GTGAGTGGGAGCCCCAGTGTGTG | GTGAGTGGGAGCCCCAGTGTGTGGTTGGGGCCATGGCGGGTGGGCAGCCCAGCCTCTGAGCCTTCCTCGTCTGTCTGCCCCAG |
| hsa-miR-4799-5p | ATCTAAATGCAGCATGCCAGTC | ACTGCTAATATCTAAATGCAGCATGCCAGTCCTGAGATGCAGGGACTGGCATGCTGCATTTATATATTAGCAGT |
| hsa-miR-3074-5p | GTTCCTGCTGAACTGAGCCAG | GCTCGACTCCTGTTCCTGCTGAACTGAGCCAGTGTGTAAAATGAGAACTGATATCAGCTCAGTAGGCACCGGAGGGCGGGT |
| hsa-miR-95-5p | TCAATAAATGTCTGTTGAATT | AACACAGTGGGCACTCAATAAATGTCTGTTGAATTGAAATGCGTTACATTCAACGGGTATTTATTGAGCACCCACTCTGTG |
| hsa-miR-7157-5p | TCAGCATTCATTGGCACCAGAGA | TCAGCATTCATTGGCACCAGAGATGAAATTGGGACTCCTCTGTGCTACTGGATGAAGAGT |
| hsa-miR-4634 | CGGCGCGACCGGCCCGGGG | GGACAAGGGCGGCGCGACCGGCCCGGGGCTCTTGGGCGGCCGCGTTTCCCCTCC |
| hsa-miR-664b-5p | TGGGCTAAGGGAGATGATTGGGTA | TGGGCTAAGGGAGATGATTGGGTAGAAAGTATTATTCTATTCATTTGCCTCCCAGCCTACA |
| hsa-miR-4525 | GGGGGGATGTGCATGCTGGTT | GTCAGAGGGGGGATGTGCATGCTGGTTGGGGTGGGCTGCCTGTGGACCAATCAGCGTGCACTTCCCCACCCTGAA |
| hsa-miR-486-3p | CGGGGCAGCTCAGTACAGGAT | GCATCCTGTACTGAGCTGCCCCGAGGCCCTTCATGCTGCCCAGCTCGGGGCAGCTCAGTACAGGATAC |
| hsa-miR-449a | TGGCAGTGTATTGTTAGCTGGT | CTGTGTGTGATGAGCTGGCAGTGTATTGTTAGCTGGTTGAATATGTGAATGGCATCGGCTAACATGCAACTGCTGTCTTATTGCATATACA |
| hsa-miR-4765 | TGAGTGATTGATAGCTATGTTC | TGGTGATTTTGAACGTAGCTATCCACCACTCAGCCTGGAAAAAGCTGAGTGATTGATAGCTATGTTCAAAATCACCA |
| hsa-miR-3619-5p | TCAGCAGGCAGGCTGGTGCAGC | ACGGCATCTTTGCACTCAGCAGGCAGGCTGGTGCAGCCCGTGGTGGGGGACCATCCTGCCTGCTGTGGGGTAAGGACGGCTGT |
| hsa-miR-7113-5p | TCCAGGGAGACAGTGTGTGAG | CTCCAGGGAGACAGTGTGTGAGGCCTCTTGCCATGGCCTCCCTGCCCGCCTCTCTGCAG |
| hsa-miR-766-3p | ACTCCAGCCCCACAGCCTCAGC | GCATCCTCAGGACCTGGGCTTGGGTGGTAGGAGGAATTGGTGCTGGTCTTTCATTTTGGATTTGACTCCAGCCCCACAGCCTCAGCCACCCCAGCCAATTGTCATAGGAGC |
| hsa-miR-487b-3p | AATCGTACAGGGTCATCCACTT | TTGGTACTTGGAGAGTGGTTATCCCTGTCCTGTTCGTTTTGCTCATGTCGAATCGTACAGGGTCATCCACTTTTTCAGTATCAA |
| hsa-miR-330-3p | GCAAAGCACACGGCCTGCAGAGA | CTTTGGCGATCACTGCCTCTCTGGGCCTGTGTCTTAGGCTCTGCAAGATCAACCGAGCAAAGCACACGGCCTGCAGAGAGGCAGCGCTCTGCCC |
| hsa-miR-92b-5p | AGGGACGGGACGCGGTGCAGTG | CGGGCCCCGGGCGGGCGGGAGGGACGGGACGCGGTGCAGTGTTGTTTTTTCCCCCGCCAATATTGCACTCGTCCCGGCCTCCGGCCCCCCCGGCCC |
| hsa-miR-6829-3p | TGCCTCCTCCGTGGCCTCAG | CAGCGTGGGCTGCTGAGAAGGGGCAGGGTCCTCCAGCTCATTCCTCCTGCCTCCTCCGTGGCCTCAG |
| hsa-miR-6766-3p | TGATTGTCTTCCCCCACCCTCA | ATGAGCGGGTGGGAGCAGATCTTATTGAGAGTTCCTTCTCCTGCTCCTGATTGTCTTCCCCCACCCTCACAG |
| hsa-miR-5584-5p | CAGGGAAATGGGAAGAACTAGA | CAGGGAAATGGGAAGAACTAGATTTGAATCCAGACCTTTAGTTCTTCCCTTTGCCCAATT |
| hsa-miR-4710 | GGGTGAGGGCAGGTGGTT | GACCGAGTGGGGTGAGGGCAGGTGGTTCTTCCCGAAGCAGCTCTCGCCTCTTCGTC |
| hsa-miR-3910 | AAAGGCATAAAACCAAGACA | CTTTTGCTGTCAGTTTTTCTGTTGCTTGTCTTGGTTTTATGCCTTTTATATCAAGGCACATAAAAGGCATAAAACCAAGACAAGCAACAAAAAAAGGATTGATCACAGAAG |
| hsa-miR-11400 | TCGGCTGTGTATCTCTGTGTC | CAAGGGCGACTCGGCTGTGTATCTCTGTGTCAGAAGCTTAGCCACAGTGTGGCACAGTCGTGTCCTTCCTGCTCACA |
| hsa-miR-10524-5p | CAGGATGCCAGCATAGT | CCAGGATGCCAGCATAGTGAGTTCTGGTGAGGGCTGTTTTCCTGGTT |
| hsa-miR-885-5p | TCCATTACACTACCCTGCCTCT | CCGCACTCTCTCCATTACACTACCCTGCCTCTTCTCCATGAGAGGCAGCGGGGTGTAGTGGATAGAGCACGGGT |
| hsa-miR-4715-3p | GTGCCACCTTAACTGCAGCCAAT | GGGGAATGAAAGTTGGCTGCAGTTAAGGTGGCTAATCAGCTGATGGTGCCACCTTAACTGCAGCCAATTCTAATTCCCC |
| hsa-miR-3124-5p | TTCGCGGGCGAAGGCAAAGTC | GCGGGCTTCGCGGGCGAAGGCAAAGTCGATTTCCAAAAGTGACTTTCCTCACTCCCGTGAAGTCGGC |
| hsa-miR-103a-1-5p | GGCTTCTTTACAGTGCTGCCTTG | TACTGCCCTCGGCTTCTTTACAGTGCTGCCTTGTTGCATATGGATCAAGCAGCATTGTACAGGGCTATGAAGGCATTG |
| hsa-miR-4750-5p | CTCGGGCGGAGGTGGTTGAGTG | CGCTCGGGCGGAGGTGGTTGAGTGCCGACTGGCGCCTGACCCACCCCCTCCCGCAG |
| hsa-miR-191-3p | GCTGCGCTTGGATTTCGTCCCC | CGGCTGGACAGCGGGCAACGGAATCCCAAAAGCAGCTGTTGTCTCCAGAGCATTCCAGCTGCGCTTGGATTTCGTCCCCTGCTCTCCTGCCT |
| hsa-miR-3928-3p | GGAGGAACCTTGGAGCTTCGGC | GCTGAAGCTCTAAGGTTCCGCCTGCGGGCAGGAAGCGGAGGAACCTTGGAGCTTCGGC |
| hsa-miR-6799-5p | GGGGAGGTGTGCAGGGCTGG | GAGGAGGGGAGGTGTGCAGGGCTGGGGTCACTGACTCTGCTTCCCCTGCCCTGCATGGTGTCCCCACAG |
| hsa-miR-20a-3p | ACTGCATTATGAGCACTTAAAG | GTAGCACTAAAGTGCTTATAGTGCAGGTAGTGTTTAGTTATCTACTGCATTATGAGCACTTAAAGTACTGC |
| hsa-miR-6787-3p | TCTCAGCTGCTGCCCTCTCCAG | TCGGCTGGCGGGGGTAGAGCTGGCTGCAGGCCCGGCCCCTCTCAGCTGCTGCCCTCTCCAG |
| hsa-miR-1915-3p | CCCCAGGGCGACGCGGCGGG | TGAGAGGCCGCACCTTGCCTTGCTGCCCGGGCCGTGCACCCGTGGGCCCCAGGGCGACGCGGCGGGGGCGGCCCTAGCGA |
| hsa-miR-603 | CACACACTGCAATTACTTTTGC | GATTGATGCTGTTGGTTTGGTGCAAAAGTAATTGCAGTGCTTCCCATTTAAAAGTAATGGCACACACTGCAATTACTTTTGCTCCAACTTAATACTT |
| hsa-miR-32-5p | TATTGCACATTACTAAGTTGCA | GGAGATATTGCACATTACTAAGTTGCATGTTGTCACGGCCTCAATGCAATTTAGTGTGTGTGATATTTTC |
| hsa-miR-5706 | TTCTGGATAACATGCTGAAGCT | AGCTAGGTCTTCTGGATAACATGCTGAAGCTTCTACGTCATTCAGCACTTGCTTCAGCATGTTTTCCAGAGGATCTAGCT |
| hsa-miR-1301-3p | TTGCAGCTGCCTGGGAGTGACTTC | GGATTGTGGGGGGTCGCTCTAGGCACCGCAGCACTGTGCTGGGGATGTTGCAGCTGCCTGGGAGTGACTTCACACAGTCCTC |
| hsa-miR-3938 | AATTCCCTTGTAGATAACCCGG | AGGAATTTTTAACCCGATCACTAGATTATCTACAAGGGAATTTTTTTTTAATTTAAAAAATTCCCTTGTAGATAACCCGGTGGTCAGGTTGGATGGCTCCATG |
| hsa-miR-7112-3p | TGCATCACAGCCTTTGGCCCTAG | ACGGGCAGGGCAGTGCACCCTGCAGGTGAGAGCGGGAACACCTGCATCACAGCCTTTGGCCCTAG |
| hsa-miR-370-3p | GCCTGCTGGGGTGGAACCTGGT | AGACAGAGAAGCCAGGTCACGTCTCTGCAGTTACACAGCTCACGAGTGCCTGCTGGGGTGGAACCTGGTCTGTCT |
| hsa-miR-574-3p | CACGCTCATGCACACACCCACA | GGGACCTGCGTGGGTGCGGGCGTGTGAGTGTGTGTGTGTGAGTGTGTGTCGCTCCGGGTCCACGCTCATGCACACACCCACACGCCCACACTCAGG |
| hsa-miR-23a-5p | GGGGTTCCTGGGGATGGGATTT | GGCCGGCTGGGGTTCCTGGGGATGGGATTTGCTTCCTGTCACAAATCACATTGCCAGGGATTTCCAACCGACC |
| hsa-miR-372-3p | AAAGTGCTGCGACATTTGAGCGT | GTGGGCCTCAAATGTGGAGCACTATTCTGATGTCCAAGTGGAAAGTGCTGCGACATTTGAGCGTCAC |
| hsa-miR-4479 | CGCGCGGCCGTGCTCGGAGCAG | GAAACCAAGTCCGAGCGTGGCTGGCGCGGGAAAGTTCGGGAACGCGCGCGGCCGTGCTCGGAGCAGCGCCA |
| hsa-miR-4326 | TGTTCCTCTGTCTCCCAGAC | GCTGCTCTGCTGTTCCTCTGTCTCCCAGACTCTGGGTGGATGGAGCAGGTCGGGGGCCA |
| hsa-let-7b-5p | TGAGGTAGTAGGTTGTGTGGTT | CGGGGTGAGGTAGTAGGTTGTGTGGTTTCAGGGCAGTGATGTTGCCCCTCGGAAGATAACTATACAACCTACTGCCTTCCCTG |
| hsa-miR-6751-5p | TTGGGGGTGAGGTTGGTGTCTGG | TCTTCTTGGGGGTGAGGTTGGTGTCTGGCCCCAGCAGCCCAGACTGAGCCTCTCTCTCTCCAG |
| hsa-miR-3192-3p | CTCTGATCGCCCTCTCAGCTC | GGAAGGGATTCTGGGAGGTTGTAGCAGTGGAAAAAGTTCTTTTCTTCCTCTGATCGCCCTCTCAGCTCTTTCCTTCT |
| hsa-miR-6856-3p | TACAGCCCTGTGATCTTTCCAG | TGGAAAAGAGAGGAGCAGTGGTGCTGTGGCAGTGGCAGAGGTCGCTACAGCCCTGTGATCTTTCCAG |
| hsa-miR-6807-5p | GTGAGCCAGTGGAATGGAGAGG | GTGAGCCAGTGGAATGGAGAGGCTGTGGGCAGGGGGAGATGTGAAGGAAAGAACTAGGACCCATTCATCCACTGCATTCCTGCTTGGCCCAG |
| hsa-miR-6735-5p | CAGGGCAGAGGGCACAGGAATCTGA | GCAGCCAGGGCAGAGGGCACAGGAATCTGAGGTGACTGGCACAGAAGACTCAGGCCTGTGGCTCCTCCCTCAG |
| hsa-miR-5680 | GAGAAATGCTGGACTAATCTGC | GCATTGGGTTAGCAGGTTAGCCCAGCATTTCCCTTCCTGGACACACAGGAGGAGAAATGCTGGACTAATCTGCTAATCCAATGC |
| hsa-miR-30a-5p | TGTAAACATCCTCGACTGGAAG | GCGACTGTAAACATCCTCGACTGGAAGCTGTGAAGCCACAGATGGGCTTTCAGTCGGATGTTTGCAGCTGC |
| hsa-miR-6844 | TTCTTTGTTTTTAATTCACAG | GAACTTAAGAATTTTGTAGAAATCAAGCTATTTGCTAAAAGTTCTTTGTTTTTAATTCACAG |
| hsa-miR-3189-3p | CCCTTGGGTCTGATGGGGTAG | GCCTCAGTTGCCCCATCTGTGCCCTGGGTAGGAATATCCTGGATCCCCTTGGGTCTGATGGGGTAGCCGATGC |
| hsa-miR-6880-5p | TGGTGGAGGAAGAGGGCAGCTC | GAGGGTGGTGGAGGAAGAGGGCAGCTCCCATGACTGCCTGACCGCCTTCTCTCCTCCCCCAG |
| hsa-miR-6805-3p | TTGCTCTGCTCCCCCGCCCCCAG | TGGCCTAGGGGGCGGCTTGTGGAGTGTATGGGCTGAGCCTTGCTCTGCTCCCCCGCCCCCAG |
| hsa-miR-4689 | TTGAGGAGACATGGTGGGGGCC | GGTTTCTCCTTGAGGAGACATGGTGGGGGCCGGTCAGGCAGCCCATGCCATGTGTCCTCATGGAGAGGCC |
| hsa-miR-136-3p | CATCATCGTCTCAAATGAGTCT | TGAGCCCTCGGAGGACTCCATTTGTTTTGATGATGGATTCTTATGCTCCATCATCGTCTCAAATGAGTCTTCAGAGGGTTCT |
| hsa-miR-519a-3p | AAAGTGCATCCTTTTAGAGTGT | CTCAGGCTGTGACACTCTAGAGGGAAGCGCTTTCTGTTGTCTGAAAGAAAGGAAAGTGCATCCTTTTAGAGTGTTACTGTTTGAG |
| hsa-miR-5003-3p | TACTTTTCTAGGTTGTTGGGG | ATGAGTTTGCTTTGTGTCATCCTCACAACAACCTTGCAGGGTAGAGATGATTTTTCCTACTTTTCTAGGTTGTTGGGGGCTGGGGCAGGGGGAACAGAG |
| hsa-miR-576-5p | ATTCTAATTTCTCCACGTCTTT | TACAATCCAACGAGGATTCTAATTTCTCCACGTCTTTGGTAATAAGGTTTGGCAAAGATGTGGAAAAATTGGAATCCTCATTCGATTGGTTATAACCA |
| hsa-miR-371a-3p | AAGTGCCGCCATCTTTTGAGTGT | GTGGCACTCAAACTGTGGGGGCACTTTCTGCTCTCTGGTGAAAGTGCCGCCATCTTTTGAGTGTTAC |
| hsa-miR-6762-3p | TGGCTGCTTCCCTTGGTCTCCAG | AGAGCCGGGGCCATGGAGCAGCCTGTGTAGACGGGGACCTGCCCTGCATGGGCACCCCCTCACTGGCTGCTTCCCTTGGTCTCCAG |
| hsa-miR-586 | TATGCATTGTATTTTTAGGTCC | ATGGGGTAAAACCATTATGCATTGTATTTTTAGGTCCCAATACATGTGGGCCCTAAAAATACAATGCATAATGGTTTTTCACTCTTTATCTTCTTAT |
| hsa-miR-6791-3p | TGCCTCCTTGGTCTCCGGCAG | CCAGACCCCTGGGGCTGGGCAGGCGGAAAGAGGTCTGAACTGCCTCTGCCTCCTTGGTCTCCGGCAG |
| hsa-miR-4664-5p | TGGGGTGCCCACTCCGCAAGTT | GTTGGGGGCTGGGGTGCCCACTCCGCAAGTTATCACTGAGCGACTTCCGGTCTGTGAGCCCCGTCCTCCGC |
| hsa-miR-3150a-5p | CAACCTCGACGATCTCCTCAGC | GGGAAGCAGGCCAACCTCGACGATCTCCTCAGCACCTGAACGCCAAGGCTGGGGAGATCCTCGAGGTTGGCCTGCTTTCC |
| hsa-miR-3692-3p | GTTCCACACTGACACTGCAGAAGT | CCATTCCTGCTGGTCAGGAGTGGATACTGGAGCAATAGATACAGTTCCACACTGACACTGCAGAAGTGG |
| hsa-miR-338-5p | AACAATATCCTGGTGCTGAGTG | TCTCCAACAATATCCTGGTGCTGAGTGATGACTCAGGCGACTCCAGCATCAGTGATTTTGTTGAAGA |
| hsa-miR-543 | AAACATTCGCGGTGCACTTCTT | TACTTAATGAGAAGTTGCCCGTGTTTTTTTCGCTTTATTTGTGACGAAACATTCGCGGTGCACTTCTTTTTCAGTATC |
| hsa-miR-151b | TCGAGGAGCTCACAGTCT | ACCTCTGATGTGTCAGTCTCTCTTCAGGGCTCCCGAGACACAGAAACAGACACCTGCCCTCGAGGAGCTCACAGTCTAGACAAACAAACCCAGGGT |
| hsa-miR-211-3p | GCAGGGACAGCAAAGGGGTGC | TCACCTGGCCATGTGACTTGTGGGCTTCCCTTTGTCATCCTTCGCCTAGGGCTCTGAGCAGGGCAGGGACAGCAAAGGGGTGCTCAGTTGTCACTTCCCACAGCACGGAG |
| hsa-miR-96-5p | TTTGGCACTAGCACATTTTTGCT | TGGCCGATTTTGGCACTAGCACATTTTTGCTTGTGTCTCTCCGCTCTGAGCAATCATGTGCAGTGCCAATATGGGAAA |
| hsa-miR-5579-5p | TATGGTACTCCTTAAGCTAAC | TATGGTACTCCTTAAGCTAACAGGCCCCTGTCACCATTAGCTTAAGGAGTACCAGATC |
| hsa-miR-887-3p | GTGAACGGGCGCCATCCCGAGG | GTGCAGATCCTTGGGAGCCCTGTTAGACTCTGGATTTTACACTTGGAGTGAACGGGCGCCATCCCGAGGCTTTGCACAG |
| hsa-miR-3689a-5p | TGTGATATCATGGTTCCTGGGA | CCTGGGAGGTGTGATATCATGGTTCCTGGGAGGTGTGATCCTGTGCTTCCTGGGAGGTGTGATATCGTGGTTCCTGGG |
| hsa-miR-618 | AAACTCTACTTGTCCTTCTGAGT | CTCTTGTTCACAGCCAAACTCTACTTGTCCTTCTGAGTGTAATTACGTACATGCAGTAGCTCAGGAGACAAGCAGGTTTACCCTGTGGATGAGTCTGA |
| hsa-miR-6504-3p | CATTACAGCACAGCCATTCT | GCAGTCTGGCTGTGCTGTAATGCAGTCTGCACCCTGCTGCATTACAGCACAGCCATTCTCT |
| hsa-miR-1228-3p | TCACACCTGCCTCGCCCCCC | GTGGGCGGGGGCAGGTGTGTGGTGGGTGGTGGCCTGCGGTGAGCAGGGCCCTCACACCTGCCTCGCCCCCCAG |
| hsa-miR-491-5p | AGTGGGGAACCCTTCCATGAGG | TTGACTTAGCTGGGTAGTGGGGAACCCTTCCATGAGGAGTAGAACACTCCTTATGCAAGATTCCCTTCTACCTGGCTGGGTTGG |
| hsa-miR-4685-3p | TCTCCCTTCCTGCCCTGGCTAG | TAGCCCAGGGCTTGGAGTGGGGCAAGGTTGTTGGTGATATGGCTTCCTCTCCCTTCCTGCCCTGGCTAG |
| hsa-miR-548al | AACGGCAATGACTTTTGTACCA | GGTCGGTGCAAAAGTAATTGCTGTTTTTGCCATTAAAAATAATGGCATTAAAAGTAATGGCAAAAACGGCAATGACTTTTGTACCAATCTAATATCT |
| hsa-miR-6765-3p | TCACCTGGCTGGCCCGCCCAG | GTGAGGCGGGGCCAGGAGGGTGTGTGGCGTGGGTGCTGCGGGGCCGTCAGGGTGCCTGCGGGACGCTCACCTGGCTGGCCCGCCCAG |
| hsa-miR-511-3p | AATGTGTAGCAAAAGACAGA | CAATAGACACCCATCGTGTCTTTTGCTCTGCAGTCAGTAAATATTTTTTTGTGAATGTGTAGCAAAAGACAGAATGGTGGTCCATTG |
| hsa-miR-4286 | ACCCCACTCCTGGTACC | TACTTATGGCACCCCACTCCTGGTACCATAGTCATAAGTTAGGAGATGTTAGAGCTGTGAGTACCATGACTTAAGTGTGGTGGCTTAAACATG |
| hsa-miR-627-3p | TCTTTTCTTTGAGACTCACT | TACTTATTACTGGTAGTGAGTCTCTAAGAAAAGAGGAGGTGGTTGTTTTCCTCCTCTTTTCTTTGAGACTCACTACCAATAATAAGAAATACTACTA |
| hsa-miR-124-5p | CGTGTTCACAGCGGACCTTGAT | AGGCCTCTCTCTCCGTGTTCACAGCGGACCTTGATTTAAATGTCCATACAATTAAGGCACGCGGTGAATGCCAAGAATGGGGCTG |
| hsa-miR-6887-3p | TCCCCTCCACTTTCCTCCTAG | GAGAATGGGGGGACAGATGGAGAGGACACAGGCTGGCACTGAGGTCCCCTCCACTTTCCTCCTAG |
| hsa-miR-641 | AAAGACATAGGATAGAGTCACCTC | TGGGTGAAAGGAAGGAAAGACATAGGATAGAGTCACCTCTGTCCTCTGTCCTCTACCTATAGAGGTGACTGTCCTATGTCTTTCCTTCCTCTTACCCCT |
| hsa-miR-1285-3p | TCTGGGCAACAAAGTGAGACCT | TGTAGAGATAGGATCTCACTTTGTTGCCCAGGCTGGTCTCAAACTCCTGGTCTGGGCAACAAAGTGAGACCTTATCTCTACAAG |
| hsa-miR-30a-3p | CTTTCAGTCGGATGTTTGCAGC | GCGACTGTAAACATCCTCGACTGGAAGCTGTGAAGCCACAGATGGGCTTTCAGTCGGATGTTTGCAGCTGC |
| hsa-miR-5006-5p | TTGCCAGGGCAGGAGGTGGAA | AACCATTAGGGGGCTGTGGTTTGCCAGGGCAGGAGGTGGAAGGGAGCCCCATTTACAGTGGTAACTTCCTTTCCCTTTCCATCCTGGCAGGCTTCAGAGAACTTTACCAG |
| hsa-miR-525-5p | CTCCAGAGGGATGCACTTTCT | CTCAAGCTGTGACTCTCCAGAGGGATGCACTTTCTCTTATGTGAAAAAAAAGAAGGCGCTTCCCTTTAGAGCGTTACGGTTTGGG |
| hsa-miR-6733-3p | TCAGTGTCTGGATTTCCTAG | GTGCTTGGGAAAGACAAACTCAGAGTTCCCTTCTTGTGAGCTCAGTGTCTGGATTTCCTAG |
| hsa-miR-203a-5p | AGTGGTTCTTAACAGTTCAACAGTT | GTGTTGGGGACTCGCGCGCTGGGTCCAGTGGTTCTTAACAGTTCAACAGTTCTGTAGCGCAATTGTGAAATGTTTAGGACCACTAGACCCGGCGGGCGCGGCGACAGCGA |
| hsa-miR-20b-5p | CAAAGTGCTCATAGTGCAGGTAG | AGTACCAAAGTGCTCATAGTGCAGGTAGTTTTGGCATGACTCTACTGTAGTATGGGCACTTCCAGTACT |
| hsa-miR-5699-5p | TGCCCCAACAAGGAAGGACAAG | CTGTACCCCTGCCCCAACAAGGAAGGACAAGAGGTGTGAGCCACACACACGCCTGGCCTCCTGTCTTTCCTTGTTGGAGCAGGGATGTAG |
| hsa-miR-1260a | ATCCCACCTCTGCCACCA | ACCTTTCCAGCTCATCCCACCTCTGCCACCAAAACACTCATCGCGGGGTCAGAGGGAGTGCCAAAAAAGGTAA |
| hsa-miR-5692c | AATAATATCACAGTAGGTGTAC | TATAACATTGTATATACCCACTGTGATATTAAGAGTAATAGCTCTCTAGGTTATTATGAATAATATCACAGTAGGTGTACACAATGTTGTA |
| hsa-miR-6763-3p | CTCCCCGGCCTCTGCCCCCAG | TTCTCCTGGGGAGTGGCTGGGGAGCAGACAGACCCAACCTCATGCTCCCCGGCCTCTGCCCCCAG |
| hsa-miR-92a-1-5p | AGGTTGGGATCGGTTGCAATGCT | CTTTCTACACAGGTTGGGATCGGTTGCAATGCTGTGTTTCTGTATGGTATTGCACTTGTCCCGGCCTGTTGAGTTTGG |
| hsa-miR-548h-3p | CAAAAACCGCAATTACTTTTGCA | GCTATTAGGTTGGTGCAAAAGTAATCGCGGTTTTTGTCATTACTTTAATTACTTTACGTTTCATTAATGACAAAAACCGCAATTACTTTTGCACCAACCTAATACTTGCTA |
| hsa-miR-3680-3p | TTTTGCATGACCCTGGGAGTAGG | AAATTTAAGGAGGGACTCACTCACAGGATTGTGCAAATGCAAAGTTGGCTTTTGCATGACCCTGGGAGTAGGTGCCTCCTTAAATTT |
| hsa-miR-548t-3p | AAAAACCACAATTACTTTTGCACCA | AGGGTGGTGCAAAAGTGATCGTGGTTTTTGCAATTTTTTAATGACAAAAACCACAATTACTTTTGCACCAACCT |
| hsa-miR-4784 | TGAGGAGATGCTGGGACTGA | TGACTGGGCTGAGGAGATGCTGGGACTGAGAGTGTCATGGTGGAGCCTCCGTCCCTGCTCATCCTCTCCGCATGTTG |
| hsa-miR-1273c | GGCGACAAAACGAGACCCTGTC | TGCAGCCTGGGCGACAAAACGAGACCCTGTCTTTTTTTTTTTCTGAGACAGAGTCTCGTTCTGTTGCCCAAGCTGGA |
| hsa-miR-5690 | TCAGCTACTACCTCTATTAGG | CTTTTAATTTCAGCTACTACCTCTATTAGGATTTGGGAGTTATACTAATAGAGGTAATAGTTGAAATTAAGAG |
| hsa-miR-891a-5p | TGCAACGAACCTGAGCCACTGA | CCTTAATCCTTGCAACGAACCTGAGCCACTGATTCAGTAAAATACTCAGTGGCACATGTTTGTTGTGAGGGTCAAAAGA |
| hsa-miR-378f | ACTGGACTTGGAGCCAGAAG | GTCAGGTCCTGGACTCCCATAGTTTTCAGGCTGCTAAACAACAGAACGAGCACTGGACTTGGAGCCAGAAGTCTTGGG |
| hsa-miR-183-3p | GTGAATTACCGAAGGGCCATAA | CCGCAGAGTGTGACTCCTGTTCTGTGTATGGCACTGGTAGAATTCACTGTGAACAGTCTCAGTCAGTGAATTACCGAAGGGCCATAAACAGAGCAGAGACAGATCCACGA |
| hsa-miR-4640-3p | CACCCCCTGTTTCCTGGCCCAC | CTGTGGGCTGGGCCAGGGAGCAGCTGGTGGGTGGGAAGTAAGATCTGACCTGGACTCCATCCCACCCACCCCCTGTTTCCTGGCCCACAG |
| hsa-miR-106b-5p | TAAAGTGCTGACAGTGCAGAT | CCTGCCGGGGCTAAAGTGCTGACAGTGCAGATAGTGGTCCTCTCCGTGCTACCGCACTGTGGGTACTTGCTGCTCCAGCAGG |
| hsa-miR-4712-5p | TCCAGTACAGGTCTCTCATTTC | GACAGGATTCCAGTACAGGTCTCTCATTTCCTTCATGATTAGGAATACTACTTTGAAATGAGAGACCTGTACTGTATCTGTT |
| hsa-miR-379-5p | TGGTAGACTATGGAACGTAGG | AGAGATGGTAGACTATGGAACGTAGGCGTTATGATTTCTGACCTATGTAACATGGTCCACTAACTCT |
| hsa-miR-3126-5p | TGAGGGACAGATGCCAGAAGCA | ATGATTATATGAGGGACAGATGCCAGAAGCACTGGTTATGATTTGCATCTGGCATCCGTCACACAGATAATTAT |
| hsa-miR-1255b-5p | CGGATGAGCAAAGAAAGTGGTT | TACGGATGAGCAAAGAAAGTGGTTTCTTAAAATGGAATCTACTCTTTGTGAAGATGCTGTGAA |
| hsa-miR-6715a-3p | CCAAACCAGTCGTGCCTGTGG | TGGGCTGCGTCCACAGGCACAGCCGGTTTGAGCATTTTTACTGAATTGCCAAACCAGTCGTGCCTGTGGGCACAACCTT |
| hsa-miR-6812-3p | CCGCTCTTCCCCTGACCCCAG | TGAGGATGGGGTGAGATGGGGAGGAGCAGCCAGTCCTGTCTCACCGCTCTTCCCCTGACCCCAG |
| hsa-miR-4680-3p | TCTGAATTGTAAGAGTTGTTA | TATAAGAACTCTTGCAGTCTTAGATGTTATAAAAATATATATCTGAATTGTAAGAGTTGTTAGCAC |
| hsa-let-7g-5p | TGAGGTAGTAGTTTGTACAGTT | AGGCTGAGGTAGTAGTTTGTACAGTTTGAGGGTCTATGATACCACCCGGTACAGGAGATAACTGTACAGGCCACTGCCTTGCCA |
| hsa-miR-4757-3p | CATGACGTCACAGAGGCTTCGC | TTCCAGCCCGAGGCCTCTGTGACGTCACGGTGTCTGCGGGAGGAGACCATGACGTCACAGAGGCTTCGCGCTCTGAG |
| hsa-miR-4731-3p | CACACAAGTGGCCCCCAACACT | CCCTGCCAGTGCTGGGGGCCACATGAGTGTGCAGTCATCCACACACAAGTGGCCCCCAACACTGGCAGGG |
| hsa-miR-4645-5p | ACCAGGCAAGAAATATTGT | TGATAGGGAAACCAGGCAAGAAATATTGTCTCCTCAAGTTGCGACGAGACAGTAGTTCTTGCCTGGTTTCTCTATCA |
| hsa-miR-197-5p | CGGGTAGAGAGGGCAGTGGGAGG | GGCTGTGCCGGGTAGAGAGGGCAGTGGGAGGTAAGAGCTCTTCACCCTTCACCACCTTCTCCACCCAGCATGGCC |
| hsa-miR-34b-3p | CAATCACTAACTCCACTGCCAT | GTGCTCGGTTTGTAGGCAGTGTCATTAGCTGATTGTACTGTGGTGGTTACAATCACTAACTCCACTGCCATCAAAACAAGGCAC |
| hsa-miR-6846-5p | TGGGGGCTGGATGGGGTAGAGT | CAGGCTGGGGGCTGGATGGGGTAGAGTAGGAGAGCCCACTGACCCCTTCTGTCTCCCTAG |
| hsa-miR-519e-5p | TTCTCCAAAAGGGAGCACTTTC | TCTCATGCAGTCATTCTCCAAAAGGGAGCACTTTCTGTTTGAAAGAAAACAAAGTGCCTCCTTTTAGAGTGTTACTGTTTGAGA |
| hsa-miR-6715b-3p | CTCAAACCGGCTGTGCCTGTGG | GGTTGTGCCCACAGGCACGACTGGTTTGGCAATTCAGTAAAAATGCTCAAACCGGCTGTGCCTGTGGACGCAGCCCA |
| hsa-miR-494-5p | AGGTTGTCCGTGTTGTCTTCTCT | GATACTCGAAGGAGAGGTTGTCCGTGTTGTCTTCTCTTTATTTATGATGAAACATACACGGGAAACCTCTTTTTTAGTATC |
| hsa-miR-106a-5p | AAAAGTGCTTACAGTGCAGGTAG | CCTTGGCCATGTAAAAGTGCTTACAGTGCAGGTAGCTTTTTGAGATCTACTGCAATGTAAGCACTTCTTACATTACCATGG |
| hsa-miR-5697 | TCAAGTAGTTTCATGATAAAGG | AGCATATTCTCAAGTAGTTTCATGATAAAGGGTGTATGAGAGATCAACCCTTTATCATGAAACGCTTGAGGATACGCT |
| hsa-miR-4682 | TCTGAGTTCCTGGAGCCTGGTCT | TGCCCCTGGTCTGAGTTCCTGGAGCCTGGTCTGTCACTGGGGAAGTCCAGAGCTCCAAGGCTCAGTGCCCAGGGGACGCA |
| hsa-miR-3692-5p | CCTGCTGGTCAGGAGTGGATACTG | CCATTCCTGCTGGTCAGGAGTGGATACTGGAGCAATAGATACAGTTCCACACTGACACTGCAGAAGTGG |
| hsa-miR-4519 | CAGCAGTGCGCAGGGCTG | AACCTCAGCAGTGCGCAGGGCTGCACTGTCTCCGTCTGCGGCCTGCAGTAAGCGGGTA |
| hsa-miR-6801-5p | TGGTCAGAGGCAGCAGGAAATGA | TGGCCTGGTCAGAGGCAGCAGGAAATGAGAGTTAGCCAGGAGCTTTGCATACTCACCCCTGCCACTCACTGGCCCCCAG |
| hsa-miR-548v | AGCTACAGTTACTTTTGCACCA | AATACTAGGTTTGAGCAAAAGTAATTGCGGTTTTGCCATCATGCCAAAAGCTACAGTTACTTTTGCACCAGCCTAATATT |
| hsa-miR-3661 | TGACCTGGGACTCGGACAGCTG | CACCTTCTCGCAGAGGCTCTTGACCTGGGACTCGGACAGCTGCTTGCACTCGTTCAGCTGCTCGATCCACTGGTCCAGCTCCTTGGTGAACACCTT |
| hsa-miR-4791 | TGGATATGATGACTGAAA | TAAGAACTGGATATGATGACTGAAATAAGCTCCATATCAATGAGAATTTCAATGGGATTATGTGCAGTCAATGTCCAGTAATTA |
| hsa-miR-4638-3p | CCTGGACACCGCTCAGCCGGCCG | GACTCGGCTGCGGTGGACAAGTCCGGCTCCAGAACCTGGACACCGCTCAGCCGGCCGCGGCAGGGGTC |
| hsa-miR-214-5p | TGCCTGTCTACACTTGCTGTGC | GGCCTGGCTGGACAGAGTTGTCATGTGTCTGCCTGTCTACACTTGCTGTGCAGAACATCCGCTCACCTGTACAGCAGGCACAGACAGGCAGTCACATGACAACCCAGCCT |
| hsa-miR-513b-3p | AAATGTCACCTTTTTGAGAGGA | GTGTACAGTGCCTTTCACAAGGAGGTGTCATTTATGTGAACTAAAATATAAATGTCACCTTTTTGAGAGGAGTAATGTACAGCA |
| hsa-miR-6500-3p | ACACTTGTTGGGATGACCTGC | CCTGCCTGCAGAAAGGAGCTATCCACTCCAGGTGTCCTTTCTTCTGAGAGCTGGACACTTGTTGGGATGACCTGCCTGCAGGTAGG |
| hsa-miR-1296-5p | TTAGGGCCCTGGCTCCATCTCC | ACCTACCTAACTGGGTTAGGGCCCTGGCTCCATCTCCTTTAGGAAAACCTTCTGTGGGGAGTGGGGCTTCGACCCTAACCCAGGTGGGCTGT |
| hsa-miR-6892-5p | GTAAGGGACCGGAGAGTAGGA | GTAAGGGACCGGAGAGTAGGAAAAGCAGGGCTCAGGGCCAGAGAGACTGGGCATAGAACTAAGGAGGATGGTGTCCTCCTGACTGCATCTCTCTTCCCTCTCCCACCCCTTGCAG |
| hsa-miR-328-3p | CTGGCCCTCTCTGCCCTTCCGT | TGGAGTGGGGGGGCAGGAGGGGCTCAGGGAGAAAGTGCATACAGCCCCTGGCCCTCTCTGCCCTTCCGTCCCCTG |
| hsa-miR-1291 | TGGCCCTGACTGAAGACCAGCAGT | GGTAGAATTCCAGTGGCCCTGACTGAAGACCAGCAGTTGTACTGTGGCTGTTGGTTTCAAGCAGAGGCCTAAAGGACTGTCTTCCTG |
| hsa-miR-3912-3p | TAACGCATAATATGGACATGT | AGAGAGGAATGAACAGTTAAATTATAACATGTCCATATTATGGGTTAGTTGTGGACACATACTAACGCATAATATGGACATGTTATAATTTAACTGTTCCTTTCT |
| hsa-miR-526a-5p | CTCTAGAGGGAAGCACTTTCTG | CTCAGGCTGTGACCCTCTAGAGGGAAGCACTTTCTGTTGCTTGAAAGAAGAGAAAGCGCTTCCTTTTAGAGGATTACTCTTTGAG |
| hsa-miR-885-3p | AGGCAGCGGGGTGTAGTGGATA | CCGCACTCTCTCCATTACACTACCCTGCCTCTTCTCCATGAGAGGCAGCGGGGTGTAGTGGATAGAGCACGGGT |
| hsa-miR-495-5p | GAAGTTGCCCATGTTATTTTCG | TGGTACCTGAAAAGAAGTTGCCCATGTTATTTTCGCTTTATATGTGACGAAACAAACATGGTGCACTTCTTTTTCGGTATCA |
| hsa-miR-4759 | TAGGACTAGATGTTGGAATTA | CATTTAGGACTAGATGTTGGAATTAGACAGAAAAAAGTTAGACACAAAAAATTGTGTCTAATTCCAACATCTAGTCCTAAATG |
| hsa-miR-141-5p | CATCTTCCAGTACAGTGTTGGA | CGGCCGGCCCTGGGTCCATCTTCCAGTACAGTGTTGGATGGTCTAATTGTGAAGCTCCTAACACTGTCTGGTAAAGATGGCTCCCGGGTGGGTTC |
| hsa-miR-4484 | AAAAGGCGGGAGAAGCCCCA | GGGTTTCCTCTGCCTTTTTTTCCAATGAAAATAACGAAACCTGTTATTTCCCATTGAGGGGGAAAAAGGCGGGAGAAGCCCCA |
| hsa-miR-643 | ACTTGTATGCTAGCTCAGGTAG | ACCAAGTGATATTCATTGTCTACCTGAGCTAGAATACAAGTAGTTGGCGTCTTCAGAGACACTTGTATGCTAGCTCAGGTAGATATTGAATGAAAAA |
| hsa-miR-4676-3p | CACTGTTTCACCACTGGCTCTT | TGAATGAAAGAGCCAGTGGTGAGACAGTGAGTTGATTACTTCTCACTGTTTCACCACTGGCTCTTTGGTTCA |
| hsa-miR-1908-5p | CGGCGGGGACGGCGATTGGTC | CGGGAATGCCGCGGCGGGGACGGCGATTGGTCCGTATGTGTGGTGCCACCGGCCGCCGGCTCCGCCCCGGCCCCCGCCCC |
| hsa-miR-2277-3p | TGACAGCGCCCTGCCTGGCTC | GTGCTTCCTGCGGGCTGAGCGCGGGCTGAGCGCTGCCAGTCAGCGCTCACATTAAGGCTGACAGCGCCCTGCCTGGCTCGGCCGGCGAAGCTC |
| hsa-miR-3162-5p | TTAGGGAGTAGAAGGGTGGGGAG | CTGACTTTTTTAGGGAGTAGAAGGGTGGGGAGCATGAACAATGTTTCTCACTCCCTACCCCTCCACTCCCCAAAAAAGTCAG |
| hsa-miR-551b-3p | GCGACCCATACTTGGTTTCAG | AGATGTGCTCTCCTGGCCCATGAAATCAAGCGTGGGTGAGACCTGGTGCAGAACGGGAAGGCGACCCATACTTGGTTTCAGAGGCTGTGAGAATAA |
| hsa-miR-6893-5p | CAGGCAGGTGTAGGGTGGAGC | CCGGGCAGGCAGGTGTAGGGTGGAGCCCACTGTGGCTCCTGACTCAGCCCTGCTGCCTTCACCTGCCAG |
| hsa-miR-548ae-3p | CAAAAACTGCAATTACTTTCA | GCAGTTTTTGCCATTAAGTTGCGGTTTTTGCCATTATAATGGCAAAAACTGCAATTACTTTCACACCTGC |
| hsa-miR-6821-3p | TGACCTCTCCGCTCCGCACAG | GTGCGTGGTGGCTCGAGGCGGGGGTGGGGGCCTCGCCCTGCTTGGGCCCTCCCTGACCTCTCCGCTCCGCACAG |
| hsa-miR-378i | ACTGGACTAGGAGTCAGAAGG | GGGAGCACTGGACTAGGAGTCAGAAGGTGGAGTTCTGGGTGCTGTTTTCCCACTCTTGGGCCCTGGGCATGTTCTG |
| hsa-miR-323b-5p | AGGTTGTCCGTGGTGAGTTCGCA | TGGTACTCGGAGGGAGGTTGTCCGTGGTGAGTTCGCATTATTTAATGATGCCCAATACACGGTCGACCTCTTTTCGGTATCA |
| hsa-miR-375-3p | TTTGTTCGTTCGGCTCGCGTGA | CCCCGCGACGAGCCCCTCGCACAAACCGGACCTGAGCGTTTTGTTCGTTCGGCTCGCGTGAGGC |
| hsa-miR-518a-5p | CTGCAAAGGGAAGCCCTTTC | TCTCAAGCTGTGACTGCAAAGGGAAGCCCTTTCTGTTGTCTGAAAGAAGAGAAAGCGCTTCCCTTTGCTGGATTACGGTTTGAGA |
| hsa-miR-6514-5p | TATGGAGTGGACTTTCAGCTGGC | TATGGAGTGGACTTTCAGCTGGCATTTACGAGTCAGAGTTCTTACAGAGCTGCCTGTTCTTCCACTCCAG |
| hsa-miR-3615 | TCTCTCGGCTCCTCGCGGCTC | GACTCTGGGACGCTCAGACGCCGCGCGGGGCGGGGATTGGTCTGTGGTCCTCTCTCGGCTCCTCGCGGCTCGCGGCGGCCGACGGTT |
| hsa-miR-770-5p | TCCAGTACCACGTGTCAGGGCCA | AGGAGCCACCTTCCGAGCCTCCAGTACCACGTGTCAGGGCCACATGAGCTGGGCCTCGTGGGCCTGATGTGGTGCTGGGGCCTCAGGGGTCTGCTCTT |
| hsa-miR-4508 | GCGGGGCTGGGCGCGCG | AGGACCCAGCGGGGCTGGGCGCGCGGAGCAGCGCTGGGTGCAGCGCCTGCGCCGGCAGCTGCAAGGGCCG |
| hsa-miR-4521 | GCTAAGGAAGTCCTGTGCTCAG | TCGGCTAAGGAAGTCCTGTGCTCAGTTTTGTAGCATCAAAACTAGGATTTCTCTTGTTAC |
| hsa-miR-33a-5p | GTGCATTGTAGTTGCATTGCA | CTGTGGTGCATTGTAGTTGCATTGCATGTTCTGGTGGTACCCATGCAATGTTTCCACAGTGCATCACAG |
| hsa-miR-1287-5p | TGCTGGATCAGTGGTTCGAGTC | GTTGTGCTGTCCAGGTGCTGGATCAGTGGTTCGAGTCTGAGCCTTTAAAAGCCACTCTAGCCACAGATGCAGTGATTGGAGCCATGACAA |
| hsa-miR-154-5p | TAGGTTATCCGTGTTGCCTTCG | GTGGTACTTGAAGATAGGTTATCCGTGTTGCCTTCGCTTTATTTGTGACGAATCATACACGGTTGACCTATTTTTCAGTACCAA |
| hsa-miR-1912-5p | CTCATTGCATGGGCTGTGTATA | CTCTAGGATGTGCTCATTGCATGGGCTGTGTATAGTATTATTCAATACCCAGAGCATGCAGTGTGAACATAATAGAGATT |
| hsa-miR-6857-3p | TGACTGAGCTTCTCCCCACAG | GCTTGTTGGGGATTGGGTCAGGCCAGTGTTCAAGGGCCCCTCCTCTAGTACTCCCTGTTTGTGTTCTGCCACTGACTGAGCTTCTCCCCACAG |
| hsa-miR-449b-3p | CAGCCACAACTACCCTGCCACT | TGACCTGAATCAGGTAGGCAGTGTATTGTTAGCTGGCTGCTTGGGTCAAGTCAGCAGCCACAACTACCCTGCCACTTGCTTCTGGATAAATTCTTCT |
| hsa-miR-3617-3p | CATCAGCACCCTATGTCCTTTCT | AGGTCATAGAAAGACATAGTTGCAAGATGGGATTAGAAACCATATGTCTCATCAGCACCCTATGTCCTTTCTCTGCCCT |
| hsa-miR-216b-5p | AAATCTCTGCAGGCAAATGTGA | GCAGACTGGAAAATCTCTGCAGGCAAATGTGATGTCACTGAGGAAATCACACACTTACCCGTAGAGATTCTACAGTCTGACA |
| hsa-miR-6817-3p | TCTCTCTGACTCCATGGCA | AGGATTCTGCCATAGGAAGCTTGGAGTGGAACTGACCTGCCCCCTTTCTCTCTGACTCCATGGCAG |
| hsa-miR-1226-3p | TCACCAGCCCTGTGTTCCCTAG | GTGAGGGCATGCAGGCCTGGATGGGGCAGCTGGGATGGTCCAAAAGGGTGGCCTCACCAGCCCTGTGTTCCCTAG |
| hsa-miR-127-3p | TCGGATCCGTCTGAGCTTGGCT | TGTGATCACTGTCTCCAGCCTGCTGAAGCTCAGAGGGCTCTGATTCAGAAAGATCATCGGATCCGTCTGAGCTTGGCTGGTCGGAAGTCTCATCATC |
| hsa-miR-5004-3p | CTTGGATTTTCCTGGGCCTCAG | GGCACTTGCTTGGGGGTTAGTGAGGACAGGGCAAATTCACGAGATTGGGTTGTGCAGAGGCTGACACTTGGATTTTCCTGGGCCTCAGGACTTCCTTTCAGACATGG |
| hsa-miR-2681-5p | GTTTTACCACCTCCAGGAGACT | GCCCCCTTTTCACGCATTTGTGTTTTACCACCTCCAGGAGACTGCCCAAAGACTCTTCAGTATCATGGAGTTGGTAAAGCACAGATGCATGAATAATTCAACGTG |
| hsa-miR-23b-3p | ATCACATTGCCAGGGATTACCAC | CTCAGGTGCTCTGGCTGCTTGGGTTCCTGGCATGCTGATTTGTGACTTAAGATTAAAATCACATTGCCAGGGATTACCACGCAACCACGACCTTGGC |
| hsa-miR-3613-5p | TGTTGTACTTTTTTTTTTGTTC | TGGTTGGGTTTGGATTGTTGTACTTTTTTTTTTGTTCGTTGCATTTTTAGGAACAAAAAAAAAAGCCCAACCCTTCACACCACTTCA |
| hsa-miR-3614-5p | CCACTTGGATCTGAAGGCTGCCC | GGTTCTGTCTTGGGCCACTTGGATCTGAAGGCTGCCCCTTTGCTCTCTGGGGTAGCCTTCAGATCTTGGTGTTTTGAATTCTTACT |
| hsa-miR-98-3p | CTATACAACTTACTACTTTCCC | AGGATTCTGCTCATGCCAGGGTGAGGTAGTAAGTTGTATTGTTGTGGGGTAGGGATATTAGGCCCCAATTAGAAGATAACTATACAACTTACTACTTTCCCTGGTGTGTGGCATATTCA |
| hsa-miR-6795-3p | ACCCCTCGTTTCTTCCCCCAG | AGGGTTGGGGGGACAGGATGAGAGGCTGTCTTCATTCCCTCTTGACCACCCCTCGTTTCTTCCCCCAG |
| hsa-miR-6501-5p | AGTTGCCAGGGCTGCCTTTGGT | GGAGTTGCCAGGGCTGCCTTTGGTGACAGCAGCAGTAGAGTTGCCAGAGCAGCCTGCGGTAACAGTA |
| hsa-miR-4520-5p | CCTGCGTGTTTTCTGTCCAA | GTGTGCCACCTGCGTGTTTTCTGTCCAAATCAGAAAAGGATTTGGACAGAAAACACGCAGGAAGAAGGAA |
| hsa-miR-4774-3p | ATTGCCTAACATGTGCCAGAA | TATATTGTTGTCTGGTATGTAGTAGGTAATAACTGACAAACAGACAATTGCCTAACATGTGCCAGAAAACAACATA |
| hsa-miR-4323 | CAGCCCCACAGCCTCAGA | CGGGGCCCAGGCGGGCATGTGGGGTGTCTGGAGACGCCAGGCAGCCCCACAGCCTCAGACCTCGGGCAC |
| hsa-miR-4750-3p | CCTGACCCACCCCCTCCCGCAG | CGCTCGGGCGGAGGTGGTTGAGTGCCGACTGGCGCCTGACCCACCCCCTCCCGCAG |
| hsa-miR-4665-5p | CTGGGGGACGCGTGAGCGCGAGC | CTCGAGGTGCTGGGGGACGCGTGAGCGCGAGCCGCTTCCTCACGGCTCGGCCGCGGCGCGTAGCCCCCGCCACATCGGG |
| hsa-miR-302a-3p | TAAGTGCTTCCATGTTTTGGTGA | CCACCACTTAAACGTGGATGTACTTGCTTTGAAACTAAAGAAGTAAGTGCTTCCATGTTTTGGTGATGG |
| hsa-miR-148b-5p | AAGTTCTGTTATACACTCAGGC | CAAGCACGATTAGCATTTGAGGTGAAGTTCTGTTATACACTCAGGCTGTGGCTCTCTGAAAGTCAGTGCATCACAGAACTTTGTCTCGAAAGCTTTCTA |
| hsa-miR-3129-5p | GCAGTAGTGTAGAGATTGGTTT | GTACTTGGGCAGTAGTGTAGAGATTGGTTTGCCTGTTAATGAATTCAAACTAATCTCTACACTGCTGCCCAAGAGC |
| hsa-miR-1843 | TATGGAGGTCTCTGTCTGGC | GCAGCGATCCTACATAAATATATGGAGGTCTCTGTCTGGCTTAGGACAGCTGGCTAAGTCTGATCGTTCCCCTCCGTACAGCCTTTAAAACTGCCACTCTAAATACTTGG |
| hsa-miR-1343-5p | TGGGGAGCGGCCCCCGGGTGGG | GCTGGCGTCGGTGCTGGGGAGCGGCCCCCGGGTGGGCCTCTGCTCTGGCCCCTCCTGGGGCCCGCACTCTCGCTCTGGGCCCGC |
| hsa-miR-3960 | GGCGGCGGCGGAGGCGGGGG | GGCGCCCCGGCTCCCCGCGCCCCCGATCGGGGCCGCCGCTAGTAGTGGCGGCGGCGGAGGCGGGGGCAGCGGCGGCGGCGGCGGAGGCGCC |
| hsa-miR-3132 | TGGGTAGAGAAGGAGCTCAGAGGA | GGTGGGATGGGTAGAGAAGGAGCTCAGAGGACGGTGCGCCTTGTTTCCCTTGAGCCCTCCCTCTCTCATCCCACC |
| hsa-miR-31-5p | AGGCAAGATGCTGGCATAGCT | GGAGAGGAGGCAAGATGCTGGCATAGCTGTTGAACTGGGAACCTGCTATGCCAACATATTGCCATCTTTCC |
| hsa-miR-518f-3p | GAAAGCGCTTCTCTTTAGAGG | TCTCATGCTGTGACCCTCTAGAGGGAAGCACTTTCTCTTGTCTAAAAGAAAAGAAAGCGCTTCTCTTTAGAGGATTACTCTTTGAGA |
| hsa-miR-6825-3p | GCGCTGACCCGCCTTCTCCGCA | GGGCATGGGGAGGTGTGGAGTCAGCATGGGGCTAGGAGGCCCCGCGCTGACCCGCCTTCTCCGCAG |
| hsa-miR-126-5p | CATTATTACTTTTGGTACGCG | CGCTGGCGACGGGACATTATTACTTTTGGTACGCGCTGTGACACTTCAAACTCGTACCGTGAGTAATAATGCGCCGTCCACGGCA |
| hsa-miR-6770-3p | CTGGCGGCTGTGTCTTCACAG | TATCCTGAGAAGGCACAGCTTGCACGTGACCTCCTGGGCCTGGCGGCTGTGTCTTCACAG |
| hsa-miR-4420 | GTCACTGATGTCTGTAGCTGAG | CTCTTGGTATGAACATCTGTGTGTTCATGTCTCTCTGTGCACAGGGGACGAGAGTCACTGATGTCTGTAGCTGAGAC |
| hsa-miR-1258 | AGTTAGGATTAGGTCGTGGAA | CTGTGGCTTCCACGACCTAATCCTAACTCCTGCGAGTCCCTGGAGTTAGGATTAGGTCGTGGAAGCCACAGGA |
| hsa-miR-488-5p | CCCAGATAATGGCACTCTCAA | GAGAATCATCTCTCCCAGATAATGGCACTCTCAAACAAGTTTCCAAATTGTTTGAAAGGCTATTTCTTGGTCAGATGACTCTC |
| hsa-miR-3138 | TGTGGACAGTGAGGTAGAGGGAGT | CCCTCCTCGGCACTTCCCCCACCTCACTGCCCGGGTGCCCACAAGACTGTGGACAGTGAGGTAGAGGGAGTGCCGAGGAGGG |
| hsa-miR-4662b | AAAGATGGACAATTGGCTAAAT | CACAATTTCTATTTAGCCAATTGTCTATCTTTAGGCATTCAGAATAGCTAAAGATGGACAATTGGCTAAATAGACACTGTG |
| hsa-miR-6775-3p | AGGCCCTGTCCTCTGCCCCAG | GAACCTCGGGGCATGGGGGAGGGAGGCTGGACAGGAGAGGGCTCACCCAGGCCCTGTCCTCTGCCCCAG |
| hsa-miR-6864-5p | TTGAAGGGACAAGTCAGATATGCC | GAAAGTTGAAGGGACAAGTCAGATATGCCATTATGTGGGTCATGAGGTGGTGAGACTTCTCTCCCTTCAG |
| hsa-miR-6721-5p | TGGGCAGGGGCTTATTGTAGGAG | CCCTCATCTCTGGGCAGGGGCTTATTGTAGGAGTCTCTGAAGAGAGCTGTGGACTGACCTGCTTTAACCCTTCCCCAGGTTCCCATT |
| hsa-miR-6782-3p | CACCTTTGTGTCCCCATCCTGCA | TGGGGTAGGGGTGGGGGAATTCAGGGGTGTCGAACTCATGGCTGCCACCTTTGTGTCCCCATCCTGCAG |
| hsa-miR-139-3p | TGGAGACGCGGCCCTGTTGGAGT | GTGTATTCTACAGTGCACGTGTCTCCAGTGTGGCTCGGAGGCTGGAGACGCGGCCCTGTTGGAGTAAC |
| hsa-miR-491-3p | CTTATGCAAGATTCCCTTCTAC | TTGACTTAGCTGGGTAGTGGGGAACCCTTCCATGAGGAGTAGAACACTCCTTATGCAAGATTCCCTTCTACCTGGCTGGGTTGG |
| hsa-miR-4778-3p | TCTTCTTCCTTTGCAGAGTTGA | TCACATGTCCAATTCTGTAAAGGAAGAAGAGGTAAGAAGAAGTGAAGCCCTCTTCTTCCTTTGCAGAGTTGAATATGTGG |
| hsa-miR-4659a-5p | CTGCCATGTCTAAGAAGAAAAC | GAAACTGCTGAAGCTGCCATGTCTAAGAAGAAAACTTTGGAGAAAAATTTTCTTCTTAGACATGGCAACGTCAACAGTTTC |
| hsa-miR-6882-3p | TGCTGCCTCTCCTCTTGCCTGCAG | GGCTTTACAAGTCAGGAGCTGAAGCAGCTGGAATTCAAGCCCTGCTGCCTCTCCTCTTGCCTGCAG |
| hsa-miR-6754-5p | CCAGGGAGGCTGGTTTGGAGGA | GGCTGCCAGGGAGGCTGGTTTGGAGGAGTCTGGTGGCCTGTTCTCTTCACCTGCCTCTGCCTGCAG |
| hsa-miR-376c-3p | AACATAGAGGAAATTCCACGT | AAAAGGTGGATATTCCTTCTATGTTTATGTTATTTATGGTTAAACATAGAGGAAATTCCACGTTTT |
| hsa-miR-6826-3p | CTCCCCTCTCTTTCCTGTTCAG | CTTGGTCAATAGGAAAGAGGTGGGACCTCCTGGCTTTTCCTCTGCAGCATGGCTCGGACCTAGTGCAATGTTTAAGCTCCCCTCTCTTTCCTGTTCAG |
| hsa-miR-6867-3p | CTCTCCCTCTTTACCCACTAG | CCCGGTGTGTGTGTAGAGGAAGAAGGGAAGCTGGGAACCTGACTGCCTCTCCCTCTTTACCCACTAG |
| hsa-miR-30b-5p | TGTAAACATCCTACACTCAGCT | ACCAAGTTTCAGTTCATGTAAACATCCTACACTCAGCTGTAATACATGGATTGGCTGGGAGGTGGATGTTTACTTCAGCTGACTTGGA |
| hsa-miR-5687 | TTAGAACGTTTTAGGGTCAAAT | CCTCACTTATCTGACTCTGAAATCTTCTAAATGGTACCCACTTTATTTAGAACGTTTTAGGGTCAAATAAGTACAGG |
| hsa-miR-9-3p | ATAAAGCTAGATAACCGAAAGT | CGGGGTTGGTTGTTATCTTTGGTTATCTAGCTGTATGAGTGGTGTGGAGTCTTCATAAAGCTAGATAACCGAAAGTAAAAATAACCCCA |
| hsa-miR-6858-3p | CAGCCAGCCCCTGCTCACCCCT | GTGAGGAGGGGCTGGCAGGGACCCCTCCAAGTTGGGGACGGCAGCCAGCCCCTGCTCACCCCTCGCC |
| hsa-miR-2681-3p | TATCATGGAGTTGGTAAAGCAC | GCCCCCTTTTCACGCATTTGTGTTTTACCACCTCCAGGAGACTGCCCAAAGACTCTTCAGTATCATGGAGTTGGTAAAGCACAGATGCATGAATAATTCAACGTG |
| hsa-miR-3684 | TTAGACCTAGTACACGTCCTT | AATCTAAAGGACCTGTACTAGGTTTAACATGTTGAGCATTACTCATGTTAGACCTAGTACACGTCCTTTAGATT |
| hsa-miR-208a-5p | GAGCTTTTGGCCCGGGTTATAC | TGACGGGCGAGCTTTTGGCCCGGGTTATACCTGATGCTCACGTATAAGACGAGCAAAAAGCTTGTTGGTCA |
| hsa-miR-12133 | CTTGGCACCATTAAAAAGTACA | GAAGTGTACTTTTTAATGGTGCCAAACAGCAGTTGATCTATAATAACTGCTGCTTGGCACCATTAAAAAGTACATATC |
| hsa-miR-3191-3p | TGGGGACGTAGCTGGCCAGACAG | GGGGTCACCTCTCTGGCCGTCTACCTTCCACACTGACAAGGGCCGTGGGGACGTAGCTGGCCAGACAGGTGACCCC |
| hsa-miR-4524b-5p | ATAGCAGCATAAGCCTGTCTC | TAGCTGGGTGGATGTGTTCTTTTGAAGGATAGCAGCATAAGCCTGTCTCAAAATAATTCTGCAGTGAGACAGGTTCATGCTGCTATCGTTCCAAAGAGGAAGGGTAATCACTGTC |
| hsa-miR-6797-3p | TGCATGACCCTTCCCTCCCCAC | CAGCCAGGAGGGAAGGGGCTGAGAACAGGACCTGTGCTCACTGGGGCCTGCATGACCCTTCCCTCCCCACAG |
| hsa-miR-1914-5p | CCCTGTGCCCGGCCCACTTCTG | CGTGTGAGCCCGCCCTGTGCCCGGCCCACTTCTGCTTCCTCTTAGCGCAGGAGGGGTCCCGCACTGGGAGGGGCCCTCAC |
| hsa-miR-330-5p | TCTCTGGGCCTGTGTCTTAGGC | CTTTGGCGATCACTGCCTCTCTGGGCCTGTGTCTTAGGCTCTGCAAGATCAACCGAGCAAAGCACACGGCCTGCAGAGAGGCAGCGCTCTGCCC |
| hsa-miR-139-5p | TCTACAGTGCACGTGTCTCCAGT | GTGTATTCTACAGTGCACGTGTCTCCAGTGTGGCTCGGAGGCTGGAGACGCGGCCCTGTTGGAGTAAC |
| hsa-miR-10401-3p | ACCTCGCCGTCCCGCCCGCCG | CGTGTGGGAAGGCGTGGGGTGCGGACCCCGGCCCGACCTCGCCGTCCCGCCCGCCG |
| hsa-miR-6761-5p | TCTGAGAGAGCTCGATGGCAG | TCTGCTCTGAGAGAGCTCGATGGCAGGTGCCTCCGTGTTGCCGAACCCTCCTACGCTGCTCTCTCACTCCAG |
| hsa-miR-941 | CACCCGGCTGTGTGCACATGTGC | TGTGGACATGTGCCCAGGGCCCGGGACAGCGCCACGGAAGAGGACGCACCCGGCTGTGTGCACATGTGCCCA |
| hsa-miR-4795-5p | AGAAGTGGCTAATAATATTGA | TGATATGGAAGAAATCCAGAAGTGGCTAATAATATTGACACTATAACAATAATGTCAATATTATTAGCCACTTCTGGATTTATGAATCA |
| hsa-miR-181b-3p | CTCACTGAACAATGAATGCAA | CCTGTGCAGAGATTATTTTTTAAAAGGTCACAATCAACATTCATTGCTGTCGGTGGGTTGAACTGTGTGGACAAGCTCACTGAACAATGAATGCAACTGTGGCCCCGCTT |
| hsa-miR-301a-5p | GCTCTGACTTTATTGCACTACT | ACTGCTAACGAATGCTCTGACTTTATTGCACTACTGTACTTTACAGCTAGCAGTGCAATAGTATTGTCAAAGCATCTGAAAGCAGG |
| hsa-miR-548ap-3p | AAAAACCACAATTACTTTT | ACCAATTCCTAGGTTGGTGCAAAAGTAATTGCGGTCTTTGTCATTAAAACCAATAACAAAAACCACAATTACTTTTTACTGACCTAAAGATTAATT |
| hsa-miR-6855-3p | AGACTGACCTTCAACCCCACAG | GCTGCTTGGGGTTTGGGGTGCAGACATTGCCAGAGGATGGGCAGCAGACTGACCTTCAACCCCACAG |
| hsa-miR-340-3p | TCCGTCTCAGTTACTTTATAGC | TTGTACCTGGTGTGATTATAAAGCAATGAGACTGATTGTCATATGTCGTTTGTGGGATCCGTCTCAGTTACTTTATAGCCATACCTGGTATCTTA |
| hsa-miR-4742-5p | TCAGGCAAAGGGATATTTACAGA | TCAGGCAAAGGGATATTTACAGATACTTTTTAAAATTTGTTTGAGTTGAGGCAGATTAAATATCTGTATTCTCCTTTGCCTGCAG |
| hsa-miR-4446-5p | ATTTCCCTGCCATTCCCTTGGC | CTGGTCCATTTCCCTGCCATTCCCTTGGCTTCAATTTACTCCCAGGGCTGGCAGTGACATGGGTCAA |
| hsa-miR-126-3p | TCGTACCGTGAGTAATAATGCG | CGCTGGCGACGGGACATTATTACTTTTGGTACGCGCTGTGACACTTCAAACTCGTACCGTGAGTAATAATGCGCCGTCCACGGCA |
| hsa-miR-451a | AAACCGTTACCATTACTGAGTT | CTTGGGAATGGCAAGGAAACCGTTACCATTACTGAGTTTAGTAATGGTAATGGTTCTCTTGCTATACCCAGA |
| hsa-miR-548ag | AAAGGTAATTGTGGTTTCTGC | GTGCAAAGGTAATTGTGGTTTCTGCTTTTAAAGGTAATGGCAAATATTACATTTACTTTTGCACCA |
| hsa-miR-3194-3p | AGCTCTGCTGCTCACTGGCAGT | AGGTGGCAGGGCCAGCCACCAGGAGGGCTGCGTGCCACCCGGGCAGCTCTGCTGCTCACTGGCAGTGTCACCT |
| hsa-miR-7151-5p | GATCCATCTCTGCCTGTATTGGC | GATCCATCTCTGCCTGTATTGGCTTGGATTCTGCAAAGCCTACAGGCTGGAATGGGCTCA |
| hsa-miR-4709-5p | ACAACAGTGACTTGCTCTCCAA | CTGCTTCAACAACAGTGACTTGCTCTCCAATGGTATCCAGTGATTCGTTGAAGAGGAGGTGCTCTGTAGCAG |
| hsa-miR-2277-5p | AGCGCGGGCTGAGCGCTGCCAGTC | GTGCTTCCTGCGGGCTGAGCGCGGGCTGAGCGCTGCCAGTCAGCGCTCACATTAAGGCTGACAGCGCCCTGCCTGGCTCGGCCGGCGAAGCTC |
| hsa-miR-4498 | TGGGCTGGCAGGGCAAGTGCTG | AGGGCTGGGCTGGCAGGGCAAGTGCTGCAGATCTTTGTCTAAGCAGCCCCTGCCTTGGATCTCCCA |
| hsa-miR-103a-3p | AGCAGCATTGTACAGGGCTATGA | TTGTGCTTTCAGCTTCTTTACAGTGCTGCCTTGTAGCATTCAGGTCAAGCAGCATTGTACAGGGCTATGAAAGAACCA |
| hsa-let-7i-3p | CTGCGCAAGCTACTGCCTTGCT | CTGGCTGAGGTAGTAGTTTGTGCTGTTGGTCGGGTTGTGACATTGCCCGCTGTGGAGATAACTGCGCAAGCTACTGCCTTGCTA |
| hsa-miR-5702 | TGAGTCAGCAACATATCCCATG | GCCTCAACTCCTGGGATATGTTGCTGATCCAACCTGAAATCCTTCTGTAGGTTGAGTCAGCAACATATCCCATGACTTTTGGGT |
| hsa-miR-199b-5p | CCCAGTGTTTAGACTATCTGTTC | CCAGAGGACACCTCCACTCCGTCTACCCAGTGTTTAGACTATCTGTTCAGGACTCCCAAATTGTACAGTAGTCTGCACATTGGTTAGGCTGGGCTGGGTTAGACCCTCGG |
| hsa-miR-383-3p | ACAGCACTGCCTGGTCAGA | CTCCTCAGATCAGAAGGTGATTGTGGCTTTGGGTGGATATTAATCAGCCACAGCACTGCCTGGTCAGAAAGAG |
| hsa-miR-4696 | TGCAAGACGGATACTGTCATCT | CAAAGCCACTGCAAGACGGATACTGTCATCTATTCCAGAAGATGACAATGTCCATTTTGCAGTGGCTTTG |
| hsa-miR-518e-3p | AAAGCGCTTCCCTTCAGAGTG | TCTCAGGCTGTGACCCTCTAGAGGGAAGCGCTTTCTGTTGGCTAAAAGAAAAGAAAGCGCTTCCCTTCAGAGTGTTAACGCTTTGAGA |
| hsa-miR-7114-5p | TCTGTGGAGTGGGGTGCCTGT | TCCGCTCTGTGGAGTGGGGTGCCTGTCCCCTGCCACTGGGTGACCCACCCCTCTCCACCAG |
| hsa-miR-6821-5p | GTGCGTGGTGGCTCGAGGCGGGG | GTGCGTGGTGGCTCGAGGCGGGGGTGGGGGCCTCGCCCTGCTTGGGCCCTCCCTGACCTCTCCGCTCCGCACAG |
| hsa-miR-6879-3p | TGTCACCCGCTCCTTGCCCAG | CAGAGCAGGGCAGGGAAGGTGGGAGAGGGGCCCAGCTGACCCTCCTGTCACCCGCTCCTTGCCCAG |
| hsa-miR-5580-5p | TGCTGGCTCATTTCATATGTGT | TGCTGGCTCATTTCATATGTGTGCTGAGAAAATTCACACATATGAAGTGAGCCAGCAC |
| hsa-miR-518a-5p | CTGCAAAGGGAAGCCCTTTC | TCTCAAGCTGTGACTGCAAAGGGAAGCCCTTTCTGTTGTCTAAAAGAAAAGAAAGTGCTTCCCTTTGGTGAATTACGGTTTGAGA |
| hsa-miR-450a-1-3p | ATTGGGAACATTTTGCATGTAT | AAACGATACTAAACTGTTTTTGCGATGTGTTCCTAATATGCACTATAAATATATTGGGAACATTTTGCATGTATAGTTTTGTATCAATATA |
| hsa-miR-4781-5p | TAGCGGGGATTCCAATATTGG | AGGTGCACGCTCTAGCGGGGATTCCAATATTGGGCCAATTCCCCCAATGTTGGAATCCTCGCTAGAGCGTGCACTT |
| hsa-miR-585-3p | TGGGCGTATCTGTATGCTA | TGGGGTGTCTGTGCTATGGCAGCCCTAGCACACAGATACGCCCAGAGAAAGCCTGAACGTTGGGCGTATCTGTATGCTAGGGCTGCTGTAACAA |
| hsa-miR-2276-5p | GCCCTCTGTCACCTTGCAGACG | GTGTTCTTCCAGTCCGCCCTCTGTCACCTTGCAGACGGCTTTCTCTCCGAATGTCTGCAAGTGTCAGAGGCGAGGAGTGGCAGCTGCAT |
| hsa-miR-329-3p | AACACACCTGGTTAACCTCTTT | GGTACCTGAAGAGAGGTTTTCTGGGTTTCTGTTTCTTTAATGAGGACGAAACACACCTGGTTAACCTCTTTTCCAGTATC |
| hsa-miR-1915-5p | ACCTTGCCTTGCTGCCCGGGCC | TGAGAGGCCGCACCTTGCCTTGCTGCCCGGGCCGTGCACCCGTGGGCCCCAGGGCGACGCGGCGGGGGCGGCCCTAGCGA |
| hsa-miR-224-5p | TCAAGTCACTAGTGGTTCCGTTTAG | GGGCTTTCAAGTCACTAGTGGTTCCGTTTAGTAGATGATTGTGCATTGTTTCAAAATGGTGCCCTAGTGACTACAAAGCCC |
| hsa-miR-6511b-5p | CTGCAGGCAGAAGTGGGGCTGACA | GGGACGGGGCCTGCAGGCAGAAGTGGGGCTGACAGGGCAGAGGGTTGCGCCCCCTCACCACCCCTTCTGCCTGCAGCGGTGGGCT |
| hsa-miR-548ax | AGAAGTAATTGCGGTTTTGCCA | GATTGGTGCAGAAGTAATTGCGGTTTTGCCATGGAAAGTAATGGCAAAAACCGTAATTACTTTTGTACCAACC |
| hsa-miR-144-3p | TACAGTATAGATGATGTACT | TGGGGCCCTGGCTGGGATATCATCATATACTGTAAGTTTGCGATGAGACACTACAGTATAGATGATGTACTAGTCCGGGCACCCCC |
| hsa-miR-4700-5p | TCTGGGGATGAGGACAGTGTGT | TCAGTGAGGTCTGGGGATGAGGACAGTGTGTCCTGAAATTCACAGGACTGACTCCTCACCCCAGTGCACGAGGA |
| hsa-miR-4713-5p | TTCTCCCACTACCAGGCTCCCA | GTCCCCATTTTTCTCCCACTACCAGGCTCCCATAAGGGTCGAATGGGATCCAGACAGTGGGAGAAAAATGGGGAC |
| hsa-miR-520h | ACAAAGTGCTTCCCTTTAGAGT | TCCCATGCTGTGACCCTCTAGAGGAAGCACTTTCTGTTTGTTGTCTGAGAAAAAACAAAGTGCTTCCCTTTAGAGTTACTGTTTGGGA |
| hsa-miR-3616-3p | CGAGGGCATTTCATGATGCAGGC | TGTCACTCCGCCAGCATCATGAAGTGCACTCATGATATGTTTGCCCCATCAGCGTGTCACGAGGGCATTTCATGATGCAGGCGGGGTTGGCA |
| hsa-miR-499a-5p | TTAAGACTTGCAGTGATGTTT | GCCCTGTCCCCTGTGCCTTGGGCGGGCGGCTGTTAAGACTTGCAGTGATGTTTAACTCCTCTCCACGTGAACATCACAGCAAGTCTGTGCTGCTTCCCGTCCCTACGCTGCCTGGGCAGGGT |
| hsa-miR-520e-3p | AAAGTGCTTCCTTTTTGAGGG | TCTCCTGCTGTGACCCTCAAGATGGAAGCAGTTTCTGTTGTCTGAAAGGAAAGAAAGTGCTTCCTTTTTGAGGGTTACTGTTTGAGA |
| hsa-miR-374a-3p | CTTATCAGATTGTATTGTAATT | TACATCGGCCATTATAATACAACCTGATAAGTGTTATAGCACTTATCAGATTGTATTGTAATTGTCTGTGTA |
| hsa-miR-141-3p | TAACACTGTCTGGTAAAGATGG | CGGCCGGCCCTGGGTCCATCTTCCAGTACAGTGTTGGATGGTCTAATTGTGAAGCTCCTAACACTGTCTGGTAAAGATGGCTCCCGGGTGGGTTC |
| hsa-miR-6856-5p | AAGAGAGGAGCAGTGGTGCTGTGG | TGGAAAAGAGAGGAGCAGTGGTGCTGTGGCAGTGGCAGAGGTCGCTACAGCCCTGTGATCTTTCCAG |
| hsa-miR-186-5p | CAAAGAATTCTCCTTTTGGGCT | TGCTTGTAACTTTCCAAAGAATTCTCCTTTTGGGCTTTCTGGTTTTATTTTAAGCCCAAAGGTGAATTTTTTGGGAAGTTTGAGCT |
| hsa-miR-339-5p | TCCCTGTCCTCCAGGAGCTCACG | CGGGGCGGCCGCTCTCCCTGTCCTCCAGGAGCTCACGTGTGCCTGCCTGTGAGCGCCTCGACGACAGAGCCGGCGCCTGCCCCAGTGTCTGCGC |
| hsa-miR-486-5p | TCCTGTACTGAGCTGCCCCGAG | GCATCCTGTACTGAGCTGCCCCGAGGCCCTTCATGCTGCCCAGCTCGGGGCAGCTCAGTACAGGATAC |
| hsa-miR-548ay-5p | AAAAGTAATTGTGGTTTTTGC | AGAAGATGCTTACTACTAGGTTGGTGCAAAAGTAATTGTGGTTTTTGCATTTAAAGTAATGGCCAAAACCGCGATTACTCTTGCACGAACCTAACGGTAACACTTCT |
| hsa-miR-548j-5p | AAAAGTAATTGCGGTCTTTGGT | GGGCAGCCAGTGAATAGTTAGCTGGTGCAAAAGTAATTGCGGTCTTTGGTATTACTTTCAGTGGCAAAAACTGCATTACTTTTGCACCAGCCTACTAGAACGCTGAGTTCAG |
| hsa-miR-1273h-5p | CTGGGAGGTCAAGGCTGCAGT | TACTTGGGTGACTAAGGCAGGATTGCTTGAGCCTGGGAGGTCAAGGCTGCAGTGTCGTGGTCACAGCTTGCTGCAGACTCGACCTCCCAGGCTTAAGCAATCCTCCTGCTCGAGTG |
| hsa-miR-218-5p | TTGTGCTTGATCTAACCATGT | GTGATAATGTAGCGAGATTTTCTGTTGTGCTTGATCTAACCATGTGGTTGCGAGGTATGAGTAAAACATGGTTCCGTCAAGCACCATGGAACGTCACGCAGCTTTCTACA |
| hsa-miR-663b | GGTGGCCCGGCCGTGCCTGAGG | GGTGCCGAGGGCCGTCCGGCATCCTAGGCGGGTCGCTGCGGTACCTCCCTCCTGTCTGTGGCGGTGGGATCCCGTGGCCGTGTTTTCCTGGTGGCCCGGCCGTGCCTGAGGTTTC |
| hsa-miR-135a-5p | TATGGCTTTTTATTCCTATGTGA | AGGCCTCGCTGTTCTCTATGGCTTTTTATTCCTATGTGATTCTACTGCTCACTCATATAGGGATTGGAGCCGTGGCGCACGGCGGGGACA |
| hsa-miR-3922-5p | TCAAGGCCAGAGGTCCCACAGCA | GGAAGAGTCAAGTCAAGGCCAGAGGTCCCACAGCAGGGCTGGAAAGCACACCTGTGGGACTTCTGGCCTTGACTTGACTCTTTC |
| hsa-miR-3186-5p | CAGGCGTCTGTCTACGTGGCTT | AGCCTGCGGTTCCAACAGGCGTCTGTCTACGTGGCTTCAACCAAGTTCAAAGTCACGCGGAGAGATGGCTTTGGAACCAGGGGCT |
| hsa-miR-6785-5p | TGGGAGGGCGTGGATGATGGTG | CTCCCTGGGAGGGCGTGGATGATGGTGGGAGAGGAGCCCCACTGTGGAAGTCTGACCCCCACATCGCCCCACCTTCCCCAG |
| hsa-miR-1587 | TTGGGCTGGGCTGGGTTGGG | TTTGGGCTGGGCTGGGTTGGGCAGTTCTTCTGCTGGACTCACCTGTGACCAGC |
| hsa-miR-93-5p | CAAAGTGCTGTTCGTGCAGGTAG | CTGGGGGCTCCAAAGTGCTGTTCGTGCAGGTAGTGTGATTACCCAACCTACTGCTGAGCTAGCACTTCCCGAGCCCCCGG |
| hsa-miR-376c-5p | GGTGGATATTCCTTCTATGTT | AAAAGGTGGATATTCCTTCTATGTTTATGTTATTTATGGTTAAACATAGAGGAAATTCCACGTTTT |
| hsa-miR-4452 | TTGAATTCTTGGCCTTAAGTGAT | TGGATCACTTGAGGCCAAGAGTGCAAGGCTGTAGTGTGCACAGCCTTGAATTCTTGGCCTTAAGTGATCCC |
| hsa-miR-548k | AAAAGTACTTGCGGATTTTGCT | CTTTTCTCAAGTATTGCTGTTAGGTTGGTGCAAAAGTACTTGCGGATTTTGCTTTACTTTTAATGGCAAAAACCGCAATTATTTTTGCTTCAACCTAATATGATGCAAAATTGGCT |
| hsa-miR-6736-5p | CTGGGTGAGGGCATCTGTGGT | CTGAGCTGGGTGAGGGCATCTGTGGTTTGCTGGCTGCCTCAGCTCCTCTCTACCCACAG |
| hsa-miR-10395-5p | GTGATGGAGAGCAATACC | GTGATGGAGAGCAATACCCGGGGATGACTGTGACCACATTGGGATGTATTCGTACTGTCTGATG |
| hsa-miR-4482-5p | AACCCAGTGGGCTATGGAAATG | AGTGAGCAACCCAGTGGGCTATGGAAATGTGTGGAAGATGGCATTTCTATTTCTCAGTGGGGCTCTTACC |
| hsa-miR-6866-3p | GATCCCTTTATCTGTCCTCTAG | CCATTTTAGAGGCTGGAATAGAGATTCTTGAGGCTTGGAAGAGTAAGGATCCCTTTATCTGTCCTCTAG |
| hsa-miR-518f-5p | CTCTAGAGGGAAGCACTTTCTC | TCTCATGCTGTGACCCTCTAGAGGGAAGCACTTTCTCTTGTCTAAAAGAAAAGAAAGCGCTTCTCTTTAGAGGATTACTCTTTGAGA |
| hsa-miR-6511b-3p | CCTCACCACCCCTTCTGCCTGCA | GGGACGGGGCCTGCAGGCAGAAGTGGGGCTGACAGGGCAGAGGGTTGCGCCCCCTCACCACCCCTTCTGCCTGCAGCGGTGGGCT |
| hsa-miR-196a-1-3p | CAACAACATTAAACCACCCGA | GTGAATTAGGTAGTTTCATGTTGTTGGGCCTGGGTTTCTGAACACAACAACATTAAACCACCCGATTCAC |
| hsa-miR-4803 | TAACATAATAGTGTGGATTGA | AGTGGGATTTAACATAATAGTGTGGATTGAATCACACACACATTTCAACCCACACTATGATGTTAAATCCCATT |
| hsa-miR-887-5p | CTTGGGAGCCCTGTTAGACTC | GTGCAGATCCTTGGGAGCCCTGTTAGACTCTGGATTTTACACTTGGAGTGAACGGGCGCCATCCCGAGGCTTTGCACAG |
| hsa-miR-6509-3p | TTCCACTGCCACTACCTAATTT | TTTTTGTGTGTGAAATTAGGTAGTGGCAGTGGAACACTATATTAATCAGGTTTCCACTGCCACTACCTAATTTCTCAGATGGAAA |
| hsa-miR-4772-3p | CCTGCAACTTTGCCTGATCAGA | GTGATTGCCTCTGATCAGGCAAAATTGCAGACTGTCTTCCCAAATAGCCTGCAACTTTGCCTGATCAGAGGCAGTCAC |
| hsa-miR-3193 | TCCTGCGTAGGATCTGAGGAGT | TCCTGCGTAGGATCTGAGGAGTGGACGAGTCTCATTACCCAGCTCCTGAGCAGGA |
| hsa-miR-4783-3p | CCCCGGTGTTGGGGCGCGTCTGC | GGGAAAGCGGAGGGCGCGCCCAGCTCCCGGGCTGATTGCGCTAACAGTGGCCCCGGTGTTGGGGCGCGTCTGCCGCTGCCCC |
| hsa-miR-10396a-5p | GGCGGGGCTCGGAGCCGGG | GGCGGGGCTCGGAGCCGGGCTTCGGCCGGGCCCCGGGCCCTCGACCGGG |
| hsa-miR-6761-3p | TCCTACGCTGCTCTCTCACTCC | TCTGCTCTGAGAGAGCTCGATGGCAGGTGCCTCCGTGTTGCCGAACCCTCCTACGCTGCTCTCTCACTCCAG |
| hsa-miR-6734-3p | CCCTTCCCTCACTCTTCTCTCAG | AGAACTTGAGGGGAGAATGAGGTGGAGAAGCCCAGGTTCTGAATCCCCTTCCCTCACTCTTCTCTCAG |
| hsa-miR-892a | CACTGTGTCCTTTCTGCGTAG | GCAGTGCCTTACTCAGAAAGGTGCCAGTCACTTACACTACATGTCACTGTGTCCTTTCTGCGTAGAGTAAGGCTC |
| hsa-miR-379-3p | TATGTAACATGGTCCACTAACT | AGAGATGGTAGACTATGGAACGTAGGCGTTATGATTTCTGACCTATGTAACATGGTCCACTAACTCT |
| hsa-miR-1304-3p | TCTCACTGTAGCCTCGAACCCC | AAACACTTGAGCCCAGCGGTTTGAGGCTACAGTGAGATGTGATCCTGCCACATCTCACTGTAGCCTCGAACCCCTGGGCTCAAGTGATTCA |
| hsa-miR-24-1-5p | TGCCTACTGAGCTGATATCAGT | CTCCGGTGCCTACTGAGCTGATATCAGTTCTCATTTTACACACTGGCTCAGTTCAGCAGGAACAGGAG |
| hsa-miR-3689a-5p | TGTGATATCATGGTTCCTGGGA | GGGAGGTGTGATATCATGGTTCCTGGGAGGTATGATATCGTGGTTCCTGGGAGGTGTGATCCCGTGCTCCCT |
| hsa-miR-5010-5p | AGGGGGATGGCAGAGCAAAATT | GATCCAGGGAACCCTAGAGCAGGGGGATGGCAGAGCAAAATTCATGGCCTACAGCTGCCTCTTGCCAAACTGCACTGGATTTTGTGTCTCCCATTCCCCAGAGCTGTCTGAGGTGCTTTG |
| hsa-miR-4424 | AGAGTTAACTCAAAATGGACTA | CTTACATCACACACAGAGTTAACTCAAAATGGACTAATTTTTCCACTAGTTAGTCCATTTCAAGTTAACTCTGTGTGTGATGTAGT |
| hsa-miR-7703 | TTGCACTCTGGCCTTCTCCCAGG | TTAGGGGAGGTGGATGAGTGGGGGCAGGCAGGGGATTGGCTAAGAACCTGACTCTTGCACTCTGGCCTTCTCCCAGG |
| hsa-miR-652-5p | CAACCCTAGGAGAGGGTGCCATTCA | ACGAATGGCTATGCACTGCACAACCCTAGGAGAGGGTGCCATTCACATAGACTATAATTGAATGGCGCCACTAGGGTTGTGCAGTGCACAACCTACAC |
| hsa-miR-3691-3p | ACCAAGTCTGCGTCATCCTCTC | TTGAGGCACTGGGTAGTGGATGATGGAGACTCGGTACCCACTGCTGAGGGTGGGGACCAAGTCTGCGTCATCCTCTCCTCAGTGCCTCAA |
| hsa-miR-3183 | GCCTCTCTCGGAGTCGCTCGGA | CTCTGCCCTGCCTCTCTCGGAGTCGCTCGGAGCAGTCACGTTGACGGAATCCTCCGGCGCCTCCTCGAGGGAGGAGAGGCAGGG |
| hsa-miR-6812-5p | ATGGGGTGAGATGGGGAGGAGCAGC | TGAGGATGGGGTGAGATGGGGAGGAGCAGCCAGTCCTGTCTCACCGCTCTTCCCCTGACCCCAG |
| hsa-miR-556-3p | ATATTACCATTAGCTCATCTTT | GATAGTAATAAGAAAGATGAGCTCATTGTAATATGAGCTTCATTTATACATTTCATATTACCATTAGCTCATCTTTTTTATTACTACCTTCAACA |
| hsa-miR-548c-5p | AAAAGTAATTGCGGTTTTTGCC | TGGTGCAAAAGTAATTGCGGTTTTTGCCATTAAAAGTAATGCGGCCAAAACTGCAGTTACTTTTGCACCC |
| hsa-miR-564 | AGGCACGGTGTCAGCAGGC | CGGGCAGCGGGTGCCAGGCACGGTGTCAGCAGGCAACATGGCCGAGAGGCCGGGGCCTCCGGGCGGCGCCGTGTCCGCGACCGCGTACCCTGAC |
| hsa-miR-548aq-3p | CAAAAACTGCAATTACTTTTGC | GAAAGTAATTGCTGTTTTTGCCATTACTTTCAGTGGCAAAAACTGCAATTACTTTTGC |
| hsa-miR-4738-3p | TGAAACTGGAGCGCCTGGAGGA | GGTCGCATTTCTCCTTCTTACCAGCGCGTTTTCAGTTTCATAGGGAAGCCTTTCCATGAAACTGGAGCGCCTGGAGGAGAAGGGGCC |
| hsa-miR-876-3p | TGGTGGTTTACAAAGTAATTCA | TGAAGTGCTGTGGATTTCTTTGTGAATCACCATATCTAAGCTAATGTGGTGGTGGTTTACAAAGTAATTCATAGTGCTTCA |
| hsa-miR-6792-3p | CTCCTCCACAGCCCCTGCTCAT | GTAAGCAGGGGCTCTGGGTGATGTGAGGAGCAACAGGCACCCTCCTCCACAGCCCCTGCTCATTCCT |
| hsa-miR-3922-3p | TCTGGCCTTGACTTGACTCTTT | GGAAGAGTCAAGTCAAGGCCAGAGGTCCCACAGCAGGGCTGGAAAGCACACCTGTGGGACTTCTGGCCTTGACTTGACTCTTTC |
| hsa-miR-423-5p | TGAGGGGCAGAGAGCGAGACTTT | ATAAAGGAAGTTAGGCTGAGGGGCAGAGAGCGAGACTTTTCTATTTTCCAAAAGCTCGGTCTGAGGCCCCTCAGTCTTGCTTCCTAACCCGCGC |
| hsa-miR-9901 | CGGTCGCCGCGGTTCGCCGCC | GGAGAGGGTGGGGGAGCCCGTCCCGGTCGCCGCGGTTCGCCGCCGCCCCTGGTGGCGGTCCGGCGACCGGCCCACCATCGCTCCAGCGCCCCTCCT |
| hsa-miR-200a-3p | TAACACTGTCTGGTAACGATGT | CCGGGCCCCTGTGAGCATCTTACCGGACAGTGCTGGATTTCCCAGCTTGACTCTAACACTGTCTGGTAACGATGTTCAAAGGTGACCCGC |
| hsa-miR-6870-5p | TGGGGGAGATGGGGGTTGA | CAAGGTGGGGGAGATGGGGGTTGAACTTCATTTCTCATGCTCATCCCCATCTCCTTTCAG |
| hsa-miR-509-5p | TACTGCAGACAGTGGCAATCA | CATGCTGTGTGTGGTACCCTACTGCAGACAGTGGCAATCATGTATAATTAAAAATGATTGGTACGTCTGTGGGTAGAGTACTGCATGACACATG |
| hsa-miR-944 | AAATTATTGTACATCGGATGAG | GTTCCAGACACATCTCATCTGATATACAATATTTTCTTAAATTGTATAAAGAGAAATTATTGTACATCGGATGAGCTGTGTCTGGGAT |
| hsa-miR-3184-3p | AAAGTCTCGCTCTCTGCCCCTCA | AAGCAAGACTGAGGGGCCTCAGACCGAGCTTTTGGAAAATAGAAAAGTCTCGCTCTCTGCCCCTCAGCCTAACTT |
| hsa-miR-548e-3p | AAAAACTGAGACTACTTTTGCA | TTATTAGGTTGGTACAAAAGCAATCGCGGTTTTTGCTATTACTTTTAAAGGCAAAAACTGAGACTACTTTTGCACCAACCTGATAGAA |
| hsa-miR-29b-1-5p | GCTGGTTTCATATGGTGGTTTAGA | CTTCAGGAAGCTGGTTTCATATGGTGGTTTAGATTTAAATAGTGATTGTCTAGCACCATTTGAAATCAGTGTTCTTGGGGG |
| hsa-miR-1343-3p | CTCCTGGGGCCCGCACTCTCGC | GCTGGCGTCGGTGCTGGGGAGCGGCCCCCGGGTGGGCCTCTGCTCTGGCCCCTCCTGGGGCCCGCACTCTCGCTCTGGGCCCGC |
| hsa-miR-515-3p | GAGTGCCTTCTTTTGGAGCGTT | TCTCATGCAGTCATTCTCCAAAAGAAAGCACTTTCTGTTGTCTGAAAGCAGAGTGCCTTCTTTTGGAGCGTTACTGTTTGAGA |
| hsa-miR-3166 | CGCAGACAATGCCTACTGGCCTA | AAATTTTTTTGAGGCCAGTAGGCATTGTCTGCGTTAGGATTTCTGTATCATCCTCCTAACGCAGACAATGCCTACTGGCCTAAGAAAAATTT |
| hsa-miR-137-5p | ACGGGTATTCTTGGGTGGATAAT | GGTCCTCTGACTCTCTTCGGTGACGGGTATTCTTGGGTGGATAATACGGATTACGTTGTTATTGCTTAAGAATACGCGTAGTCGAGGAGAGTACCAGCGGCA |
| hsa-miR-10395-3p | ATGTATTCGTACTGTCTGATG | GTGATGGAGAGCAATACCCGGGGATGACTGTGACCACATTGGGATGTATTCGTACTGTCTGATG |
| hsa-miR-203b-5p | TAGTGGTCCTAAACATTTCACA | GCGCCCGCCGGGTCTAGTGGTCCTAAACATTTCACAATTGCGCTACAGAACTGTTGAACTGTTAAGAACCACTGGACCCAGCGCGC |
| hsa-miR-410-3p | AATATAACACAGATGGCCTGT | GGTACCTGAGAAGAGGTTGTCTGTGATGAGTTCGCTTTTATTAATGACGAATATAACACAGATGGCCTGTTTTCAGTACC |
| hsa-miR-3164 | TGTGACTTTAAGGGAAATGGCG | CTTGGAAACTGTGACTTTAAGGGAAATGGCGCACAGCAGACCCTGCAATCATGCCGTTTTGCTTGAAGTCGCAGTTTCCCAGG |
| hsa-miR-365a-3p | TAATGCCCCTAAAAATCCTTAT | AGAGTGTTCAAGGACAGCAAGAAAAATGAGGGACTTTCAGGGGCAGCTGTGTTTTCTGACTCAGTCATAATGCCCCTAAAAATCCTTATTGTTCTTGCAGTGTGCATCGGG |
| hsa-miR-216a-5p | TAATCTCAGCTGGCAACTGTGA | GATGGCTGTGAGTTGGCTTAATCTCAGCTGGCAACTGTGAGATGTTCATACAATCCCTCACAGTGGTCTCTGGGATTATGCTAAACAGAGCAATTTCCTAGCCCTCACGA |
| hsa-miR-514a-5p | TACTCTGGAGAGTGACAATCATG | AACATGTTGTCTGTGGTACCCTACTCTGGAGAGTGACAATCATGTATAATTAAATTTGATTGACACTTCTGTGAGTAGAGTAACGCATGACACGTACG |
| hsa-miR-3190-5p | TCTGGCCAGCTACGTCCCCA | CTGGGGTCACCTGTCTGGCCAGCTACGTCCCCACGGCCCTTGTCAGTGTGGAAGGTAGACGGCCAGAGAGGTGACCCCGG |
| hsa-miR-6795-5p | TGGGGGGACAGGATGAGAGGCTGT | AGGGTTGGGGGGACAGGATGAGAGGCTGTCTTCATTCCCTCTTGACCACCCCTCGTTTCTTCCCCCAG |
| hsa-miR-4467 | TGGCGGCGGTAGTTATGGGCTT | TGGTGGCGGCGGTAGTTATGGGCTTCTCTTTCTCACCAGCAGCCCCTGGGCCGCCGCCTCCCT |
| hsa-miR-6734-5p | TTGAGGGGAGAATGAGGTGGAGA | AGAACTTGAGGGGAGAATGAGGTGGAGAAGCCCAGGTTCTGAATCCCCTTCCCTCACTCTTCTCTCAG |
| hsa-miR-155-5p | TTAATGCTAATCGTGATAGGGGTT | CTGTTAATGCTAATCGTGATAGGGGTTTTTGCCTCCAACTGACTCCTACATATTAGCATTAACAG |
| hsa-miR-548x-3p | TAAAAACTGCAATTACTTTC | AGGTTAGTGCAAAAGTAATTGCAGTTTTTGCGTTACTTTCAATCGTAAAAACTGCAATTACTTTCACACCAATCT |
| hsa-miR-3144-5p | AGGGGACCAAAGAGATATATAG | AACTACACTTTAAGGGGACCAAAGAGATATATAGATATCAGCTACCTATATACCTGTTCGGTCTCTTTAAAGTGTAGTT |
| hsa-let-7d-3p | CTATACGACCTGCTGCCTTTCT | CCTAGGAAGAGGTAGTAGGTTGCATAGTTTTAGGGCAGGGATTTTGCCCACAAGGAGGTAACTATACGACCTGCTGCCTTTCTTAGG |
| hsa-miR-520g-5p | TCTAGAGGAAGCACTTTCTGTTT | TCCCATGCTGTGACCCTCTAGAGGAAGCACTTTCTGTTTGTTGTCTGAGAAAAAACAAAGTGCTTCCCTTTAGAGTGTTACCGTTTGGGA |
| hsa-miR-7854-3p | TGAGGTGACCGCAGATGGGAA | TTCCTTCCATCTCCATCACCTTGAGCATCTTCTGGGCAGCTGAGGTGACCGCAGATGGGAAGGAA |
| hsa-miR-3652 | CGGCTGGAGGTGTGAGGA | CGGCTGGAGGTGTGAGGATCCGAACCCAGGGGTGGGGGGTGGAGGCGGCTCCTGCGATCGAAGGGGACTTGAGACTCACCGGCCGCACGCCATGAGGGCCCTGTGGGTGCTGGGCCTCTGCTGCGTCCTGC |
| hsa-miR-1275 | GTGGGGGAGAGGCTGTC | CCTCTGTGAGAAAGGGTGTGGGGGAGAGGCTGTCTTGTGTCTGTAAGTATGCCAAACTTATTTTCCCCAAGGCAGAGGGA |
| hsa-miR-500a-3p | ATGCACCTGGGCAAGGATTCTG | GCTCCCCCTCTCTAATCCTTGCTACCTGGGTGAGAGTGCTGTCTGAATGCAATGCACCTGGGCAAGGATTCTGAGAGCGAGAGC |
| hsa-miR-498-5p | TTTCAAGCCAGGGGGCGTTTTTC | AACCCTCCTTGGGAAGTGAAGCTCAGGCTGTGATTTCAAGCCAGGGGGCGTTTTTCTATAACTGGATGAAAAGCACCTCCAGAGCTTGAAGCTCACAGTTTGAGAGCAATCGTCTAAGGAAGTT |
| hsa-miR-552-3p | AACAGGTGACTGGTTAGACAA | AACCATTCAAATATACCACAGTTTGTTTAACCTTTTGCCTGTTGGTTGAAGATGCCTTTCAACAGGTGACTGGTTAGACAAACTGTGGTATATACA |
| hsa-miR-22-3p | AAGCTGCCAGTTGAAGAACTGT | GGCTGAGCCGCAGTAGTTCTTCAGTGGCAAGCTTTATGTCCTGACCCAGCTAAAGCTGCCAGTTGAAGAACTGTTGCCCTCTGCC |
| hsa-miR-219a-2-3p | AGAATTGTGGCTGGACATCTGT | ACTCAGGGGCTTCGCCACTGATTGTCCAAACGCAATTCTTGTACGAGTCTGCGGCCAACCGAGAATTGTGGCTGGACATCTGTGGCTGAGCTCCGGG |
| hsa-miR-411-5p | TAGTAGACCGTATAGCGTACG | TGGTACTTGGAGAGATAGTAGACCGTATAGCGTACGCTTTATCTGTGACGTATGTAACACGGTCCACTAACCCTCAGTATCAAATCCATCCCCGAG |
| hsa-miR-570-5p | AAAGGTAATTGCAGTTTTTCCC | CTAGATAAGTTATTAGGTGGGTGCAAAGGTAATTGCAGTTTTTCCCATTATTTTAATTGCGAAAACAGCAATTACCTTTGCACCAACCTGATGGAGT |
| hsa-miR-6884-3p | CCCATCACCTTTCCGTCTCCCCT | CCCGCAGAGGCTGAGAAGGTGATGTTGGCTCAAGAAAGGGAGATAGATGGTAGCCCATCACCTTTCCGTCTCCCCTAG |
| hsa-miR-891b | TGCAACTTACCTGAGTCATTGA | CCTTAATCCTTGCAACTTACCTGAGTCATTGATTCAGTAAAACATTCAATGGCACATGTTTGTTGTTAGGGTCAAAAGA |
| hsa-miR-3606-5p | TTAGTGAAGGCTATTTTAATT | TTGTTGCTATCTAGGTTAGTGAAGGCTATTTTAATTTTTTTAAAATTTCTTTCACTACTTAGG |
| hsa-miR-1247-3p | CCCCGGGAACGTCGAGACTGGAGC | CCGCTTGCCTCGCCCAGCGCAGCCCCGGCCGCTGGGCGCACCCGTCCCGTTCGTCCCCGGACGTTGCTCTCTACCCCGGGAACGTCGAGACTGGAGCGCCCGAACTGAGCCACCTTCGCGGACCCCGAGAGCGGCG |
| hsa-miR-5090 | CCGGGGCAGATTGGTGTAGGGTG | TCTGAGGTACCCGGGGCAGATTGGTGTAGGGTGCAAAGCCTGCCCGCCCCCTAAGCCTTCTGCCCCCAACTCCAGCCTGTCAGGA |
| hsa-miR-365a-3p | TAATGCCCCTAAAAATCCTTAT | ACCGCAGGGAAAATGAGGGACTTTTGGGGGCAGATGTGTTTCCATTCCACTATCATAATGCCCCTAAAAATCCTTATTGCTCTTGCA |
| hsa-miR-3179 | AGAAGGGGTGAAATTTAAACGT | CAGGATCACAGACGTTTAAATTACACTCCTTCTGCTGTGCCTTACAGCAGTAGAAGGGGTGAAATTTAAACGTCTGTGATCCTG |
| hsa-miR-3152-5p | ATTGCCTCTGTTCTAACACAAG | GTGCAGAGTTATTGCCTCTGTTCTAACACAAGACTAGGCTTCCCTGTGTTAGAATAGGGGCAATAACTCTGCAC |
| hsa-miR-744-3p | CTGTTGCCACTAACCTCAACCT | TTGGGCAAGGTGCGGGGCTAGGGCTAACAGCAGTCTTACTGAAGGTTTCCTGGAAACCACGCACATGCTGTTGCCACTAACCTCAACCTTACTCGGTC |
| hsa-miR-138-2-3p | GCTATTTCACGACACCAGGGTT | CGTTGCTGCAGCTGGTGTTGTGAATCAGGCCGACGAGCAGCGCATCCTCTTACCCGGCTATTTCACGACACCAGGGTTGCATCA |
| hsa-miR-502-3p | AATGCACCTGGGCAAGGATTCA | TGCTCCCCCTCTCTAATCCTTGCTATCTGGGTGCTAGTGCTGGCTCAATGCAATGCACCTGGGCAAGGATTCAGAGAGGGGGAGCT |
| hsa-miR-3937 | ACAGGCGGCTGTAGCAATGGGGG | AGAAGAATGCCCAACCAGCCCTCAGTTGCTACAGTTCCCTGTTGTTTCAGCTCGACAACAACAGGCGGCTGTAGCAATGGGGGGCTGGATGGGCATCTCAATGTGC |
| hsa-miR-3064-5p | TCTGGCTGTTGTGGTGTGCAA | GGTCTGGCTGTTGTGGTGTGCAAAACTCCGTACATTGCTATTTTGCCACACTGCAACACCTTACAG |
| hsa-miR-509-3-5p | TACTGCAGACGTGGCAATCATG | GTGGTACCCTACTGCAGACGTGGCAATCATGTATAATTAAAAATGATTGGTACGTCTGTGGGTAGAGTACTGCAT |
| hsa-miR-889-5p | AATGGCTGTCCGTAGTATGGTC | GTGCTTAAAGAATGGCTGTCCGTAGTATGGTCTCTATATTTATGATGATTAATATCGGACAACCATTGTTTTAGTATCC |
| hsa-miR-3918 | ACAGGGCCGCAGATGGAGACT | AGGCGGTTAAGCCATGGGACAGGGCCGCAGATGGAGACTGCTCAAGGTCAAAGGGGTCTCCAGCTGGGACCCTGCACCTGGTTCGTAGCCCCT |
| hsa-miR-4755-5p | TTTCCCTTCAGAGCCTGGCTTT | AGATTCAGCTTTCCCTTCAGAGCCTGGCTTTGGCATCTATGAAAGCCAGGCTCTGAAGGGAAAGTTGAATCT |
| hsa-miR-3619-3p | GGGACCATCCTGCCTGCTGTGG | ACGGCATCTTTGCACTCAGCAGGCAGGCTGGTGCAGCCCGTGGTGGGGGACCATCCTGCCTGCTGTGGGGTAAGGACGGCTGT |
| hsa-miR-500b-5p | AATCCTTGCTACCTGGGT | CCCCCTCTCTAATCCTTGCTACCTGGGTGAGAGTGCTTTCTGAATGCAGTGCACCCAGGCAAGGATTCTGCAAGGGGGA |
| hsa-miR-192-3p | CTGCCAATTCCATAGGTCACAG | GCCGAGACCGAGTGCACAGGGCTCTGACCTATGAATTGACAGCCAGTGCTCTCGTCTCCCCTCTGGCTGCCAATTCCATAGGTCACAGGTATGTTCGCCTCAATGCCAGC |
| hsa-miR-517a-3p | ATCGTGCATCCCTTTAGAGTGT | TCTCAGGCAGTGACCCTCTAGATGGAAGCACTGTCTGTTGTATAAAAGAAAAGATCGTGCATCCCTTTAGAGTGTTACTGTTTGAGA |
| hsa-miR-873-5p | GCAGGAACTTGTGAGTCTCCT | GTGTGCATTTGCAGGAACTTGTGAGTCTCCTATTGAAAATGAACAGGAGACTGATGAGTTCCCGGGAACACCCACAA |
| hsa-miR-2114-5p | TAGTCCCTTCCTTGAAGCGGTC | CCTCCATGCTCCTAGTCCCTTCCTTGAAGCGGTCGGATAATCACATGACGAGCCTCAAGCAAGGGACTTCAAGCTGGTGG |
| hsa-miR-222-5p | CTCAGTAGCCAGTGTAGATCCT | GCTGCTGGAAGGTGTAGGTACCCTCAATGGCTCAGTAGCCAGTGTAGATCCTGTCTTTCGTAATCAGCAGCTACATCTGGCTACTGGGTCTCTGATGGCATCTTCTAGCT |
| hsa-miR-542-3p | TGTGACAGATTGATAACTGAAA | CAGATCTCAGACATCTCGGGGATCATCATGTCACGAGATACCAGTGTGCACTTGTGACAGATTGATAACTGAAAGGTCTGGGAGCCACTCATCTTCA |
| hsa-miR-4768-5p | ATTCTCTCTGGATCCCATGGAT | AAACTTTGATTCTCTCTGGATCCCATGGATATGGGAACTGTGATGTCCAGGAGATCCAGAGAGAATCAGAGTTT |
| hsa-miR-589-5p | TGAGAACCACGTCTGCTCTGAG | TCCAGCCTGTGCCCAGCAGCCCCTGAGAACCACGTCTGCTCTGAGCTGGGTACTGCCTGTTCAGAACAAATGCCGGTTCCCAGACGCTGCCAGCTGGCC |
| hsa-miR-16-1-3p | CCAGTATTAACTGTGCTGCTGA | GTCAGCAGTGCCTTAGCAGCACGTAAATATTGGCGTTAAGATTCTAAAATTATCTCCAGTATTAACTGTGCTGCTGAAGTAAGGTTGAC |
| hsa-miR-181c-3p | AACCATCGACCGTTGAGTGGAC | CGGAAAATTTGCCAAGGGTTTGGGGGAACATTCAACCTGTCGGTGAGTTTGGGCAGCTCAGGCAAACCATCGACCGTTGAGTGGACCCTGAGGCCTGGAATTGCCATCCT |
| hsa-miR-517a-3p | ATCGTGCATCCCTTTAGAGTGT | GTGACCCTCTAGATGGAAGCACTGTCTGTTGTCTAAGAAAAGATCGTGCATCCCTTTAGAGTGTTAC |
| hsa-miR-624-3p | CACAAGGTATTGGTATTACCT | AATGCTGTTTCAAGGTAGTACCAGTACCTTGTGTTCAGTGGAACCAAGGTAAACACAAGGTATTGGTATTACCTTGAGATAGCATTACACCTAAGTG |
| hsa-miR-548o-3p | CCAAAACTGCAGTTACTTTTGC | TGGTGAAAATGTGTTGATTGTAATGGTTCCTATTCTGATCAATAAACATGGTTTGAGCCTAGTTACAATGATCTAAAATTCACGGTCCAAAACTGCAGTTACTTTTGCACCAAC |
| hsa-miR-4777-3p | ATACCTCATCTAGAATGCTGTA | TAGAATATTTCGGCATTCTAGATGAGAGATATATATATACCTCATATGTATATGGTATACCTCATCTAGAATGCTGTAATATTCTA |
| hsa-miR-181a-2-3p | ACCACTGACCGTTGACTGTACC | AGAAGGGCTATCAGGCCAGCCTTCAGAGGACTCCAAGGAACATTCAACGCTGTCGGTGAGTTTGGGATTTGAAAAAACCACTGACCGTTGACTGTACCTTGGGGTCCTTA |
| hsa-miR-6814-5p | TCCCAAGGGTGAGATGCTGCCA | TTTCCTCCCAAGGGTGAGATGCTGCCACCCAGCCCTGCAGAGCCCCTGACTCGCATCCTTCCCTTGGCAG |
| hsa-miR-4732-3p | GCCCTGACCTGTCCTGTTCTG | GAGGGAGCTGTAGAGCAGGGAGCAGGAAGCTGTGTGTGTCCAGCCCTGACCTGTCCTGTTCTGCCCCCAGCCCCTC |
| hsa-miR-335-3p | TTTTTCATTATTGCTCCTGACC | TGTTTTGAGCGGGGGTCAAGAGCAATAACGAAAAATGTTTGTCATAAACCGTTTTTCATTATTGCTCCTGACCTCCTCTCATTTGCTATATTCA |
| hsa-miR-5581-5p | AGCCTTCCAGGAGAAATGGAGA | AGCCTTCCAGGAGAAATGGAGACCCTATACATACCTGTTTCCATGCCTCCTAGAAGTTCC |
| hsa-miR-27a-5p | AGGGCTTAGCTGCTTGTGAGCA | CTGAGGAGCAGGGCTTAGCTGCTTGTGAGCAGGGTCCACACCAAGTCGTGTTCACAGTGGCTAAGTTCCGCCCCCCAG |
| hsa-miR-942-3p | CACATGGCCGAAACAGAGAAGT | ATTAGGAGAGTATCTTCTCTGTTTTGGCCATGTGTGTACTCACAGCCCCTCACACATGGCCGAAACAGAGAAGTTACTTTCCTAAT |
| hsa-miR-376a-2-5p | GGTAGATTTTCCTTCTATGGT | GGTATTTAAAAGGTAGATTTTCCTTCTATGGTTACGTGTTTGATGGTTAATCATAGAGGAAAATCCACGTTTTCAGTATC |
| hsa-miR-1245a | AAGTGATCTAAAGGCCTACAT | ATTTATGTATAGGCCTTTAGATCATCTGATGTTGAATACTCTTTAAGTGATCTAAAGGCCTACATATAAA |
| hsa-miR-6884-5p | AGAGGCTGAGAAGGTGATGTTG | CCCGCAGAGGCTGAGAAGGTGATGTTGGCTCAAGAAAGGGAGATAGATGGTAGCCCATCACCTTTCCGTCTCCCCTAG |
| hsa-miR-517-5p | CCTCTAGATGGAAGCACTGTCT | TCTCAGGCAGTGACCCTCTAGATGGAAGCACTGTCTGTTGTATAAAAGAAAAGATCGTGCATCCCTTTAGAGTGTTACTGTTTGAGA |
| hsa-miR-4635 | TCTTGAAGTCAGAACCCGCAA | CCGGGACTTTGTGGGTTCTGACCCCACTTGGATCACGCCGACAACACTGGTCTTGAAGTCAGAACCCGCAAAGTCCTGG |
| hsa-miR-4797-5p | GACAGAGTGCCACTTACTGAA | GACTCAGAAGACAGAGTGCCACTTACTGAAAGGTTTTTTCTCTCAGTAAGTGGCACTCTGTCTTCTGAGTT |
| hsa-miR-18b-5p | TAAGGTGCATCTAGTGCAGTTAG | TGTGTTAAGGTGCATCTAGTGCAGTTAGTGAAGCAGCTTAGAATCTACTGCCCTAAATGCCCCTTCTGGCA |
| hsa-miR-18a-5p | TAAGGTGCATCTAGTGCAGATAG | TGTTCTAAGGTGCATCTAGTGCAGATAGTGAAGTAGATTAGCATCTACTGCCCTAAGTGCTCCTTCTGGCA |
| hsa-miR-3943 | TAGCCCCCAGGCTTCACTTGGCG | CACACAGACGGCAGCTGCGGCCTAGCCCCCAGGCTTCACTTGGCGTGGACAACTTGCTAAGTAAAGTGGGGGGTGGGCCACGGCTGGCTCCTACCTGGAC |
| hsa-miR-6507-3p | CAAAGTCCTTCCTATTTTTCCC | GGAGGGAAGAATAGGAGGGACTTTGTATTGTGGTTCAGTACCATGCAAAGTCCTTCCTATTTTTCCCTCC |
| hsa-miR-378j | ACTGGATTTGGAGCCAGAA | ATGCAGTGAGTCGGGGAGGAACTGGATTTGGAGCCAGAAGAACTGGTTCTAATATCTACTTCCCTGTGTAGAGTTGGGATTTGGGATTATATGAGTTAATATACACCAA |
| hsa-miR-7-1-3p | CAACAAATCACAGTCTGCCATA | TTGGATGTTGGCCTAGTTCTGTGTGGAAGACTAGTGATTTTGTTGTTTTTAGATAACTAAATCGACAACAAATCACAGTCTGCCATATGGCACAGGCCATGCCTCTACAG |
| hsa-miR-6839-5p | TCTGGATTGAAGAGACGACCCA | TGTAGTCTGGATTGAAGAGACGACCCAAGCAGGCTTTGTGTGAGCAGTGAGGCTATTTATTCACTTGGGTGCGAGCTCACACGAAGCCTGCTTGGGTTTTCTCTTCAATCCAG |
| hsa-miR-6788-3p | TTCGCCACTTCCCTCCCTGCAG | GACGGCTGGGAGAAGAGTGGTGAAGAAGAGTATTGATTGTGCTGTTCGCCACTTCCCTCCCTGCAG |
| hsa-miR-337-5p | GAACGGCTTCATACAGGAGTT | GTAGTCAGTAGTTGGGGGGTGGGAACGGCTTCATACAGGAGTTGATGCACAGTTATCCAGCTCCTATATGATGCCTTTCTTCATCCCCTTCAA |
| hsa-miR-374a-5p | TTATAATACAACCTGATAAGTG | TACATCGGCCATTATAATACAACCTGATAAGTGTTATAGCACTTATCAGATTGTATTGTAATTGTCTGTGTA |
| hsa-miR-6513-3p | TCAAGTGTCATCTGTCCCTAG | GCTTTGGGATTGACGCCACATGTCTCAGGTCCCCAGCTGAGTCAAGTGTCATCTGTCCCTAGGC |
| hsa-miR-4524a-5p | ATAGCAGCATGAACCTGTCTCA | GAACGATAGCAGCATGAACCTGTCTCACTGCAGAATTATTTTGAGACAGGCTTATGCTGCTATCCTTCA |
| hsa-miR-28-3p | CACTAGATTGTGAGCTCCTGGA | GGTCCTTGCCCTCAAGGAGCTCACAGTCTATTGAGTTACCTTTCTGACTTTCCCACTAGATTGTGAGCTCCTGGAGGGCAGGCACT |
| hsa-miR-4640-5p | TGGGCCAGGGAGCAGCTGGTGGG | CTGTGGGCTGGGCCAGGGAGCAGCTGGTGGGTGGGAAGTAAGATCTGACCTGGACTCCATCCCACCCACCCCCTGTTTCCTGGCCCACAG |
| hsa-miR-3611 | TTGTGAAGAAAGAAATTCTTA | AGCAGGTCTAATAAGAATTTCTTTTTCTTCACAATTATGAAAGAAAAGAAATTGTGAAGAAAGAAATTCTTACTAGTTTTGCT |
| hsa-miR-10a-5p | TACCCTGTAGATCCGAATTTGTG | GATCTGTCTGTCTTCTGTATATACCCTGTAGATCCGAATTTGTGTAAGGAATTTTGTGGTCACAAATTCGTATCTAGGGGAATATGTAGTTGACATAAACACTCCGCTCT |
| hsa-miR-29b-2-5p | CTGGTTTCACATGGTGGCTTAG | CTTCTGGAAGCTGGTTTCACATGGTGGCTTAGATTTTTCCATCTTTGTATCTAGCACCATTTGAAATCAGTGTTTTAGGAG |
| hsa-miR-4727-3p | ATAGTGGGAAGCTGGCAGATTC | AATCTGCCAGCTTCCACAGTGGCAGATTTTCCCATAGTGGGAAGCTGGCAGATTC |
| hsa-miR-561-3p | CAAAGTTTAAGATCCTTGAAGT | CTTCATCCACCAGTCCTCCAGGAACATCAAGGATCTTAAACTTTGCCAGAGCTACAAAGGCAAAGTTTAAGATCCTTGAAGTTCCTGGGGGAACCAT |
| hsa-miR-514b-5p | TTCTCAAGAGGGAGGCAATCAT | CATGTGGTACTCTTCTCAAGAGGGAGGCAATCATGTGTAATTAGATATGATTGACACCTCTGTGAGTGGAGTAACACATG |
| hsa-miR-6516-5p | TTTGCAGTAACAGGTGTGAGCA | TGGGTTTTGAATTTGCAGTAACAGGTGTGAGCATTCTAGCAGCAGTTTGATGATCATGTATGATACTGCAAACAGGACCTA |
| hsa-miR-3146 | CATGCTAGGATAGAAAGAATGG | GCTAAGTCCCTTCTTTCTATCCTAGTATAACTTGAAGAATTCAAATAGTCATGCTAGGATAGAAAGAATGGGACTTGGC |
| hsa-miR-4707-3p | AGCCCGCCCCAGCCGAGGTTCT | GGTTCCGGAGCCCCGGCGCGGGCGGGTTCTGGGGTGTAGACGCTGCTGGCCAGCCCGCCCCAGCCGAGGTTCTCGGCACC |
| hsa-miR-10396b-3p | GGCCCCGGGCCCTCGACCGGAC | CGGCGGGGCTCGGAGCCGGGCTTCGGCCGGGCCCCGGGCCCTCGACCGGAC |
| hsa-miR-510-5p | TACTCAGGAGAGTGGCAATCAC | GTGGTGTCCTACTCAGGAGAGTGGCAATCACATGTAATTAGGTGTGATTGAAACCTCTAAGAGTGGAGTAACAC |
| hsa-miR-4726-5p | AGGGCCAGAGGAGCCTGGAGTGG | AGGGCCAGAGGAGCCTGGAGTGGTCGGGTCGACTGAACCCAGGTTCCCTCTGGCCGCA |
| hsa-miR-490-3p | CAACCTGGAGGACTCCATGCTG | TGGAGGCCTTGCTGGTTTGGAAAGTTCATTGTTCGACACCATGGATCTCCAGGTGGGTCAAGTTTAGAGATGCACCAACCTGGAGGACTCCATGCTGTTGAGCTGTTCACAAGCAGCGGACACTTCCA |
| hsa-miR-4726-3p | ACCCAGGTTCCCTCTGGCCGCA | AGGGCCAGAGGAGCCTGGAGTGGTCGGGTCGACTGAACCCAGGTTCCCTCTGGCCGCA |
| hsa-miR-559 | TAAAGTAAATATGCACCAAAA | GCTCCAGTAACATCTTAAAGTAAATATGCACCAAAATTACTTTTGGTAAATACAGTTTTGGTGCATATTTACTTTAGGATGTTACTGGAGCTCCCA |
| hsa-miR-101-3p | TACAGTACTGTGATAACTGAA | TGCCCTGGCTCAGTTATCACAGTGCTGATGCTGTCTATTCTAAAGGTACAGTACTGTGATAACTGAAGGATGGCA |
| hsa-miR-127-5p | CTGAAGCTCAGAGGGCTCTGAT | TGTGATCACTGTCTCCAGCCTGCTGAAGCTCAGAGGGCTCTGATTCAGAAAGATCATCGGATCCGTCTGAGCTTGGCTGGTCGGAAGTCTCATCATC |
| hsa-miR-193b-3p | AACTGGCCCTCAAAGTCCCGCT | GTGGTCTCAGAATCGGGGTTTTGAGGGCGAGATGAGTTTATGTTTTATCCAACTGGCCCTCAAAGTCCCGCTTTTGGGGTCAT |
| hsa-miR-589-3p | TCAGAACAAATGCCGGTTCCCAGA | TCCAGCCTGTGCCCAGCAGCCCCTGAGAACCACGTCTGCTCTGAGCTGGGTACTGCCTGTTCAGAACAAATGCCGGTTCCCAGACGCTGCCAGCTGGCC |
| hsa-miR-6861-3p | TGGACCTCTCCTCCCCAG | GAGGCACTGGGTAGGTGGGGCTCCAGGGCTCCTGACACCTGGACCTCTCCTCCCCAGGCCCACA |
| hsa-miR-520e-5p | CTCAAGATGGAAGCAGTTTCTG | TCTCCTGCTGTGACCCTCAAGATGGAAGCAGTTTCTGTTGTCTGAAAGGAAAGAAAGTGCTTCCTTTTTGAGGGTTACTGTTTGAGA |
| hsa-miR-520a-3p | AAAGTGCTTCCCTTTGGACTGT | CTCAGGCTGTGACCCTCCAGAGGGAAGTACTTTCTGTTGTCTGAGAGAAAAGAAAGTGCTTCCCTTTGGACTGTTTCGGTTTGAG |
| hsa-miR-3192-5p | TCTGGGAGGTTGTAGCAGTGGAA | GGAAGGGATTCTGGGAGGTTGTAGCAGTGGAAAAAGTTCTTTTCTTCCTCTGATCGCCCTCTCAGCTCTTTCCTTCT |
| hsa-miR-6836-3p | ATGCCTCCCCCGGCCCCGCAG | GGCTCCGCAGGGCCCTGGCGCAGGCATCCAGACAGCGGGCGAATGCCTCCCCCGGCCCCGCAG |
| hsa-miR-597-3p | TGGTTCTCTTGTGGCTCAAGCGT | TACTTACTCTACGTGTGTGTCACTCGATGACCACTGTGAAGACAGTAAAATGTACAGTGGTTCTCTTGTGGCTCAAGCGTAATGTAGAGTACTGGTC |
| hsa-miR-548g-5p | TGCAAAAGTAATTGCAGTTTTTG | AGGTTAGTGCAAAAGTAATTGCAGTTTTTGCGTTACTTTCAATCGTAAAAACTGCAATTACTTTCACACCAATCT |
| hsa-miR-6888-3p | ATCTGTCTCGATTGTTTCCAG | GTGGGAAGGAGATGCTCAGGCAGATCTGTCTCTGATTGTTTCCAAGATCTGTCTCGATTGTTTCCAG |
| hsa-miR-6793-5p | TGTGGGTTCTGGGTTGGGGTGA | GTCACTGTGGGTTCTGGGTTGGGGTGATACACAAGCCTGACCCTCCCCAACCCCTGCCCGCAG |
| hsa-miR-8075 | TGCTGATGGCAGATGTCGGGTCTG | CCTTGCTGATGGCAGATGTCGGATCTGCCTCGCTTATACGTGCCCTTGCTGATGGCAGATGTCGGGTCTGCCTCGCTTAT |
| hsa-miR-24-3p | TGGCTCAGTTCAGCAGGAACAG | CTCCGGTGCCTACTGAGCTGATATCAGTTCTCATTTTACACACTGGCTCAGTTCAGCAGGAACAGGAG |
| hsa-miR-6825-5p | TGGGGAGGTGTGGAGTCAGCAT | GGGCATGGGGAGGTGTGGAGTCAGCATGGGGCTAGGAGGCCCCGCGCTGACCCGCCTTCTCCGCAG |
| hsa-miR-3934-5p | TCAGGTGTGGAAACTGAGGCAG | CACAGCCCTTCCTGTCCCCAGTTTTCAGGTGTGGAAACTGAGGCAGGAGGCAGTGAAGTAACTTGCTCAGGTTGCACAGCTGGGAAGTGGAGCAGGGATTTGAATCC |
| hsa-miR-1238-3p | CTTCCTCGTCTGTCTGCCCC | GTGAGTGGGAGCCCCAGTGTGTGGTTGGGGCCATGGCGGGTGGGCAGCCCAGCCTCTGAGCCTTCCTCGTCTGTCTGCCCCAG |
| hsa-miR-598-3p | TACGTCATCGTTGTCATCGTCA | GCTTGATGATGCTGCTGATGCTGGCGGTGATCCCGATGGTGTGAGCTGGAAATGGGGTGCTACGTCATCGTTGTCATCGTCATCATCATCATCCGAG |
| hsa-miR-6773-5p | TTGGGCCCAGGAGTAAACAGGAT | GGGAGTTGGGCCCAGGAGTAAACAGGATTAGTGCTTATGCTAAGATGTCTTCACTGTCACTTCTCTGCCCATAG |
| hsa-miR-548an | AAAAGGCATTGTGGTTTTTG | CATTAGGTTGGTGCAAAAGGCATTGTGGTTTTTGCCTATAAAAGTAATGGCAAAAACCGCAATTCCTTTTGCACCAACCTAAT |
| hsa-miR-134-5p | TGTGACTGGTTGACCAGAGGGG | CAGGGTGTGTGACTGGTTGACCAGAGGGGCATGCACTGTGTTCACCCTGTGGGCCACCTAGTCACCAACCCTC |
| hsa-miR-4512 | CAGGGCCTCACTGTATCGCCCA | CTCAGCCCGGGCAATATAGTGAGACCTCGTCTCTACAAAAAATTGAGACAGGGCCTCACTGTATCGCCCAGGCTGGA |
| hsa-miR-6733-5p | TGGGAAAGACAAACTCAGAGTT | GTGCTTGGGAAAGACAAACTCAGAGTTCCCTTCTTGTGAGCTCAGTGTCTGGATTTCCTAG |
| hsa-miR-182-3p | TGGTTCTAGACTTGCCAACTA | GAGCTGCTTGCCTCCCCCCGTTTTTGGCAATGGTAGAACTCACACTGGTGAGGTAACAGGATCCGGTGGTTCTAGACTTGCCAACTATGGGGCGAGGACTCAGCCGGCAC |
| hsa-miR-625-3p | GACTATAGAACTTTCCCCCTCA | AGGGTAGAGGGATGAGGGGGAAAGTTCTATAGTCCTGTAATTAGATCTCAGGACTATAGAACTTTCCCCCTCATCCCTCTGCCCT |
| hsa-miR-616-5p | ACTCAAAACCCTTCAGTGACTT | TTAGGTAATTCCTCCACTCAAAACCCTTCAGTGACTTCCATGACATGAAATAGGAAGTCATTGGAGGGTTTGAGCAGAGGAATGACCTGTTTTAAAA |
| hsa-miR-138-5p | AGCTGGTGTTGTGAATCAGGCCG | CGTTGCTGCAGCTGGTGTTGTGAATCAGGCCGACGAGCAGCGCATCCTCTTACCCGGCTATTTCACGACACCAGGGTTGCATCA |
| hsa-miR-4448 | GGCTCCTTGGTCTAGGGGTA | AGGAGTGACCAAAAGACAAGAGTGCGAGCCTTCTATTATGCCCAGACAGGGCCACCAGAGGGCTCCTTGGTCTAGGGGTAATGCCA |
| hsa-miR-4430 | AGGCTGGAGTGAGCGGAG | GTGAGGCTGGAGTGAGCGGAGATCGTACCACTGCACTCCAACCTGGTGA |
| hsa-miR-3065-3p | TCAGCACCAGGATATTGTTGGAG | CTGCCCTCTTCAACAAAATCACTGATGCTGGAGTCGCCTGAGTCATCACTCAGCACCAGGATATTGTTGGAGAGGACAG |
| hsa-miR-3117-3p | ATAGGACTCATATAGTGCCAG | CCCTAAAGGGCCAGACACTATACGAGTCATATAAGGGAAGGCATTATAGGACTCATATAGTGCCAGGTGTTTTGTGGG |
| hsa-miR-6852-3p | TGTCCTCTGTTCCTCAG | TGCTGCCCTGGGGTTCTGAGGACATGCTCTGACTCCCCTGATGTCCTCTGTTCCTCAGGTGCTGGG |
| hsa-miR-6505-5p | TTGGAATAGGGGATATCTCAGC | GCATTGGAATAGGGGATATCTCAGCATGTTGAGCCCTGTCTCTGGGGAGCTGACTTCTACCTCTTCCAAAG |
| hsa-miR-365a-5p | AGGGACTTTTGGGGGCAGATGTG | ACCGCAGGGAAAATGAGGGACTTTTGGGGGCAGATGTGTTTCCATTCCACTATCATAATGCCCCTAAAAATCCTTATTGCTCTTGCA |
| hsa-miR-380-3p | TATGTAATATGGTCCACATCTT | AAGATGGTTGACCATAGAACATGCGCTATCTCTGTGTCGTATGTAATATGGTCCACATCTT |
| hsa-miR-539-3p | ATCATACAAGGACAATTTCTTT | ATACTTGAGGAGAAATTATCCTTGGTGTGTTCGCTTTATTTATGATGAATCATACAAGGACAATTTCTTTTTGAGTAT |
| hsa-miR-4487 | AGAGCTGGCTGAAGGGCAG | ACTGTCCTTCAGCCAGAGCTGGCTGAAGGGCAGAAGGGAACTGTCCTTCAGCCAGAGCTGGCTGAAGGGCAGA |
| hsa-miR-548n | CAAAAGTAATTGTGGATTTTGT | AGGTTGGTGCAAAAGTAATTGTGGATTTTGTCGTTAAAAATAGCAAAACCCGCAATTACTTTTGCACCAACCTAA |
| hsa-miR-892b | CACTGGCTCCTTTCTGGGTAGA | TGCAATGCCCTACTCAGAAAGGTGCCATTTATGTAGATTTTATGTCACTGGCTCCTTTCTGGGTAGAGCAAGGCTCA |
| hsa-miR-6805-5p | TAGGGGGCGGCTTGTGGAGTGT | TGGCCTAGGGGGCGGCTTGTGGAGTGTATGGGCTGAGCCTTGCTCTGCTCCCCCGCCCCCAG |
| hsa-miR-1245b-5p | TAGGCCTTTAGATCACTTAAA | TTTATATGTAGGCCTTTAGATCACTTAAAGAGTATTCAACATCAGATGATCTAAAGGCCTATACATAAA |
| hsa-let-7e-3p | CTATACGGCCTCCTAGCTTTCC | CCCGGGCTGAGGTAGGAGGTTGTATAGTTGAGGAGGACACCCAAGGAGATCACTATACGGCCTCCTAGCTTTCCCCAGG |
| hsa-miR-659-5p | AGGACCTTCCCTGAACCAAGGA | TACCGACCCTCGATTTGGTTCAGGACCTTCCCTGAACCAAGGAAGAGTCACAGTCTCTTCCTTGGTTCAGGGAGGGTCCCCAACAATGTCCTCATGG |
| hsa-miR-3191-5p | CTCTCTGGCCGTCTACCTTCCA | GGGGTCACCTCTCTGGCCGTCTACCTTCCACACTGACAAGGGCCGTGGGGACGTAGCTGGCCAGACAGGTGACCCC |
| hsa-miR-3180-3p | TGGGGCGGAGCTTCCGGAGGCC | CAGTGCGACGGGCGGAGCTTCCAGACGCTCCGCCCCACGTCGCATGCGCCCCGGGAAAGCGTGGGGCGGAGCTTCCGGAGGCCCCGCCCTGCTG |
| hsa-miR-2467-5p | TGAGGCTCTGTTAGCCTTGGCTC | GGACAGGCACCTGAGGCTCTGTTAGCCTTGGCTCTGGGTCCTGCTCCTTAGAGCAGAGGCAGAGAGGCTCAGGGTCTGTCT |
| hsa-miR-6872-3p | CCCATGCCTCCTGCCGCGGTC | GTGGGTCTCGCATCAGGAGGCAAGGCCAGGACCCGCTGACCCATGCCTCCTGCCGCGGTCAG |
| hsa-miR-1288-3p | TGGACTGCCCTGATCTGGAGA | GAGGGTGTTGATCAGCAGATCAGGACTGTAACTCACCATAGTGGTGGACTGCCCTGATCTGGAGACCACTGCCTT |
| hsa-miR-3679-5p | TGAGGATATGGCAGGGAAGGGGA | CGTGGTGAGGATATGGCAGGGAAGGGGAGTTTCCCTCTATTCCCTTCCCCCCAGTAATCTTCATCATG |
| hsa-miR-4494 | CCAGACTGTGGCTGACCAGAGG | AGTTTTAGTTACCCTGGTCATCTGCAGTCTGAAAATACAAAATGGAAAATTCCAGACTGTGGCTGACCAGAGGTAACTGAAACC |
| hsa-miR-548c-5p | AAAAGTAATTGCGGTTTTTGCC | CATTGGCATCTATTAGGTTGGTGCAAAAGTAATTGCGGTTTTTGCCATTACTTTCAGTAGCAAAAATCTCAATTACTTTTGCACCAACTTAATACTT |
| hsa-miR-128-1-5p | CGGGGCCGTAGCACTGTCTGAGA | TGAGCTGTTGGATTCGGGGCCGTAGCACTGTCTGAGAGGTTTACATTTCTCACAGTGAACCGGTCTCTTTTTCAGCTGCTTC |
| hsa-miR-7704 | CGGGGTCGGCGGCGACGTG | CGGGGTCGGCGGCGACGTGCTCAGCTTGGCACCCAAGTTCTGCCGCTCCGACGCCCGGC |
| hsa-miR-664b-3p | TTCATTTGCCTCCCAGCCTACA | TGGGCTAAGGGAGATGATTGGGTAGAAAGTATTATTCTATTCATTTGCCTCCCAGCCTACA |
| hsa-miR-15b-3p | CGAATCATTATTTGCTGCTCTA | TTGAGGCCTTAAAGTACTGTAGCAGCACATCATGGTTTACATGCTACAGTCAAGATGCGAATCATTATTTGCTGCTCTAGAAATTTAAGGAAATTCAT |
| hsa-miR-4697-3p | TGTCAGTGACTCCTGCCCCTTGGT | GGGCCCAGAAGGGGGCGCAGTCACTGACGTGAAGGGACCACATCCCGCTTCATGTCAGTGACTCCTGCCCCTTGGTCT |
| hsa-miR-4645-3p | AGACAGTAGTTCTTGCCTGGTT | TGATAGGGAAACCAGGCAAGAAATATTGTCTCCTCAAGTTGCGACGAGACAGTAGTTCTTGCCTGGTTTCTCTATCA |
| hsa-miR-181b-5p | AACATTCATTGCTGTCGGTGGGT | CCTGTGCAGAGATTATTTTTTAAAAGGTCACAATCAACATTCATTGCTGTCGGTGGGTTGAACTGTGTGGACAAGCTCACTGAACAATGAATGCAACTGTGGCCCCGCTT |
| hsa-miR-196b-5p | TAGGTAGTTTCCTGTTGTTGGG | ACTGGTCGGTGATTTAGGTAGTTTCCTGTTGTTGGGATCCACCTTTCTCTCGACAGCACGACACTGCCTTCATTACTTCAGTTG |
| hsa-miR-219b-5p | AGATGTCCAGCCACAATTCTCG | GGAGCTCAGCCACAGATGTCCAGCCACAATTCTCGGTTGGCCGCAGACTCGTACAAGAATTGCGTTTGGACAATCAGTGGCGAAGCCC |
| hsa-miR-3934-3p | TGCTCAGGTTGCACAGCTGGGA | CACAGCCCTTCCTGTCCCCAGTTTTCAGGTGTGGAAACTGAGGCAGGAGGCAGTGAAGTAACTTGCTCAGGTTGCACAGCTGGGAAGTGGAGCAGGGATTTGAATCC |
| hsa-miR-4746-3p | AGCGGTGCTCCTGCGGGCCGA | GTGTCTGTGCCGGTCCCAGGAGAACCTGCAGAGGCATCGGGTCAGCGGTGCTCCTGCGGGCCGACACTCAC |
| hsa-miR-1266-5p | CCTCAGGGCTGTAGAACAGGGCT | ACAGGTAGTGTCCCTCAGGGCTGTAGAACAGGGCTGGGATTACTAAAGCCCTGTTCTATGCCCTGAGGGACACTGAGCATGTCA |
| hsa-miR-409-5p | AGGTTACCCGAGCAACTTTGCAT | TGGTACTCGGGGAGAGGTTACCCGAGCAACTTTGCATCTGGACGACGAATGTTGCTCGGTGAACCCCTTTTCGGTATCA |
| hsa-miR-508-5p | TACTCCAGAGGGCGTCACTCATG | CCACCTTCAGCTGAGTGTAGTGCCCTACTCCAGAGGGCGTCACTCATGTAAACTAAAACATGATTGTAGCCTTTTGGAGTAGAGTAATACACATCACGTAACGCATATTTGGTGG |
| hsa-miR-3909 | TGTCCTCTAGGGCCTGCAGTCT | GGTATGCTGTTGCGCTGTCCTTCCTCTGGGGAGCAGGCTCCGGGGGACAGGGAAAAGCACACAAGGAACTTGTCCTCTAGGGCCTGCAGTCTCATGGGAGAGTGACATGCACCAGGACC |
| hsa-miR-4504 | TGTGACAATAGAGATGAACATG | CTAAGATAATGTCCTCCAGGTTCATCTCTGTTGTCATTTGTGGCATGGACCATTTGTGACAATAGAGATGAACATGGAGGATATTATCTTAA |
| hsa-miR-3683 | TGCGACATTGGAAGTAGTATCA | GGGTGTACACCCCCTGCGACATTGGAAGTAGTATCATCTCTCCCTTGGATGCTACGAACAATATCACAGAAGGTGTACACCC |
| hsa-miR-4469 | GCTCCCTCTAGGGTCGCTCGGA | CCGACGCGGAGAGCGGCTCTAGGTGGGTTTGGCGGCGGCGAGGACACCGCCGCTCCCTCTAGGGTCGCTCGGAGCGTGA |
| hsa-miR-6804-3p | CGCACCTGCCTCTCACCCACAG | GGATGTGAGGGTGTCAGCAGGTGACGGTGGGGGCCACGCTGACAGCCGCACCTGCCTCTCACCCACAG |
| hsa-miR-510-3p | ATTGAAACCTCTAAGAGTGGA | GTGGTGTCCTACTCAGGAGAGTGGCAATCACATGTAATTAGGTGTGATTGAAACCTCTAAGAGTGGAGTAACAC |
| hsa-miR-624-5p | TAGTACCAGTACCTTGTGTTCA | AATGCTGTTTCAAGGTAGTACCAGTACCTTGTGTTCAGTGGAACCAAGGTAAACACAAGGTATTGGTATTACCTTGAGATAGCATTACACCTAAGTG |
| hsa-miR-548ap-5p | AAAAGTAATTGCGGTCTTT | ACCAATTCCTAGGTTGGTGCAAAAGTAATTGCGGTCTTTGTCATTAAAACCAATAACAAAAACCACAATTACTTTTTACTGACCTAAAGATTAATT |
| hsa-miR-193b-5p | CGGGGTTTTGAGGGCGAGATGA | GTGGTCTCAGAATCGGGGTTTTGAGGGCGAGATGAGTTTATGTTTTATCCAACTGGCCCTCAAAGTCCCGCTTTTGGGGTCAT |
| hsa-miR-1304-5p | TTTGAGGCTACAGTGAGATGTG | AAACACTTGAGCCCAGCGGTTTGAGGCTACAGTGAGATGTGATCCTGCCACATCTCACTGTAGCCTCGAACCCCTGGGCTCAAGTGATTCA |
| hsa-miR-7155-3p | TGGCCCAAGACCTCAGACC | TCTGGGGTCTTGGGCCATCTGGTTGTGACATCACTGATGGCCCAAGACCTCAGACC |
| hsa-miR-550a-5p | AGTGCCTGAGGGAGTAAGAGCCC | TGATGCTTTGCTGGCTGGTGCAGTGCCTGAGGGAGTAAGAGCCCTGTTGTTGTAAGATAGTGTCTTACTCCCTCAGGCACATCTCCAACAAGTCTCT |
| hsa-miR-6510-5p | CAGCAGGGGAGAGAGAGGAGTC | AGCAGCAGGGGAGAGAGAGGAGTCCTCTAGACACCGACTCTGTCTCCTGCAGAT |
| hsa-miR-1268b | CGGGCGTGGTGGTGGGGGTG | ACCCGGGCGTGGTGGTGGGGGTGGGTGCCTGTAATTCCAGCTAGTTGGGA |
| hsa-miR-3605-5p | TGAGGATGGATAGCAAGGAAGCC | ACTTTATACGTGTAATTGTGATGAGGATGGATAGCAAGGAAGCCGCTCCCACCTGACCCTCACGGCCTCCGTGTTACCTGTCCTCTAGGTGGGACGCTCG |
| hsa-miR-6857-5p | TTGGGGATTGGGTCAGGCCAGT | GCTTGTTGGGGATTGGGTCAGGCCAGTGTTCAAGGGCCCCTCCTCTAGTACTCCCTGTTTGTGTTCTGCCACTGACTGAGCTTCTCCCCACAG |
| hsa-miR-10398-5p | TGGCTCCCTTCTCTCCGTCTG | TGGCTCCCTTCTCTCCGTCTGCCTCCTGGCCGCGGGGCCCGGAGAGCTGGGAGCCAG |
| hsa-miR-30b-3p | CTGGGAGGTGGATGTTTACTTC | ACCAAGTTTCAGTTCATGTAAACATCCTACACTCAGCTGTAATACATGGATTGGCTGGGAGGTGGATGTTTACTTCAGCTGACTTGGA |
| hsa-miR-4687-3p | TGGCTGTTGGAGGGGGCAGGC | ACCTGAGGAGCCAGCCCTCCTCCCGCACCCAAACTTGGAGCACTTGACCTTTGGCTGTTGGAGGGGGCAGGCTCGCGGGT |
| hsa-miR-520b-5p | CCTCTACAGGGAAGCGCTTTC | TCTCAGGCTGTGTCCCTCTACAGGGAAGCGCTTTCTGTTGTCTGAAAGAAAGGAAAGTGCATCCTTTTAGAGTGTTACTGTTTGAGA |
| hsa-miR-4794 | TCTGGCTATCTCACGAGACTGT | TTTTAACATCTGGCTATCTCACGAGACTGTATGTCCTAACAGTGCTTGTAGTCTCATGAGATAGCCAGATGTTAAAA |
| hsa-miR-6727-3p | TCCTGCCACCTCCTCCGCAG | GGGTGCTCGGGGCAGGCGGCTGGGAGCGGCCCTCACATTGATGGCTCCTGCCACCTCCTCCGCAG |
| hsa-miR-516a-5p | TTCTCGAGGAAAGAAGCACTTTC | TCTCAGGCTGTGACCTTCTCGAGGAAAGAAGCACTTTCTGTTGTCTGAAAGAAAAGAAAGTGCTTCCTTTCAGAGGGTTACGGTTTGAGA |
| hsa-miR-29b-3p | TAGCACCATTTGAAATCAGTGTT | CTTCAGGAAGCTGGTTTCATATGGTGGTTTAGATTTAAATAGTGATTGTCTAGCACCATTTGAAATCAGTGTTCTTGGGGG |
| hsa-miR-4713-3p | TGGGATCCAGACAGTGGGAGAA | GTCCCCATTTTTCTCCCACTACCAGGCTCCCATAAGGGTCGAATGGGATCCAGACAGTGGGAGAAAAATGGGGAC |
| hsa-miR-4433b-5p | ATGTCCCACCCCCACTCCTGT | TGTGTTCCCTATCCTCCTTATGTCCCACCCCCACTCCTGTTTGAATATTTCACCAGAAACAGGAGTGGGGGGTGGGACGTAAGGAGGATGGGGGAAAGAACA |
| hsa-miR-6755-3p | TGTTGTCATGTTTTTTCCCTAG | TGTTTTAGGGTAGACACTGACAACGTTATGTGTGGTCTTTAACCTGTTGTCATGTTTTTTCCCTAG |
| hsa-miR-4659b-3p | TTTCTTCTTAGACATGGCAGCT | CTGTTGACGTTGCCATGTCTAAGAAGAAAATTTTTCTCCAAAGTTTTCTTCTTAGACATGGCAGCTTCAGCAG |
| hsa-miR-6513-5p | TTTGGGATTGACGCCACATGTCT | GCTTTGGGATTGACGCCACATGTCTCAGGTCCCCAGCTGAGTCAAGTGTCATCTGTCCCTAGGC |
| hsa-miR-6872-5p | TCTCGCATCAGGAGGCAAGG | GTGGGTCTCGCATCAGGAGGCAAGGCCAGGACCCGCTGACCCATGCCTCCTGCCGCGGTCAG |
| hsa-miR-431-5p | TGTCTTGCAGGCCGTCATGCA | TCCTGCTTGTCCTGCGAGGTGTCTTGCAGGCCGTCATGCAGGCCACACTGACGGTAACGTTGCAGGTCGTCTTGCAGGGCTTCTCGCAAGACGACATCCTCATCACCAACGACG |
| hsa-miR-7974 | AGGCTGTGATGCTCTCCTGAGCCC | GCTCGGCCCCCACAGCGAAACGGCCGCCTAAACCACCCAGGCCTTATGGCTTCATAGGCTGTGATGCTCTCCTGAGCCC |
| hsa-miR-6516-3p | ATCATGTATGATACTGCAAACA | TGGGTTTTGAATTTGCAGTAACAGGTGTGAGCATTCTAGCAGCAGTTTGATGATCATGTATGATACTGCAAACAGGACCTA |
| hsa-miR-6768-5p | CACACAGGAAAAGCGGGGCCCTG | CCAGGCACACAGGAAAAGCGGGGCCCTGGGTTCGGCTGCTACCCCAAAGGCCACATTCTCCTGTGCACACAG |
| hsa-miR-548ar-5p | AAAAGTAATTGCAGTTTTTGC | AAAAGTAATTGCAGTTTTTGCTGTTGAACGTAGTGGTAAAACTGCAGTTATTTTTGC |
| hsa-miR-200b-3p | TAATACTGCCTGGTAATGATGA | CCAGCTCGGGCAGCCGTGGCCATCTTACTGGGCAGCATTGGATGGAGTCAGGTCTCTAATACTGCCTGGTAATGATGACGGCGGAGCCCTGCACG |
| hsa-miR-1249-5p | AGGAGGGAGGAGATGGGCCAAGTT | GGGAGGAGGGAGGAGATGGGCCAAGTTCCCTCTGGCTGGAACGCCCTTCCCCCCCTTCTTCACCTG |
| hsa-miR-6499-5p | TCGGGCGCAAGAGCACTGCAGT | TCAGTCGGGCGCAAGAGCACTGCAGTTCTGTTGGGTGACAGCAGTGTTTGTTTTGCCCACAG |
| hsa-miR-1305 | TTTTCAACTCTAATGGGAGAGA | AAGATCCTGCTGTTTCTACCATTAGTTTTGAATGTTTATTGTAAAGATACTTTTCAACTCTAATGGGAGAGACAGCAGGATTCTCC |
| hsa-miR-937-3p | ATCCGCGCTCTGACTCTCTGCC | AGCACTGCCCCCGGTGAGTCAGGGTGGGGCTGGCCCCCTGCTTCGTGCCCATCCGCGCTCTGACTCTCTGCCCACCTGCAGGAGCT |
| hsa-miR-432-3p | CTGGATGGCTCCTCCATGTCT | TGACTCCTCCAGGTCTTGGAGTAGGTCATTGGGTGGATCCTCTATTTCCTTACGTGGGCCACTGGATGGCTCCTCCATGTCTTGGAGTAGATCA |
| hsa-miR-6870-3p | GCTCATCCCCATCTCCTTTCAG | CAAGGTGGGGGAGATGGGGGTTGAACTTCATTTCTCATGCTCATCCCCATCTCCTTTCAG |
| hsa-miR-6510-3p | CACCGACTCTGTCTCCTGCAG | AGCAGCAGGGGAGAGAGAGGAGTCCTCTAGACACCGACTCTGTCTCCTGCAGAT |
| hsa-miR-550b-3p | TCTTACTCCCTCAGGCACTG | AGAGACTTGTTGGAGATGTGCCTGAGGGAGTAAGACACTATCTTACAACAACAGGGCTCTTACTCCCTCAGGCACTGCACCAGCCAGCAAAGCATCA |
| hsa-miR-3180 | TGGGGCGGAGCTTCCGGAG | GCTCCGCCCCACGTCGCATGCGCCCCGGGAACGCGTGGGGCGGAGCTTCCGGAGGCCCCGCTCTGCTGCCGACCCTGTGGAGCGGAGGGTGAAGCCTCCGGATGCCAGTCCCTCATCGCTGGCCTGGTCGCGCTGTGGCGAAGGGGGCGGAGC |
| hsa-miR-519e-3p | AAGTGCCTCCTTTTAGAGTGTT | TCTCATGCAGTCATTCTCCAAAAGGGAGCACTTTCTGTTTGAAAGAAAACAAAGTGCCTCCTTTTAGAGTGTTACTGTTTGAGA |
| hsa-miR-421 | ATCAACAGACATTAATTGGGCGC | GCACATTGTAGGCCTCATTAAATGTTTGTTGAATGAAAAAATGAATCATCAACAGACATTAATTGGGCGCCTGCTCTGTGATCTC |
| hsa-miR-378g | ACTGGGCTTGGAGTCAGAAG | CACTGGGCTTGGAGTCAGAAGACCTGGCTCCAGCCCAGCTC |
| hsa-miR-151a-5p | TCGAGGAGCTCACAGTCTAGT | TTTCCTGCCCTCGAGGAGCTCACAGTCTAGTATGTCTCATCCCCTACTAGACTGAAGCTCCTTGAGGACAGGGATGGTCATACTCACCTC |
| hsa-miR-29a-5p | ACTGATTTCTTTTGGTGTTCAG | ATGACTGATTTCTTTTGGTGTTCAGAGTCAATATAATTTTCTAGCACCATCTGAAATCGGTTAT |
| hsa-miR-6894-3p | TTGCCTGCCCTCTTCCTCCAG | CAAGAAGGAGGATGGAGAGCTGGGCCAGACATGCTCTTGCCTGCCCTCTTCCTCCAG |
| hsa-miR-521 | AACGCACTTCCCTTTAGAGTGT | TCTCGGGCTGTGACTCTCCAAAGGGAAGAATTTTCTCTTGTCTAAAAGAAAAGAACGCACTTCCCTTTAGAGTGTTACCGTGTGAGA |
| hsa-miR-518c-5p | TCTCTGGAGGGAAGCACTTTCTG | GCGAGAAGATCTCATGCTGTGACTCTCTGGAGGGAAGCACTTTCTGTTGTCTGAAAGAAAACAAAGCGCTTCTCTTTAGAGTGTTACGGTTTGAGAAAAGC |
| hsa-miR-4788 | TTACGGACCAGCTAAGGGAGGC | AATGAAGGATTACGGACCAGCTAAGGGAGGCATTAGGATCCTTATTCTTGCCTCCCTTAGTTGGTCCCTAATCCTTCGTT |
| hsa-miR-590-5p | GAGCTTATTCATAAAAGTGCAG | TAGCCAGTCAGAAATGAGCTTATTCATAAAAGTGCAGTATGGTGAAGTCAATCTGTAATTTTATGTATAAGCTAGTCTCTGATTGAAACATGCAGCA |
| hsa-miR-4483 | GGGGTGGTCTGTTGTTG | AAAAAACAACATACTTAGTGCATACCCATATAATATTAGGGGTGGTCTGTTGTTGTTTTTCT |
| hsa-miR-3657 | TGTGTCCCATTATTGGTGATT | TGTGTCCCATAATTAAATAATGAAATCTGAAATCACCAATAATGGGACACTAATGTGATTAATGTTGTTGTGTCCCATTATTGGTGATTTCAGATTTCATATATGATTAAGGACATA |
| hsa-miR-148a-3p | TCAGTGCACTACAGAACTTTGT | GAGGCAAAGTTCTGAGACACTCCGACTCTGAGTATGATAGAAGTCAGTGCACTACAGAACTTTGTCTC |
| hsa-miR-3151-5p | GGTGGGGCAATGGGATCAGGT | GGGGTGATGGGTGGGGCAATGGGATCAGGTGCCTCAAAGGGCATCCCACCTGATCCCACAGCCCACCTGTCACCCC |
| hsa-miR-4482-3p | TTTCTATTTCTCAGTGGGGCTC | AGTGAGCAACCCAGTGGGCTATGGAAATGTGTGGAAGATGGCATTTCTATTTCTCAGTGGGGCTCTTACC |
| hsa-miR-412-5p | TGGTCGACCAGTTGGAAAGTAAT | CTGGGGTACGGGGATGGATGGTCGACCAGTTGGAAAGTAATTGTTTCTAATGTACTTCACCTGGTCCACTAGCCGTCCGTATCCGCTGCAG |
| hsa-miR-6851-3p | TGGCCCTTTGTACCCCTCCAG | CAGGGAGGAGGTGGTACTAGGGGCCAGCAACCTGATTACCCCTCTTTGGCCCTTTGTACCCCTCCAG |
| hsa-miR-6502-5p | AGCTCTAGAAAGATTGTTGACC | CAGAGTGGGAGCTCTAGAAAGATTGTTGACCAATCATCTTATTGACTAGACCATCTTTCTAGAGTATAACTATTTT |
| hsa-miR-570-3p | CGAAAACAGCAATTACCTTTGC | CTAGATAAGTTATTAGGTGGGTGCAAAGGTAATTGCAGTTTTTCCCATTATTTTAATTGCGAAAACAGCAATTACCTTTGCACCAACCTGATGGAGT |
| hsa-miR-2113 | ATTTGTGCTTGGCTCTGTCAC | TTTTCAAAGCAATGTGTGACAGGTACAGGGACAAATCCCGTTAATAAGTAAGAGGATTTGTGCTTGGCTCTGTCACATGCCACTTTGAAAA |
| hsa-miR-6736-3p | TCAGCTCCTCTCTACCCACAG | CTGAGCTGGGTGAGGGCATCTGTGGTTTGCTGGCTGCCTCAGCTCCTCTCTACCCACAG |
| hsa-miR-4757-5p | AGGCCTCTGTGACGTCACGGTGT | TTCCAGCCCGAGGCCTCTGTGACGTCACGGTGTCTGCGGGAGGAGACCATGACGTCACAGAGGCTTCGCGCTCTGAG |
| hsa-miR-3135a | TGCCTAGGCTGAGACTGCAGTG | TCACTTTGGTGCCTAGGCTGAGACTGCAGTGGTGCAATCTCAGTTCACTGCAGCCTTGACCTCCTGGGCTCAGGTGA |
| hsa-miR-324-3p | CCCACTGCCCCAGGTGCTGCTGG | CTGACTATGCCTCCCCGCATCCCCTAGGGCATTGGTGTAAAGCTGGAGACCCACTGCCCCAGGTGCTGCTGGGGGTTGTAGTC |
| hsa-miR-581 | TCTTGTGTTCTCTAGATCAGT | GTTATGTGAAGGTATTCTTGTGTTCTCTAGATCAGTGCTTTTAGAAAATTTGTGTGATCTAAAGAACACAAAGAATACCTACACAGAACCACCTGC |
| hsa-miR-520g-3p | ACAAAGTGCTTCCCTTTAGAGTGT | TCCCATGCTGTGACCCTCTAGAGGAAGCACTTTCTGTTTGTTGTCTGAGAAAAAACAAAGTGCTTCCCTTTAGAGTGTTACCGTTTGGGA |
| hsa-miR-506-3p | TAAGGCACCCTTCTGAGTAGA | GCCACCACCATCAGCCATACTATGTGTAGTGCCTTATTCAGGAAGGTGTTACTTAATAGATTAATATTTGTAAGGCACCCTTCTGAGTAGAGTAATGTGCAACATGGACAACATTTGTGGTGGC |
| hsa-miR-6811-5p | ATGCAGGCCTGTGTACAGCACT | TATGCAGGCCTGTGTACAGCACTCAGGCAGTGCCATGAGCCTGTGCTTGTCCCTGCAG |
| hsa-miR-1273h-3p | CTGCAGACTCGACCTCCCAGGC | TACTTGGGTGACTAAGGCAGGATTGCTTGAGCCTGGGAGGTCAAGGCTGCAGTGTCGTGGTCACAGCTTGCTGCAGACTCGACCTCCCAGGCTTAAGCAATCCTCCTGCTCGAGTG |
| hsa-miR-31-3p | TGCTATGCCAACATATTGCCAT | GGAGAGGAGGCAAGATGCTGGCATAGCTGTTGAACTGGGAACCTGCTATGCCAACATATTGCCATCTTTCC |
| hsa-miR-676-5p | TCTTCAACCTCAGGACTTGCA | GCATGACTCTTCAACCTCAGGACTTGCAGAATTAATGGAATGCTGTCCTAAGGTTGTTGAGTTGTGC |
| hsa-miR-2115-3p | CATCAGAATTCATGGAGGCTAG | ACTGTCATCCCACTGCTTCCAGCTTCCATGACTCCTGATGGAGGAATCACATGAATTCATCAGAATTCATGGAGGCTAGAAGCAGTATGAGGATCATTTA |
| hsa-miR-7152-5p | TTTCCTGTCCTCCAACCAGACC | TTTCCTGTCCTCCAACCAGACCATGCCACATCCGTCTGGTCCTGGACAGGAGGC |
| hsa-miR-7156-5p | TTGTTCTCAAACTGGCTGTCAGA | TTGTTCTCAAACTGGCTGTCAGAGTGTGCATGGCAGGCTGCAGCCACTTGGGGAACTGGT |
| hsa-miR-6802-3p | TTCACCCCTCTCACCTAAGCAG | GAGGGCTAGGTGGGGGGCTTGAAGCCCCGAGATGCCTCACGTCTTCACCCCTCTCACCTAAGCAG |
| hsa-miR-6780a-5p | TTGGGAGGGAAGACAGCTGGAGA | GACACTTGGGAGGGAAGACAGCTGGAGAGTATGGTCACAGCAGCATCCTCCTCTGTTTTCTTTCCTAG |
| hsa-miR-484 | TCAGGCTCAGTCCCCTCCCGAT | AGCCTCGTCAGGCTCAGTCCCCTCCCGATAAACCCCTAAATAGGGACTTTCCCGGGGGGTGACCCTGGCTTTTTTGGCG |
| hsa-miR-6770-5p | TGAGAAGGCACAGCTTGCACGTGA | TATCCTGAGAAGGCACAGCTTGCACGTGACCTCCTGGGCCTGGCGGCTGTGTCTTCACAG |
| hsa-miR-382-3p | AATCATTCACGGACAACACTT | TACTTGAAGAGAAGTTGTTCGTGGTGGATTCGCTTTACTTATGACGAATCATTCACGGACAACACTTTTTTCAGTA |
| hsa-miR-4701-5p | TTGGCCACCACACCTACCCCTT | CCTTGGCCACCACACCTACCCCTTGTGAATGTCGGGCAATGGGTGATGGGTGTGGTGTCCACA |
| hsa-miR-6848-5p | TGGGGGCTGGGATGGGCCATGGT | GTCCCTGGGGGCTGGGATGGGCCATGGTGTGCTCTGATCCCCCTGTGGTCTCTTGGCCCCCAGGAACTCC |
| hsa-miR-6724-5p | CTGGGCCCGCGGCGGGCGTGGGG | CGCTGCGCTTCTGGGCCCGCGGCGGGCGTGGGGCTGCCCGGGCCGGTCGACCAGCGCGCCGTAGCTCCCGAGGCCCGAGCCGCGACCCGCGG |
| hsa-miR-124-3p | TAAGGCACGCGGTGAATGCCAA | AGGCCTCTCTCTCCGTGTTCACAGCGGACCTTGATTTAAATGTCCATACAATTAAGGCACGCGGTGAATGCCAAGAATGGGGCTG |
| hsa-miR-3923 | AACTAGTAATGTTGGATTAGGG | GGTAGAGTGAGCTCTAATCCAATATTACTAGCTTCTTTATAAGAAGAGGAAACTAGTAATGTTGGATTAGGGCTCACTCTACT |
| hsa-miR-4422 | AAAAGCATCAGGAAGTACCCA | AGTTCTTCTGCAGACAAAAGCATCAGGAAGTACCCACCATGTACCAGTGGGCCCTTCTTGATGCTCTTGATTGCAGAGGAGCC |
| hsa-miR-96-3p | AATCATGTGCAGTGCCAATATG | TGGCCGATTTTGGCACTAGCACATTTTTGCTTGTGTCTCTCCGCTCTGAGCAATCATGTGCAGTGCCAATATGGGAAA |
| hsa-miR-4676-5p | GAGCCAGTGGTGAGACAGTGA | TGAATGAAAGAGCCAGTGGTGAGACAGTGAGTTGATTACTTCTCACTGTTTCACCACTGGCTCTTTGGTTCA |
| hsa-miR-320d | AAAAGCTGGGTTGAGAGGA | AAAATGTTGGCCTTCTCGTCCCAGTTCTTCCCAAAGTTGAGAAAAGCTGGGTTGAGAGGATGAAAAGAAAAA |
| hsa-miR-526b-5p | CTCTTGAGGGAAGCACTTTCTGT | TCAGGCTGTGACCCTCTTGAGGGAAGCACTTTCTGTTGTCTGAAAGAAGAGAAAGTGCTTCCTTTTAGAGGCTTACTGTCTGA |
| hsa-miR-3926 | TGGCCAAAAAGCAGGCAGAGA | AAAATGGAGCTGGCCAAAAAGCAGGCAGAGACTTTAAAAGCGTCTCTGCCTGCTTTTTGGCCAGCTCCGTTTT |
| hsa-miR-95-3p | TTCAACGGGTATTTATTGAGCA | AACACAGTGGGCACTCAATAAATGTCTGTTGAATTGAAATGCGTTACATTCAACGGGTATTTATTGAGCACCCACTCTGTG |
| hsa-miR-4688 | TAGGGGCAGCAGAGGACCTGGG | GTCTACTCCCAGGGTGCCAAGCTGTTTCGTGTTCCCTCCCTAGGGGATCCCAGGTAGGGGCAGCAGAGGACCTGGGCCTGGAC |
| hsa-miR-222-3p | AGCTACATCTGGCTACTGGGT | GCTGCTGGAAGGTGTAGGTACCCTCAATGGCTCAGTAGCCAGTGTAGATCCTGTCTTTCGTAATCAGCAGCTACATCTGGCTACTGGGTCTCTGATGGCATCTTCTAGCT |
| hsa-miR-4796-5p | TGTCTATACTCTGTCACTTTAC | TAAATTTGTGTCTATACTCTGTCACTTTACTTTTGGCCTCAAGTCATTGCAGTAAAGTGGCAGAGTATAGACACAAATTTA |
| hsa-miR-1297 | TTCAAGTAATTCAGGTG | TGTTTATCTCTAGGGTTGATCTATTAGAATTACTTATCTGAGCCAAAGTAATTCAAGTAATTCAGGTGTAGTGAAAC |
| hsa-miR-4522 | TGACTCTGCCTGTAGGCCGGT | GCGGGCGTTGCCTGGGGGCCTCGCAGGGGGAGATCCAGCCCAGGCTGGTTCCGCTGACTCTGCCTGTAGGCCGGTGGCGTCTTCTGG |
| hsa-miR-34c-3p | AATCACTAACCACACGGCCAGG | AGTCTAGTTACTAGGCAGTGTAGTTAGCTGATTGCTAATAGTACCAATCACTAACCACACGGCCAGGTAAAAAGATT |
| hsa-miR-6784-3p | TCTCACCCCAACTCTGCCCCAG | TACAGGCCGGGGCTTTGGGTGAGGGACCCCCGGAGTCTGTCACGGTCTCACCCCAACTCTGCCCCAG |
| hsa-miR-767-3p | TCTGCTCATACCCCATGGTTTCT | GCTTTTATATTGTAGGTTTTTGCTCATGCACCATGGTTGTCTGAGCATGCAGCATGCTTGTCTGCTCATACCCCATGGTTTCTGAGCAGGAACCTTCATTGTCTACTGC |
| hsa-miR-6771-5p | CTCGGGAGGGCATGGGCCAGGC | GGTGCCTCGGGAGGGCATGGGCCAGGCCACATAATGAGCCAAACCCCTGTCTACCCGCAG |
| hsa-miR-6875-3p | ATTCTTCCTGCCCTGGCTCCAT | GAGTCTGAGGGACCCAGGACAGGAGAAGGCCTATGGTGATTTGCATTCTTCCTGCCCTGGCTCCATCCTCAG |
| hsa-miR-1253 | AGAGAAGAAGATCAGCCTGCA | AGCAGCAAGAGATAGAATCCAAAAGAGAAGAAGATCAGCCTGCAGATGTGGACTGCTAAATGCAGGCTGATCTTCTCCCCTTTGGGATTCTCTTATGAGAAGCCA |
| hsa-miR-6789-3p | CGGCGCCCGTGTCTCCTCCAG | CGAGGTAGGGGCGTCCCGGGCGCGCGGGCGGGTCCCAGGCTGGGCCCCTCGGAGGCCGGGTGCTCACTGCCCCGTCCCGGCGCCCGTGTCTCCTCCAG |
| hsa-miR-134-3p | CCTGTGGGCCACCTAGTCACCAA | CAGGGTGTGTGACTGGTTGACCAGAGGGGCATGCACTGTGTTCACCCTGTGGGCCACCTAGTCACCAACCCTC |
| hsa-miR-936 | ACAGTAGAGGGAGGAATCGCAG | TCAAGGCCACTGGGACAGTAGAGGGAGGAATCGCAGAAATCACTCCAGGAGCAACTGAGAGACCTTGCTTCTACTTTACCAGGTCCTGCTGGCCCAGA |
| hsa-miR-193a-3p | AACTGGCCTACAAAGTCCCAGT | CGAGGATGGGAGCTGAGGGCTGGGTCTTTGCGGGCGAGATGAGGGTGTCGGATCAACTGGCCTACAAAGTCCCAGTTCTCGGCCCCCG |
| hsa-miR-1185-5p | AGAGGATACCCTTTGTATGTT | TTTGGTACTTAAAGAGAGGATACCCTTTGTATGTTCACTTGATTAATGGCGAATATACAGGGGGAGACTCTCATTTGCGTATCAAA |
| hsa-miR-6847-5p | ACAGAGGACAGTGGAGTGTGAGC | GACCCACAGAGGACAGTGGAGTGTGAGCTGGAAGGAGTGGGCCTGGCTCATGTGTCTGTCCTCTTCCAG |
| hsa-miR-6833-3p | TTTCTCTCTCCACTTCCTCAG | AAACGGTGTGGAAGATGGGAGGAGAAAAATCCCTGTTAACTTTCTCTCTCCACTTCCTCAG |
| hsa-miR-1277-3p | TACGTAGATATATATGTATTTT | ACCTCCCAAATATATATATATATGTACGTATGTGTATATAAATGTATACGTAGATATATATGTATTTTTGGTGGGTTT |
| hsa-miR-4776-3p | CTTGCCATCCTGGTCCACTGCAT | CTATATGCAGTGGACCAGGATGGCAAGGGCTCTCCTGAAAGGACAGTAGAGCCCTTGCCATCCTGGTCCACTGCATATAG |
| hsa-miR-1538 | CGGCCCGGGCTGCTGCTGTTCCT | GGGAACAGCAGCAACATGGGCCTCGCTTCCTGCCGGCGCGGCCCGGGCTGCTGCTGTTCCT |
| hsa-miR-3116 | TGCCTGGAACATAGTAGGGACT | CTTTATTGAGTCCCTACTATGTTCCAGGCACTGGGTATCGTAGGTGCCTGGAACATAGTAGGGACTCAATAAAG |
| hsa-miR-30c-2-3p | CTGGGAGAAGGCTGTTTACTCT | AGATACTGTAAACATCCTACACTCTCAGCTGTGGAAAGTAAGAAAGCTGGGAGAAGGCTGTTTACTCTTTCT |
| hsa-miR-30e-3p | CTTTCAGTCGGATGTTTACAGC | GGGCAGTCTTTGCTACTGTAAACATCCTTGACTGGAAGCTGTAAGGTGTTCAGAGGAGCTTTCAGTCGGATGTTTACAGCGGCAGGCTGCCA |
| hsa-miR-149-5p | TCTGGCTCCGTGTCTTCACTCCC | GCCGGCGCCCGAGCTCTGGCTCCGTGTCTTCACTCCCGTGCTTGTCCGAGGAGGGAGGGAGGGACGGGGGCTGTGCTGGGGCAGCTGGA |
| hsa-miR-4646-5p | ACTGGGAAGAGGAGCTGAGGGA | ACTGGGAAGAGGAGCTGAGGGACATTGCGGAGAGGGTCTCACATTGTCCCTCTCCCTTCCCAG |
| hsa-miR-3198 | GTGGAGTCCTGGGGAATGGAGA | GACTGTGCTCTCACTGTTCACCCAGCACTAGCAGTACCAGACGGTTCTGTGGAGTCCTGGGGAATGGAGAGAGCACAGTC |
| hsa-miR-221-3p | AGCTACATTGTCTGCTGGGTTTC | TGAACATCCAGGTCTGGGGCATGAACCTGGCATACAATGTAGATTTCTGTGTTCGTTAGGCAACAGCTACATTGTCTGCTGGGTTTCAGGCTACCTGGAAACATGTTCTC |
| hsa-miR-3139 | TAGGAGCTCAACAGATGCCTGTT | GGCTCAGAGTAGGAGCTCAACAGATGCCTGTTGACTGAATAATAAACAGGTATCGCAGGAGCTTTTGTTATGTGCC |
| hsa-miR-520f-5p | CCTCTAAAGGGAAGCGCTTTCT | TCTCAGGCTGTGACCCTCTAAAGGGAAGCGCTTTCTGTGGTCAGAAAGAAAAGCAAGTGCTTCCTTTTAGAGGGTTACCGTTTGGGA |
| hsa-miR-4999-5p | TGCTGTATTGTCAGGTAGTGA | ATAGAAAATAAAACACATACTGCTGTATTGTCAGGTAGTGATAGGATTTATCACTACCTGACAATACAGTATGTGTTTGTTTTATATATTT |
| hsa-miR-20b-3p | ACTGTAGTATGGGCACTTCCAG | AGTACCAAAGTGCTCATAGTGCAGGTAGTTTTGGCATGACTCTACTGTAGTATGGGCACTTCCAGTACT |
| hsa-miR-5188 | AATCGGACCCATTTAAACCGGAG | GGGAGGCATGGAAATTTCTCTGGTTTCAATGGGTACGATTATTGTAAGCAGGATCCATTCAATAATCGGACCCATTTAAACCGGAGATTTTAAAAGACAGGAATAGAATCCCA |
| hsa-miR-652-3p | AATGGCGCCACTAGGGTTGTG | ACGAATGGCTATGCACTGCACAACCCTAGGAGAGGGTGCCATTCACATAGACTATAATTGAATGGCGCCACTAGGGTTGTGCAGTGCACAACCTACAC |
| hsa-miR-2115-5p | AGCTTCCATGACTCCTGATGGA | ACTGTCATCCCACTGCTTCCAGCTTCCATGACTCCTGATGGAGGAATCACATGAATTCATCAGAATTCATGGAGGCTAGAAGCAGTATGAGGATCATTTA |
| hsa-miR-5681b | AGGTATTGCCACCCTTTCTAGT | GAAGAGGTATTGCCACCCTTTCTAGTCTAATAGGGACTAGAAAGGGTGGCAATACTCTTC |
| hsa-miR-4662a-5p | TTAGCCAATTGTCCATCTTTAG | TCTATTTAGCCAATTGTCCATCTTTAGCTATTCTGAATGCCTAAAGATAGACAATTGGCTAAATAGA |
| hsa-miR-212-3p | TAACAGTCTCCAGTCACGGCC | CGGGGCACCCCGCCCGGACAGCGCGCCGGCACCTTGGCTCTAGACTGCTTACTGCCCGGGCCGCCCTCAGTAACAGTCTCCAGTCACGGCCACCGACGCCTGGCCCCGCC |
| hsa-miR-585-5p | CTAGCACACAGATACGCCCAGA | TGGGGTGTCTGTGCTATGGCAGCCCTAGCACACAGATACGCCCAGAGAAAGCCTGAACGTTGGGCGTATCTGTATGCTAGGGCTGCTGTAACAA |
| hsa-miR-10396b-5p | CGGCGGGGCTCGGAGCCGGG | CGGCGGGGCTCGGAGCCGGGCTTCGGCCGGGCCCCGGGCCCTCGACCGGAC |
| hsa-miR-1224-5p | GTGAGGACTCGGGAGGTGG | GTGAGGACTCGGGAGGTGGAGGGTGGTGCCGCCGGGGCCGGGCGCTGTTTCAGCTCGCTTCTCCCCCCACCTCCTCTCTCCTCAG |
| hsa-miR-4772-5p | TGATCAGGCAAAATTGCAGACT | GTGATTGCCTCTGATCAGGCAAAATTGCAGACTGTCTTCCCAAATAGCCTGCAACTTTGCCTGATCAGAGGCAGTCAC |
| hsa-miR-6764-3p | TCTCTGGTCTTTCCTTGACAG | CTGACTCCCAGGGTCTGGTCAGAGTTGCTGAGTGGGTTGATCTCTGGTCTTTCCTTGACAG |
| hsa-miR-3158-3p | AAGGGCTTCCTCTCTGCAGGAC | ATTCAGGCCGGTCCTGCAGAGAGGAAGCCCTTCTGCTTACAGGTATTGGAAGGGCTTCCTCTCTGCAGGACCGGCCTGAAT |
| hsa-miR-3913-5p | TTTGGGACTGATCTTGATGTCT | TTGTTTATAATAAACTGAAATATTTGGGACTGATCTTGATGTCTGCCAAAACCTTGGCAGACATCAAGATCAGTCCCAAATATTTCAGTTTATTATAGACAG |
| hsa-miR-6740-3p | TGTCTTCTCTCCTCCCAAACAG | GAAAGAGTTTGGGATGGAGAGAGGAGAAACTTGAGGTCTCTGGGAGTTGCTTAAACCAGTTGACCGTAACCTGGCCAGAGAATTCTGATAGTGTCTTCTCTCCTCCCAAACAG |
| hsa-miR-592 | TTGTGTCAATATGCGATGATGT | TATTATGCCATGACATTGTGTCAATATGCGATGATGTGTTGTGATGGCACAGCGTCATCACGTGGTGACGCAACATCATGACGTAAGACGTCACAAC |
| hsa-miR-6741-5p | GTGGGTGCTGGTGGGAGCCGTG | AATGGGTGGGTGCTGGTGGGAGCCGTGCCCTGGCCACTCATTCGGCTCTCTCCCTCACCCTAG |
| hsa-miR-9983-3p | TTTTTTGCTGGAACATTTCTGG | GGAAATGTTCTAGCCAAAAAAGTTTGCCAAGAACCATTGTGTCTTTTTTTTTGCTGGAACATTTCTGG |
| hsa-miR-6753-5p | CACCAGGGCAGAGCAGGGCTGA | CACCAGGGCAGAGCAGGGCTGATCATCTCACGTCAGAGAGAGGGGAAGGGGCTGCCCAGTGAGCCCCCACAGGGCTCTACATCTCCAGCTGGGCCTGGCTGGAGATCCCAGGGTCCCTGAAGGCCCCCGCCACCGTTCTGGTCTGTCTCTGCCCTGGCACCCAG |
| hsa-miR-520b-5p | CCTCTACAGGGAAGCGCTTTC | CCCTCTACAGGGAAGCGCTTTCTGTTGTCTGAAAGAAAAGAAAGTGCTTCCTTTTAGAGGG |
| hsa-miR-940 | AAGGCAGGGCCCCCGCTCCCC | GTGAGGTGTGGGCCCGGCCCCAGGAGCGGGGCCTGGGCAGCCCCGTGTGTTGAGGAAGGAAGGCAGGGCCCCCGCTCCCCGGGCCTGACCCCAC |
| hsa-miR-33b-3p | CAGTGCCTCGGCAGTGCAGCCC | GCGGGCGGCCCCGCGGTGCATTGCTGTTGCATTGCACGTGTGTGAGGCGGGTGCAGTGCCTCGGCAGTGCAGCCCGGAGCCGGCCCCTGGCACCAC |
| hsa-miR-4668-3p | GAAAATCCTTTTTGTTTTTCCAG | AGGGAAAAAAAAAAGGATTTGTCTTGTAGCCAGGATATTGTTTTAAAGAAAATCCTTTTTGTTTTTCCAG |
| hsa-miR-1251-5p | ACTCTAGCTGCCAAAGGCGCT | GTGGACTCTAGCTGCCAAAGGCGCTTCTCCTTCTGAACAGAGCGCTTTGCTCAGCCAGTGTAGACATGGC |
| hsa-miR-5703 | AGGAGAAGTCGGGAAGGT | TTGCCGTCCCCTTCCTCGTCTTTTCCCCTCAGGAGAAGTCGGGAAGGTGGCGGCGG |
| hsa-miR-146a-3p | CCTCTGAAATTCAGTTCTTCAG | CCGATGTGTATCCTCAGCTTTGAGAACTGAATTCCATGGGTTGTGTCAGTGTCAGACCTCTGAAATTCAGTTCTTCAGCTGGGATATCTCTGTCATCGT |
| hsa-miR-449c-5p | TAGGCAGTGTATTGCTAGCGGCTGT | GCTGGGATGTGTCAGGTAGGCAGTGTATTGCTAGCGGCTGTTAATGATTTTAACAGTTGCTAGTTGCACTCCTCTCTGTTGCATTCAGAAGC |
| hsa-miR-6800-3p | CACCTCTCCTGGCATCGCCCC | ACCTGTAGGTGACAGTCAGGGGCGGGGTGTGGTGGGGCTGGGGCTGGCCCCCTCCTCACACCTCTCCTGGCATCGCCCCCAG |
| hsa-miR-3913-3p | AGACATCAAGATCAGTCCCAAA | TTGTTTATAATAAACTGAAATATTTGGGACTGATCTTGATGTCTGCCAAAACCTTGGCAGACATCAAGATCAGTCCCAAATATTTCAGTTTATTATAGACAG |
| hsa-miR-6814-3p | ACTCGCATCCTTCCCTTGGCAG | TTTCCTCCCAAGGGTGAGATGCTGCCACCCAGCCCTGCAGAGCCCCTGACTCGCATCCTTCCCTTGGCAG |
| hsa-miR-1292-3p | TCGCGCCCCGGCTCCCGTTC | CCTGGGAACGGGTTCCGGCAGACGCTGAGGTTGCGTTGACGCTCGCGCCCCGGCTCCCGTTCCAGG |
| hsa-miR-6735-3p | AGGCCTGTGGCTCCTCCCTCAG | GCAGCCAGGGCAGAGGGCACAGGAATCTGAGGTGACTGGCACAGAAGACTCAGGCCTGTGGCTCCTCCCTCAG |
| hsa-miR-548av-3p | AAAACTGCAGTTACTTTTGC | AAAAGTACTTGCGGATTTGCCATCACCTTTACCTTTAATGGCAAAACTGCAGTTACTTTTGC |
| hsa-miR-5701 | TTATTGTCACGTTCTGATT | GATTGGACTTTATTGTCACGTTCTGATTGGTTAGCCTAAGACTTGTTCTGATCCAATCAGAACATGAAAATAACGTCCAATC |
| hsa-miR-450b-3p | TTGGGATCATTTTGCATCCATA | GCAGAATTATTTTTGCAATATGTTCCTGAATATGTAATATAAGTGTATTGGGATCATTTTGCATCCATAGTTTTGTAT |
| hsa-miR-4497 | CTCCGGGACGGCTGGGC | ACCTCCGGGACGGCTGGGCGCCGGCGGCCGGGAGATCCGCGCTTCCTGAATCCCGGCCGGCCCGCCCGGCGCCCGTCCGCCCGCGGGTC |
| hsa-miR-3187-5p | CCTGGGCAGCGTGTGGCTGAAGG | GCTGGCCCTGGGCAGCGTGTGGCTGAAGGTCACCATGTTCTCCTTGGCCATGGGGCTGCGCGGGGCCAGC |
| hsa-miR-6820-5p | TGCGGCAGAGCTGGGGTCA | CCTTCTGCGGCAGAGCTGGGGTCACCAGCCCTCATGTACTTGTGACTTCTCCCCTGCCACAG |
| hsa-miR-3121-3p | TAAATAGAGTAGGCAAAGGACA | AAATGGTTATGTCCTTTGCCTATTCTATTTAAGACACCCTGTACCTTAAATAGAGTAGGCAAAGGACAGAAACATTT |
| hsa-miR-452-5p | AACTGTTTGCAGAGGAAACTGA | GCTAAGCACTTACAACTGTTTGCAGAGGAAACTGAGACTTTGTAACTATGTCTCAGTCTCATCTGCAAAGAAGTAAGTGCTTTGC |
| hsa-miR-10397-5p | TCCTTGACCTGATGCTGTAGGG | TCCTTGACCTGATGCTGTAGGGCTTGGCAACTCTTGTGAAATTTCATAGATCTCGTCGCTTACTGGGA |
| hsa-miR-642a-5p | GTCCCTCTCCAAATGTGTCTTG | ATCTGAGTTGGGAGGGTCCCTCTCCAAATGTGTCTTGGGGTGGGGGATCAAGACACATTTGGAGAGGGAACCTCCCAACTCGGCCTCTGCCATCATT |
| hsa-miR-12125 | TCTCTCACCTGGCATAAGCAAT | CAGTTAATTCAGAGTTGCTTATGTCAGGTGAGAGGGGTAGACACCAATTCTCTCTCACCTGGCATAAGCAATTCACAATTAACTG |
| hsa-miR-6753-3p | TGGTCTGTCTCTGCCCTGGCAC | CACCAGGGCAGAGCAGGGCTGATCATCTCACGTCAGAGAGAGGGGAAGGGGCTGCCCAGTGAGCCCCCACAGGGCTCTACATCTCCAGCTGGGCCTGGCTGGAGATCCCAGGGTCCCTGAAGGCCCCCGCCACCGTTCTGGTCTGTCTCTGCCCTGGCACCCAG |
| hsa-miR-363-5p | CGGGTGGATCACGATGCAATTT | TGTTGTCGGGTGGATCACGATGCAATTTTGATGAGTATCATAGGAGAAAAATTGCACGGTATCCATCTGTAAACC |
| hsa-miR-7851-3p | TACCTGGGAGACTGAGGTTGGA | TGGCTCACTGCAGCCTCCCCGCCCCCTCAGGTGATCCTCCCACCTCATCCTCCCAAGTAGCTGGGAATACAGGTGTGTGCCACCATGCCTCTACAAGCTACCTGGGAGACTGAGGTTGGAAGATTGCTTGAGCCTAGGAGGTCGAGGCGACAGTGAGCCA |
| hsa-miR-6783-5p | TAGGGGAAAAGTCCTGATCCGG | CCTGTTAGGGGAAAAGTCCTGATCCGGGAACCCACAGCCCCGTTCCTGGGCTTCTCCTCTGTAG |
| hsa-miR-526a-5p | CTCTAGAGGGAAGCACTTTCTG | TCTCAGGCTGTCGTCCTCTAGAGGGAAGCACTTTCTGTTGTCTGAAAGAAAAGAAAGTGCTTCCTTTTAGAGGGTTACCGTTTGAGA |
| hsa-miR-4766-5p | TCTGAAAGAGCAGTTGGTGTT | CTGAAGCTCCTTCTGAAAGAGCAGTTGGTGTTTATTTTTTACTAAATAGCAATTGCTCTTTTGGAAGGAACTTGAG |
| hsa-miR-580-3p | TTGAGAATGATGAATCATTAGG | ATAAAATTTCCAATTGGAACCTAATGATTCATCAGACTCAGATATTTAAGTTAACAGTATTTGAGAATGATGAATCATTAGGTTCCGGTCAGAAATT |
| hsa-miR-4716-3p | AAGGGGGAAGGAAACATGGAGA | CATACTTTGTCTCCATGTTTCCTTCCCCCTTCTGTATACATGTATACAGGAGGAAGGGGGAAGGAAACATGGAGACAAAGTGTG |
| hsa-miR-25-5p | AGGCGGAGACTTGGGCAATTG | GGCCAGTGTTGAGAGGCGGAGACTTGGGCAATTGCTGGACGCTGCCCTGGGCATTGCACTTGTCTCGGTCTGACAGTGCCGGCC |
| hsa-miR-137-3p | TTATTGCTTAAGAATACGCGTAG | GGTCCTCTGACTCTCTTCGGTGACGGGTATTCTTGGGTGGATAATACGGATTACGTTGTTATTGCTTAAGAATACGCGTAGTCGAGGAGAGTACCAGCGGCA |
| hsa-miR-548b-3p | CAAGAACCTCAGTTGCTTTTGT | CAGACTATATATTTAGGTTGGCGCAAAAGTAATTGTGGTTTTGGCCTTTATTTTCAATGGCAAGAACCTCAGTTGCTTTTGTGCCAACCTAATACTT |
| hsa-miR-11401 | TCACGTCTGCGGCTGTCACG | CTCGCGGCTGTCACGTCTGCGGCTGTCACGTCTGCTAGGGGGTTGGGGGGTCCAGGAAGGGCAGCGAGT |
| hsa-miR-4651 | CGGGGTGGGTGAGGTCGGGC | CGGCGACGGCGGGGTGGGTGAGGTCGGGCCCCAAGACTCGGGGTTTGCCGGGCGCCTCAGTTCACCGCGGCCG |
| hsa-miR-4798-5p | TTCGGTATACTTTGTGAATTGG | AAGTACAACTTCGGTATACTTTGTGAATTGGCTTTTACAAAAGACCAACTCACGAAGTATACCGAAGTCATACTT |
| hsa-miR-148a-5p | AAAGTTCTGAGACACTCCGACT | GAGGCAAAGTTCTGAGACACTCCGACTCTGAGTATGATAGAAGTCAGTGCACTACAGAACTTTGTCTC |
| hsa-miR-4781-3p | AATGTTGGAATCCTCGCTAGAG | AGGTGCACGCTCTAGCGGGGATTCCAATATTGGGCCAATTCCCCCAATGTTGGAATCCTCGCTAGAGCGTGCACTT |
| hsa-miR-4716-5p | TCCATGTTTCCTTCCCCCTTCT | CATACTTTGTCTCCATGTTTCCTTCCCCCTTCTGTATACATGTATACAGGAGGAAGGGGGAAGGAAACATGGAGACAAAGTGTG |
| hsa-miR-99b-5p | CACCCGTAGAACCGACCTTGCG | GGCACCCACCCGTAGAACCGACCTTGCGGGGCCTTCGCCGCACACAAGCTCGTGTCTGTGGGTCCGTGTC |
| hsa-miR-548au-5p | AAAAGTAATTGCGGTTTTTGC | AAAAGTAATTGCGGTTTTTGCTATTGGTTTTAATGGCAGTTACTTTTGCACCAG |
| hsa-miR-494-3p | TGAAACATACACGGGAAACCTC | GATACTCGAAGGAGAGGTTGTCCGTGTTGTCTTCTCTTTATTTATGATGAAACATACACGGGAAACCTCTTTTTTAGTATC |
| hsa-miR-221-5p | ACCTGGCATACAATGTAGATTT | TGAACATCCAGGTCTGGGGCATGAACCTGGCATACAATGTAGATTTCTGTGTTCGTTAGGCAACAGCTACATTGTCTGCTGGGTTTCAGGCTACCTGGAAACATGTTCTC |
| hsa-miR-6780a-3p | CTCCTCTGTTTTCTTTCCTAG | GACACTTGGGAGGGAAGACAGCTGGAGAGTATGGTCACAGCAGCATCCTCCTCTGTTTTCTTTCCTAG |
| hsa-miR-548at-3p | CAAAACCGCAGTAACTTTTGT | AAAAGTTATTGCGGTTTTGGCTGCCAAAAGAAATGGCCAAAACCGCAGTAACTTTTGT |
| hsa-miR-3664-3p | TCTCAGGAGTAAAGACAGAGTT | CTGTAAACTTGAAGGTAGGGAACTCTGTCTTCACTCATGAGTACCTTCCAACACGAGCTCTCAGGAGTAAAGACAGAGTTCCCTACCTTCAATGTGGAT |
| hsa-let-7f-2-3p | CTATACAGTCTACTGTCTTTCC | TGTGGGATGAGGTAGTAGATTGTATAGTTTTAGGGTCATACCCCATCTTGGAGATAACTATACAGTCTACTGTCTTTCCCACG |
| hsa-miR-551a | GCGACCCACTCTTGGTTTCCA | GGGGACTGCCGGGTGACCCTGGAAATCCAGAGTGGGTGGGGCCAGTCTGACCGTTTCTAGGCGACCCACTCTTGGTTTCCAGGGTTGCCCTGGAAA |
| hsa-miR-4749-5p | TGCGGGGACAGGCCAGGGCATC | CCTGCGGGGACAGGCCAGGGCATCTAGGCTGTGCACAGTGACGCCCCTCCTGCCCCCACAG |
| hsa-miR-3167 | AGGATTTCAGAAATACTGGTGT | GGCTGTGGAGGCACCAGTATTTCTGAAATTCTTTTTTCTGAAATTCTTCAGGAAGGATTTCAGAAATACTGGTGTCCCGACAGCC |
| hsa-miR-1295b-5p | CACCCAGATCTGCGGCCTAAT | CACCCAGATCTGCGGCCTAATCACAGGCCACATTTCTGAATAGGCCACGGATCTGGGCAA |
| hsa-miR-891a-3p | AGTGGCACATGTTTGTTGTGAG | CCTTAATCCTTGCAACGAACCTGAGCCACTGATTCAGTAAAATACTCAGTGGCACATGTTTGTTGTGAGGGTCAAAAGA |
| hsa-miR-215-5p | ATGACCTATGAATTGACAGAC | ATCATTCAGAAATGGTATACAGGAAAATGACCTATGAATTGACAGACAATATAGCTGAGTTTGTCTGTCATTTCTTTAGGCCAATATTCTGTATGACTGTGCTACTTCAA |
| hsa-miR-1292-5p | TGGGAACGGGTTCCGGCAGACGCTG | CCTGGGAACGGGTTCCGGCAGACGCTGAGGTTGCGTTGACGCTCGCGCCCCGGCTCCCGTTCCAGG |
| hsa-miR-4670-3p | TGAAGTTACATCATGGTCGCTT | CTCTAGGAAGCGACCATGATGTAACTTCACAGACTCTCCAAAAGTCTGAAGTTACATCATGGTCGCTTCCTAGAG |
| hsa-miR-605-5p | TAAATCCCATGGTGCCTTCTCCT | GCCCTAGCTTGGTTCTAAATCCCATGGTGCCTTCTCCTTGGGAAAAACAGAGAAGGCACTATGAGATTTAGAATCAAGTTAGG |
| hsa-miR-6806-3p | TGAAGCTCTGACATTCCTGCAG | TGCTCTGTAGGCATGAGGCAGGGCCCAGGTTCCATGTGATGCTGAAGCTCTGACATTCCTGCAG |
| hsa-miR-6758-3p | ACTCATTCTCCTCTGTCCAG | TGGGCTAGAGAGGGGAAGGATGTGATGTGAGCAGATGGTTCTCACTCATTCTCCTCTGTCCAG |
| hsa-miR-5684 | AACTCTAGCCTGAGCAACAG | GCTGAACTCTAGCCTGAGCAACAGAGTGAGATGGTCTTGTTTTGTTGCCCAGGCTGGAGTCCAGT |
| hsa-miR-150-5p | TCTCCCAACCCTTGTACCAGTG | CTCCCCATGGCCCTGTCTCCCAACCCTTGTACCAGTGCTGGGCTCAGACCCTGGTACAGGCCTGGGGGACAGGGACCTGGGGAC |
| hsa-miR-361-3p | TCCCCCAGGTGTGATTCTGATTT | GGAGCTTATCAGAATCTCCAGGGGTACTTTATAATTTCAAAAAGTCCCCCAGGTGTGATTCTGATTTGCTTC |
| hsa-miR-4446-3p | CAGGGCTGGCAGTGACATGGGT | CTGGTCCATTTCCCTGCCATTCCCTTGGCTTCAATTTACTCCCAGGGCTGGCAGTGACATGGGTCAA |
| hsa-miR-628-5p | ATGCTGACATATTTACTAGAGG | ATAGCTGTTGTGTCACTTCCTCATGCTGACATATTTACTAGAGGGTAAAATTAATAACCTTCTAGTAAGAGTGGCAGTCGAAGGGAAGGGCTCAT |
| hsa-miR-100-3p | CAAGCTTGTATCTATAGGTATG | CCTGTTGCCACAAACCCGTAGATCCGAACTTGTGGTATTAGTCCGCACAAGCTTGTATCTATAGGTATGTGTCTGTTAGG |
| hsa-miR-3133 | TAAAGAACTCTTAAAACCCAAT | CAGAAATTGTAAAGAACTCTTAAAACCCAATAGTAAAAAGACAACCTGTTGAGTTTTAAGAGTTCTTTATATATTCTG |
| hsa-miR-6777-3p | TCCACTCTCCTGGCCCCCAG | TCAAGACGGGGAGTCAGGCAGTGGTGGAGATGGAGAGCCCTGAGCCTCCACTCTCCTGGCCCCCAG |
| hsa-miR-323a-5p | AGGTGGTCCGTGGCGCGTTCGC | TTGGTACTTGGAGAGAGGTGGTCCGTGGCGCGTTCGCTTTATTTATGGCGCACATTACACGGTCGACCTCTTTGCAGTATCTAATC |
| hsa-miR-345-5p | GCTGACTCCTAGTCCAGGGCTC | ACCCAAACCCTAGGTCTGCTGACTCCTAGTCCAGGGCTCGTGATGGCTGGTGGGCCCTGAACGAGGGGTCTGGAGGCCTGGGTTTGAATATCGACAGC |
| hsa-miR-877-5p | GTAGAGGAGATGGCGCAGGG | GTAGAGGAGATGGCGCAGGGGACACGGGCAAAGACTTGGGGGTTCCTGGGACCCTCAGACGTGTGTCCTCTTCTCCCTCCTCCCAG |
| hsa-miR-5008-5p | TGAGGCCCTTGGGGCACAGTGG | GGGCTGACCCCTAGGGTCAGGTGAGGCCCTTGGGGCACAGTGGTGCCATCTCCCCTGTGCTCCCAGGGCCTCGCCTGTCCCTTGAGGTCGGCCC |
| hsa-miR-27b-5p | AGAGCTTAGCTGATTGGTGAAC | ACCTCTCTAACAAGGTGCAGAGCTTAGCTGATTGGTGAACAGTGATTGGTTTCCGCTTTGTTCACAGTGGCTAAGTTCTGCACCTGAAGAGAAGGTG |
| hsa-miR-301b-3p | CAGTGCAATGATATTGTCAAAGC | GCCGCAGGTGCTCTGACGAGGTTGCACTACTGTGCTCTGAGAAGCAGTGCAATGATATTGTCAAAGCATCTGGGACCA |
| hsa-miR-5000-3p | TCAGGACACTTCTGAACTTGGA | CTGAAGAGTAGAGTGTGTGGTCCCAGTTCAGAAGTGTTCCTGAGTAACTTGTGCTTATAACTCAGGACACTTCTGAACTTGGACCATACAGGTCTCCCTGCTT |
| hsa-miR-1227-3p | CGTGCCACCCTTTTCCCCAG | GTGGGGCCAGGCGGTGGTGGGCACTGCTGGGGTGGGCACAGCAGCCATGCAGAGCGGGCATTTGACCCCGTGCCACCCTTTTCCCCAG |
| hsa-miR-659-3p | CTTGGTTCAGGGAGGGTCCCCA | TACCGACCCTCGATTTGGTTCAGGACCTTCCCTGAACCAAGGAAGAGTCACAGTCTCTTCCTTGGTTCAGGGAGGGTCCCCAACAATGTCCTCATGG |
| hsa-miR-6813-5p | CAGGGGCTGGGGTTTCAGGTTCT | GTAGGCAGGGGCTGGGGTTTCAGGTTCTCAGTCAGAACCTTGGCCCCTCTCCCCAG |
| hsa-miR-1256 | AGGCATTGACTTCTCACTAGCT | AGTCAGCCTGTTGAAGCTTTGAAGCTTTGATGCCAGGCATTGACTTCTCACTAGCTGTGAAAGTCCTAGCTAAAGAGAAGTCAATGCATGACATCTTGTTTCAATAGATGGCTGTTTCA |
| hsa-miR-105-5p | TCAAATGCTCAGACTCCTGTGGT | TGTGCATCGTGGTCAAATGCTCAGACTCCTGTGGTGGCTGCTCATGCACCACGGATGTTTGAGCATGTGCTACGGTGTCTA |
| hsa-miR-4755-3p | AGCCAGGCTCTGAAGGGAAAGT | AGATTCAGCTTTCCCTTCAGAGCCTGGCTTTGGCATCTATGAAAGCCAGGCTCTGAAGGGAAAGTTGAATCT |
| hsa-miR-199a-3p | ACAGTAGTCTGCACATTGGTTA | GCCAACCCAGTGTTCAGACTACCTGTTCAGGAGGCTCTCAATGTGTACAGTAGTCTGCACATTGGTTAGGC |
| hsa-miR-1224-3p | CCCCACCTCCTCTCTCCTCAG | GTGAGGACTCGGGAGGTGGAGGGTGGTGCCGCCGGGGCCGGGCGCTGTTTCAGCTCGCTTCTCCCCCCACCTCCTCTCTCCTCAG |
| hsa-miR-30d-3p | CTTTCAGTCAGATGTTTGCTGC | GTTGTTGTAAACATCCCCGACTGGAAGCTGTAAGACACAGCTAAGCTTTCAGTCAGATGTTTGCTGCTAC |
| hsa-miR-32-3p | CAATTTAGTGTGTGTGATATTT | GGAGATATTGCACATTACTAAGTTGCATGTTGTCACGGCCTCAATGCAATTTAGTGTGTGTGATATTTTC |
| hsa-miR-1307-5p | TCGACCGGACCTCGACCGGCT | CATCAAGACCCAGCTGAGTCACTGTCACTGCCTACCAATCTCGACCGGACCTCGACCGGCTCGTCTGTGTTGCCAATCGACTCGGCGTGGCGTCGGTCGTGGTAGATAGGCGGTCATGCATACGAATTTTCAGCTCTTGTTCTGGTGAC |
| hsa-miR-4500 | TGAGGTAGTAGTTTCTT | CAGGAGAGAAAGTACTGCCCAGAAGCTAAAGTGTAGATCAAACGCATAATGGCTGAGGTAGTAGTTTCTTGAACTT |
| hsa-miR-525-3p | GAAGGCGCTTCCCTTTAGAGCG | CTCAAGCTGTGACTCTCCAGAGGGATGCACTTTCTCTTATGTGAAAAAAAAGAAGGCGCTTCCCTTTAGAGCGTTACGGTTTGGG |
| hsa-miR-708-5p | AAGGAGCTTACAATCTAGCTGGG | AACTGCCCTCAAGGAGCTTACAATCTAGCTGGGGGTAAATGACTTGCACATGAACACAACTAGACTGTGAGCTTCTAGAGGGCAGGGA |
| hsa-miR-488-3p | TTGAAAGGCTATTTCTTGGTC | GAGAATCATCTCTCCCAGATAATGGCACTCTCAAACAAGTTTCCAAATTGTTTGAAAGGCTATTTCTTGGTCAGATGACTCTC |
| hsa-miR-2467-3p | AGCAGAGGCAGAGAGGCTCAGG | GGACAGGCACCTGAGGCTCTGTTAGCCTTGGCTCTGGGTCCTGCTCCTTAGAGCAGAGGCAGAGAGGCTCAGGGTCTGTCT |
| hsa-miR-4466 | GGGTGCGGGCCGGCGGGG | ACGCGGGTGCGGGCCGGCGGGGTAGAAGCCACCCGGCCCGGCCCGGCCCGGCGA |
| hsa-miR-6764-5p | TCCCAGGGTCTGGTCAGAGTTG | CTGACTCCCAGGGTCTGGTCAGAGTTGCTGAGTGGGTTGATCTCTGGTCTTTCCTTGACAG |
| hsa-miR-548y | AAAAGTAATCACTGTTTTTGCC | GCCTAAACTATTAGGTTGGTGCAAAAGTAATCACTGTTTTTGCCATTACTCTCAGTGGCAAAAACCGTGATTACTTTTGCACCAACCTAGTAACACCTTCACTGTGGGGG |
| hsa-miR-3928-5p | TGAAGCTCTAAGGTTCCGCCTGC | GCTGAAGCTCTAAGGTTCCGCCTGCGGGCAGGAAGCGGAGGAACCTTGGAGCTTCGGC |
| hsa-miR-3914 | AAGGAACCAGAAAATGAGAAGT | TGGACTTCAGATTTAACTTCTCATTTTCTGGTTCCTTCTAATGAGTATGCTTAACTTGGTAGAAGGAACCAGAAAATGAGAAGTTGAGTAGGAACTCTA |
| hsa-miR-34a-5p | TGGCAGTGTCTTAGCTGGTTGT | GGCCAGCTGTGAGTGTTTCTTTGGCAGTGTCTTAGCTGGTTGTTGTGAGCAATAGTAAGGAAGCAATCAGCAAGTATACTGCCCTAGAAGTGCTGCACGTTGTGGGGCCC |
| hsa-miR-335-5p | TCAAGAGCAATAACGAAAAATGT | TGTTTTGAGCGGGGGTCAAGAGCAATAACGAAAAATGTTTGTCATAAACCGTTTTTCATTATTGCTCCTGACCTCCTCTCATTTGCTATATTCA |
| hsa-miR-5588-3p | AAGTCCCACTAATGCCAGC | ACTGGCATTAGTGGGACTTTTTTTTTTTTTTTTTTTAATGTTAAAAGTCCCACTAATGCCAGC |
| hsa-miR-6848-3p | GTGGTCTCTTGGCCCCCAG | GTCCCTGGGGGCTGGGATGGGCCATGGTGTGCTCTGATCCCCCTGTGGTCTCTTGGCCCCCAGGAACTCC |
| hsa-miR-6769a-5p | AGGTGGGTATGGAGGAGCCCT | AGGCCAGGTGGGTATGGAGGAGCCCTCATATGGCAGTTGGCGAGGGCCCAGTGAGCCCCTCTCTGCTCTCCAG |
| hsa-miR-5091 | ACGGAGACGACAAGACTGTGCTG | GACTGTGGCGACGGAGACGACAAGACTGTGCTGGTCGCGGGTTGTGGGGTTTAGGTCACCGGCAGGGGTCTGGAGTCCCTGGAGGTTAGGGCT |
| hsa-miR-99b-3p | CAAGCTCGTGTCTGTGGGTCCG | GGCACCCACCCGTAGAACCGACCTTGCGGGGCCTTCGCCGCACACAAGCTCGTGTCTGTGGGTCCGTGTC |
| hsa-miR-26a-5p | TTCAAGTAATCCAGGATAGGCT | GTGGCCTCGTTCAAGTAATCCAGGATAGGCTGTGCAGGTCCCAATGGGCCTATTCTTGGTTACTTGCACGGGGACGC |
| hsa-miR-6819-5p | TTGGGGTGGAGGGCCAAGGAGC | GAGGGTTGGGGTGGAGGGCCAAGGAGCTGGGTGGGGTGCCAAGCCTCTGTCCCCACCCCAG |
| hsa-miR-6747-3p | TCCTGCCTTCCTCTGCACCAG | TTTGGAGGGGTGTGGAAAGAGGCAGAACATTCGTTCACTTTCCTGCCTTCCTCTGCACCAG |
| hsa-miR-190a-5p | TGATATGTTTGATATATTAGGT | TGCAGGCCTCTGTGTGATATGTTTGATATATTAGGTTGTTATTTAATCCAACTATATATCAAACATATTCCTACAGTGTCTTGCC |
| hsa-miR-6728-3p | TCTCTGCTCTGCTCTCCCCAG | CTAGATTGGGATGGTAGGACCAGAGGGGCTTACTGCCCTGTGGGGCTCTCTGGACCCAGTGCCATGCTTCTCTGCTCTGCTCTCCCCAG |
| hsa-miR-3165 | AGGTGGATGCAATGTGACCTCA | CCGGTGGCAAGGTGGATGCAATGTGACCTCAACTCTTGGTCCTCTGAGGTCACATTGTATCCACCTTACCACTGG |
| hsa-miR-548c-3p | CAAAAATCTCAATTACTTTTGC | CATTGGCATCTATTAGGTTGGTGCAAAAGTAATTGCGGTTTTTGCCATTACTTTCAGTAGCAAAAATCTCAATTACTTTTGCACCAACTTAATACTT |
| hsa-miR-6731-5p | TGGGAGAGCAGGGTATTGTGGA | ACAGGTGGGAGAGCAGGGTATTGTGGAAGCTCCAGGTGCCAACCACCTGCCTCTATTCCCCACTCTCCCCAG |
| hsa-miR-3130-3p | GCTGCACCGGAGACTGGGTAA | CTTGTCATGTCTTACCCAGTCTCCGGTGCAGCCTGTTGTCAAGGCTGCACCGGAGACTGGGTAAGACATGACAAG |
| hsa-miR-122b-5p | TTTAGTGTGATAATGGCGTTTGA | CAGTAGCTATTTAGTGTGATAATGGCGTTTGATAGTTTAGACACAAACACCATTGTCACACTCCACAGCTCTG |
| hsa-miR-4639-5p | TTGCTAAGTAGGCTGAGATTGA | TTGCTAAGTAGGCTGAGATTGATGTCAGGTTATCCCCAAGCATAACCTCACTCTCACCTTGCTTTGCAG |
| hsa-miR-1264 | CAAGTCTTATTTGAGCACCTGTT | AGGTCCTCAATAAGTATTTGTTGAAAGAATAAATAAACCAACAAGTCTTATTTGAGCACCTGTTATGTG |
| hsa-miR-3188 | AGAGGCTTTGTGCGGATACGGGG | GGCGCCTCCTGCTCTGCTGTGCCGCCAGGGCCTCCCCTAGCGCGCCTTCTGGAGAGGCTTTGTGCGGATACGGGGCTGGAGGCCT |
| hsa-let-7a-5p | TGAGGTAGTAGGTTGTATAGTT | TGGGATGAGGTAGTAGGTTGTATAGTTTTAGGGTCACACCCACCACTGGGAGATAACTATACAATCTACTGTCTTTCCTA |
| hsa-miR-7-5p | TGGAAGACTAGTGATTTTGTTGTT | TTGGATGTTGGCCTAGTTCTGTGTGGAAGACTAGTGATTTTGTTGTTTTTAGATAACTAAATCGACAACAAATCACAGTCTGCCATATGGCACAGGCCATGCCTCTACAG |
| hsa-miR-3136-5p | CTGACTGAATAGGTAGGGTCATT | AATATGAAACTGACTGAATAGGTAGGGTCATTTTTCTGTGACTGCACATGGCCCAACCTATTCAGTTAGTTCCATATT |
| hsa-miR-629-3p | GTTCTCCCAACGTAAGCCCAGC | TCCCTTTCCCAGGGGAGGGGCTGGGTTTACGTTGGGAGAACTTTTACGGTGAACCAGGAGGTTCTCCCAACGTAAGCCCAGCCCCTCCCCTCTGCCT |
| hsa-miR-518d-3p | CAAAGCGCTTCCCTTTGGAGC | TCCCATGCTGTGACCCTCTAGAGGGAAGCACTTTCTGTTGTCTGAAAGAAACCAAAGCGCTTCCCTTTGGAGCGTTACGGTTTGAGA |
| hsa-miR-6815-5p | TAGGTGGCGCCGGAGGAGTCATT | CACTGTAGGTGGCGCCGGAGGAGTCATTTCCCATCACTAATGGCTTCTCTTGCACACCCAG |
| hsa-miR-6746-5p | CCGGGAGAAGGAGGTGGCCTGG | CTTGCCCGGGAGAAGGAGGTGGCCTGGAGAGCTGCTGTCTCCAGCCGCCGCCTGTCTCCACAG |
| hsa-miR-6772-3p | TTGCTCCTGACTCTGTGCCCACA | AGGCCTGGGTGTAGGCTGGAGCTGAGGACTGAGGCTCACCTTGCTCCTGACTCTGTGCCCACAG |
| hsa-miR-130a-5p | GCTCTTTTCACATTGTGCTACT | TGCTGCTGGCCAGAGCTCTTTTCACATTGTGCTACTGTCTGCACCTGTCACTAGCAGTGCAATGTTAAAAGGGCATTGGCCGTGTAGTG |
| hsa-miR-874-3p | CTGCCCTGGCCCGAGGGACCGA | TTAGCCCTGCGGCCCCACGCACCAGGGTAAGAGAGACTCTCGCTTCCTGCCCTGGCCCGAGGGACCGACTGGCTGGGC |
| hsa-miR-331-3p | GCCCCTGGGCCTATCCTAGAA | GAGTTTGGTTTTGTTTGGGTTTGTTCTAGGTATGGTCCCAGGGATCCCAGATCAAACCAGGCCCCTGGGCCTATCCTAGAACCAACCTAAGCTC |
| hsa-miR-187-3p | TCGTGTCTTGTGTTGCAGCCGG | GGTCGGGCTCACCATGACACAGTGTGAGACCTCGGGCTACAACACAGGACCCGGGCGCTGCTCTGACCCCTCGTGTCTTGTGTTGCAGCCGGAGGGACGCAGGTCCGCA |
| hsa-miR-3150a-3p | CTGGGGAGATCCTCGAGGTTGG | GGGAAGCAGGCCAACCTCGACGATCTCCTCAGCACCTGAACGCCAAGGCTGGGGAGATCCTCGAGGTTGGCCTGCTTTCC |
| hsa-miR-523-3p | GAACGCGCTTCCCTATAGAGGGT | TCTCATGCTGTGACCCTCTAGAGGGAAGCGCTTTCTGTTGTCTGAAAGAAAAGAACGCGCTTCCCTATAGAGGGTTACCCTTTGAGA |
| hsa-miR-577 | TAGATAAAATATTGGTACCTG | TGGGGGAGTGAAGAGTAGATAAAATATTGGTACCTGATGAATCTGAGGCCAGGTTTCAATACTTTATCTGCTCTTCATTTCCCCATATCTACTTAC |
| hsa-miR-579-3p | TTCATTTGGTATAAACCGCGATT | CATATTAGGTTAATGCAAAAGTAATCGCGGTTTGTGCCAGATGACGATTTGAATTAATAAATTCATTTGGTATAAACCGCGATTATTTTTGCATCAAC |
| hsa-miR-382-5p | GAAGTTGTTCGTGGTGGATTCG | TACTTGAAGAGAAGTTGTTCGTGGTGGATTCGCTTTACTTATGACGAATCATTCACGGACAACACTTTTTTCAGTA |
| hsa-miR-4770 | TGAGATGACACTGTAGCT | GAGTTATGGGGTCATCTATCCTTCCCTTGGAAAATGATCTGAGATGACACTGTAGCTC |
| hsa-miR-218-1-3p | ATGGTTCCGTCAAGCACCATGG | GTGATAATGTAGCGAGATTTTCTGTTGTGCTTGATCTAACCATGTGGTTGCGAGGTATGAGTAAAACATGGTTCCGTCAAGCACCATGGAACGTCACGCAGCTTTCTACA |
| hsa-miR-6877-3p | CAGCCTCTGCCCTTGGCCTCC | AGTTCAGGGCCGAAGGGTGGAAGCTGCTGGTGCTCATCTCAGCCTCTGCCCTTGGCCTCCCCAG |
| hsa-miR-3115 | ATATGGGTTTACTAGTTGGT | TCTGAATATGGGTTTACTAGTTGGTGGTGAATTCATGAGTCGCCAACTATTAGGCCTTTATGTCCAGA |
| hsa-miR-4746-5p | CCGGTCCCAGGAGAACCTGCAGA | GTGTCTGTGCCGGTCCCAGGAGAACCTGCAGAGGCATCGGGTCAGCGGTGCTCCTGCGGGCCGACACTCAC |
| hsa-miR-5010-3p | TTTTGTGTCTCCCATTCCCCAG | GATCCAGGGAACCCTAGAGCAGGGGGATGGCAGAGCAAAATTCATGGCCTACAGCTGCCTCTTGCCAAACTGCACTGGATTTTGTGTCTCCCATTCCCCAGAGCTGTCTGAGGTGCTTTG |
| hsa-miR-376a-5p | GTAGATTCTCCTTCTATGAGTA | TAAAAGGTAGATTCTCCTTCTATGAGTACATTATTTATGATTAATCATAGAGGAAAATCCACGTTTTC |
| hsa-miR-188-5p | CATCCCTTGCATGGTGGAGGG | TGCTCCCTCTCTCACATCCCTTGCATGGTGGAGGGTGAGCTTTCTGAAAACCCCTCCCACATGCAGGGTTTGCAGGATGGCGAGCC |
| hsa-miR-7978 | TCTGGTGTATAGCGTTGCTCA | TCTGGTGTATAGCGTTGCTCAAGTCCTCTATTTCTTTGAACAACAGTATACACCAAATA |
| hsa-miR-4507 | CTGGGTTGGGCTGGGCTGGG | TCTGGGCTGAGCCGAGCTGGGTTAAGCCGAGCTGGGTTGGGCTGGGCTGGGT |
| hsa-miR-495-3p | AAACAAACATGGTGCACTTCTT | TGGTACCTGAAAAGAAGTTGCCCATGTTATTTTCGCTTTATATGTGACGAAACAAACATGGTGCACTTCTTTTTCGGTATCA |
| hsa-miR-4516 | GGGAGAAGGGTCGGGGC | AGGGAGAAGGGTCGGGGCAGGGAGGGCAGGGCAGGCTCTGGGGTGGGGGGTCTGTGAGTCAGCCACGGCTCTGCCCACGTCTCCCC |
| hsa-miR-1910-3p | GAGGCAGAAGCAGGATGACA | TGTCCCTTCAGCCAGTCCTGTGCCTGCCGCCTTTGTGCTGTCCTTGGAGGGAGGCAGAAGCAGGATGACAATGAGGGCAA |
| hsa-miR-152-5p | AGGTTCTGTGATACACTCCGACT | TGTCCCCCCCGGCCCAGGTTCTGTGATACACTCCGACTCGGGCTCTGGAGCAGTCAGTGCATGACAGAACTTGGGCCCGGAAGGACC |
| hsa-miR-409-3p | GAATGTTGCTCGGTGAACCCCT | TGGTACTCGGGGAGAGGTTACCCGAGCAACTTTGCATCTGGACGACGAATGTTGCTCGGTGAACCCCTTTTCGGTATCA |
| hsa-miR-204-5p | TTCCCTTTGTCATCCTATGCCT | GGCTACAGTCTTTCTTCATGTGACTCGTGGACTTCCCTTTGTCATCCTATGCCTGAGAATATATGAAGGAGGCTGGGAAGGCAAAGGGACGTTCAATTGTCATCACTGGC |
| hsa-miR-1249-3p | ACGCCCTTCCCCCCCTTCTTCA | GGGAGGAGGGAGGAGATGGGCCAAGTTCCCTCTGGCTGGAACGCCCTTCCCCCCCTTCTTCACCTG |
| hsa-miR-4787-3p | GATGCGCCGCCCACTGCCCCGCGC | CGGTCCAGACGTGGCGGGGGTGGCGGCGGCATCCCGGACGGCCTGTGAGGGATGCGCCGCCCACTGCCCCGCGCCGCCTGACCG |
| hsa-miR-6879-5p | CAGGGCAGGGAAGGTGGGAGAG | CAGAGCAGGGCAGGGAAGGTGGGAGAGGGGCCCAGCTGACCCTCCTGTCACCCGCTCCTTGCCCAG |
| hsa-miR-548az-3p | AAAAACTGCAATCACTTTTGC | AGATTGTATTAGGTTGGTGCAAAAGTGATTGTGGTTTTTGCTGTTACTTTTAATGGCAAAAACTGCAATCACTTTTGCACCAACCTAATAAATTT |
| hsa-miR-6878-3p | CTGGCCTCTTCTTTCTCCTAG | ATGAGAGGGAGAAAGCTAGAAGCTGAAGATTCTGAAAATCACTAACTGGCCTCTTCTTTCTCCTAG |
| hsa-miR-183-5p | TATGGCACTGGTAGAATTCACT | CCGCAGAGTGTGACTCCTGTTCTGTGTATGGCACTGGTAGAATTCACTGTGAACAGTCTCAGTCAGTGAATTACCGAAGGGCCATAAACAGAGCAGAGACAGATCCACGA |
| hsa-miR-544a | ATTCTGCATTTTTAGCAAGTTC | ATTTTCATCACCTAGGGATCTTGTTAAAAAGCAGATTCTGATTCAGGGACCAAGATTCTGCATTTTTAGCAAGTTCTCAAGTGATGCTAAT |
| hsa-miR-135b-3p | ATGTAGGGCTAAAAGCCATGGG | CACTCTGCTGTGGCCTATGGCTTTTCATTCCTATGTGATTGCTGTCCCAAACTCATGTAGGGCTAAAAGCCATGGGCTACAGTGAGGGGCGAGCTCC |
| hsa-miR-570-5p | AAAGGTAATTGCAGTTTTTCCC | GTATTAGGTTGGTGCAAAGGTAATTGCAGTTTTTCCCATTTAAAATATGGAAAAAAAAATCACAATTACTTTTGCATCAACCTAATAA |
| hsa-miR-501-3p | AATGCACCCGGGCAAGGATTCT | GCTCTTCCTCTCTAATCCTTTGTCCCTGGGTGAGAGTGCTTTCTGAATGCAATGCACCCGGGCAAGGATTCTGAGAGGGTGAGC |
| hsa-miR-4695-5p | CAGGAGGCAGTGGGCGAGCAGG | CCTGCAGGAGGCAGTGGGCGAGCAGGCGGGGCAGCCCAATGCCATGGGCCTGATCTCACCGCTGCCTCCTTCCC |
| hsa-miR-103b | TCATAGCCCTGTACAATGCTGCT | TCATAGCCCTGTACAATGCTGCTTGATCCATATGCAACAAGGCAGCACTGTAAAGAAGCCGA |
| hsa-miR-524-3p | GAAGGCGCTTCCCTTTGGAGT | TCTCATGCTGTGACCCTACAAAGGGAAGCACTTTCTCTTGTCCAAAGGAAAAGAAGGCGCTTCCCTTTGGAGTGTTACGGTTTGAGA |
| hsa-miR-214-3p | ACAGCAGGCACAGACAGGCAGT | GGCCTGGCTGGACAGAGTTGTCATGTGTCTGCCTGTCTACACTTGCTGTGCAGAACATCCGCTCACCTGTACAGCAGGCACAGACAGGCAGTCACATGACAACCCAGCCT |
| hsa-miR-1197 | TAGGACACATGGTCTACTTCT | ACTTCCTGGTATTTGAAGATGCGGTTGACCATGGTGTGTACGCTTTATTTGTGACGTAGGACACATGGTCTACTTCTTCTCAATATCA |
| hsa-miR-3141 | GAGGGCGGGTGGAGGAGGA | TCACCCGGTGAGGGCGGGTGGAGGAGGAGGGTCCCCACCATCAGCCTTCACTGGGACGGGA |
| hsa-miR-6885-3p | CTTTGCTTCCTGCTCCCCTAG | CCTGGAGGGGGGCACTGCGCAAGCAAAGCCAGGGACCCTGAGAGGCTTTGCTTCCTGCTCCCCTAG |
| hsa-miR-346 | TGTCTGCCCGCATGCCTGCCTCT | GGTCTCTGTGTTGGGCGTCTGTCTGCCCGCATGCCTGCCTCTCTGTTGCTCTGAAGGAGGCAGGGGCTGGGCCTGCAGCTGCCTGGGCAGAGCGG |
| hsa-miR-21-5p | TAGCTTATCAGACTGATGTTGA | TGTCGGGTAGCTTATCAGACTGATGTTGACTGTTGAATCTCATGGCAACACCAGTCGATGGGCTGTCTGACA |
| hsa-miR-197-3p | TTCACCACCTTCTCCACCCAGC | GGCTGTGCCGGGTAGAGAGGGCAGTGGGAGGTAAGAGCTCTTCACCCTTCACCACCTTCTCCACCCAGCATGGCC |
| hsa-miR-5582-3p | TAAAACTTTAAGTGTGCCTAGG | TAGGCACACTTAAAGTTATAGCTACATCAGTTATAACTATATCAGTTAAAACTTTAAGTGTGCCTAGG |
| hsa-miR-4786-5p | TGAGACCAGGACTGGATGCACC | GGGCATGGCCTGAGACCAGGACTGGATGCACCACTCTCCCTGTGATGAGGTGAAGCCAGCTCTGGTCTGGGCCATTTCAC |
| hsa-miR-4485-5p | ACCGCCTGCCCAGTGA | AGAGGCACCGCCTGCCCAGTGACATGCGTTTAACGGCCGCGGTACCCTAACTGTGCA |
| hsa-miR-205-3p | GATTTCAGTGGAGTGAAGTTC | AAAGATCCTCAGACAATCCATGTGCTTCTCTTGTCCTTCATTCCACCGGAGTCTGTCTCATACCCAACCAGATTTCAGTGGAGTGAAGTTCAGGAGGCATGGAGCTGACA |
| hsa-miR-513c-5p | TTCTCAAGGAGGTGTCGTTTAT | GCGTACAGTGCCTTTCTCAAGGAGGTGTCGTTTATGTGAACTAAAATATAAATTTCACCTTTCTGAGAAGAGTAATGTACAGCA |
| hsa-miR-145-5p | GTCCAGTTTTCCCAGGAATCCCT | CACCTTGTCCTCACGGTCCAGTTTTCCCAGGAATCCCTTAGATGCTAAGATGGGGATTCCTGGAAATACTGTTCTTGAGGTCATGGTT |
| hsa-miR-4780 | ACCCTTGAGCCTGATCCCTAGC | GGCCAGTGCCAGGGGGTCAGGCTCAAGGACCAGCCCAAAGGCCAGGCCTGACCCTTGAGCCTGATCCCTAGCACTGATCCC |
| hsa-miR-3935 | TGTAGATACGAGCACCAGCCAC | GGATGTGTTCCTGTCCCAGAAGGAGCTGATGGTTGTATCTATGAAGGTAAGCATTTTTGTAGATACGAGCACCAGCCACCCTAAGCAAAGGCAGAGAATGCTTA |
| hsa-miR-602 | GACACGGGCGACAGCTGCGGCCC | TTCTCACCCCCGCCTGACACGGGCGACAGCTGCGGCCCGCTGTGTTCACTCGGGCCGAGTGCGTCTCCTGTCAGGCAAGGGAGAGCAGAGCCCCCCTG |
| hsa-miR-2355-5p | ATCCCCAGATACAATGGACAA | CAGACGTGTCATCCCCAGATACAATGGACAATATGCTATTATAATCGTATGGCATTGTCCTTGCTGTTTGGAGATAATACTGCTGAC |
| hsa-miR-552-5p | GTTTAACCTTTTGCCTGTTGG | AACCATTCAAATATACCACAGTTTGTTTAACCTTTTGCCTGTTGGTTGAAGATGCCTTTCAACAGGTGACTGGTTAGACAAACTGTGGTATATACA |
| hsa-miR-10401-5p | CGTGTGGGAAGGCGTGGGGT | CGTGTGGGAAGGCGTGGGGTGCGGACCCCGGCCCGACCTCGCCGTCCCGCCCGCCG |
| hsa-miR-433-3p | ATCATGATGGGCTCCTCGGTGT | CCGGGGAGAAGTACGGTGAGCCTGTCATTATTCAGAGAGGCTAGATCCTCTGTGTTGAGAAGGATCATGATGGGCTCCTCGGTGTTCTCCAGG |
| hsa-miR-187-5p | GGCTACAACACAGGACCCGGGC | GGTCGGGCTCACCATGACACAGTGTGAGACCTCGGGCTACAACACAGGACCCGGGCGCTGCTCTGACCCCTCGTGTCTTGTGTTGCAGCCGGAGGGACGCAGGTCCGCA |
| hsa-miR-1293 | TGGGTGGTCTGGAGATTTGTGC | AGGTTGTTCTGGGTGGTCTGGAGATTTGTGCAGCTTGTACCTGCACAAATCTCCGGACCACTTAGTCTTTA |
| hsa-miR-4749-3p | CGCCCCTCCTGCCCCCACAG | CCTGCGGGGACAGGCCAGGGCATCTAGGCTGTGCACAGTGACGCCCCTCCTGCCCCCACAG |
| hsa-miR-302d-3p | TAAGTGCTTCCATGTTTGAGTGT | CCTCTACTTTAACATGGAGGCACTTGCTGTGACATGACAAAAATAAGTGCTTCCATGTTTGAGTGTGG |
| hsa-miR-210-3p | CTGTGCGTGTGACAGCGGCTGA | ACCCGGCAGTGCCTCCAGGCGCAGGGCAGCCCCTGCCCACCGCACACTGCGCTGCCCCAGACCCACTGTGCGTGTGACAGCGGCTGATCTGTGCCTGGGCAGCGCGACCC |
| hsa-miR-6762-5p | CGGGGCCATGGAGCAGCCTGTGT | AGAGCCGGGGCCATGGAGCAGCCTGTGTAGACGGGGACCTGCCCTGCATGGGCACCCCCTCACTGGCTGCTTCCCTTGGTCTCCAG |
| hsa-miR-3177-3p | TGCACGGCACTGGGGACACGT | CCACGTGCCATGTGTACACACGTGCCAGGCGCTGTCTTGAGACATTCGCGCAGTGCACGGCACTGGGGACACGTGGCACTGG |
| hsa-miR-4687-5p | CAGCCCTCCTCCCGCACCCAAA | ACCTGAGGAGCCAGCCCTCCTCCCGCACCCAAACTTGGAGCACTTGACCTTTGGCTGTTGGAGGGGGCAGGCTCGCGGGT |
| hsa-miR-2682-5p | CAGGCAGTGACTGTTCAGACGTC | ACCTTCCTGAAAGAGGTTGGGGCAGGCAGTGACTGTTCAGACGTCCAATCTCTTTGGGACGCCTCTTCAGCGCTGTCTTCCCTGCCTCTGCCTTTAGGACGAGTCTCAAA |
| hsa-miR-520f-3p | AAGTGCTTCCTTTTAGAGGGTT | TCTCAGGCTGTGACCCTCTAAAGGGAAGCGCTTTCTGTGGTCAGAAAGAAAAGCAAGTGCTTCCTTTTAGAGGGTTACCGTTTGGGA |
| hsa-miR-656-5p | AGGTTGCCTGTGAGGTGTTCA | CTGAAATAGGTTGCCTGTGAGGTGTTCACTTTCTATATGATGAATATTATACAGTCAACCTCTTTCCGATATCGAATC |
| hsa-miR-487a-3p | AATCATACAGGGACATCCAGTT | GGTACTTGAAGAGTGGTTATCCCTGCTGTGTTCGCTTAATTTATGACGAATCATACAGGGACATCCAGTTTTTCAGTATC |
| hsa-miR-30d-5p | TGTAAACATCCCCGACTGGAAG | GTTGTTGTAAACATCCCCGACTGGAAGCTGTAAGACACAGCTAAGCTTTCAGTCAGATGTTTGCTGCTAC |
| hsa-miR-548av-5p | AAAAGTACTTGCGGATTT | AAAAGTACTTGCGGATTTGCCATCACCTTTACCTTTAATGGCAAAACTGCAGTTACTTTTGC |
| hsa-miR-6827-3p | ACCGTCTCTTCTGTTCCCCAG | TCTGGTGGGAGCCATGAGGGTCTGTGCTGTCTCTGAGCACCGTCTCTTCTGTTCCCCAG |
| hsa-miR-526a-5p | CTCTAGAGGGAAGCACTTTCTG | TCCCATGCTGTGACCCTCTAGAGGGAAGCACTTTCTGTTGTCTGAAAGAAACCAAAGCGCTTCCCTTTGGAGCGTTACGGTTTGAGA |
| hsa-miR-7108-5p | GTGTGGCCGGCAGGCGGGTGG | GTGTGGCCGGCAGGCGGGTGGGCGGGGGCGGCCGGTGGGAACCCCGCCCCGCCCCGCGCCCGCACTCACCCGCCCGTCTCCCCACAG |
| hsa-miR-6887-5p | TGGGGGGACAGATGGAGAGGACA | GAGAATGGGGGGACAGATGGAGAGGACACAGGCTGGCACTGAGGTCCCCTCCACTTTCCTCCTAG |
| hsa-miR-3690 | ACCTGGACCCAGCGTAGACAAAG | CCCATCTCCACCTGGACCCAGCGTAGACAAAGAGGTGTTTCTACTCCATATCTACCTGGACCCAGTGTAGATGGG |
| hsa-miR-34b-5p | TAGGCAGTGTCATTAGCTGATTG | GTGCTCGGTTTGTAGGCAGTGTCATTAGCTGATTGTACTGTGGTGGTTACAATCACTAACTCCACTGCCATCAAAACAAGGCAC |
| hsa-miR-6786-3p | TGACGCCCCTTCTGATTCTGCCT | GCCGGGTGGGGCGGGGCGGCCTCAGGAGGGGCCCAGCTCCCCTGGATGTGCTGCGGTGGGGCCGGAGGGGCGTCACGTGCACCCAAGTGACGCCCCTTCTGATTCTGCCTCAG |
| hsa-miR-518a-3p | GAAAGCGCTTCCCTTTGCTGGA | TCTCAAGCTGTGACTGCAAAGGGAAGCCCTTTCTGTTGTCTGAAAGAAGAGAAAGCGCTTCCCTTTGCTGGATTACGGTTTGAGA |
| hsa-miR-17-5p | CAAAGTGCTTACAGTGCAGGTAG | GTCAGAATAATGTCAAAGTGCTTACAGTGCAGGTAGTGATATGTGCATCTACTGCAGTGAAGGCACTTGTAGCATTATGGTGAC |
| hsa-miR-4524b-3p | GAGACAGGTTCATGCTGCTA | TAGCTGGGTGGATGTGTTCTTTTGAAGGATAGCAGCATAAGCCTGTCTCAAAATAATTCTGCAGTGAGACAGGTTCATGCTGCTATCGTTCCAAAGAGGAAGGGTAATCACTGTC |
| hsa-miR-6895-3p | TGTCTCTCGCCCTTGGCCTTAG | CAGCTCAGGGCCAGGCACAGAGTAAGCATCAATAGCATTGGCAAGTTGAACTGAGCTGTCTCTCGCCCTTGGCCTTAG |
| hsa-miR-6873-5p | CAGAGGGAATACAGAGGGCAAT | CCCAGCAGAGGGAATACAGAGGGCAATCAGGACTGGGTCATTCTCTCTGTCTTTCTCTCTCAG |
| hsa-miR-4674 | CTGGGCTCGGGACGCGCGGCT | CCCAGGCGCCCGCTCCCGACCCACGCCGCGCCGCCGGGTCCCTCCTCCCCGGAGAGGCTGGGCTCGGGACGCGCGGCTCAGCTCGGG |
| hsa-miR-3200-5p | AATCTGAGAAGGCGCACAAGGT | GGTGGTCGAGGGAATCTGAGAAGGCGCACAAGGTTTGTGTCCAATACAGTCCACACCTTGCGCTACTCAGGTCTGCTCGTGCCCT |
| hsa-miR-4730 | CTGGCGGAGCCCATTCCATGCCA | CGCAGGCCTCTGGCGGAGCCCATTCCATGCCAGATGCTGAGCGATGGCTGGTGTGTGCTGCTCCACAGGCCTGGTG |
| hsa-miR-6876-5p | CAGGAAGGAGACAGGCAGTTCA | AGTTGCAGGAAGGAGACAGGCAGTTCAGGAGGTGGCACTGCTGTGTGTGAGCTGTCTGTGTTTTCCTTCTCAG |
| hsa-miR-302b-3p | TAAGTGCTTCCATGTTTTAGTAG | GCTCCCTTCAACTTTAACATGGAAGTGCTTTCTGTGACTTTAAAAGTAAGTGCTTCCATGTTTTAGTAGGAGT |
| hsa-miR-92b-3p | TATTGCACTCGTCCCGGCCTCC | CGGGCCCCGGGCGGGCGGGAGGGACGGGACGCGGTGCAGTGTTGTTTTTTCCCCCGCCAATATTGCACTCGTCCCGGCCTCCGGCCCCCCCGGCCC |
| hsa-miR-3140-5p | ACCTGAATTACCAAAAGCTTT | CCTCTTGAGGTACCTGAATTACCAAAAGCTTTATGTATTCTGAAGTTATTGAAAATAAGAGCTTTTGGGAATTCAGGTAGTTCAGGAGTG |
| hsa-miR-3680-5p | GACTCACTCACAGGATTGTGCA | AAATTTAAGGAGGGACTCACTCACAGGATTGTGCAAATGCAAAGTTGGCTTTTGCATGACCCTGGGAGTAGGTGCCTCCTTAAATTT |
| hsa-miR-526a-3p | GAAAGCGCTTCCTTTTAGAGGA | CTCAGGCTGTGACCCTCTAGAGGGAAGCACTTTCTGTTGCTTGAAAGAAGAGAAAGCGCTTCCTTTTAGAGGATTACTCTTTGAG |
| hsa-miR-3187-3p | TTGGCCATGGGGCTGCGCGG | GCTGGCCCTGGGCAGCGTGTGGCTGAAGGTCACCATGTTCTCCTTGGCCATGGGGCTGCGCGGGGCCAGC |
| hsa-miR-758-5p | GATGGTTGACCAGAGAGCACAC | GCCTGGATACATGAGATGGTTGACCAGAGAGCACACGCTTTATTTGTGCCGTTTGTGACCTGGTCCACTAACCCTCAGTATCTAATGC |
| hsa-miR-5089-3p | ATGCTACTCGGAAATCCCACTGA | AAGGACTTCAGTGGGATTTCTGAGTAGCATCCTTGGAATCTGCACTCAAGGGATGCTACTCGGAAATCCCACTGAAGTCCTTTT |
| hsa-miR-5682 | GTAGCACCTTGCAGGATAAGGT | GGCCCATGGGTCTTATCCTGCAAGGTGCTGCAGAGACGAGGCCTGTAGCACCTTGCAGGATAAGGTCTACTGGGCC |
| hsa-miR-377-5p | AGAGGTTGCCCTTGGTGAATTC | TTGAGCAGAGGTTGCCCTTGGTGAATTCGCTTTATTTATGTTGAATCACACAAAGGCAACTTTTGTTTG |
| hsa-miR-4695-3p | TGATCTCACCGCTGCCTCCTTC | CCTGCAGGAGGCAGTGGGCGAGCAGGCGGGGCAGCCCAATGCCATGGGCCTGATCTCACCGCTGCCTCCTTCCC |
| hsa-miR-202-3p | AGAGGTATAGGGCATGGGAA | CGCCTCAGAGCCGCCCGCCGTTCCTTTTTCCTATGCATATACTTCTTTGAGGATCTGGCCTAAAGAGGTATAGGGCATGGGAAAACGGGGCGGTCGGGTCCTCCCCAGCG |
| hsa-miR-5196-3p | TCATCCTCGTCTCCCTCCCAG | TCTGAGGAGACCTGGGCTGTCAGAGGCCAGGGAAGGGGACGAGGGTTGGGGAACAGGTGGTTAGCACTTCATCCTCGTCTCCCTCCCAGGTTAGAAGGGCCCCCCTCTCTGAAGG |
| hsa-miR-2114-3p | CGAGCCTCAAGCAAGGGACTT | CCTCCATGCTCCTAGTCCCTTCCTTGAAGCGGTCGGATAATCACATGACGAGCCTCAAGCAAGGGACTTCAAGCTGGTGG |
| hsa-miR-5692a | CAAATAATACCACAGTGGGTGT | GACAGTACAAATAATACCACAGTGGGTGTACCTCATGTGTGTACACCCTGTGATATTATTTGTAATATC |
| hsa-miR-615-5p | GGGGGTCCCCGGTGCTCGGATC | CTCGGGAGGGGCGGGAGGGGGGTCCCCGGTGCTCGGATCTCGAGGGTGCTTATTGTTCGGTCCGAGCCTGGGTCTCCCTCTTCCCCCCAACCCCCC |
| hsa-miR-3677-3p | CTCGTGGGCTCTGGCCACGGCC | GGCAGTGGCCAGAGCCCTGCAGTGCTGGGCATGGGCTTCTCGTGGGCTCTGGCCACGGCC |
| hsa-miR-138-1-3p | GCTACTTCACAACACCAGGGCC | CCCTGGCATGGTGTGGTGGGGCAGCTGGTGTTGTGAATCAGGCCGTTGCCAATCAGAGAACGGCTACTTCACAACACCAGGGCCACACCACACTACAGG |
| hsa-miR-4641 | TGCCCATGCCATACTTTTGCCTCA | GGGGGGCAGGGGGCAGAGGGCATCAGAGGACAGCCGCCTGGTGCCCATGCCATACTTTTGCCTCAG |
| hsa-miR-370-5p | CAGGTCACGTCTCTGCAGTTAC | AGACAGAGAAGCCAGGTCACGTCTCTGCAGTTACACAGCTCACGAGTGCCTGCTGGGGTGGAACCTGGTCTGTCT |
| hsa-miR-4732-5p | TGTAGAGCAGGGAGCAGGAAGCT | GAGGGAGCTGTAGAGCAGGGAGCAGGAAGCTGTGTGTGTCCAGCCCTGACCTGTCCTGTTCTGCCCCCAGCCCCTC |
| hsa-miR-6760-3p | ACACTGTCCCCTTCTCCCCAG | CAGTGCAGGGAGAAGGTGGAAGTGCAGAGTGGGCTCACCTCTCGCCCACACTGTCCCCTTCTCCCCAG |
| hsa-miR-1237-5p | CGGGGGCGGGGCCGAAGCGCG | GTGGGAGGGCCCAGGCGCGGGCAGGGGTGGGGGTGGCAGAGCGCTGTCCCGGGGGCGGGGCCGAAGCGCGGCGACCGTAACTCCTTCTGCTCCGTCCCCCAG |
| hsa-miR-4421 | ACCTGTCTGTGGAAAGGAGCTA | CTGGGTCTCCTTTCTGCTGAGAGTTGAACACTTGTTGGGACAACCTGTCTGTGGAAAGGAGCTACCTAC |
| hsa-miR-3195 | CGCGCCGGGCCCGGGTT | CCGCAGCCGCCGCGCCGGGCCCGGGTTGGCCGCTGACCCCCGCGGGGCCCCCGGCGGCCGGGGCGGGGGCGGGGGCTGCCCCGG |
| hsa-miR-193a-5p | TGGGTCTTTGCGGGCGAGATGA | CGAGGATGGGAGCTGAGGGCTGGGTCTTTGCGGGCGAGATGAGGGTGTCGGATCAACTGGCCTACAAAGTCCCAGTTCTCGGCCCCCG |
| hsa-miR-2278 | GAGAGCAGTGTGTGTTGCCTGG | GTGCTGCAGGTGTTGGAGAGCAGTGTGTGTTGCCTGGGGACTGTGTGGACTGGTATCACCCAGACAGCTTGCACTGACTCCAGACCCTGCCGTCAT |
| hsa-miR-892c-5p | TATTCAGAAAGGTGCCAGTCA | GGCAGTGCCCTATTCAGAAAGGTGCCAGTCACTTAGATTACATGTCACTGTTTCCTTTCTGAGTGGAGTAGGGCTTA |
| hsa-miR-1179 | AAGCATTCTTTCATTGGTTGG | GGCTGGAAAGGAAGAAGCATTCTTTCATTGGTTGGTGTGTATTGCCTTGTCAACCAATAAGAGGATGCCATTTATCCTTTTCTGACTAGCT |
| hsa-miR-6759-3p | TGACCTTTGCCTCTCCCCTCAG | TATTGTTGTGGGTGGGCAGAAGTCTGTTTTCTTCATGGTTTTCTGACCTTTGCCTCTCCCCTCAG |
| hsa-miR-19b-1-5p | AGTTTTGCAGGTTTGCATCCAGC | CACTGTTCTATGGTTAGTTTTGCAGGTTTGCATCCAGCTGTGTGATATTCTGCTGTGCAAATCCATGCAAAACTGACTGTGGTAGTG |
| hsa-miR-500a-5p | TAATCCTTGCTACCTGGGTGAGA | GCTCCCCCTCTCTAATCCTTGCTACCTGGGTGAGAGTGCTGTCTGAATGCAATGCACCTGGGCAAGGATTCTGAGAGCGAGAGC |
| hsa-miR-744-5p | TGCGGGGCTAGGGCTAACAGCA | TTGGGCAAGGTGCGGGGCTAGGGCTAACAGCAGTCTTACTGAAGGTTTCCTGGAAACCACGCACATGCTGTTGCCACTAACCTCAACCTTACTCGGTC |
| hsa-miR-26a-2-3p | CCTATTCTTGATTACTTGTTTC | GGCTGTGGCTGGATTCAAGTAATCCAGGATAGGCTGTTTCCATCTGTGAGGCCTATTCTTGATTACTTGTTTCTGGAGGCAGCT |
| hsa-miR-3605-3p | CCTCCGTGTTACCTGTCCTCTAG | ACTTTATACGTGTAATTGTGATGAGGATGGATAGCAAGGAAGCCGCTCCCACCTGACCCTCACGGCCTCCGTGTTACCTGTCCTCTAGGTGGGACGCTCG |
| hsa-miR-34a-3p | CAATCAGCAAGTATACTGCCCT | GGCCAGCTGTGAGTGTTTCTTTGGCAGTGTCTTAGCTGGTTGTTGTGAGCAATAGTAAGGAAGCAATCAGCAAGTATACTGCCCTAGAAGTGCTGCACGTTGTGGGGCCC |
| hsa-miR-4677-3p | TCTGTGAGACCAAAGAACTACT | GCAAAGCAGCAATTGTTCTTTGGTCTTTCAGCCATGACCTGACCTTCTGTCTGTGAGACCAAAGAACTACTTTGCTTGGC |
| hsa-miR-758-3p | TTTGTGACCTGGTCCACTAACC | GCCTGGATACATGAGATGGTTGACCAGAGAGCACACGCTTTATTTGTGCCGTTTGTGACCTGGTCCACTAACCCTCAGTATCTAATGC |
| hsa-miR-6732-5p | TAGGGGGTGGCAGGCTGGCC | AGGCCTAGGGGGTGGCAGGCTGGCCATCAGTGTGGGCTAACCCTGTCCTCTCCCTCCCAG |
| hsa-miR-598-5p | GCGGTGATCCCGATGGTGTGAGC | GCTTGATGATGCTGCTGATGCTGGCGGTGATCCCGATGGTGTGAGCTGGAAATGGGGTGCTACGTCATCGTTGTCATCGTCATCATCATCATCCGAG |
| hsa-miR-548ar-3p | TAAAACTGCAGTTATTTTTGC | AAAAGTAATTGCAGTTTTTGCTGTTGAACGTAGTGGTAAAACTGCAGTTATTTTTGC |
| hsa-miR-4690-3p | GCAGCCCAGCTGAGGCCTCTG | GAGCAGGCGAGGCTGGGCTGAACCCGTGGGTGAGGAGTGCAGCCCAGCTGAGGCCTCTGC |
| hsa-miR-202-5p | TTCCTATGCATATACTTCTTTG | CGCCTCAGAGCCGCCCGCCGTTCCTTTTTCCTATGCATATACTTCTTTGAGGATCTGGCCTAAAGAGGTATAGGGCATGGGAAAACGGGGCGGTCGGGTCCTCCCCAGCG |
| hsa-miR-378d | ACTGGACTTGGAGTCAGAAA | GAATGGTTACAAGGAGAGAACACTGGACTTGGAGTCAGAAAACTTTCATCCAAGTCATTCCCTGCTCTAAGTCCCATTTCTGTTCCATGAGATTGTTT |
| hsa-miR-6765-5p | GTGAGGCGGGGCCAGGAGGGTGTGT | GTGAGGCGGGGCCAGGAGGGTGTGTGGCGTGGGTGCTGCGGGGCCGTCAGGGTGCCTGCGGGACGCTCACCTGGCTGGCCCGCCCAG |
| hsa-miR-6511a-5p | CAGGCAGAAGTGGGGCTGACAGG | CCTGCAGGCAGAAGTGGGGCTGACAGGGCAGAGGGTTGCGCCCCCTCACCATCCCTTCTGCCTGCAG |
| hsa-miR-1267 | CCTGTTGAAGTGTAATCCCCA | CTCCCAAATCTCCTGTTGAAGTGTAATCCCCACCTCCAGCATTGGGGATTACATTTCAACATGAGATTTGGATGAGGA |
| hsa-miR-4668-5p | AGGGAAAAAAAAAAGGATTTGTC | AGGGAAAAAAAAAAGGATTTGTCTTGTAGCCAGGATATTGTTTTAAAGAAAATCCTTTTTGTTTTTCCAG |
| hsa-miR-4671-5p | ACCGAAGACTGTGCGCTAATCT | TATTTTAAGACCGAAGACTGTGCGCTAATCTCTTAGCACTGAAGATTAGTGCATAGTCTTTGGTCTCAAAATA |
| hsa-miR-135a-3p | TATAGGGATTGGAGCCGTGGCG | AGGCCTCGCTGTTCTCTATGGCTTTTTATTCCTATGTGATTCTACTGCTCACTCATATAGGGATTGGAGCCGTGGCGCACGGCGGGGACA |
| hsa-miR-548b-5p | AAAAGTAATTGTGGTTTTGGCC | CAGACTATATATTTAGGTTGGCGCAAAAGTAATTGTGGTTTTGGCCTTTATTTTCAATGGCAAGAACCTCAGTTGCTTTTGTGCCAACCTAATACTT |
| hsa-miR-519c-5p | CTCTAGAGGGAAGCGCTTTCTG | TCTCAGGCTGTGACCCTCTAGAGGGAAGCGCTTTCTGTTGGCTAAAAGAAAAGAAAGCGCTTCCCTTCAGAGTGTTAACGCTTTGAGA |
| hsa-miR-205-5p | TCCTTCATTCCACCGGAGTCTG | AAAGATCCTCAGACAATCCATGTGCTTCTCTTGTCCTTCATTCCACCGGAGTCTGTCTCATACCCAACCAGATTTCAGTGGAGTGAAGTTCAGGAGGCATGGAGCTGACA |
| hsa-miR-6836-5p | CGCAGGGCCCTGGCGCAGGCAT | GGCTCCGCAGGGCCCTGGCGCAGGCATCCAGACAGCGGGCGAATGCCTCCCCCGGCCCCGCAG |
| hsa-miR-6515-5p | TTGGAGGGTGTGGAAGACATC | CATTGGAGGGTGTGGAAGACATCTGGGCCAACTCTGATCTCTTCATCTACCCCCCAG |
| hsa-miR-6503-5p | AGGTCTGCATTCAAATCCCCAGA | AATGGTCCCCCCAGGGAGGTCTGCATTCAAATCCCCAGAAGCTGAGGATTAGGGGACTAGGATGCAGACCTCCCTGGGGGACCATT |
| hsa-miR-6504-5p | TCTGGCTGTGCTGTAATGCAG | GCAGTCTGGCTGTGCTGTAATGCAGTCTGCACCCTGCTGCATTACAGCACAGCCATTCTCT |
| hsa-miR-627-5p | GTGAGTCTCTAAGAAAAGAGGA | TACTTATTACTGGTAGTGAGTCTCTAAGAAAAGAGGAGGTGGTTGTTTTCCTCCTCTTTTCTTTGAGACTCACTACCAATAATAAGAAATACTACTA |
| hsa-miR-513a-3p | TAAATTTCACCTTTCTGAGAAGG | GGGATGCCACATTCAGCCATTCAGCGTACAGTGCCTTTCACAGGGAGGTGTCATTTATGTGAACTAAAATATAAATTTCACCTTTCTGAGAAGGGTAATGTACAGCATGCACTGCATATGTGGTGTCCC |
| hsa-miR-210-5p | AGCCCCTGCCCACCGCACACTG | ACCCGGCAGTGCCTCCAGGCGCAGGGCAGCCCCTGCCCACCGCACACTGCGCTGCCCCAGACCCACTGTGCGTGTGACAGCGGCTGATCTGTGCCTGGGCAGCGCGACCC |
| hsa-miR-4708-3p | AGCAAGGCGGCATCTCTCTGAT | TTTAGGAGAGAGATGCCGCCTTGCTCCTTGAACAGGAGGAGCAAGGCGGCATCTCTCTGATACTAAA |
| hsa-miR-3173-5p | TGCCCTGCCTGTTTTCTCCTTT | TCCCTGCCCTGCCTGTTTTCTCCTTTGTGATTTTATGAGAACAAAGGAGGAAATAGGCAGGCCAGGGA |
| hsa-miR-4748 | GAGGTTTGGGGAGGATTTGCT | TGGCTGGCTGAGGTTTGGGGAGGATTTGCTGGTGCTAGAGAGGAAAGCAGACCCTACCCAACCCCACGCCCTACTACAGCCA |
| hsa-miR-223-3p | TGTCAGTTTGTCAAATACCCCA | CCTGGCCTCCTGCAGTGCCACGCTCCGTGTATTTGACAAGCTGAGTTGGACACTCCATGTGGTAGAGTGTCAGTTTGTCAAATACCCCAAGTGCGGCACATGCTTACCAG |
| hsa-miR-142-5p | CATAAAGTAGAAAGCACTACT | GACAGTGCAGTCACCCATAAAGTAGAAAGCACTACTAACAGCACTGGAGGGTGTAGTGTTTCCTACTTTATGGATGAGTGTACTGTG |
| hsa-miR-6803-3p | TCCCTCGCCTTCTCACCCTCAG | CTCCTCTGGGGGTGGGGGGCTGGGCGTGGTGGACAGCGATGCATCCCTCGCCTTCTCACCCTCAG |
| hsa-miR-6511a-3p | CCTCACCATCCCTTCTGCCTGC | CCTGCAGGCAGAAGTGGGGCTGACAGGGCAGAGGGTTGCGCCCCCTCACCATCCCTTCTGCCTGCAG |
| hsa-miR-507 | TTTTGCACCTTTTGGAGTGAA | GTGCTGTGTGTAGTGCTTCACTTCAAGAAGTGCCATGCATGTGTCTAGAAATATGTTTTGCACCTTTTGGAGTGAAATAATGCACAACAGATAC |
| hsa-miR-4761-5p | ACAAGGTGTGCATGCCTGACC | GGACAAGGTGTGCATGCCTGACCCGTTGTCAGACCTGGAAAAAGGGCCGGCTGTGGGCAGGGAGGGCATGCGCACTTTGTCC |
| hsa-miR-186-3p | GCCCAAAGGTGAATTTTTTGGG | TGCTTGTAACTTTCCAAAGAATTCTCCTTTTGGGCTTTCTGGTTTTATTTTAAGCCCAAAGGTGAATTTTTTGGGAAGTTTGAGCT |
| hsa-miR-6769b-3p | CCCTCTCTGTCCCACCCATAG | CTTCCTGGTGGGTGGGGAGGAGAAGTGCCGTCCTCATGAGCCCCTCTCTGTCCCACCCATAG |
| hsa-miR-7106-3p | AGCTCCCTGAATCCCTGTCCCAG | GCTTCTGGGAGGAGGGGATCTTGGGAGTGATCCCAACAGCTGAGCTCCCTGAATCCCTGTCCCAG |
| hsa-miR-10a-3p | CAAATTCGTATCTAGGGGAATA | GATCTGTCTGTCTTCTGTATATACCCTGTAGATCCGAATTTGTGTAAGGAATTTTGTGGTCACAAATTCGTATCTAGGGGAATATGTAGTTGACATAAACACTCCGCTCT |
| hsa-miR-6815-3p | TGGCTTCTCTTGCACACCCAG | CACTGTAGGTGGCGCCGGAGGAGTCATTTCCCATCACTAATGGCTTCTCTTGCACACCCAG |
| hsa-miR-1-3p | TGGAATGTAAAGAAGTATGTAT | ACCTACTCAGAGTACATACTTCTTTATGTACCCATATGAACATACAATGCTATGGAATGTAAAGAAGTATGTATTTTTGGTAGGC |
| hsa-miR-200a-5p | CATCTTACCGGACAGTGCTGGA | CCGGGCCCCTGTGAGCATCTTACCGGACAGTGCTGGATTTCCCAGCTTGACTCTAACACTGTCTGGTAACGATGTTCAAAGGTGACCCGC |
| hsa-miR-943 | CTGACTGTTGCCGTCCTCCAG | GGGACGTTCTGAGCTCGGGGTGGGGGACGTTTGCCGGTCACTGCTGCTGGCGCCCTGACTGTTGCCGTCCTCCAGCCCCACTCAAAGGCATCCC |
| hsa-miR-6890-3p | CCACTGCCTATGCCCCACAG | TGGGCCATGGGGTAGGGCAGAGTAGGGCTGGATGGTAGGGCCCACTGCCTATGCCCCACAG |
| hsa-let-7b-3p | CTATACAACCTACTGCCTTCCC | CGGGGTGAGGTAGTAGGTTGTGTGGTTTCAGGGCAGTGATGTTGCCCCTCGGAAGATAACTATACAACCTACTGCCTTCCCTG |
| hsa-miR-3149 | TTTGTATGGATATGTGTGTGTAT | ATACATACATGTACACACACATGTCATCCACACACATACATATATATATGTTTGTATGGATATGTGTGTGTATGTGTGTGTAT |
| hsa-miR-146b-3p | GCCCTGTGGACTCAGTTCTGGT | CCTGGCACTGAGAACTGAATTCCATAGGCTGTGAGCTCTAGCAATGCCCTGTGGACTCAGTTCTGGTGCCCGG |
| hsa-miR-10399-3p | CTCTCGGACAAGCTGTAGGTC | AATTACAGATTGTCTCAGAGAAAACAAATGAGTTACTCTCTCGGACAAGCTGTAGGTC |
| hsa-miR-4731-5p | TGCTGGGGGCCACATGAGTGTG | CCCTGCCAGTGCTGGGGGCCACATGAGTGTGCAGTCATCCACACACAAGTGGCCCCCAACACTGGCAGGG |
| hsa-let-7g-3p | CTGTACAGGCCACTGCCTTGC | AGGCTGAGGTAGTAGTTTGTACAGTTTGAGGGTCTATGATACCACCCGGTACAGGAGATAACTGTACAGGCCACTGCCTTGCCA |
| hsa-miR-551b-5p | GAAATCAAGCGTGGGTGAGACC | AGATGTGCTCTCCTGGCCCATGAAATCAAGCGTGGGTGAGACCTGGTGCAGAACGGGAAGGCGACCCATACTTGGTTTCAGAGGCTGTGAGAATAA |
| hsa-miR-769-5p | TGAGACCTCTGGGTTCTGAGCT | GCCTTGGTGCTGATTCCTGGGCTCTGACCTGAGACCTCTGGGTTCTGAGCTGTGATGTTGCTCTCGAGCTGGGATCTCCGGGGTCTTGGTTCAGGGCCGGGGCCTCTGGGTTCCAAGC |
| hsa-miR-3186-3p | TCACGCGGAGAGATGGCTTTG | AGCCTGCGGTTCCAACAGGCGTCTGTCTACGTGGCTTCAACCAAGTTCAAAGTCACGCGGAGAGATGGCTTTGGAACCAGGGGCT |
| hsa-miR-19a-3p | TGTGCAAATCTATGCAAAACTGA | GCAGTCCTCTGTTAGTTTTGCATAGTTGCACTACAAGAAGAATGTAGTTGTGCAAATCTATGCAAAACTGATGGTGGCCTGC |
| hsa-miR-381-5p | AGCGAGGTTGCCCTTTGTATAT | TACTTAAAGCGAGGTTGCCCTTTGTATATTCGGTTTATTGACATGGAATATACAAGGGCAAGCTCTCTGTGAGTA |
| hsa-miR-6741-3p | TCGGCTCTCTCCCTCACCCTAG | AATGGGTGGGTGCTGGTGGGAGCCGTGCCCTGGCCACTCATTCGGCTCTCTCCCTCACCCTAG |
| hsa-miR-7155-5p | TCTGGGGTCTTGGGCCATC | TCTGGGGTCTTGGGCCATCTGGTTGTGACATCACTGATGGCCCAAGACCTCAGACC |
| hsa-miR-544b | ACCTGAGGTTGTGCATTTCTAA | GGAATTTTGTTAAAATGCAGAATCCATTTCTGTAGCTCTGAGACTAGACCTGAGGTTGTGCATTTCTAACAAAGTGCC |
| hsa-miR-4505 | AGGCTGGGCTGGGACGGA | GGAGGCTGGGCTGGGACGGACACCCGGCCTCCACTTTCTGTGGCAGGTACCTCCTCCATGTCGGCCCGCCTTG |
| hsa-miR-4633-3p | AGGAGCTAGCCAGGCATATGCA | TGGCAAGTCTCCGCATATGCCTGGCTAGCTCCTCCACAAATGCGTGTGGAGGAGCTAGCCAGGCATATGCAGAGCGTCA |
| hsa-miR-3677-5p | CAGTGGCCAGAGCCCTGCAGTG | GGCAGTGGCCAGAGCCCTGCAGTGCTGGGCATGGGCTTCTCGTGGGCTCTGGCCACGGCC |
| hsa-miR-4491 | AATGTGGACTGGTGTGACCAAA | ACATTTGGTCACACCAGTCCACATTAACGTGGACCAGACAATATTAATGTGGACTGGTGTGACCAAAA |
| hsa-miR-668-3p | TGTCACTCGGCTCGGCCCACTAC | GGTAAGTGCGCCTCGGGTGAGCATGCACTTAATGTGGGTGTATGTCACTCGGCTCGGCCCACTACC |
| hsa-miR-938 | TGCCCTTAAAGGTGAACCCAGT | GAAGGTGTACCATGTGCCCTTAAAGGTGAACCCAGTGCACCTTCATGAACCGTGGTACACCTTTAAGAACTTGGTATGCCTTC |
| hsa-miR-3074-3p | GATATCAGCTCAGTAGGCACCG | GCTCGACTCCTGTTCCTGCTGAACTGAGCCAGTGTGTAAAATGAGAACTGATATCAGCTCAGTAGGCACCGGAGGGCGGGT |
| hsa-miR-6716-3p | TCCGAACTCTCCATTCCTCTGC | GAGAGGCCAAGACCTTGGGAATGGGGGTAAGGGCCTTCTGAGCCCAGGTCCGAACTCTCCATTCCTCTGCAGAGCGCTCT |
| hsa-miR-342-3p | TCTCACACAGAAATCGCACCCGT | GAAACTGGGCTCAAGGTGAGGGGTGCTATCTGTGATTGAGGGACATGGTTAATGGAATTGTCTCACACAGAAATCGCACCCGTCACCTTGGCCTACTTA |
| hsa-miR-584-5p | TTATGGTTTGCCTGGGACTGAG | TAGGGTGACCAGCCATTATGGTTTGCCTGGGACTGAGGAATTTGCTGGGATATGTCAGTTCCAGGCCAACCAGGCTGGTTGGTCTCCCTGAAGCAAC |
| hsa-miR-5587-5p | ATGGTCACCTCCGGGACT | ATGGTCACCTCCGGGACTCAGCCCTGTGCTGAGCCCCGGGCAGTGTGATCATC |
| hsa-miR-542-5p | TCGGGGATCATCATGTCACGAGA | CAGATCTCAGACATCTCGGGGATCATCATGTCACGAGATACCAGTGTGCACTTGTGACAGATTGATAACTGAAAGGTCTGGGAGCCACTCATCTTCA |
| hsa-miR-19b-3p | TGTGCAAATCCATGCAAAACTGA | CACTGTTCTATGGTTAGTTTTGCAGGTTTGCATCCAGCTGTGTGATATTCTGCTGTGCAAATCCATGCAAAACTGACTGTGGTAGTG |
| hsa-miR-10396a-3p | GGCCCCGGGCCCTCGACCGGG | GGCGGGGCTCGGAGCCGGGCTTCGGCCGGGCCCCGGGCCCTCGACCGGG |
| hsa-miR-675-5p | TGGTGCGGAGAGGGCCCACAGTG | CCCAGGGTCTGGTGCGGAGAGGGCCCACAGTGGACTTGGTGACGCTGTATGCCCTCACCGCTCAGCCCCTGGG |
| hsa-miR-199a-5p | CCCAGTGTTCAGACTACCTGTTC | GCCAACCCAGTGTTCAGACTACCTGTTCAGGAGGCTCTCAATGTGTACAGTAGTCTGCACATTGGTTAGGC |
| hsa-miR-513b-5p | TTCACAAGGAGGTGTCATTTAT | GTGTACAGTGCCTTTCACAAGGAGGTGTCATTTATGTGAACTAAAATATAAATGTCACCTTTTTGAGAGGAGTAATGTACAGCA |
| hsa-miR-4455 | AGGGTGTGTGTGTTTTT | AGAAGGGTGTGTGTGTTTTTCCTGAGAATAAGAGAAGGAAGGACAGCCAAATTCTTCA |
| hsa-miR-3682-3p | TGATGATACAGGTGGAGGTAG | TAAGTTATATATGTCTACTTCTACCTGTGTTATCATAATAAAGGTGTCATGATGATACAGGTGGAGGTAGAAATATATAACTTA |
| hsa-miR-939-3p | CCCTGGGCCTCTGCTCCCCAG | TGTGGGCAGGGCCCTGGGGAGCTGAGGCTCTGGGGGTGGCCGGGGCTGACCCTGGGCCTCTGCTCCCCAGTGTCTGACCGCG |
| hsa-miR-548ad-5p | AAAAGTAATTGTGGTTTTTG | CTGTTAGGTTGGTGCAAAAGTAATTGTGGTTTTTGAAAGTAACTTGGCGAAAACGACAATGACTTTTGCACCAATCTAATAC |
| hsa-miR-339-3p | TGAGCGCCTCGACGACAGAGCCG | CGGGGCGGCCGCTCTCCCTGTCCTCCAGGAGCTCACGTGTGCCTGCCTGTGAGCGCCTCGACGACAGAGCCGGCGCCTGCCCCAGTGTCTGCGC |
| hsa-miR-1246 | AATGGATTTTTGGAGCAGG | TGTATCCTTGAATGGATTTTTGGAGCAGGAGTGGACACCTGACCCAAAGGAAATCAATCCATAGGCTAGCAAT |
| hsa-miR-600 | ACTTACAGACAAGAGCCTTGCTC | AAGTCACGTGCTGTGGCTCCAGCTTCATAGGAAGGCTCTTGTCTGTCAGGCAGTGGAGTTACTTACAGACAAGAGCCTTGCTCAGGCCAGCCCTGCCC |
| hsa-miR-3135b | GGCTGGAGCGAGTGCAGTGGTG | TGCCCAGGCTGGAGCGAGTGCAGTGGTGCAGTCAGTCCTAGCTCACTGCAGCCTCGAACTCCTGGGCT |
| hsa-miR-129-1-3p | AAGCCCTTACCCCAAAAAGTAT | GGATCTTTTTGCGGTCTGGGCTTGCTGTTCCTCTCAACAGTAGTCAGGAAGCCCTTACCCCAAAAAGTATCT |
| hsa-miR-876-5p | TGGATTTCTTTGTGAATCACCA | TGAAGTGCTGTGGATTTCTTTGTGAATCACCATATCTAAGCTAATGTGGTGGTGGTTTACAAAGTAATTCATAGTGCTTCA |
| hsa-miR-3085-3p | TCTGGCTGCTATGGCCCCCTC | CCCTACTCTGGGAAGGTGCCATTCTGAGGGCCAGGAGTTTGATTATGTGTCACTCTGGCTGCTATGGCCCCCTCCCAGGGTCTGG |
| hsa-miR-582-5p | TTACAGTTGTTCAACCAGTTACT | ATCTGTGCTCTTTGATTACAGTTGTTCAACCAGTTACTAATCTAACTAATTGTAACTGGTTGAACAACTGAACCCAAAGGGTGCAAAGTAGAAACATT |
| hsa-miR-766-5p | AGGAGGAATTGGTGCTGGTCTT | GCATCCTCAGGACCTGGGCTTGGGTGGTAGGAGGAATTGGTGCTGGTCTTTCATTTTGGATTTGACTCCAGCCCCACAGCCTCAGCCACCCCAGCCAATTGTCATAGGAGC |
| hsa-miR-548ad-5p | AAAAGTAATTGTGGTTTTTG | TGTGCAAAAGTAATTGTGGTTTTTGTCATTTAAAAGTAATGGCAAAAACTGCAATTACTTTCACACC |
| hsa-miR-1271-3p | AGTGCCTGCTATGTGCCAGGCA | CACCCAGATCAGTGCTTGGCACCTAGCAAGCACTCAGTAAATATTTGTTGAGTGCCTGCTATGTGCCAGGCATTGTGCTGAGGGCT |
| hsa-miR-200c-3p | TAATACTGCCGGGTAATGATGGA | CCCTCGTCTTACCCAGCAGTGTTTGGGTGCGGTTGGGAGTCTCTAATACTGCCGGGTAATGATGGAGG |
| hsa-miR-6810-5p | ATGGGGACAGGGATCAGCATGGC | CTGGGATGGGGACAGGGATCAGCATGGCACAGATCCAATACCTTCTGTCCCCTGCTCCCTTGTTCCCCAG |
| hsa-miR-4744 | TCTAAAGACTAGACTTCGCTATG | GTAATCACATCTAAAGACTAGACTTCGCTATGACCAGGCCATAGTAAACATCATAGTATGTCTAGTCTTTAGGTTTGATTAC |
| hsa-miR-3925-3p | ACTCCAGTTTTAGTTCTCTTG | GTGGGAATAGCAAGAGAACTGAAAGTGGAGCCTGTCACATCTCCAGACTCCAGTTTTAGTTCTCTTGCTATTTCCAC |
| hsa-miR-17-3p | ACTGCAGTGAAGGCACTTGTAG | GTCAGAATAATGTCAAAGTGCTTACAGTGCAGGTAGTGATATGTGCATCTACTGCAGTGAAGGCACTTGTAGCATTATGGTGAC |
| hsa-miR-196b-3p | TCGACAGCACGACACTGCCTTC | ACTGGTCGGTGATTTAGGTAGTTTCCTGTTGTTGGGATCCACCTTTCTCTCGACAGCACGACACTGCCTTCATTACTTCAGTTG |
| hsa-miR-196a-3p | CGGCAACAAGAAACTGCCTGAG | TGCTCGCTCAGCTGATCTGTGGCTTAGGTAGTTTCATGTTGTTGGGATTGAGTTTTGAACTCGGCAACAAGAAACTGCCTGAGTTACATCAGTCGGTTTTCGTCGAGGGC |
| hsa-miR-3200-3p | CACCTTGCGCTACTCAGGTCTG | GGTGGTCGAGGGAATCTGAGAAGGCGCACAAGGTTTGTGTCCAATACAGTCCACACCTTGCGCTACTCAGGTCTGCTCGTGCCCT |
| hsa-miR-320e | AAAGCTGGGTTGAGAAGG | CTCCATGGGGCCTTCTCTTCCCAGTTCTTCCTGGAGTCGGGGAAAAGCTGGGTTGAGAAGGTGAAAAGAAAAA |
| hsa-miR-4433a-5p | CGTCCCACCCCCCACTCCTGT | CATCCTCCTTACGTCCCACCCCCCACTCCTGTTTCTGGTGAAATATTCAAACAGGAGTGGGGGTGGGACATAAGGAGGATA |
| hsa-miR-4804-3p | TGCTTAACCTTGCCCTCGAAA | TCAGTGTATTTGGACGGTAAGGTTAAGCAAGGTGCGTCGTATCTTGCTTAACCTTGCCCTCGAAATACACTGA |
| hsa-miR-548ba | AAAGGTAACTGTGATTTTTGCT | AAAGGTAACTGTGATTTTTGCTATTAGAAAGTAATGGCAAAAACTGCAATTACTTT |
| hsa-miR-548d-3p | CAAAAACCACAGTTTCTTTTGC | AAACAAGTTATATTAGGTTGGTGCAAAAGTAATTGTGGTTTTTGCCTGTAAAAGTAATGGCAAAAACCACAGTTTCTTTTGCACCAGACTAATAAAG |
| hsa-miR-548az-5p | CAAAAGTGATTGTGGTTTTTGC | AGATTGTATTAGGTTGGTGCAAAAGTGATTGTGGTTTTTGCTGTTACTTTTAATGGCAAAAACTGCAATCACTTTTGCACCAACCTAATAAATTT |
| hsa-miR-4717-5p | TAGGCCACAGCCACCCATGTGT | GGCAGTGTTTAGGCCACAGCCACCCATGTGTAGGGGTGGCTACACATGGGTGGCTGTGGCCTAAACACTGCC |
| hsa-miR-3194-5p | GGCCAGCCACCAGGAGGGCTG | AGGTGGCAGGGCCAGCCACCAGGAGGGCTGCGTGCCACCCGGGCAGCTCTGCTGCTCACTGGCAGTGTCACCT |
| hsa-miR-605-3p | AGAAGGCACTATGAGATTTAGA | GCCCTAGCTTGGTTCTAAATCCCATGGTGCCTTCTCCTTGGGAAAAACAGAGAAGGCACTATGAGATTTAGAATCAAGTTAGG |
| hsa-miR-4706 | AGCGGGGAGGAAGTGGGCGCTGCTT | GCTACGGGGAGCGGGGAGGAAGTGGGCGCTGCTTCTGCGTTATCTGGAAGGAGCAGCCCACTCCTGTCCTGGGCTCTGTGGT |
| hsa-miR-4725-3p | TGGGGAAGGCGTCAGTGTCGGG | GTGTCTCTCTGGAGACCCTGCAGCCTTCCCACCCACCAGGGAGCTTTCCATGGGCTGTGGGGAAGGCGTCAGTGTCGGGTGAGGGAACAC |
| hsa-miR-503-3p | GGGGTATTGTTTCCGCTGCCAGG | TGCCCTAGCAGCGGGAACAGTTCTGCAGTGAGCGATCGGTGCTCTGGGGTATTGTTTCCGCTGCCAGGGTA |
| hsa-miR-6731-3p | TCTATTCCCCACTCTCCCCAG | ACAGGTGGGAGAGCAGGGTATTGTGGAAGCTCCAGGTGCCAACCACCTGCCTCTATTCCCCACTCTCCCCAG |
| hsa-miR-1307-3p | ACTCGGCGTGGCGTCGGTCGTG | CATCAAGACCCAGCTGAGTCACTGTCACTGCCTACCAATCTCGACCGGACCTCGACCGGCTCGTCTGTGTTGCCAATCGACTCGGCGTGGCGTCGGTCGTGGTAGATAGGCGGTCATGCATACGAATTTTCAGCTCTTGTTCTGGTGAC |
| hsa-miR-376a-3p | ATCATAGAGGAAAATCCACGT | TAAAAGGTAGATTCTCCTTCTATGAGTACATTATTTATGATTAATCATAGAGGAAAATCCACGTTTTC |
| hsa-miR-5000-5p | CAGTTCAGAAGTGTTCCTGAGT | CTGAAGAGTAGAGTGTGTGGTCCCAGTTCAGAAGTGTTCCTGAGTAACTTGTGCTTATAACTCAGGACACTTCTGAACTTGGACCATACAGGTCTCCCTGCTT |
| hsa-miR-3065-5p | TCAACAAAATCACTGATGCTGGA | CTGCCCTCTTCAACAAAATCACTGATGCTGGAGTCGCCTGAGTCATCACTCAGCACCAGGATATTGTTGGAGAGGACAG |
| hsa-miR-6822-3p | AGGCTCTAACTGGCTTTCCCTGCA | TGGCCCAGGGAACCAGTTGGGGCTTCCGCTCTGCAGAGGCTCTAACTGGCTTTCCCTGCAG |
| hsa-miR-217-3p | CATCAGTTCCTAATGCATTGCC | AGTATAATTATTACATAGTTTTTGATGTCGCAGATACTGCATCAGGAACTGATTGGATAAGAATCAGTCACCATCAGTTCCTAATGCATTGCCTTCAGCATCTAAACAAG |
| hsa-miR-125b-2-3p | TCACAAGTCAGGCTCTTGGGAC | ACCAGACTTTTCCTAGTCCCTGAGACCCTAACTTGTGAGGTATTTTAGTAACATCACAAGTCAGGCTCTTGGGACCTAGGCGGAGGGGA |
| hsa-miR-548c-5p | AAAAGTAATTGCGGTTTTTGCC | AGTTGGTGCAAAAGTAATTGCGGTTTTTGCCGTCGAAAATAATGGCAAAAACTGCAGTTACTTTTGTACCAATG |
| hsa-miR-323b-3p | CCCAATACACGGTCGACCTCTT | TGGTACTCGGAGGGAGGTTGTCCGTGGTGAGTTCGCATTATTTAATGATGCCCAATACACGGTCGACCTCTTTTCGGTATCA |
| hsa-miR-4489 | TGGGGCTAGTGATGCAGGACG | GGGGGTGGGGCTAGTGATGCAGGACGCTGGGGACTGGAGAAGTCCTGCCTGACCCTGTCCCA |
| hsa-miR-6862-3p | CCTCACCCAGCTCTCTGGCCCTCT | CGAAGCGGGCATGCTGGGAGAGACTTTGTGATTTGTCTCCAAAGCCTCACCCAGCTCTCTGGCCCTCTAG |
| hsa-miR-194-3p | CCAGTGGGGCTGCTGTTATCTG | TGGTTCCCGCCCCCTGTAACAGCAACTCCATGTGGAAGTGCCCACTGGTTCCAGTGGGGCTGCTGTTATCTGGGGCGAGGGCCAG |
| hsa-miR-223-5p | CGTGTATTTGACAAGCTGAGTT | CCTGGCCTCCTGCAGTGCCACGCTCCGTGTATTTGACAAGCTGAGTTGGACACTCCATGTGGTAGAGTGTCAGTTTGTCAAATACCCCAAGTGCGGCACATGCTTACCAG |
| hsa-miR-520b-3p | AAAGTGCTTCCTTTTAGAGGG | CCCTCTACAGGGAAGCGCTTTCTGTTGTCTGAAAGAAAAGAAAGTGCTTCCTTTTAGAGGG |
| hsa-miR-4740-3p | GCCCGAGAGGATCCGTCCCTGC | GCCAAGGACTGATCCTCTCGGGCAGGGAGTCAGAGGGGACCGCCCGAGAGGATCCGTCCCTGC |
| hsa-miR-518b | CAAAGCGCTCCCCTTTAGAGGT | TCATGCTGTGGCCCTCCAGAGGGAAGCGCTTTCTGTTGTCTGAAAGAAAACAAAGCGCTCCCCTTTAGAGGTTTACGGTTTGA |
| hsa-miR-504-3p | GGGAGTGCAGGGCAGGGTTTC | GCTGCTGTTGGGAGACCCTGGTCTGCACTCTATCTGTATTCTTACTGAAGGGAGTGCAGGGCAGGGTTTCCCATACAGAGGGC |
| hsa-miR-149-3p | AGGGAGGGACGGGGGCTGTGC | GCCGGCGCCCGAGCTCTGGCTCCGTGTCTTCACTCCCGTGCTTGTCCGAGGAGGGAGGGAGGGACGGGGGCTGTGCTGGGGCAGCTGGA |
| hsa-miR-26b-5p | TTCAAGTAATTCAGGATAGGT | CCGGGACCCAGTTCAAGTAATTCAGGATAGGTTGTGTGCTGTCCAGCCTGTTCTCCATTACTTGGCTCGGGGACCGG |
| hsa-miR-5708 | ATGAGCGACTGTGCCTGACC | ATTACAGACATGAGCGACTGTGCCTGACCAAAAGTCAACATTAAACAACAAATCTTGGCCAGGCACAGTGGCTCATGCCTGTAAT |
| hsa-miR-573 | CTGAAGTGATGTGTAACTGATCAG | TTTAGCGGTTTCTCCCTGAAGTGATGTGTAACTGATCAGGATCTACTCATGTCGTCTTTGGTAAAGTTATGTCGCTTGTCAGGGTGAGGAGAGTTTTTG |
| hsa-miR-1236-3p | CCTCTTCCCCTTGTCTCTCCAG | GTGAGTGACAGGGGAAATGGGGATGGACTGGAAGTGGGCAGCATGGAGCTGACCTTCATCATGGCTTGGCCAACATAATGCCTCTTCCCCTTGTCTCTCCAG |
| hsa-miR-3912-5p | ATGTCCATATTATGGGTTAGT | AGAGAGGAATGAACAGTTAAATTATAACATGTCCATATTATGGGTTAGTTGTGGACACATACTAACGCATAATATGGACATGTTATAATTTAACTGTTCCTTTCT |
| hsa-miR-660-3p | ACCTCCTGTGTGCATGGATTA | CTGCTCCTTCTCCCATACCCATTGCATATCGGAGTTGTGAATTCTCAAAACACCTCCTGTGTGCATGGATTACAGGAGGGTGAGCCTTGTCATCGTG |
| hsa-miR-3655 | GCTTGTCGCTGCGGTGTTGCT | GCTTGTCGCTGCGGTGTTGCTGTTGGAGACTCGATTGTTGGTGACAGCGAAAGAACGATAACAAAATGCCGGAGCGAGATAGT |
| hsa-miR-3160-5p | GGCTTTCTAGTCTCAGCTCTCC | GGACCTGCCCTGGGCTTTCTAGTCTCAGCTCTCCTCCAGCTCAGCTGGTCAGGAGAGCTGAGACTAGAAAGCCCAGGGCAGGTTC |
| hsa-miR-765 | TGGAGGAGAAGGAAGGTGATG | TTTAGGCGCTGATGAAAGTGGAGTTCAGTAGACAGCCCTTTTCAAGCCCTACGAGAAACTGGGGTTTCTGGAGGAGAAGGAAGGTGATGAAGGATCTGTTCTCGTGAGCCTGAA |
| hsa-miR-12135 | TAAAGGTTTGTTTGTAAA | TGTGGATATTCTTTTTTGATACTACAGCAAAACTCAGCAAGTTGTAGTTTTTTAAAGGTTTGTTTGTAAA |
| hsa-miR-628-3p | TCTAGTAAGAGTGGCAGTCGA | ATAGCTGTTGTGTCACTTCCTCATGCTGACATATTTACTAGAGGGTAAAATTAATAACCTTCTAGTAAGAGTGGCAGTCGAAGGGAAGGGCTCAT |
| hsa-miR-532-5p | CATGCCTTGAGTGTAGGACCGT | CGACTTGCTTTCTCTCCTCCATGCCTTGAGTGTAGGACCGTTGGCATCTTAATTACCCTCCCACACCCAAGGCTTGCAGAAGAGCGAGCCT |
| hsa-miR-296-5p | AGGGCCCCCCCTCAATCCTGT | AGGACCCTTCCAGAGGGCCCCCCCTCAATCCTGTTGTGCCTAATTCAGAGGGTTGGGTGGAGGCTCTCCTGAAGGGCTCT |
| hsa-miR-378a-5p | CTCCTGACTCCAGGTCCTGTGT | AGGGCTCCTGACTCCAGGTCCTGTGTGTTACCTAGAAATAGCACTGGACTTGGAGTCAGAAGGCCT |
| hsa-miR-6769a-3p | GAGCCCCTCTCTGCTCTCCAG | AGGCCAGGTGGGTATGGAGGAGCCCTCATATGGCAGTTGGCGAGGGCCCAGTGAGCCCCTCTCTGCTCTCCAG |
| hsa-miR-6802-5p | CTAGGTGGGGGGCTTGAAGC | GAGGGCTAGGTGGGGGGCTTGAAGCCCCGAGATGCCTCACGTCTTCACCCCTCTCACCTAAGCAG |
| hsa-miR-1911-5p | TGAGTACCGCCATGTCTGTTGGG | TCGGCATCTGCTGAGTACCGCCATGTCTGTTGGGCATCCACAGTCTCCCACCAGGCATTGTGGTCTCCGCTGACGCTTTG |
| hsa-miR-6128 | ACTGGAATTGGAGTCAAAA | AAGAAGCTTGTAGATTTTTCTCCCTTACTATCTAGAATTATAGGACTTCAGTCCATGATTTGGAAAAATTACTGGAATTGGAGTCAAAAATAATTTGAAAATTAGGAAT |
| hsa-miR-20a-5p | TAAAGTGCTTATAGTGCAGGTAG | GTAGCACTAAAGTGCTTATAGTGCAGGTAGTGTTTAGTTATCTACTGCATTATGAGCACTTAAAGTACTGC |
| hsa-miR-5586-5p | TATCCAGCTTGTTACTATATGC | TATCCAGCTTGTTACTATATGCTTTTTAAATGGGGCACAGAGTGACAAGCTGGTTAAAG |
| hsa-miR-188-3p | CTCCCACATGCAGGGTTTGCA | TGCTCCCTCTCTCACATCCCTTGCATGGTGGAGGGTGAGCTTTCTGAAAACCCCTCCCACATGCAGGGTTTGCAGGATGGCGAGCC |
| hsa-miR-21-3p | CAACACCAGTCGATGGGCTGT | TGTCGGGTAGCTTATCAGACTGATGTTGACTGTTGAATCTCATGGCAACACCAGTCGATGGGCTGTCTGACA |
| hsa-miR-6859-5p | GAGAGGAACATGGGCTCAGGACA | TGTGGGAGAGGAACATGGGCTCAGGACAGCGGGTGTCAGCTTGCCTGACCCCCATGTCGCCTCTGTAG |
| hsa-miR-550b-2-5p | ATGTGCCTGAGGGAGTAAGACA | AGAGACTTGTTGGAGATGTGCCTGAGGGAGTAAGACACTATCTTACAACAACAGGGCTCTTACTCCCTCAGGCACTGCACCAGCCAGCAAAGCATCA |
| hsa-miR-2355-3p | ATTGTCCTTGCTGTTTGGAGAT | CAGACGTGTCATCCCCAGATACAATGGACAATATGCTATTATAATCGTATGGCATTGTCCTTGCTGTTTGGAGATAATACTGCTGAC |
| hsa-miR-148b-3p | TCAGTGCATCACAGAACTTTGT | CAAGCACGATTAGCATTTGAGGTGAAGTTCTGTTATACACTCAGGCTGTGGCTCTCTGAAAGTCAGTGCATCACAGAACTTTGTCTCGAAAGCTTTCTA |
| hsa-miR-6864-3p | GTGAGACTTCTCTCCCTTCAG | GAAAGTTGAAGGGACAAGTCAGATATGCCATTATGTGGGTCATGAGGTGGTGAGACTTCTCTCCCTTCAG |
| hsa-miR-590-3p | TAATTTTATGTATAAGCTAGT | TAGCCAGTCAGAAATGAGCTTATTCATAAAAGTGCAGTATGGTGAAGTCAATCTGTAATTTTATGTATAAGCTAGTCTCTGATTGAAACATGCAGCA |
| hsa-miR-100-5p | AACCCGTAGATCCGAACTTGTG | CCTGTTGCCACAAACCCGTAGATCCGAACTTGTGGTATTAGTCCGCACAAGCTTGTATCTATAGGTATGTGTCTGTTAGG |
| hsa-miR-548u | CAAAGACTGCAATTACTTTTGCG | ATTAGGATGGTGCAAAAGTAATGTGGTTTTTTTCTTTACTTTTAATGGCAAAGACTGCAATTACTTTTGCGCCAACCTAAT |
| hsa-miR-6837-3p | CCTTCACTGTGACTCTGCTGCAG | GTGGGACCAGGGCCAGCAGGGAATGTCAGGGCCACCCCTGACCTTCACTGTGACTCTGCTGCAG |
| hsa-miR-320a-3p | AAAAGCTGGGTTGAGAGGGCGA | CTCCCCTCCGCCTTCTCTTCCCGGTTCTTCCCGGAGTCGGGAAAAGCTGGGTTGAGAGGGCGAAAAAGGATG |
| hsa-miR-6808-3p | GTGTGACCACCGTTCCTGCAG | GGGGCCAGGCAGGGAGGTGGGACCATGGGGGCCTTGCTGTGTGACCACCGTTCCTGCAG |
| hsa-miR-6743-3p | AGCCGCTCTTCTCCCTGCCCACA | GGGTAAAGGGGCAGGGACGGGTGGCCCCAGGAAGAAGGGCCTGGTGGAGCCGCTCTTCTCCCTGCCCACAG |
| hsa-miR-511-5p | GTGTCTTTTGCTCTGCAGTCA | CAATAGACACCCATCGTGTCTTTTGCTCTGCAGTCAGTAAATATTTTTTTGTGAATGTGTAGCAAAAGACAGAATGGTGGTCCATTG |
| hsa-miR-301a-3p | CAGTGCAATAGTATTGTCAAAGC | ACTGCTAACGAATGCTCTGACTTTATTGCACTACTGTACTTTACAGCTAGCAGTGCAATAGTATTGTCAAAGCATCTGAAAGCAGG |
| hsa-miR-3199 | AGGGACTGCCTTAGGAGAAAGTT | GGTGACTCCAGGGACTGCCTTAGGAGAAAGTTTCTGGAAGTTCTGACATTCCAGAAACTTTCTCCTAAGGCAGTCCCTGGGAGTCACT |
| hsa-miR-152-3p | TCAGTGCATGACAGAACTTGG | TGTCCCCCCCGGCCCAGGTTCTGTGATACACTCCGACTCGGGCTCTGGAGCAGTCAGTGCATGACAGAACTTGGGCCCGGAAGGACC |
| hsa-miR-361-5p | TTATCAGAATCTCCAGGGGTAC | GGAGCTTATCAGAATCTCCAGGGGTACTTTATAATTTCAAAAAGTCCCCCAGGTGTGATTCTGATTTGCTTC |
| hsa-miR-6757-3p | AACACTGGCCTTGCTATCCCCA | GGGCTTAGGGATGGGAGGCCAGGATGAAGATTAATCCCTAATCCCCAACACTGGCCTTGCTATCCCCAG |
| hsa-miR-6871-5p | CATGGGAGTTCGGGGTGGTTGC | CTCCTCATGGGAGTTCGGGGTGGTTGCTGGAGGTCAGCACCCTGTGGCTCCCACAG |
| hsa-miR-656-3p | AATATTATACAGTCAACCTCT | CTGAAATAGGTTGCCTGTGAGGTGTTCACTTTCTATATGATGAATATTATACAGTCAACCTCTTTCCGATATCGAATC |
| hsa-miR-4728-5p | TGGGAGGGGAGAGGCAGCAAGCA | GTGGGAGGGGAGAGGCAGCAAGCACACAGGGCCTGGGACTAGCATGCTGACCTCCCTCCTGCCCCAG |
| hsa-miR-23c | ATCACATTGCCAGTGATTACCC | AGTGACTTTCCAGGTGTCACACAGTGAGTGGCATAATCAGAGTACAATTTGAGTCATGCCCATACATCACATTGCCAGTGATTACCCAAGGAAAGTGACG |
| hsa-miR-4717-3p | ACACATGGGTGGCTGTGGCCT | GGCAGTGTTTAGGCCACAGCCACCCATGTGTAGGGGTGGCTACACATGGGTGGCTGTGGCCTAAACACTGCC |
| hsa-miR-517c-3p | ATCGTGCATCCTTTTAGAGTGT | GAAGATCTCAGGCAGTGACCCTCTAGATGGAAGCACTGTCTGTTGTCTAAGAAAAGATCGTGCATCCTTTTAGAGTGTTACTGTTTGAGAAAATC |
| hsa-miR-520c-3p | AAAGTGCTTCCTTTTAGAGGGT | TCTCAGGCTGTCGTCCTCTAGAGGGAAGCACTTTCTGTTGTCTGAAAGAAAAGAAAGTGCTTCCTTTTAGAGGGTTACCGTTTGAGA |
| hsa-miR-6854-5p | AAGCTCAGGTTTGAGAACTGCTGA | AAAGCAAGCTCAGGTTTGAGAACTGCTGATGTCATCAGTCATAACTTCTGCGTTTCTCCTCTTGAGCAG |
| hsa-miR-6849-3p | ACCAGCCTGTGTCCACCTCCAG | CCTGGGAGTGGATAGGGGAGTGTGTGGAGAGAGCACTGAGCCTGCCCACCAGCCTGTGTCCACCTCCAG |
| hsa-miR-509-3p | TGATTGGTACGTCTGTGGGTAG | CATGCTGTGTGTGGTACCCTACTGCAGACAGTGGCAATCATGTATAATTAAAAATGATTGGTACGTCTGTGGGTAGAGTACTGCATGACACATG |
| hsa-miR-4722-3p | ACCTGCCAGCACCTCCCTGCAG | GGCAGGAGGGCTGTGCCAGGTTGGCTGGGCCAGGCCTGACCTGCCAGCACCTCCCTGCAG |
| hsa-miR-6729-5p | TGGGCGAGGGCGGCTGAGCGGC | GAGGGTGGGCGAGGGCGGCTGAGCGGCTCCATCCCCCGGCCTGCTCATCCCCCTCGCCCTCTCAG |
| hsa-miR-4496 | GAGGAAACTGAAGCTGAGAGGG | ACATCAGCTCATATAATCCTCGAAGCTGCCTTTAGAAATGAGGAAACTGAAGCTGAGAGGG |
| hsa-miR-4782-5p | TTCTGGATATGAAGACAATCAA | ATTGCCCAGTTCTGGATATGAAGACAATCAAGAAAAGATTTGGTGTTCTTGATTGTCTTCATATCTAGAACTGGGCAGT |
| hsa-miR-1323 | TCAAAACTGAGGGGCATTTTCT | ACTGAGGTCCTCAAAACTGAGGGGCATTTTCTGTGGTTTGAAAGGAAAGTGCACCCAGTTTTGGGGATGTCAA |
| hsa-miR-708-3p | CAACTAGACTGTGAGCTTCTAG | AACTGCCCTCAAGGAGCTTACAATCTAGCTGGGGGTAAATGACTTGCACATGAACACAACTAGACTGTGAGCTTCTAGAGGGCAGGGA |
| hsa-let-7i-5p | TGAGGTAGTAGTTTGTGCTGTT | CTGGCTGAGGTAGTAGTTTGTGCTGTTGGTCGGGTTGTGACATTGCCCGCTGTGGAGATAACTGCGCAAGCTACTGCCTTGCTA |
| hsa-miR-4699-3p | AATTTACTCTGCAATCTTCTCC | AGCAATTGGAGAAGATTGCAGAGTAAGTTCCTGATTAAGAAATGGAATTTACTCTGCAATCTTCTCCAATTGCT |
| hsa-miR-4802-5p | TATGGAGGTTCTAGACCATGTT | CTGACTGGCTTGTATGGAGGTTCTAGACCATGTTAGTGTTCAAGTCTACATGGATGGAAACCTTCAAGCAGGCCAAGCAG |
| hsa-miR-654-3p | TATGTCTGCTGACCATCACCTT | GGGTAAGTGGAAAGATGGTGGGCCGCAGAACATGTGCTGAGTTCGTGCCATATGTCTGCTGACCATCACCTTTAGAAGCCC |
| hsa-miR-4679 | TCTGTGATAGAGATTCTTTGCT | GTCTTTTTTCTGTGATAGAGATTCTTTGCTTTGTTAGAAACAAAAAGCAAAGAATCTCTATCACAGAAAAAAGAT |
| hsa-miR-429 | TAATACTGTCTGGTAAAACCGT | CGCCGGCCGATGGGCGTCTTACCAGACATGGTTAGACCTGGCCCTCTGTCTAATACTGTCTGGTAAAACCGTCCATCCGCTGC |
| hsa-miR-584-3p | TCAGTTCCAGGCCAACCAGGCT | TAGGGTGACCAGCCATTATGGTTTGCCTGGGACTGAGGAATTTGCTGGGATATGTCAGTTCCAGGCCAACCAGGCTGGTTGGTCTCCCTGAAGCAAC |
| hsa-miR-508-3p | TGATTGTAGCCTTTTGGAGTAGA | CCACCTTCAGCTGAGTGTAGTGCCCTACTCCAGAGGGCGTCACTCATGTAAACTAAAACATGATTGTAGCCTTTTGGAGTAGAGTAATACACATCACGTAACGCATATTTGGTGG |
| hsa-miR-6837-5p | ACCAGGGCCAGCAGGGAATGT | GTGGGACCAGGGCCAGCAGGGAATGTCAGGGCCACCCCTGACCTTCACTGTGACTCTGCTGCAG |
| hsa-miR-200b-5p | CATCTTACTGGGCAGCATTGGA | CCAGCTCGGGCAGCCGTGGCCATCTTACTGGGCAGCATTGGATGGAGTCAGGTCTCTAATACTGCCTGGTAATGATGACGGCGGAGCCCTGCACG |
| hsa-miR-6801-3p | ACCCCTGCCACTCACTGGCC | TGGCCTGGTCAGAGGCAGCAGGAAATGAGAGTTAGCCAGGAGCTTTGCATACTCACCCCTGCCACTCACTGGCCCCCAG |
| hsa-miR-548d-5p | AAAAGTAATTGTGGTTTTTGCC | AAACAAGTTATATTAGGTTGGTGCAAAAGTAATTGTGGTTTTTGCCTGTAAAAGTAATGGCAAAAACCACAGTTTCTTTTGCACCAGACTAATAAAG |
| hsa-miR-3157-3p | CTGCCCTAGTCTAGCTGAAGCT | GGGAAGGGCTTCAGCCAGGCTAGTGCAGTCTGCTTTGTGCCAACACTGGGGTGATGACTGCCCTAGTCTAGCTGAAGCTTTTCCC |
| hsa-miR-3059-5p | TTTCCTCTCTGCCCCATAGGGTGT | AGGTGGTACACCCTTTCCTCTCTGCCCCATAGGGTGTAGCTCTAACTACCCTCTAGGGAAGAGAAGGTTGGGTGAACAGCCT |
| hsa-miR-324-5p | CGCATCCCCTAGGGCATTGGTG | CTGACTATGCCTCCCCGCATCCCCTAGGGCATTGGTGTAAAGCTGGAGACCCACTGCCCCAGGTGCTGCTGGGGGTTGTAGTC |
| hsa-miR-26a-1-3p | CCTATTCTTGGTTACTTGCACG | GTGGCCTCGTTCAAGTAATCCAGGATAGGCTGTGCAGGTCCCAATGGGCCTATTCTTGGTTACTTGCACGGGGACGC |
| hsa-miR-6855-5p | TTGGGGTTTGGGGTGCAGACATTGC | GCTGCTTGGGGTTTGGGGTGCAGACATTGCCAGAGGATGGGCAGCAGACTGACCTTCAACCCCACAG |
| hsa-miR-1294 | TGTGAGGTTGGCATTGTTGTCT | CACCTAATGTGTGCCAAGATCTGTTCATTTATGATCTCACCGAGTCCTGTGAGGTTGGCATTGTTGTCTGGCATTGTCTGATATACAACAGTGCCAACCTCACAGGACTCAGTGAGGTGAAACTGAGGATTAGGAAGGTGTA |
| hsa-miR-5696 | CTCATTTAAGTAGTCTGATGCC | GTGCTCATTTAAGTAGTCTGATGCCTACTACTGATGACATACAATGTAAGTGCTCATTTAGGCGTCAGACTACCTAAATGAGCAC |
| hsa-miR-3157-5p | TTCAGCCAGGCTAGTGCAGTCT | GGGAAGGGCTTCAGCCAGGCTAGTGCAGTCTGCTTTGTGCCAACACTGGGGTGATGACTGCCCTAGTCTAGCTGAAGCTTTTCCC |
| hsa-miR-548t-3p | AAAAACCACAATTACTTTTGCACCA | CTTTATTAGTCTGGTGCAAAAGAAACTGTGGTTTTTGCCATTACTTTTACAGGCAAAAACCACAATTACTTTTGCACCAACCTAATATAACTTGTTT |
| hsa-miR-154-3p | AATCATACACGGTTGACCTATT | GTGGTACTTGAAGATAGGTTATCCGTGTTGCCTTCGCTTTATTTGTGACGAATCATACACGGTTGACCTATTTTTCAGTACCAA |
| hsa-miR-122-3p | AACGCCATTATCACACTAAATA | CCTTAGCAGAGCTGTGGAGTGTGACAATGGTGTTTGTGTCTAAACTATCAAACGCCATTATCACACTAAATAGCTACTGCTAGGC |
| hsa-miR-1270 | CTGGAGATATGGAAGAGCTGTGT | CACAGAGTTATACTGGAGATATGGAAGAGCTGTGTTGGGTATAAGTAACAGGCTTTTCTTTATCTTCTATGTGGCTCTTTGCA |
| hsa-miR-769-3p | CTGGGATCTCCGGGGTCTTGGTT | GCCTTGGTGCTGATTCCTGGGCTCTGACCTGAGACCTCTGGGTTCTGAGCTGTGATGTTGCTCTCGAGCTGGGATCTCCGGGGTCTTGGTTCAGGGCCGGGGCCTCTGGGTTCCAAGC |
| hsa-miR-143-3p | TGAGATGAAGCACTGTAGCTC | GCGCAGCGCCCTGTCTCCCAGCCTGAGGTGCAGTGCTGCATCTCTGGTCAGTTGGGAGTCTGAGATGAAGCACTGTAGCTCAGGAAGAGAGAAGTTGTTCTGCAGC |
| hsa-miR-6874-5p | ATGGAGCTGGAACCAGATCAGGC | GCCACATGGAGCTGGAACCAGATCAGGCTTTAATGTTTGAAGTAATGTCAGTTCTGCTGTTCTGACTCTAG |
| hsa-miR-10b-5p | TACCCTGTAGAACCGAATTTGTG | CCAGAGGTTGTAACGTTGTCTATATATACCCTGTAGAACCGAATTTGTGTGGTATCCGTATAGTCACAGATTCGATTCTAGGGGAATATATGGTCGATGCAAAAACTTCA |
| hsa-miR-4677-5p | TTGTTCTTTGGTCTTTCAGCCA | GCAAAGCAGCAATTGTTCTTTGGTCTTTCAGCCATGACCTGACCTTCTGTCTGTGAGACCAAAGAACTACTTTGCTTGGC |
| hsa-miR-7705 | AATAGCTCAGAATGTCAGTTCTG | AATAGCTCAGAATGTCAGTTCTGTTTTAAGTAACAGAATTGATAACTGAGCAAGGAA |
| hsa-miR-6755-5p | TAGGGTAGACACTGACAACGTT | TGTTTTAGGGTAGACACTGACAACGTTATGTGTGGTCTTTAACCTGTTGTCATGTTTTTTCCCTAG |
| hsa-miR-512-3p | AAGTGCTGTCATAGCTGAGGTC | TCTCAGTCTGTGGCACTCAGCCTTGAGGGCACTTTCTGGTGCCAGAATGAAAGTGCTGTCATAGCTGAGGTCCAATGACTGAGG |
| hsa-miR-153-3p | TTGCATAGTCACAAAAGTGATC | CTCACAGCTGCCAGTGTCATTTTTGTGATCTGCAGCTAGTATTCTCACTCCAGTTGCATAGTCACAAAAGTGATCATTGGCAGGTGTGGC |
| hsa-miR-4454 | GGATCCGAGTCACGGCACCA | CCGGATCCGAGTCACGGCACCAAATTTCATGCGTGTCCGTGTGAAGAGACCACCA |
| hsa-miR-7110-3p | TCTCTCTCCCACTTCCCTGCAG | GGGGCTGGGGGTGTGGGGAGAGAGAGTGCACAGCCAGCTCAGGGATTAAAGCTCTTTCTCTCTCTCTCTCTCCCACTTCCCTGCAG |
| hsa-miR-423-3p | AGCTCGGTCTGAGGCCCCTCAGT | ATAAAGGAAGTTAGGCTGAGGGGCAGAGAGCGAGACTTTTCTATTTTCCAAAAGCTCGGTCTGAGGCCCCTCAGTCTTGCTTCCTAACCCGCGC |
| hsa-miR-331-5p | CTAGGTATGGTCCCAGGGATCC | GAGTTTGGTTTTGTTTGGGTTTGTTCTAGGTATGGTCCCAGGGATCCCAGATCAAACCAGGCCCCTGGGCCTATCCTAGAACCAACCTAAGCTC |
| hsa-miR-190a-3p | CTATATATCAAACATATTCCT | TGCAGGCCTCTGTGTGATATGTTTGATATATTAGGTTGTTATTTAATCCAACTATATATCAAACATATTCCTACAGTGTCTTGCC |
| hsa-miR-610 | TGAGCTAAATGTGTGCTGGGA | TCTATTTGTCTTAGGTGAGCTAAATGTGTGCTGGGACACATTTGAGCCAAATGTCCCAGCACACATTTAGCTCACATAAGAAAAATGGACTCTAGT |
| hsa-miR-4536-5p | TGTGGTAGATATATGCACGAT | ATGTGGTAGATATATGCACGATATATATACTGCCCTGCTTTTATACATACATACATACATACCTATATCGTGCATATATCTACCACAT |
| hsa-miR-5692b | AATAATATCACAGTAGGTGT | GATATTATGAATAATATCACAGTAGGTGTTCACACATAATGTGTACACCATGTGTGTACACCCATGTGATATTTGAAGTAGTATGTC |
| hsa-miR-6742-5p | AGTGGGGTGGGACCCAGCTGTT | GAGGGAGTGGGGTGGGACCCAGCTGTTGGCCATGGCGACAACACCTGGGTTGTCCCCTCTAG |
| hsa-miR-935 | CCAGTTACCGCTTCCGCTACCGC | GGCGGGGGCGCGGGCGGCAGTGGCGGGAGCGGCCCCTCGGCCATCCTCCGTCTGCCCAGTTACCGCTTCCGCTACCGCCGCCGCTCCCGCT |
| hsa-miR-6774-3p | TCGTGTCCCTCTTGTCCACAG | TGTGCACTTGGGCAGGAGGGACCCTGTATGTCTCCCCGCAGCACCGTCATCGTGTCCCTCTTGTCCACAG |
| hsa-miR-150-3p | CTGGTACAGGCCTGGGGGACAG | CTCCCCATGGCCCTGTCTCCCAACCCTTGTACCAGTGCTGGGCTCAGACCCTGGTACAGGCCTGGGGGACAGGGACCTGGGGAC |
| hsa-miR-548aq-5p | GAAAGTAATTGCTGTTTTTGCC | GAAAGTAATTGCTGTTTTTGCCATTACTTTCAGTGGCAAAAACTGCAATTACTTTTGC |
| hsa-miR-3130-5p | TACCCAGTCTCCGGTGCAGCC | CTTGTCATGTCTTACCCAGTCTCCGGTGCAGCCTGTTGTCAAGGCTGCACCGGAGACTGGGTAAGACATGACAAG |
| hsa-miR-6883-3p | TTCCCTATCTCACTCTCCTCAG | CAGACAGGGAGGGTGTGGTATGGATGTGTTGACCCCTGAAGTGGTTCTGATGACCTTTCCCTATCTCACTCTCCTCAG |
| hsa-miR-497-3p | CAAACCACACTGTGGTGTTAGA | CCACCCCGGTCCTGCTCCCGCCCCAGCAGCACACTGTGGTTTGTACGGCACTGTGGCCACGTCCAAACCACACTGTGGTGTTAGAGCGAGGGTGGGGGAGGCACCGCCGAGG |
| hsa-miR-6818-5p | TTGTGTGAGTACAGAGAGCATC | CTATTTTGTGTGAGTACAGAGAGCATCTGAATGGGTACAGTTGTTGTCTCTTGTTCCTCACACAG |
| hsa-miR-6506-5p | ACTGGGATGTCACTGAATATGGT | GACTGGGATGTCACTGAATATGGTGTTTGTGAGTTGATTGACATCGTATCAGAGATTCCAGACACA |
| hsa-miR-676-3p | CTGTCCTAAGGTTGTTGAGTT | GCATGACTCTTCAACCTCAGGACTTGCAGAATTAATGGAATGCTGTCCTAAGGTTGTTGAGTTGTGC |
| hsa-miR-6508-3p | TGGGCCATGCATTTCTAGAACT | TTCCTCTAGAAATGCATGACCCACCCTGAGTTTTGGTGGGCCATGCATTTCTAGAACTCC |
| hsa-miR-3181 | ATCGGGCCCTCGGCGCCGG | CGGCGACCATCGGGCCCTCGGCGCCGGCCCGTTAGTTGCCCGGGCCCGAGCCGGCCGGGCCCGCGGGTTGCCG |
| hsa-miR-3927-3p | CAGGTAGATATTTGATAGGCAT | TGCCAATGCCTATCACATATCTGCCTGTCCTATGACAAACATGGCAGGTAGATATTTGATAGGCATTGGCA |
| hsa-miR-5193 | TCCTCCTCTACCTCATCCCAGT | CCTAGGAAAGGCTGCTGGTAACTGGGATGGGGGTTGGGGGGAGGTAAGAAGTCTCTGACTCCTCCTCTACCTCATCCCAGTTCCATCACCTGAAGTGGACCTCTTGGGA |
| hsa-miR-6781-3p | TGCCTCTTTTCCACGGCCTCAG | AACCCCGGGCCGGAGGTCAAGGGCGTCGCTTCTCCCTAATGTTGCCTCTTTTCCACGGCCTCAG |
| hsa-miR-376b-3p | ATCATAGAGGAAAATCCATGTT | CAGTCCTTCTTTGGTATTTAAAACGTGGATATTCCTTCTATGTTTACGTGATTCCTGGTTAATCATAGAGGAAAATCCATGTTTTCAGTATCAAATGCTG |
| hsa-miR-371a-5p | ACTCAAACTGTGGGGGCACT | GTGGCACTCAAACTGTGGGGGCACTTTCTGCTCTCTGGTGAAAGTGCCGCCATCTTTTGAGTGTTAC |
| hsa-miR-651-5p | TTTAGGATAAGCTTGACTTTTG | AATCTATCACTGCTTTTTAGGATAAGCTTGACTTTTGTTCAAATAAAAATGCAAAAGGAAAGTGTATCCTAAAAGGCAATGACAGTTTAATGTGTTT |
| hsa-miR-548e-5p | CAAAAGCAATCGCGGTTTTTGC | TTATTAGGTTGGTACAAAAGCAATCGCGGTTTTTGCTATTACTTTTAAAGGCAAAAACTGAGACTACTTTTGCACCAACCTGATAGAA |
| hsa-miR-422a | ACTGGACTTAGGGTCAGAAGGC | GAGAGAAGCACTGGACTTAGGGTCAGAAGGCCTGAGTCTCTCTGCTGCAGATGGGCTCTCTGTCCCTGAGCCAAGCTTTGTCCTCCCTGG |
| hsa-miR-4642 | ATGGCATCGTCCCCTGGTGGCT | CACAACTGCATGGCATCGTCCCCTGGTGGCTGTGGCCTAGGGCAAGCCACAAAGCCACTCAGTGATGATGCCAGCAGTTGTG |
| hsa-miR-4724-3p | GTACCTTCTGGTTCAGCTAGT | ACGCAAAATGAACTGAACCAGGAGTGAGCTTCGTGTACATTATCTATTAGAAAATGAAGTACCTTCTGGTTCAGCTAGTCCCTGTGCGT |
| hsa-miR-4524a-3p | TGAGACAGGCTTATGCTGCTAT | GAACGATAGCAGCATGAACCTGTCTCACTGCAGAATTATTTTGAGACAGGCTTATGCTGCTATCCTTCA |
| hsa-miR-873-3p | GGAGACTGATGAGTTCCCGGGA | GTGTGCATTTGCAGGAACTTGTGAGTCTCCTATTGAAAATGAACAGGAGACTGATGAGTTCCCGGGAACACCCACAA |
| hsa-miR-362-3p | AACACACCTATTCAAGGATTCA | CTTGAATCCTTGGAACCTAGGTGTGAGTGCTATTTCAGTGCAACACACCTATTCAAGGATTCAAA |
| hsa-miR-4758-3p | TGCCCCACCTGCTGACCACCCTC | GGTGAGTGGGAGCCGGTGGGGCTGGAGTAAGGGCACGCCCGGGGCTGCCCCACCTGCTGACCACCCTCCCC |
| hsa-miR-1263 | ATGGTACCCTGGCATACTGAGT | CTACCCCAAAATATGGTACCCTGGCATACTGAGTATTTTAATACTGGCATACTCAGTATGCCATGTTGCCATATTTTGGGGTAGCA |
| hsa-miR-6512-3p | TTCCAGCCCTTCTAATGGTAGG | TATGCTCTTACCATTAGAAGAGCTGGAAGAAGGCTGAGGGAGATGCCTTCTTCCAGCCCTTCTAATGGTAGGAGCAT |
| hsa-miR-1257 | AGTGAATGATGGGTTCTGACC | GCCCTGGGCTTGTGCTTGGGGAGTGAATGATGGGTTCTGACCCCCATGCACCCCTGTGGGCCCCTGGCATCACTGGCCCCATCCTTCACCCCTGCCAACCACGCTTGCCCTGTGCCT |
| hsa-miR-3911 | TGTGTGGATCCTGGAGGAGGCA | GGGTGAGGATGTGTGTGGATCCTGGAGGAGGCAGAGAAGACAGTGAGCTTGCCAGTTCTGGTTTCCAACACTTCCTTTCCTGCGCTTCTCGATTCCCAGATCTGCACCC |
| hsa-miR-4429 | AAAAGCTGGGCTGAGAGGCG | AGGGAGAAAAGCTGGGCTGAGAGGCGACTGGTGTCTAATTTGTTTGTCTCTCCAACTCAGACTGCCTGGCCCA |
| hsa-miR-4711-5p | TGCATCAGGCCAGAAGACATGAG | AAATGTGCATCAGGCCAGAAGACATGAGCCCTTTGGAAAGGTCTCGTGTCTTCTGGCTTGATGCACATTT |
| hsa-miR-4667-5p | ACTGGGGAGCAGAAGGAGAACC | TGACTGGGGAGCAGAAGGAGAACCCAAGAAAAGCTGACTTGGAGGTCCCTCCTTCTGTCCCCACAG |
| hsa-miR-146b-5p | TGAGAACTGAATTCCATAGGCTG | CCTGGCACTGAGAACTGAATTCCATAGGCTGTGAGCTCTAGCAATGCCCTGTGGACTCAGTTCTGGTGCCCGG |
| hsa-miR-378a-3p | ACTGGACTTGGAGTCAGAAGGC | AGGGCTCCTGACTCCAGGTCCTGTGTGTTACCTAGAAATAGCACTGGACTTGGAGTCAGAAGGCCT |
| hsa-miR-133b | TTTGGTCCCCTTCAACCAGCTA | CCTCAGAAGAAAGATGCCCCCTGCTCTGGCTGGTCAAACGGAACCAAGTCCGTCTTCCTGAGAGGTTTGGTCCCCTTCAACCAGCTACAGCAGGGCTGGCAATGCCCAGTCCTTGGAGA |
| hsa-miR-3663-5p | GCTGGTCTGCGTGGTGCTCGG | CCCGGGACCTTGGTCCAGGCGCTGGTCTGCGTGGTGCTCGGGTGGATAAGTCTGATCTGAGCACCACACAGGCCGGGCGCCGGGACCAAGGGGGCTC |
| hsa-miR-548ah-3p | CAAAAACTGCAGTTACTTTTGC | AGGTTGGTGCAAAAGTGATTGCAGTGTTTGCCAATAAAAGTAATGACAAAAACTGCAGTTACTTTTGCACCAGCCC |
| hsa-miR-410-5p | AGGTTGTCTGTGATGAGTTCG | GGTACCTGAGAAGAGGTTGTCTGTGATGAGTTCGCTTTTATTAATGACGAATATAACACAGATGGCCTGTTTTCAGTACC |
| hsa-miR-4728-3p | CATGCTGACCTCCCTCCTGCCCCAG | GTGGGAGGGGAGAGGCAGCAAGCACACAGGGCCTGGGACTAGCATGCTGACCTCCCTCCTGCCCCAG |
| hsa-miR-15b-5p | TAGCAGCACATCATGGTTTACA | TTGAGGCCTTAAAGTACTGTAGCAGCACATCATGGTTTACATGCTACAGTCAAGATGCGAATCATTATTTGCTGCTCTAGAAATTTAAGGAAATTCAT |
| hsa-miR-572 | GTCCGCTCGGCGGTGGCCCA | GTCGAGGCCGTGGCCCGGAAGTGGTCGGGGCCGCTGCGGGCGGAAGGGCGCCTGTGCTTCGTCCGCTCGGCGGTGGCCCAGCCAGGCCCGCGGGA |
| hsa-miR-548q | GCTGGTGCAAAAGTAATGGCGG | ATATTAGGCTGGTGCAAAAGTAATGGCGGTTTTTGCCATTACTTTTCATTTTTACCATTAAAAGTAATGGCAAAAAGCATGATTACTTTTTCACCAACCT |
| hsa-miR-4659b-5p | TTGCCATGTCTAAGAAGAA | CTGTTGACGTTGCCATGTCTAAGAAGAAAATTTTTCTCCAAAGTTTTCTTCTTAGACATGGCAGCTTCAGCAG |
| hsa-miR-4664-3p | CTTCCGGTCTGTGAGCCCCGTC | GTTGGGGGCTGGGGTGCCCACTCCGCAAGTTATCACTGAGCGACTTCCGGTCTGTGAGCCCCGTCCTCCGC |
| hsa-miR-4478 | GAGGCTGAGCTGAGGAG | GGCCGAGGCTGAGCTGAGGAGCCTCCAAACCTGTAGACAGGGTCATGCAGTACTAGGGGCGAGCCTCATCCCCTGCAGCCCTGGCC |
| hsa-miR-4485-3p | TAACGGCCGCGGTACCCTAA | AGAGGCACCGCCTGCCCAGTGACATGCGTTTAACGGCCGCGGTACCCTAACTGTGCA |
| hsa-miR-3648 | AGCCGCGGGGATCGCCGAGGG | CGCGACTGCGGCGGCGGTGGTGGGGGGAGCCGCGGGGATCGCCGAGGGCCGGTCGGCCGCCCCGGGTGCCGCGCGGTGCCGCCGGCGGCGGTGAGGCCCCGCGCGTGTGTCCCGGCTGCGGTCGGCCGCGCTCGAGGGGTCCCCGTGGCGTCCCCTTCCCCGCCGGCCGCCTTTCTCGCG |
| hsa-miR-10399-5p | AATTACAGATTGTCTCAGAGA | AATTACAGATTGTCTCAGAGAAAACAAATGAGTTACTCTCTCGGACAAGCTGTAGGTC |
| hsa-miR-937-5p | GTGAGTCAGGGTGGGGCTGG | AGCACTGCCCCCGGTGAGTCAGGGTGGGGCTGGCCCCCTGCTTCGTGCCCATCCGCGCTCTGACTCTCTGCCCACCTGCAGGAGCT |
| hsa-miR-181d-3p | CCACCGGGGGATGAATGTCAC | GTCCCCTCCCCTAGGCCACAGCCGAGGTCACAATCAACATTCATTGTTGTCGGTGGGTTGTGAGGACTGAGGCCAGACCCACCGGGGGATGAATGTCACTGTGGCTGGGCCAGACACGGCTTAAGGGGAATGGGGAC |
| hsa-miR-128-3p | TCACAGTGAACCGGTCTCTTT | TGAGCTGTTGGATTCGGGGCCGTAGCACTGTCTGAGAGGTTTACATTTCTCACAGTGAACCGGTCTCTTTTTCAGCTGCTTC |
| hsa-miR-4756-5p | CAGGGAGGCGCTCACTCTCTGCT | GGGATAAAATGCAGGGAGGCGCTCACTCTCTGCTGCCGATTCTGCACCAGAGATGGTTGCCTTCCTATATTTTGTGTC |
| hsa-miR-3158-5p | CCTGCAGAGAGGAAGCCCTTC | ATTCAGGCCGGTCCTGCAGAGAGGAAGCCCTTCTGCTTACAGGTATTGGAAGGGCTTCCTCTCTGCAGGACCGGCCTGAAT |
| hsa-miR-4632-3p | TGCCGCCCTCTCGCTGCTCTAG | GAGGGCAGCGTGGGTGTGGCGGAGGCAGGCGTGACCGTTTGCCGCCCTCTCGCTGCTCTAG |
| hsa-miR-1237-3p | TCCTTCTGCTCCGTCCCCCAG | GTGGGAGGGCCCAGGCGCGGGCAGGGGTGGGGGTGGCAGAGCGCTGTCCCGGGGGCGGGGCCGAAGCGCGGCGACCGTAACTCCTTCTGCTCCGTCCCCCAG |
| hsa-miR-4517 | AAATATGATGAAACTCACAGCTGAG | AGGTAAATATGATGAAACTCACAGCTGAGGAGCTTAGCAAGTAGCTAAGGCCAGAGCTTGTGTTTGGGTGGTGTGGCTG |
| hsa-miR-155-3p | CTCCTACATATTAGCATTAACA | CTGTTAATGCTAATCGTGATAGGGGTTTTTGCCTCCAACTGACTCCTACATATTAGCATTAACAG |
| hsa-miR-1268a | CGGGCGTGGTGGTGGGGG | TAGCCGGGCGTGGTGGTGGGGGCCTGTGGTCCCAGCTACTTTGGAGGCTGAG |
| hsa-miR-4661-3p | CAGGATCCACAGAGCTAGTCCA | TTTACTCTGAACTAGCTCTGTGGATCCTGACAGACAGCCTGATAGACAGGATCCACAGAGCTAGTCCAGAGTAAA |
| hsa-miR-7152-3p | TCTGGTCCTGGACAGGAGGC | TTTCCTGTCCTCCAACCAGACCATGCCACATCCGTCTGGTCCTGGACAGGAGGC |
| hsa-miR-6828-3p | ATCTGCTCTCTTGTTCCCAG | GGCTCAGGAAGCAAGAGAACCCTGTGGTCTAACCTTTCCCATCTGCTCTCTTGTTCCCAG |
| hsa-miR-4444 | CTCGAGTTGGAAGAGGCG | GTGACGACTGGCCCCGCCTCTTCCTCTCGGTCCCATATTGAACTCGAGTTGGAAGAGGCGAGTCCGGTCTCAAA |
| hsa-miR-6838-5p | AAGCAGCAGTGGCAAGACTCCT | CAGGGAAGCAGCAGTGGCAAGACTCCTAGGTCACGGAAGTCCTGCTTCTGTTGCAG |
| hsa-miR-6757-5p | TAGGGATGGGAGGCCAGGATGA | GGGCTTAGGGATGGGAGGCCAGGATGAAGATTAATCCCTAATCCCCAACACTGGCCTTGCTATCCCCAG |
| hsa-miR-501-5p | AATCCTTTGTCCCTGGGTGAGA | GCTCTTCCTCTCTAATCCTTTGTCCCTGGGTGAGAGTGCTTTCTGAATGCAATGCACCCGGGCAAGGATTCTGAGAGGGTGAGC |
| hsa-miR-342-5p | AGGGGTGCTATCTGTGATTGA | GAAACTGGGCTCAAGGTGAGGGGTGCTATCTGTGATTGAGGGACATGGTTAATGGAATTGTCTCACACAGAAATCGCACCCGTCACCTTGGCCTACTTA |
| hsa-miR-6758-5p | TAGAGAGGGGAAGGATGTGATGT | TGGGCTAGAGAGGGGAAGGATGTGATGTGAGCAGATGGTTCTCACTCATTCTCCTCTGTCCAG |
| hsa-miR-1229-3p | CTCTCACCACTGCCCTCCCACAG | GTGGGTAGGGTTTGGGGGAGAGCGTGGGCTGGGGTTCAGGGACACCCTCTCACCACTGCCCTCCCACAG |
| hsa-miR-642a-3p | AGACACATTTGGAGAGGGAACC | ATCTGAGTTGGGAGGGTCCCTCTCCAAATGTGTCTTGGGGTGGGGGATCAAGACACATTTGGAGAGGGAACCTCCCAACTCGGCCTCTGCCATCATT |
| hsa-miR-1284 | TCTATACAGACCCTGGCTTTTC | ATTTTGATATATAAGCCAGTTTAATGTTTTCTATACAGACCCTGGCTTTTCTTAAATTTTATATATTGGAAAGCCCATGTTTGTATTGGAAACTGCTGGTTTCTTTCATACTGAAAATCT |
| hsa-miR-556-5p | GATGAGCTCATTGTAATATGAG | GATAGTAATAAGAAAGATGAGCTCATTGTAATATGAGCTTCATTTATACATTTCATATTACCATTAGCTCATCTTTTTTATTACTACCTTCAACA |
| hsa-miR-4705 | TCAATCACTTGGTAATTGCTGT | CTCACAAGATCAATCACTTGGTAATTGCTGTGATAACAACTCAGCAATTACCAAGTGATTGGTTTTGTGAG |
| hsa-miR-519c-5p | CTCTAGAGGGAAGCGCTTTCTG | CTCAGGCTGTGACACTCTAGAGGGAAGCGCTTTCTGTTGTCTGAAAGAAAGGAAAGTGCATCCTTTTAGAGTGTTACTGTTTGAG |
| hsa-miR-5094 | AATCAGTGAATGCCTTGAACCT | AAAAGAAAAAAATCAGTGAATGCCTTGAACCTAACACACTGCCTTTTATGTGGTAGGTACAGTGGGCTCACTGAAACATTCAACT |
| hsa-miR-145-3p | GGATTCCTGGAAATACTGTTCT | CACCTTGTCCTCACGGTCCAGTTTTCCCAGGAATCCCTTAGATGCTAAGATGGGGATTCCTGGAAATACTGTTCTTGAGGTCATGGTT |
| hsa-miR-2116-5p | GGTTCTTAGCATAGGAGGTCT | GACCTAGGCTAGGGGTTCTTAGCATAGGAGGTCTTCCCATGCTAAGAAGTCCTCCCATGCCAAGAACTCCCAGACTAGGA |
| hsa-miR-4741 | CGGGCTGTCCGGAGGGGTCGGCT | CGGGCGGGGCGGGTCCGGCCGCCTCCGAGCCCGGCCGGCAGCCCCCGGCCTTAAAGCGCGGGCTGTCCGGAGGGGTCGGCTTTCCCACCG |
| hsa-miR-548t-5p | CAAAAGTGATCGTGGTTTTTG | AGGGTGGTGCAAAAGTGATCGTGGTTTTTGCAATTTTTTAATGACAAAAACCACAATTACTTTTGCACCAACCT |
| hsa-miR-29c-3p | TAGCACCATTTGAAATCGGTTA | ATCTCTTACACAGGCTGACCGATTTCTCCTGGTGTTCAGAGTCTGTTTTTGTCTAGCACCATTTGAAATCGGTTATGATGTAGGGGGA |
| hsa-miR-483-5p | AAGACGGGAGGAAAGAAGGGAG | GAGGGGGAAGACGGGAGGAAAGAAGGGAGTGGTTCCATCACGCCTCCTCACTCCTCTCCTCCCGTCTTCTCCTCTC |
| hsa-let-7f-5p | TGAGGTAGTAGATTGTATAGTT | TCAGAGTGAGGTAGTAGATTGTATAGTTGTGGGGTAGTGATTTTACCCTGTTCAGGAGATAACTATACAATCTATTGCCTTCCCTGA |
| hsa-miR-1539 | TCCTGCGCGTCCCAGATGCCC | GGCTCTGCGGCCTGCAGGTAGCGCGAAAGTCCTGCGCGTCCCAGATGCCC |
| hsa-miR-6842-5p | TGGGGGTGGTCTCTAGCCAAGG | AGCCCTGGGGGTGGTCTCTAGCCAAGGCTCTGGGGTCTCACCCTTGGCTGGTCTCTGCTCCGCAG |
| hsa-miR-7111-5p | TGGGGGAGGAAGGACAGGCCAT | CTGGGGGAGGAAGGACAGGCCATCTGCTATTCGTCCACCAACCTGACTTGATCCTCTCTTCCCTCCTCCCAG |
| hsa-let-7d-5p | AGAGGTAGTAGGTTGCATAGTT | CCTAGGAAGAGGTAGTAGGTTGCATAGTTTTAGGGCAGGGATTTTGCCCACAAGGAGGTAACTATACGACCTGCTGCCTTTCTTAGG |
| hsa-miR-10526-3p | AAAAGGGGGCTGAGGTGGAG | TCCCCTTAGTTTCCTTTAAGAGTGATAAAAATGGAAAAGGGGGCTGAGGTGGAG |
| hsa-miR-30e-5p | TGTAAACATCCTTGACTGGAAG | GGGCAGTCTTTGCTACTGTAAACATCCTTGACTGGAAGCTGTAAGGTGTTCAGAGGAGCTTTCAGTCGGATGTTTACAGCGGCAGGCTGCCA |
| hsa-miR-1271-5p | CTTGGCACCTAGCAAGCACTCA | CACCCAGATCAGTGCTTGGCACCTAGCAAGCACTCAGTAAATATTTGTTGAGTGCCTGCTATGTGCCAGGCATTGTGCTGAGGGCT |
| hsa-miR-18a-3p | ACTGCCCTAAGTGCTCCTTCTGG | TGTTCTAAGGTGCATCTAGTGCAGATAGTGAAGTAGATTAGCATCTACTGCCCTAAGTGCTCCTTCTGGCA |
| hsa-miR-378b | ACTGGACTTGGAGGCAGAA | GGTCATTGAGTCTTCAAGGCTAGTGGAAAGAGCACTGGACTTGGAGGCAGAAAGACC |
| hsa-miR-6820-3p | TGTGACTTCTCCCCTGCCACAG | CCTTCTGCGGCAGAGCTGGGGTCACCAGCCCTCATGTACTTGTGACTTCTCCCCTGCCACAG |
| hsa-miR-369-5p | AGATCGACCGTGTTATATTCGC | TTGAAGGGAGATCGACCGTGTTATATTCGCTTTATTGACTTCGAATAATACATGGTTGATCTTTTCTCAG |
| hsa-miR-4745-3p | TGGCCCGGCGACGTCTCACGGTC | GTGAGTGGGGCTCCCGGGACGGCGCCCGCCCTGGCCCTGGCCCGGCGACGTCTCACGGTCCC |
| hsa-miR-4725-5p | AGACCCTGCAGCCTTCCCACC | GTGTCTCTCTGGAGACCCTGCAGCCTTCCCACCCACCAGGGAGCTTTCCATGGGCTGTGGGGAAGGCGTCAGTGTCGGGTGAGGGAACAC |
| hsa-miR-6862-5p | CGGGCATGCTGGGAGAGACTTT | CGAAGCGGGCATGCTGGGAGAGACTTTGTGATTTGTCTCCAAAGCCTCACCCAGCTCTCTGGCCCTCTAG |
| hsa-miR-654-5p | TGGTGGGCCGCAGAACATGTGC | GGGTAAGTGGAAAGATGGTGGGCCGCAGAACATGTGCTGAGTTCGTGCCATATGTCTGCTGACCATCACCTTTAGAAGCCC |
| hsa-miR-99a-3p | CAAGCTCGCTTCTATGGGTCTG | CCCATTGGCATAAACCCGTAGATCCGATCTTGTGGTGAAGTGGACCGCACAAGCTCGCTTCTATGGGTCTGTGTCAGTGTG |
| hsa-miR-10398-3p | GCCCGGAGAGCTGGGAGCCAG | TGGCTCCCTTCTCTCCGTCTGCCTCCTGGCCGCGGGGCCCGGAGAGCTGGGAGCCAG |
| hsa-miR-130b-5p | ACTCTTTCCCTGTTGCACTAC | GGCCTGCCCGACACTCTTTCCCTGTTGCACTACTATAGGCCGCTGGGAAGCAGTGCAATGATGAAAGGGCATCGGTCAGGTC |
| hsa-miR-487a-5p | GTGGTTATCCCTGCTGTGTTCG | GGTACTTGAAGAGTGGTTATCCCTGCTGTGTTCGCTTAATTTATGACGAATCATACAGGGACATCCAGTTTTTCAGTATC |
| hsa-miR-6799-3p | TGCCCTGCATGGTGTCCCCACAG | GAGGAGGGGAGGTGTGCAGGGCTGGGGTCACTGACTCTGCTTCCCCTGCCCTGCATGGTGTCCCCACAG |
| hsa-miR-3064-3p | TTGCCACACTGCAACACCTTACA | GGTCTGGCTGTTGTGGTGTGCAAAACTCCGTACATTGCTATTTTGCCACACTGCAACACCTTACAG |
| hsa-miR-6726-3p | CTCGCCCTGTCTCCCGCTAG | GGGGGCGGGAGCTGGGGTCTGCAGGTTCGCACTGATGCCTGCTCGCCCTGTCTCCCGCTAG |
| hsa-miR-939-5p | TGGGGAGCTGAGGCTCTGGGGGTG | TGTGGGCAGGGCCCTGGGGAGCTGAGGCTCTGGGGGTGGCCGGGGCTGACCCTGGGCCTCTGCTCCCCAGTGTCTGACCGCG |
| hsa-miR-485-5p | AGAGGCTGGCCGTGATGAATTC | ACTTGGAGAGAGGCTGGCCGTGATGAATTCGATTCATCAAAGCGAGTCATACACGGCTCTCCTCTCTTTTAGT |
| hsa-miR-9985 | TTCACAGTGGCTAAGCTAT | CCCATGTTCATTGAGCTTTATTCACAGTGGCTAAGCTATGGAATCCATCCAAGTGTTCACTGATGGATAAATGGATACAGAAAATGTTTATATATATATATATAATATT |
| hsa-miR-6880-3p | CCGCCTTCTCTCCTCCCCCAG | GAGGGTGGTGGAGGAAGAGGGCAGCTCCCATGACTGCCTGACCGCCTTCTCTCCTCCCCCAG |
| hsa-miR-144-5p | GGATATCATCATATACTGTAAG | TGGGGCCCTGGCTGGGATATCATCATATACTGTAAGTTTGCGATGAGACACTACAGTATAGATGATGTACTAGTCCGGGCACCCCC |
| hsa-miR-122-5p | TGGAGTGTGACAATGGTGTTTG | CCTTAGCAGAGCTGTGGAGTGTGACAATGGTGTTTGTGTCTAAACTATCAAACGCCATTATCACACTAAATAGCTACTGCTAGGC |
| hsa-miR-206 | TGGAATGTAAGGAAGTGTGTGG | TGCTTCCCGAGGCCACATGCTTCTTTATATCCCCATATGGATTACTTTGCTATGGAATGTAAGGAAGTGTGTGGTTTCGGCAAGTG |
| hsa-miR-548f-5p | TGCAAAAGTAATCACAGTTTTT | ATTAGGTTGGTGCAAAAGTAATCACAGTTTTTGACATTACTTTCAAAGACAAAAACTGTAATTACTTTTGGACCAACCTAATAG |
| hsa-miR-136-5p | ACTCCATTTGTTTTGATGATGGA | TGAGCCCTCGGAGGACTCCATTTGTTTTGATGATGGATTCTTATGCTCCATCATCGTCTCAAATGAGTCTTCAGAGGGTTCT |
| hsa-miR-4715-5p | AAGTTGGCTGCAGTTAAGGTGG | GGGGAATGAAAGTTGGCTGCAGTTAAGGTGGCTAATCAGCTGATGGTGCCACCTTAACTGCAGCCAATTCTAATTCCCC |
| hsa-miR-6810-3p | TCCCCTGCTCCCTTGTTCCCCAG | CTGGGATGGGGACAGGGATCAGCATGGCACAGATCCAATACCTTCTGTCCCCTGCTCCCTTGTTCCCCAG |
| hsa-miR-1972 | TCAGGCCAGGCACAGTGGCTCA | TATAGGCATGTGCCACCACACCTGGCTTAAATGTGTCATTTAAAAATTCAGGCCAGGCACAGTGGCTCATGCCTGTA |
| hsa-miR-340-5p | TTATAAAGCAATGAGACTGATT | TTGTACCTGGTGTGATTATAAAGCAATGAGACTGATTGTCATATGTCGTTTGTGGGATCCGTCTCAGTTACTTTATAGCCATACCTGGTATCTTA |
| hsa-miR-3120-5p | CCTGTCTGTGCCTGCTGTACA | GTCATGTGACTGCCTGTCTGTGCCTGCTGTACAGGTGAGCGGATGTTCTGCACAGCAAGTGTAGACAGGCAGACACATGAC |
| hsa-miR-485-3p | GTCATACACGGCTCTCCTCTCT | ACTTGGAGAGAGGCTGGCCGTGATGAATTCGATTCATCAAAGCGAGTCATACACGGCTCTCCTCTCTTTTAGT |
| hsa-miR-632 | GTGTCTGCTTCCTGTGGGA | CGCCTCCTACCGCAGTGCTTGACGGGAGGCGGAGCGGGGAACGAGGCCGTCGGCCATTTTGTGTCTGCTTCCTGTGGGACGTGGTGGTAGCCGT |
| hsa-miR-25-3p | CATTGCACTTGTCTCGGTCTGA | GGCCAGTGTTGAGAGGCGGAGACTTGGGCAATTGCTGGACGCTGCCCTGGGCATTGCACTTGTCTCGGTCTGACAGTGCCGGCC |
| hsa-miR-4684-3p | TGTTGCAAGTCGGTGGAGACGT | GCACCAGGGGTACCTCTCTACTGACTTGCAACATACATTTGTCTTGGTGTGTTGCAAGTCGGTGGAGACGTACCCTTGGTGC |
| hsa-miR-6728-5p | TTGGGATGGTAGGACCAGAGGGG | CTAGATTGGGATGGTAGGACCAGAGGGGCTTACTGCCCTGTGGGGCTCTCTGGACCCAGTGCCATGCTTCTCTGCTCTGCTCTCCCCAG |
| hsa-miR-147b-5p | TGGAAACATTTCTGCACAAACT | TATAAATCTAGTGGAAACATTTCTGCACAAACTAGATTCTGGACACCAGTGTGCGGAAATGCTTCTGCTACATTTTTAGG |
| hsa-miR-3688-3p | TATGGAAAGACTTTGCCACTCT | TCTTCACTTTCAAGAGTGGCAAAGTCTTTCCATATGTATGTATGTATGTCTGTTACACATATGGAAAGACTTTGCCACTCTTTAAAGTGAAGA |
| hsa-miR-6851-5p | AGGAGGTGGTACTAGGGGCCAGC | CAGGGAGGAGGTGGTACTAGGGGCCAGCAACCTGATTACCCCTCTTTGGCCCTTTGTACCCCTCCAG |
| hsa-miR-455-3p | GCAGTCCATGGGCATATACAC | TCCCTGGCGTGAGGGTATGTGCCTTTGGACTACATCGTGGAAGCCAGCACCATGCAGTCCATGGGCATATACACTTGCCTCAAGGCCTATGTCATC |
| hsa-miR-425-5p | AATGACACGATCACTCCCGTTGA | GAAAGCGCTTTGGAATGACACGATCACTCCCGTTGAGTGGGCACCCGAGAAGCCATCGGGAATGTCGTGTCCGCCCAGTGCTCTTTC |
| hsa-miR-181c-5p | AACATTCAACCTGTCGGTGAGT | CGGAAAATTTGCCAAGGGTTTGGGGGAACATTCAACCTGTCGGTGAGTTTGGGCAGCTCAGGCAAACCATCGACCGTTGAGTGGACCCTGAGGCCTGGAATTGCCATCCT |
| hsa-miR-6717-5p | AGGCGATGTGGGGATGTAGAGA | CTGGTGTTTGAGGCGATGTGGGGATGTAGAGACAACTTCCCAGTCTCATTTCCTCATCCTGCCAGGCCACCAT |
| hsa-miR-5685 | ACAGCCCAGCAGTTATCACGGG | CTCTACATCACAGCCCAGCAGTTATCACGGGCCCCTCCCCTCAATGGGCCCGTGATAACTGCAGGGCTGTGATGTAGAG |
| hsa-miR-338-3p | TCCAGCATCAGTGATTTTGTTG | TCTCCAACAATATCCTGGTGCTGAGTGATGACTCAGGCGACTCCAGCATCAGTGATTTTGTTGAAGA |
| hsa-miR-499b-5p | ACAGACTTGCTGTGATGTTCA | GGAAGCAGCACAGACTTGCTGTGATGTTCACGTGGAGAGGAGTTAAACATCACTGCAAGTCTTAACAGCCGCC |
| hsa-miR-211-5p | TTCCCTTTGTCATCCTTCGCCT | TCACCTGGCCATGTGACTTGTGGGCTTCCCTTTGTCATCCTTCGCCTAGGGCTCTGAGCAGGGCAGGGACAGCAAAGGGGTGCTCAGTTGTCACTTCCCACAGCACGGAG |
| hsa-miR-153-5p | TCATTTTTGTGATGTTGCAGCT | AGCGGTGGCCAGTGTCATTTTTGTGATGTTGCAGCTAGTAATATGAGCCCAGTTGCATAGTCACAAAAGTGATCATTGGAAACTGTG |
| hsa-miR-767-5p | TGCACCATGGTTGTCTGAGCATG | GCTTTTATATTGTAGGTTTTTGCTCATGCACCATGGTTGTCTGAGCATGCAGCATGCTTGTCTGCTCATACCCCATGGTTTCTGAGCAGGAACCTTCATTGTCTACTGC |
| hsa-miR-101-2-5p | TCGGTTATCATGGTACCGATGC | ACTGTCCTTTTTCGGTTATCATGGTACCGATGCTGTATATCTGAAAGGTACAGTACTGTGATAACTGAAGAATGGTGGT |
| hsa-miR-219a-5p | TGATTGTCCAAACGCAATTCT | CCGCCCCGGGCCGCGGCTCCTGATTGTCCAAACGCAATTCTCGAGTCTATGGCTCCGGCCGAGAGTTGAGTCTGGACGTCCCGAGCCGCCGCCCCCAAACCTCGAGCGGG |
| hsa-miR-219a-1-3p | AGAGTTGAGTCTGGACGTCCCG | CCGCCCCGGGCCGCGGCTCCTGATTGTCCAAACGCAATTCTCGAGTCTATGGCTCCGGCCGAGAGTTGAGTCTGGACGTCCCGAGCCGCCGCCCCCAAACCTCGAGCGGG |
| hsa-miR-4436b-5p | GTCCACTTCTGCCTGCCCTGCC | GTGTCCTCACTTGTCCACTTCTGCCTGCCCTGCCCAAATGGTGGAGCAGATTCGAGGGGCAGGGCAGGAAGAAGTGGACAAGTGAGGCCAT |
| hsa-miR-3121-5p | TCCTTTGCCTATTCTATTTAAG | AAATGGTTATGTCCTTTGCCTATTCTATTTAAGACACCCTGTACCTTAAATAGAGTAGGCAAAGGACAGAAACATTT |
| hsa-miR-4523 | GACCGAGAGGGCCTCGGCTGT | GCGGGGGACCGAGAGGGCCTCGGCTGTGTGAGGACTAGAGGCGGCCGAGGCCCGGGCCGGTTCCCCCGA |
| hsa-miR-140-3p | TACCACAGGGTAGAACCACGG | TGTGTCTCTCTCTGTGTCCTGCCAGTGGTTTTACCCTATGGTAGGTTACGTCATGCTGTTCTACCACAGGGTAGAACCACGGACAGGATACCGGGGCACC |
| hsa-miR-3659 | TGAGTGTTGTCTACGAGGGCA | TCTACAAGCAGATACAAGGATGCCCTTGTACACAACACACGTGCTGCTTGTATAGACATGAGTGTTGTCTACGAGGGCATCCTTGTGTCTGTGTGTGTG |
| hsa-miR-3679-3p | CTTCCCCCCAGTAATCTTCATC | CGTGGTGAGGATATGGCAGGGAAGGGGAGTTTCCCTCTATTCCCTTCCCCCCAGTAATCTTCATCATG |
| hsa-miR-4526 | GCTGACAGCAGGGCTGGCCGCT | TGCGGTGACATCAGGGCCCAGTCCCTGCTGTCATGCCCCAGGTGACGTGCTGGGCTGACAGCAGGGCTGGCCGCTAACGTCACTGTC |
| hsa-miR-625-5p | AGGGGGAAAGTTCTATAGTCC | AGGGTAGAGGGATGAGGGGGAAAGTTCTATAGTCCTGTAATTAGATCTCAGGACTATAGAACTTTCCCCCTCATCCCTCTGCCCT |
| hsa-miR-369-3p | AATAATACATGGTTGATCTTT | TTGAAGGGAGATCGACCGTGTTATATTCGCTTTATTGACTTCGAATAATACATGGTTGATCTTTTCTCAG |
| hsa-miR-1287-3p | CTCTAGCCACAGATGCAGTGAT | GTTGTGCTGTCCAGGTGCTGGATCAGTGGTTCGAGTCTGAGCCTTTAAAAGCCACTCTAGCCACAGATGCAGTGATTGGAGCCATGACAA |
| hsa-miR-132-3p | TAACAGTCTACAGCCATGGTCG | CCGCCCCCGCGTCTCCAGGGCAACCGTGGCTTTCGATTGTTACTGTGGGAACTGGAGGTAACAGTCTACAGCCATGGTCGCCCCGCAGCACGCCCACGCGC |
| hsa-miR-6882-5p | TACAAGTCAGGAGCTGAAGCAG | GGCTTTACAAGTCAGGAGCTGAAGCAGCTGGAATTCAAGCCCTGCTGCCTCTCCTCTTGCCTGCAG |
| hsa-miR-1537-5p | AGCTGTAATTAGTCAGTTTTCT | ACAGCTGTAATTAGTCAGTTTTCTGTCCTGTCCACACAGAAAACCGTCTAGTTACAGTTGT |
| hsa-miR-5002-3p | TGACTGCCTCACTGACCACTT | TCTTCCTCTCTGTCCTCTGGAATTTGGTTTCTGAGGCACTTAGTAGGTGATAGCATGACTGACTGCCTCACTGACCACTTCCAGATGAGGGTTACTC |
| hsa-miR-1908-3p | CCGGCCGCCGGCTCCGCCCCG | CGGGAATGCCGCGGCGGGGACGGCGATTGGTCCGTATGTGTGGTGCCACCGGCCGCCGGCTCCGCCCCGGCCCCCGCCCC |
| hsa-miR-6811-3p | AGCCTGTGCTTGTCCCTGCAG | TATGCAGGCCTGTGTACAGCACTCAGGCAGTGCCATGAGCCTGTGCTTGTCCCTGCAG |
| hsa-miR-6514-3p | CTGCCTGTTCTTCCACTCCAG | TATGGAGTGGACTTTCAGCTGGCATTTACGAGTCAGAGTTCTTACAGAGCTGCCTGTTCTTCCACTCCAG |
| hsa-miR-6738-3p | CTTCTGCCTGCATTCTACTCCCAG | GAAGGCGAGGGGTAGAAGAGCACAGGGGTTCTGATAAACCCTTCTGCCTGCATTCTACTCCCAG |
| hsa-miR-548h-5p | AAAAGTAATCGCGGTTTTTGTC | TCTGTCCATTAGGTGGGTGCAAAAGTAATCGCGGTTTTTGTCATTACTTTTAATGGTAAAAACTGGAATTACTTTTGCACTGACCTAATATTAAGCCAGATA |
| hsa-miR-130a-3p | CAGTGCAATGTTAAAAGGGCAT | TGCTGCTGGCCAGAGCTCTTTTCACATTGTGCTACTGTCTGCACCTGTCACTAGCAGTGCAATGTTAAAAGGGCATTGGCCGTGTAGTG |
| hsa-miR-4736 | AGGCAGGTTATCTGGGCTG | AGGCAGGTTATCTGGGCTGCCATCTCCCACTGGCTGCTTGCCTGCCT |
| hsa-miR-5088-3p | TCCCTTCTTCCTGGGCCCTCA | CCCATCAGGGCTCAGGGATTGGATGGAGGTGATGGGGGCAGGGGATGGGTCTCACCCTCCCTTCTTCCTGGGCCCTCAG |
| hsa-miR-142-3p | TGTAGTGTTTCCTACTTTATGGA | GACAGTGCAGTCACCCATAAAGTAGAAAGCACTACTAACAGCACTGGAGGGTGTAGTGTTTCCTACTTTATGGATGAGTGTACTGTG |
| hsa-miR-520d-5p | CTACAAAGGGAAGCCCTTTC | TCTCAAGCTGTGAGTCTACAAAGGGAAGCCCTTTCTGTTGTCTAAAAGAAAAGAAAGTGCTTCTCTTTGGTGGGTTACGGTTTGAGA |
| hsa-miR-1537-3p | AAAACCGTCTAGTTACAGTTGT | ACAGCTGTAATTAGTCAGTTTTCTGTCCTGTCCACACAGAAAACCGTCTAGTTACAGTTGT |
| hsa-miR-9898 | TACTTACCTGTCCCCTACCCCA | GGGGAGGACCCTGAGGGAGGGTGGGAGCACGGGAGAAGAGAAGGCATACCCAACCTGACCTACTTACCTGTCCCCTACCCCACAGAGGGCTTCCCT |
| hsa-miR-5187-5p | TGGGATGAGGGATTGAAGTGGA | GACTAAGGGTGGGATGAGGGATTGAAGTGGAGCAGGAATGCGCTTTTCTCCACTGAATCCTCTTTTCCTCAGGTGG |
| hsa-miR-4646-3p | ATTGTCCCTCTCCCTTCCCAG | ACTGGGAAGAGGAGCTGAGGGACATTGCGGAGAGGGTCTCACATTGTCCCTCTCCCTTCCCAG |
| hsa-miR-3529-5p | AGGTAGACTGGGATTTGTTGTT | GGCACCATTAGGTAGACTGGGATTTGTTGTTGAGCGCAGTAAGACAACAACAAAATCACTAGTCTTCCAGATGGGGCC |
| hsa-miR-6809-3p | CTTCTCTTCTCTCCTTCCCAG | AATGTTGGCAAGGAAAGAAGAGGATCATGTTTGCCCCCGTGGACAGCTCTCTGGTGTGCTCCTCCTGCCATCCTGCCCACCCCTGCATAATGCTGCTTCTCTTCTCTCCTTCCCAG |
| hsa-miR-1-5p | ACATACTTCTTTATATGCCCAT | TGGGAAACATACTTCTTTATATGCCCATATGGACCTGCTAAGCTATGGAATGTAAAGAAGTATGTATCTCA |
| hsa-miR-27a-3p | TTCACAGTGGCTAAGTTCCGC | CTGAGGAGCAGGGCTTAGCTGCTTGTGAGCAGGGTCCACACCAAGTCGTGTTCACAGTGGCTAAGTTCCGCCCCCCAG |
| hsa-miR-671-5p | AGGAAGCCCTGGAGGGGCTGGAG | GCAGGTGAACTGGCAGGCCAGGAAGAGGAGGAAGCCCTGGAGGGGCTGGAGGTGATGGATGTTTTCCTCCGGTTCTCAGGGCTCCACCTCTTTCGGGCCGTAGAGCCAGGGCTGGTGC |
| hsa-miR-1285-5p | GATCTCACTTTGTTGCCCAGG | TGTAGAGATAGGATCTCACTTTGTTGCCCAGGCTGGTCTCAAACTCCTGGTCTGGGCAACAAAGTGAGACCTTATCTCTACAAG |
| hsa-miR-539-5p | GGAGAAATTATCCTTGGTGTGT | ATACTTGAGGAGAAATTATCCTTGGTGTGTTCGCTTTATTTATGATGAATCATACAAGGACAATTTCTTTTTGAGTAT |
| hsa-miR-3124-3p | ACTTTCCTCACTCCCGTGAAGT | GCGGGCTTCGCGGGCGAAGGCAAAGTCGATTTCCAAAAGTGACTTTCCTCACTCCCGTGAAGTCGGC |
| hsa-miR-33b-5p | GTGCATTGCTGTTGCATTGC | GCGGGCGGCCCCGCGGTGCATTGCTGTTGCATTGCACGTGTGTGAGGCGGGTGCAGTGCCTCGGCAGTGCAGCCCGGAGCCGGCCCCTGGCACCAC |
| hsa-miR-7108-3p | ACCCGCCCGTCTCCCCACAG | GTGTGGCCGGCAGGCGGGTGGGCGGGGGCGGCCGGTGGGAACCCCGCCCCGCCCCGCGCCCGCACTCACCCGCCCGTCTCCCCACAG |
| hsa-miR-3916 | AAGAGGAAGAAATGGCTGGTTCTCAG | ATCCCAGAGAAGAAGGAAGAAGAGGAAGAAATGGCTGGTTCTCAGGTGAATGTGTCTGGGTTCAGGGGATGTGTCTCCTCTTTTCTTCTGGGAT |
| hsa-miR-6756-3p | TCCCCTTCCTCCCTGCCCAG | ACCCTAGGGTGGGGCTGGAGGTGGGGCTGAGGCTGAGTCTTCCTCCCCTTCCTCCCTGCCCAG |
| hsa-miR-3170 | CTGGGGTTCTGAGACAGACAGT | CTGGTAACACTGGGGTTCTGAGACAGACAGTGTTAGCTCCAGAAGCATTGCCTGTCTTAGAACCCCTATGTTACCAG |
| hsa-miR-4754 | ATGCGGACCTGGGTTAGCGGAGT | ACGCGCCTGATGCGGACCTGGGTTAGCGGAGTGAGGCCCAGTGGTCACCGCCGCCCTCCGCAGGTCCAGGTTGCCGTGCGCATGTGCCT |
| hsa-miR-5586-3p | CAGAGTGACAAGCTGGTTAAAG | TATCCAGCTTGTTACTATATGCTTTTTAAATGGGGCACAGAGTGACAAGCTGGTTAAAG |
| hsa-miR-490-5p | CCATGGATCTCCAGGTGGGT | TGGAGGCCTTGCTGGTTTGGAAAGTTCATTGTTCGACACCATGGATCTCCAGGTGGGTCAAGTTTAGAGATGCACCAACCTGGAGGACTCCATGCTGTTGAGCTGTTCACAAGCAGCGGACACTTCCA |
| hsa-miR-34c-5p | AGGCAGTGTAGTTAGCTGATTGC | AGTCTAGTTACTAGGCAGTGTAGTTAGCTGATTGCTAATAGTACCAATCACTAACCACACGGCCAGGTAAAAAGATT |
| hsa-miR-522-3p | AAAATGGTTCCCTTTAGAGTGT | TCTCAGGCTGTGTCCCTCTAGAGGGAAGCGCTTTCTGTTGTCTGAAAGAAAAGAAAATGGTTCCCTTTAGAGTGTTACGCTTTGAGA |
| hsa-miR-4306 | TGGAGAGAAAGGCAGTA | AAGCTGCTTAGTGTCCTTAGAGTCTCCAGAGGCATCCCTAACCCAGAATCTTTTGACTGTCCTCTGGAGAGAAAGGCAGTAGGTCTGTACC |
| hsa-miR-3689a-5p | TGTGATATCATGGTTCCTGGGA | GATCCTGTGCTCCCTGGGGGGTCTGATCCTGTGCTTCCTGGGAGGTGTGATATCATGGTTCCTGGGAGGTGTGATCCCGTGCTTCCTGGGAGGTGTGATATTGTGGTTCCTGGGAGGTGTGATCCCGTGCTCCCTGGGAGGTGTGATC |
| hsa-miR-373-3p | GAAGTGCTTCGATTTTGGGGTGT | GGGATACTCAAAATGGGGGCGCTTTCCTTTTTGTCTGTACTGGGAAGTGCTTCGATTTTGGGGTGTCCC |
| hsa-miR-365b-5p | AGGGACTTTCAGGGGCAGCTGT | AGAGTGTTCAAGGACAGCAAGAAAAATGAGGGACTTTCAGGGGCAGCTGTGTTTTCTGACTCAGTCATAATGCCCCTAAAAATCCTTATTGTTCTTGCAGTGTGCATCGGG |
| hsa-miR-4802-3p | TACATGGATGGAAACCTTCAAGC | CTGACTGGCTTGTATGGAGGTTCTAGACCATGTTAGTGTTCAAGTCTACATGGATGGAAACCTTCAAGCAGGCCAAGCAG |
| hsa-miR-6876-3p | AGCTGTCTGTGTTTTCCTTCTCAG | AGTTGCAGGAAGGAGACAGGCAGTTCAGGAGGTGGCACTGCTGTGTGTGAGCTGTCTGTGTTTTCCTTCTCAG |
| hsa-miR-185-5p | TGGAGAGAAAGGCAGTTCCTGA | AGGGGGCGAGGGATTGGAGAGAAAGGCAGTTCCTGATGGTCCCCTCCCCAGGGGCTGGCTTTCCTCTGGTCCTTCCCTCCCA |
| hsa-miR-3127-5p | ATCAGGGCTTGTGGAATGGGAAG | GGCCAGGCCCATCAGGGCTTGTGGAATGGGAAGGAGAAGGGACGCTTCCCCTTCTGCAGGCCTGCTGGGTGTGGCT |
| hsa-miR-6503-3p | GGGACTAGGATGCAGACCTCC | AATGGTCCCCCCAGGGAGGTCTGCATTCAAATCCCCAGAAGCTGAGGATTAGGGGACTAGGATGCAGACCTCCCTGGGGGACCATT |
| hsa-miR-3180-5p | CTTCCAGACGCTCCGCCCCACGTCG | CAGTGCGACGGGCGGAGCTTCCAGACGCTCCGCCCCACGTCGCATGCGCCCCGGGAAAGCGTGGGGCGGAGCTTCCGGAGGCCCCGCCCTGCTG |
| hsa-miR-4477b | ATTAAGGACATTTGTGATTGAT | ACCTCCTCCCGTGAATCACAAATGTCCTTAATAGCAATCCTTAAATGCCATTAAGGACATTTGTGATTGATGGGAGGAGGA |
| hsa-miR-888-5p | TACTCAAAAAGCTGTCAGTCA | GGCAGTGCTCTACTCAAAAAGCTGTCAGTCACTTAGATTACATGTGACTGACACCTCTTTGGGTGAAGGAAGGCTCA |
| hsa-miR-7-2-3p | CAACAAATCCCAGTCTACCTAA | CTGGATACAGAGTGGACCGGCTGGCCCCATCTGGAAGACTAGTGATTTTGTTGTTGTCTTACTGCGCTCAACAACAAATCCCAGTCTACCTAATGGTGCCAGCCATCGCA |
| hsa-miR-4520-2-3p | TTTGGACAGAAAACACGCAGGT | CCTGCGTGTTTTCTGTCCAAATCCTTTTCTGATTTGGACAGAAAACACGCAGGT |
| hsa-miR-6807-3p | CACTGCATTCCTGCTTGGCCCAG | GTGAGCCAGTGGAATGGAGAGGCTGTGGGCAGGGGGAGATGTGAAGGAAAGAACTAGGACCCATTCATCCACTGCATTCCTGCTTGGCCCAG |
| hsa-miR-549a-3p | TGACAACTATGGATGAGCTCT | AGACATGCAACTCAAGAATATATTGAGAGCTCATCCATAGTTGTCACTGTCTCAAATCAGTGACAACTATGGATGAGCTCTTAATATATCCCAGGC |
| hsa-miR-6890-5p | CATGGGGTAGGGCAGAGTAGG | TGGGCCATGGGGTAGGGCAGAGTAGGGCTGGATGGTAGGGCCCACTGCCTATGCCCCACAG |
| hsa-miR-4284 | GGGCTCACATCACCCCAT | GTTCTGTGAGGGGCTCACATCACCCCATCAAAGTGGGGACTCATGGGGAGAGGGGGTAGTTAGGAGCTTTGATAGAGGCGG |
| hsa-miR-4789-3p | CACACATAGCAGGTGTATATA | CATGCTACGTATGTATACACCTGATATGTGTATGTGTAAATACATATCCACACACATAGCAGGTGTATATATAGGTAGCCTG |
| hsa-miR-7702 | CTTAGACTGCCAGACTCCCTGA | CTTAGACTGCCAGACTCCCTGAGGACATGCAGTTTCAGGGAGTCTGGTAGTCTAAGTGT |
| hsa-miR-513a-5p | TTCACAGGGAGGTGTCAT | GGGATGCCACATTCAGCCATTCAGCGTACAGTGCCTTTCACAGGGAGGTGTCATTTATGTGAACTAAAATATAAATTTCACCTTTCTGAGAAGGGTAATGTACAGCATGCACTGCATATGTGGTGTCCC |
| hsa-miR-3667-5p | AAAGACCCATTGAGGAGAAGGT | TGAGGATGAAAGACCCATTGAGGAGAAGGTTCTGCTGGCTGAGAACCTTCCTCTCCATGGGTCTTTCATCCTCA |
| hsa-miR-450a-5p | TTTTGCGATGTGTTCCTAATAT | AAACGATACTAAACTGTTTTTGCGATGTGTTCCTAATATGCACTATAAATATATTGGGAACATTTTGCATGTATAGTTTTGTATCAATATA |
| hsa-miR-1290 | TGGATTTTTGGATCAGGGA | GAGCGTCACGTTGACACTCAAAAAGTTTCAGATTTTGGAACATTTCGGATTTTGGATTTTTGGATCAGGGATGCTCAA |
| hsa-miR-3163 | TATAAAATGAGGGCAGTAAGAC | TTCCTCATCTATAAAATGAGGGCAGTAAGACCTTCCTTCCTTGTCTTACTACCCCCATTTTATAGATGAGGAA |
| hsa-miR-663a | AGGCGGGGCGCCGCGGGACCGC | CCTTCCGGCGTCCCAGGCGGGGCGCCGCGGGACCGCCCTCGTGTCTGTGGCGGTGGGATCCCGCGGCCGTGTTTTCCTGGTGGCCCGGCCATG |
| hsa-miR-9-5p | TCTTTGGTTATCTAGCTGTATGA | CGGGGTTGGTTGTTATCTTTGGTTATCTAGCTGTATGAGTGGTGTGGAGTCTTCATAAAGCTAGATAACCGAAAGTAAAAATAACCCCA |
| hsa-miR-548f-3p | AAAAACTGTAATTACTTTT | ATTAGGTTGGTGCAAAAGTAATCACAGTTTTTGACATTACTTTCAAAGACAAAAACTGTAATTACTTTTGGACCAACCTAATAG |
| hsa-miR-378c | ACTGGACTTGGAGTCAGAAGAGTGG | GGAGGCCATCACTGGACTTGGAGTCAGAAGAGTGGAGTCGGGTCAGACTTCAACTCTGACTTTGAAGGTGGTGAGTGCCTC |
| hsa-miR-216a-3p | TCACAGTGGTCTCTGGGATTAT | GATGGCTGTGAGTTGGCTTAATCTCAGCTGGCAACTGTGAGATGTTCATACAATCCCTCACAGTGGTCTCTGGGATTATGCTAAACAGAGCAATTTCCTAGCCCTCACGA |
| hsa-miR-6831-5p | TAGGTAGAGTGTGAGGAGGAGGTC | GTAGGTAGAGTGTGAGGAGGAGGTCTGAGCCCATGTGTGGACCTAGGTCTGCTGTTAAACTGACTAACTCCCACTCTACAG |
| hsa-miR-892c-3p | CACTGTTTCCTTTCTGAGTGGA | GGCAGTGCCCTATTCAGAAAGGTGCCAGTCACTTAGATTACATGTCACTGTTTCCTTTCTGAGTGGAGTAGGGCTTA |
| hsa-miR-125a-5p | TCCCTGAGACCCTTTAACCTGTGA | TGCCAGTCTCTAGGTCCCTGAGACCCTTTAACCTGTGAGGACATCCAGGGTCACAGGTGAGGTTCTTGGGAGCCTGGCGTCTGGCC |
| hsa-miR-378e | ACTGGACTTGGAGTCAGGA | CTGACTCCAGTGTCCAGGCCAGGGGCAGACAGTGGACAGAGAACAGTGCCCAAGACCACTGGACTTGGAGTCAGGACAT |
| hsa-miR-3617-5p | AAAGACATAGTTGCAAGATGGG | AGGTCATAGAAAGACATAGTTGCAAGATGGGATTAGAAACCATATGTCTCATCAGCACCCTATGTCCTTTCTCTGCCCT |
| hsa-miR-3662 | GAAAATGATGAGTAGTGACTGATG | TGTGTTTTCCTCAACGCTCACAGTTACACTTCTTACTCTCAATCCATTCATATTGAAAATGATGAGTAGTGACTGATGAAGCACAAATCAGCCAA |
| hsa-miR-3140-3p | AGCTTTTGGGAATTCAGGTAGT | CCTCTTGAGGTACCTGAATTACCAAAAGCTTTATGTATTCTGAAGTTATTGAAAATAAGAGCTTTTGGGAATTCAGGTAGTTCAGGAGTG |
| hsa-miR-181b-2-3p | CTCACTGATCAATGAATGCA | CTGATGGCTGCACTCAACATTCATTGCTGTCGGTGGGTTTGAGTCTGAATCAACTCACTGATCAATGAATGCAAACTGCGGACCAAACA |
| hsa-miR-493-3p | TGAAGGTCTACTGTGTGCCAGG | CTGGCCTCCAGGGCTTTGTACATGGTAGGCTTTCATTCATTCGTTTGCACATTCGGTGAAGGTCTACTGTGTGCCAGGCCCTGTGCCAG |
| hsa-miR-99a-5p | AACCCGTAGATCCGATCTTGTG | CCCATTGGCATAAACCCGTAGATCCGATCTTGTGGTGAAGTGGACCGCACAAGCTCGCTTCTATGGGTCTGTGTCAGTGTG |
| hsa-miR-196a-5p | TAGGTAGTTTCATGTTGTTGGG | GTGAATTAGGTAGTTTCATGTTGTTGGGCCTGGGTTTCTGAACACAACAACATTAAACCACCCGATTCAC |
| hsa-miR-4743-5p | TGGCCGGATGGGACAGGAGGCAT | GCTGGCCGGATGGGACAGGAGGCATGAATGAGCCATCTTTCCAATGCCTTTCTGTCTTTTCTGGTCCAG |
| hsa-miR-4723-3p | CCCTCTCTGGCTCCTCCCCAAA | AGTTGGTGGGGGAGCCATGAGATAAGAGCACCTCCTAGAGAATGTTGAACTAAAGGTGCCCTCTCTGGCTCCTCCCCAAAG |
| hsa-miR-6763-5p | CTGGGGAGTGGCTGGGGAG | TTCTCCTGGGGAGTGGCTGGGGAGCAGACAGACCCAACCTCATGCTCCCCGGCCTCTGCCCCCAG |
| hsa-miR-6809-5p | TGGCAAGGAAAGAAGAGGATCA | AATGTTGGCAAGGAAAGAAGAGGATCATGTTTGCCCCCGTGGACAGCTCTCTGGTGTGCTCCTCCTGCCATCCTGCCCACCCCTGCATAATGCTGCTTCTCTTCTCTCCTTCCCAG |
| hsa-miR-890 | TACTTGGAAAGGCATCAGTTG | GGAAGTGCCCTACTTGGAAAGGCATCAGTTGCTTAGATTACATGTAACTATTCCCTTTCTGAGTAGAGTAAGTCTTA |
| hsa-miR-4667-3p | TCCCTCCTTCTGTCCCCACAG | TGACTGGGGAGCAGAAGGAGAACCCAAGAAAAGCTGACTTGGAGGTCCCTCCTTCTGTCCCCACAG |
| hsa-miR-1247-5p | ACCCGTCCCGTTCGTCCCCGGA | CCGCTTGCCTCGCCCAGCGCAGCCCCGGCCGCTGGGCGCACCCGTCCCGTTCGTCCCCGGACGTTGCTCTCTACCCCGGGAACGTCGAGACTGGAGCGCCCGAACTGAGCCACCTTCGCGGACCCCGAGAGCGGCG |
| hsa-miR-6512-5p | TACCATTAGAAGAGCTGGAAGA | TATGCTCTTACCATTAGAAGAGCTGGAAGAAGGCTGAGGGAGATGCCTTCTTCCAGCCCTTCTAATGGTAGGAGCAT |
| hsa-miR-3613-3p | ACAAAAAAAAAAGCCCAACCCTTC | TGGTTGGGTTTGGATTGTTGTACTTTTTTTTTTGTTCGTTGCATTTTTAGGAACAAAAAAAAAAGCCCAACCCTTCACACCACTTCA |
| hsa-miR-548a-5p | AAAAGTAATTGCGAGTTTTACC | CCTAGAATGTTATTAGGTCGGTGCAAAAGTAATTGCGAGTTTTACCATTACTTTCAATGGCAAAACTGGCAATTACTTTTGCACCAACGTAATACTT |
| hsa-miR-1180-5p | GGACCCACCCGGCCGGGAATA | GCTGCTGGACCCACCCGGCCGGGAATAGTGCTCCTGGTTGTTTCCGGCTCGCGTGGGTGTGTCGGCGGC |
| hsa-miR-526b-3p | GAAAGTGCTTCCTTTTAGAGGC | TCAGGCTGTGACCCTCTTGAGGGAAGCACTTTCTGTTGTCTGAAAGAAGAGAAAGTGCTTCCTTTTAGAGGCTTACTGTCTGA |
| hsa-miR-4669 | TGTGTCCGGGAAGTGGAGGAGG | GCCTCCCTTCACTTCCTGGCCATCCAGGCATCTGTGTCTGTGTCCGGGAAGTGGAGGAGGGC |
| hsa-miR-497-5p | CAGCAGCACACTGTGGTTTGT | CCACCCCGGTCCTGCTCCCGCCCCAGCAGCACACTGTGGTTTGTACGGCACTGTGGCCACGTCCAAACCACACTGTGGTGTTAGAGCGAGGGTGGGGGAGGCACCGCCGAGG |
| hsa-miR-4426 | GAAGATGGACGTACTTT | AGTTGGAAGATGGACGTACTTTGTCTGACTACAATATTCAAAAGGAGTCTACTCTTCATCTTG |
| hsa-miR-6889-5p | TCGGGGAGTCTGGGGTCCGGAAT | CTGTGTCGGGGAGTCTGGGGTCCGGAATTCTCCAGAGCCTCTGTGCCCCTACTTCCCAG |
| hsa-miR-642b-3p | AGACACATTTGGAGAGGGACCC | GAGTTGGGAGGTTCCCTCTCCAAATGTGTCTTGATCCCCCACCCCAAGACACATTTGGAGAGGGACCCTCCCAACTC |
| hsa-miR-363-3p | AATTGCACGGTATCCATCTGTA | TGTTGTCGGGTGGATCACGATGCAATTTTGATGAGTATCATAGGAGAAAAATTGCACGGTATCCATCTGTAAACC |
| hsa-miR-3620-3p | TCACCCTGCATCCCGCACCCAG | GTGAGGTGGGGGCCAGCAGGGAGTGGGCTGGGCTGGGCTGGGCCAAGGTACAAGGCCTCACCCTGCATCCCGCACCCAG |
| hsa-miR-6869-5p | GTGAGTAGTGGCGCGCGGCGGC | GTGAGTAGTGGCGCGCGGCGGCTCGGAGTACCTCTGCCGCCGCGCGCATCGGCTCAGCATGC |
| hsa-miR-320a-5p | GCCTTCTCTTCCCGGTTCTTCC | CTCCCCTCCGCCTTCTCTTCCCGGTTCTTCCCGGAGTCGGGAAAAGCTGGGTTGAGAGGGCGAAAAAGGATG |
| hsa-miR-3154 | CAGAAGGGGAGTTGGGAGCAGA | GGCCCCTCCTTCTCAGCCCCAGCTCCCGCTCACCCCTGCCACGTCAAAGGAGGCAGAAGGGGAGTTGGGAGCAGAGAGGGGACC |
| hsa-miR-6853-3p | TGTTCATTGGAACCCTGCGCAG | GGGAAAGCGTGGGATGTCCATGAAGTCAGGTGATGGTGATAAGGTCAAGGCCTGTTCATTGGAACCCTGCGCAG |
| hsa-miR-4520-3p | TTGGACAGAAAACACGCAGGAA | GTGTGCCACCTGCGTGTTTTCTGTCCAAATCAGAAAAGGATTTGGACAGAAAACACGCAGGAAGAAGGAA |
| hsa-miR-493-5p | TTGTACATGGTAGGCTTTCATT | CTGGCCTCCAGGGCTTTGTACATGGTAGGCTTTCATTCATTCGTTTGCACATTCGGTGAAGGTCTACTGTGTGCCAGGCCCTGTGCCAG |
| hsa-miR-629-5p | TGGGTTTACGTTGGGAGAACT | TCCCTTTCCCAGGGGAGGGGCTGGGTTTACGTTGGGAGAACTTTTACGGTGAACCAGGAGGTTCTCCCAACGTAAGCCCAGCCCCTCCCCTCTGCCT |
| hsa-miR-6776-3p | CAACCACCACTGTCTCTCCCCAG | CGGGCTCTGGGTGCAGTGGGGGTTCCCACGCCGCGGCAACCACCACTGTCTCTCCCCAG |
| hsa-miR-4423-5p | AGTTGCCTTTTTGTTCCCATGC | ATCATGTACTGCAGTTGCCTTTTTGTTCCCATGCTGTTTAAGCCTAGCATAGGCACCAAAAAGCAACAACAGTATGTGAA |
| hsa-miR-5009-5p | TTGGACTTTTTCAGATTTGGGGAT | GACCAGAAGTGTTTTGGATTTTGGACTTTTTCAGATTTGGGGATATTTGCATTATACTTATCCTAAATCTGAAAGTCCAAAACCTGAAATGACCAATAAG |
| hsa-miR-6780b-3p | TCCCTTGTCTCCTTTCCCTAG | CAGCCTGGGGAAGGCTTGGCAGGGAAGACACATGAGCAGTGCCTCCACTTCACGCCTCTCCCTTGTCTCCTTTCCCTAG |
| hsa-miR-655-3p | ATAATACATGGTTAACCTCTTT | AACTATGCAAGGATATTTGAGGAGAGGTTATCCGTGTTATGTTCGCTTCATTCATCATGAATAATACATGGTTAACCTCTTTTTGAATATCAGACTC |
| hsa-miR-181d-5p | AACATTCATTGTTGTCGGTGGGT | GTCCCCTCCCCTAGGCCACAGCCGAGGTCACAATCAACATTCATTGTTGTCGGTGGGTTGTGAGGACTGAGGCCAGACCCACCGGGGGATGAATGTCACTGTGGCTGGGCCAGACACGGCTTAAGGGGAATGGGGAC |
| hsa-miR-4443 | TTGGAGGCGTGGGTTTT | GGTGGGGGTTGGAGGCGTGGGTTTTAGAACCTATCCCTTTCTAGCCCTGAGCA |
| hsa-miR-4528 | TCATTATATGTATGATCTGGAC | TATTCTACTGAGAGTACAGATCTTTATATATATGATCATTATATGTATGATGAGATCATTATATGTATGATCTGGACACCCAGTAGAATC |
| hsa-miR-2116-3p | CCTCCCATGCCAAGAACTCCC | GACCTAGGCTAGGGGTTCTTAGCATAGGAGGTCTTCCCATGCTAAGAAGTCCTCCCATGCCAAGAACTCCCAGACTAGGA |
| hsa-miR-6895-5p | CAGGGCCAGGCACAGAGTAAG | CAGCTCAGGGCCAGGCACAGAGTAAGCATCAATAGCATTGGCAAGTTGAACTGAGCTGTCTCTCGCCCTTGGCCTTAG |
| hsa-miR-22-5p | AGTTCTTCAGTGGCAAGCTTTA | GGCTGAGCCGCAGTAGTTCTTCAGTGGCAAGCTTTATGTCCTGACCCAGCTAAAGCTGCCAGTTGAAGAACTGTTGCCCTCTGCC |
| hsa-miR-3190-3p | TGTGGAAGGTAGACGGCCAGAGA | CTGGGGTCACCTGTCTGGCCAGCTACGTCCCCACGGCCCTTGTCAGTGTGGAAGGTAGACGGCCAGAGAGGTGACCCCGG |
| hsa-miR-18b-3p | TGCCCTAAATGCCCCTTCTGGC | TGTGTTAAGGTGCATCTAGTGCAGTTAGTGAAGCAGCTTAGAATCTACTGCCCTAAATGCCCCTTCTGGCA |
| hsa-miR-28-5p | AAGGAGCTCACAGTCTATTGAG | GGTCCTTGCCCTCAAGGAGCTCACAGTCTATTGAGTTACCTTTCTGACTTTCCCACTAGATTGTGAGCTCCTGGAGGGCAGGCACT |
| hsa-miR-323a-3p | CACATTACACGGTCGACCTCT | TTGGTACTTGGAGAGAGGTGGTCCGTGGCGCGTTCGCTTTATTTATGGCGCACATTACACGGTCGACCTCTTTGCAGTATCTAATC |
| hsa-miR-3173-3p | AAAGGAGGAAATAGGCAGGCCA | TCCCTGCCCTGCCTGTTTTCTCCTTTGTGATTTTATGAGAACAAAGGAGGAAATAGGCAGGCCAGGGA |
| hsa-miR-6861-5p | ACTGGGTAGGTGGGGCTCCAGG | GAGGCACTGGGTAGGTGGGGCTCCAGGGCTCCTGACACCTGGACCTCTCCTCCCCAGGCCCACA |
| hsa-miR-6767-5p | TCGCAGACAGGGACACATGGAGA | TGAAATCGCAGACAGGGACACATGGAGAACGCCCCCACCAGTTCCCACGTGCTTCTCTTTCCGCAG |
| hsa-miR-519c-3p | AAAGTGCATCTTTTTAGAGGAT | TCTCAGCCTGTGACCCTCTAGAGGGAAGCGCTTTCTGTTGTCTGAAAGAAAAGAAAGTGCATCTTTTTAGAGGATTACAGTTTGAGA |
| hsa-miR-6785-3p | ACATCGCCCCACCTTCCCCAG | CTCCCTGGGAGGGCGTGGATGATGGTGGGAGAGGAGCCCCACTGTGGAAGTCTGACCCCCACATCGCCCCACCTTCCCCAG |
| hsa-miR-129-5p | CTTTTTGCGGTCTGGGCTTGC | GGATCTTTTTGCGGTCTGGGCTTGCTGTTCCTCTCAACAGTAGTCAGGAAGCCCTTACCCCAAAAAGTATCT |
| hsa-miR-1295b-3p | AATAGGCCACGGATCTGGGCAA | CACCCAGATCTGCGGCCTAATCACAGGCCACATTTCTGAATAGGCCACGGATCTGGGCAA |
| hsa-miR-9903 | TTATCCTCCAGTAGACTAGGGA | CCAGCTCTGGTTCCCCAGCCTACTGGAGGATAAGAGGATATAAAGGTCTCTTATCCTCCAGTAGACTAGGGAGCCAGAGCTGGTAATAAAAGTC |
| hsa-miR-4733-5p | AATCCCAATGCTAGACCCGGTG | GGTCGCTTAAATCCCAATGCTAGACCCGGTGGCAATCAAGGTCTAGCCACCAGGTCTAGCATTGGGATTTAAGCCC |
| hsa-miR-3136-3p | TGGCCCAACCTATTCAGTTAGT | AATATGAAACTGACTGAATAGGTAGGGTCATTTTTCTGTGACTGCACATGGCCCAACCTATTCAGTTAGTTCCATATT |
| hsa-miR-182-5p | TTTGGCAATGGTAGAACTCACACT | GAGCTGCTTGCCTCCCCCCGTTTTTGGCAATGGTAGAACTCACACTGGTGAGGTAACAGGATCCGGTGGTTCTAGACTTGCCAACTATGGGGCGAGGACTCAGCCGGCAC |
| hsa-miR-548ak | AAAAGTAACTGCGGTTTTTGA | GTGCAAAAGTAACTGCGGTTTTTGAGAAGTAATTGAAAACCGCAATTACTTTTGCAG |
| hsa-miR-5588-5p | ACTGGCATTAGTGGGACTTTT | ACTGGCATTAGTGGGACTTTTTTTTTTTTTTTTTTTAATGTTAAAAGTCCCACTAATGCCAGC |
| hsa-miR-504-5p | AGACCCTGGTCTGCACTCTATC | GCTGCTGTTGGGAGACCCTGGTCTGCACTCTATCTGTATTCTTACTGAAGGGAGTGCAGGGCAGGGTTTCCCATACAGAGGGC |
| hsa-miR-411-3p | TATGTAACACGGTCCACTAACC | TGGTACTTGGAGAGATAGTAGACCGTATAGCGTACGCTTTATCTGTGACGTATGTAACACGGTCCACTAACCCTCAGTATCAAATCCATCCCCGAG |
| hsa-miR-7111-3p | ATCCTCTCTTCCCTCCTCCCAG | CTGGGGGAGGAAGGACAGGCCATCTGCTATTCGTCCACCAACCTGACTTGATCCTCTCTTCCCTCCTCCCAG |
| hsa-miR-6727-5p | CTCGGGGCAGGCGGCTGGGAGCG | GGGTGCTCGGGGCAGGCGGCTGGGAGCGGCCCTCACATTGATGGCTCCTGCCACCTCCTCCGCAG |
| hsa-miR-514a-3p | ATTGACACTTCTGTGAGTAGA | AACATGTTGTCTGTGGTACCCTACTCTGGAGAGTGACAATCATGTATAATTAAATTTGATTGACACTTCTGTGAGTAGAGTAACGCATGACACGTACG |
| hsa-miR-4672 | TTACACAGCTGGACAGAGGCA | GGCTGCTTCTCGCCTCTGTCCAGCTGTGTGGCCTTGGACAAGCCTCTTGGTTACACAGCTGGACAGAGGCACGAAACAGCC |
| hsa-miR-4714-3p | CCAACCTAGGTGGTCAGAGTTG | ATTTTGGCCAACTCTGACCCCTTAGGTTGATGTCAGAATGAGGTGTACCAACCTAGGTGGTCAGAGTTGGCCAAAAT |
| hsa-miR-4753-3p | TTCTCTTTCTTTAGCCTTGTGT | ATATCTACACAAGGCCAAAGGAAGAGAACAGATATATCCACAGTACACTTGGCTGTTCTCTTTCTTTAGCCTTGTGTAGATAT |
| hsa-miR-3925-5p | AAGAGAACTGAAAGTGGAGCCT | GTGGGAATAGCAAGAGAACTGAAAGTGGAGCCTGTCACATCTCCAGACTCCAGTTTTAGTTCTCTTGCTATTTCCAC |
| hsa-miR-132-5p | ACCGTGGCTTTCGATTGTTACT | CCGCCCCCGCGTCTCCAGGGCAACCGTGGCTTTCGATTGTTACTGTGGGAACTGGAGGTAACAGTCTACAGCCATGGTCGCCCCGCAGCACGCCCACGCGC |
| hsa-miR-6509-5p | ATTAGGTAGTGGCAGTGGAAC | TTTTTGTGTGTGAAATTAGGTAGTGGCAGTGGAACACTATATTAATCAGGTTTCCACTGCCACTACCTAATTTCTCAGATGGAAA |
| hsa-miR-1909-3p | CGCAGGGGCCGGGTGCTCACCG | CATCCAGGACAATGGTGAGTGCCGGTGCCTGCCCTGGGGCCGTCCCTGCGCAGGGGCCGGGTGCTCACCGCATCTGCCCC |
| hsa-miR-147b-3p | GTGTGCGGAAATGCTTCTGCT | TATAAATCTAGTGGAAACATTTCTGCACAAACTAGATTCTGGACACCAGTGTGCGGAAATGCTTCTGCTACATTTTTAGG |
| hsa-miR-30c-5p | TGTAAACATCCTACACTCTCAGC | AGATACTGTAAACATCCTACACTCTCAGCTGTGGAAAGTAAGAAAGCTGGGAGAAGGCTGTTTACTCTTTCT |
| hsa-miR-455-5p | TATGTGCCTTTGGACTACATCG | TCCCTGGCGTGAGGGTATGTGCCTTTGGACTACATCGTGGAAGCCAGCACCATGCAGTCCATGGGCATATACACTTGCCTCAAGGCCTATGTCATC |
| hsa-miR-4761-3p | GAGGGCATGCGCACTTTGTCC | GGACAAGGTGTGCATGCCTGACCCGTTGTCAGACCTGGAAAAAGGGCCGGCTGTGGGCAGGGAGGGCATGCGCACTTTGTCC |
| hsa-miR-5585-3p | CTGAATAGCTGGGACTACAGGT | TGAAGTACCAGCTACTCGAGAGGTCAGAGGATTGCTCCTGAATAGCTGGGACTACAGGT |
| hsa-miR-6893-3p | CCCTGCTGCCTTCACCTGCCAG | CCGGGCAGGCAGGTGTAGGGTGGAGCCCACTGTGGCTCCTGACTCAGCCCTGCTGCCTTCACCTGCCAG |
| hsa-miR-6889-3p | TCTGTGCCCCTACTTCCCAG | CTGTGTCGGGGAGTCTGGGGTCCGGAATTCTCCAGAGCCTCTGTGCCCCTACTTCCCAG |
| hsa-miR-195-3p | CCAATATTGGCTGTGCTGCTCC | AGCTTCCCTGGCTCTAGCAGCACAGAAATATTGGCACAGGGAAGCGAGTCTGCCAATATTGGCTGTGCTGCTCCAGGCAGGGTGGTG |
| hsa-miR-1303 | TTTAGAGACGGGGTCTTGCTCT | GGCTGGGCAACATAGCGAGACCTCAACTCTACAATTTTTTTTTTTTTAAATTTTAGAGACGGGGTCTTGCTCTGTTGCCAGGCTTT |
| hsa-miR-10392-5p | GCGCTTCGACGGGCTGGGCTGTG | GCGCTTCGACGGGCTGGGCTGTGCGCCTGCGCAGTGTGGGTCGCTCCCGATTCCCTGCCCCGGCCGGCCCCGCCTCGGCTCCGCACC |
| hsa-miR-4649-3p | TCTGAGGCCTGCCTCTCCCCA | TCTGGGCGAGGGGTGGGCTCTCAGAGGGGCTGGCAGTACTGCTCTGAGGCCTGCCTCTCCCCAG |
| hsa-miR-548g-5p | TGCAAAAGTAATTGCAGTTTTTG | AAGGTATTAGGTTGGTGCAAAAGTAATTGCAGTTTTTGCTATTACTTTTAATGGTAAAAACTGCAATTACTTTTACACCAACCTAATATTTA |
| hsa-miR-4747-3p | AAGGCCCGGGCTTTCCTCCCAG | AGGGAAGGAGGCTTGGTCTTAGCACGGGGTCTAAGGCCCGGGCTTTCCTCCCAG |
| hsa-miR-7161-3p | TAGATCTTTGACTCTGGCAGTCTCCAGG | TAAAGACTGTAGAGGCAACTGGTGTTCTCACGCAAAGTGGCCAGGGTGTGGGAGACTAGATCTTTGACTCTGGCAGTCTCCAGG |
| hsa-miR-1295a | TTAGGCCGCAGATCTGGGTGA | AGGACATTTTGCCCAGATCCGTGGCCTATTCAGAAATGTGGCCTGTGATTAGGCCGCAGATCTGGGTGAAATGTCCTCC |
| hsa-miR-376b-5p | CGTGGATATTCCTTCTATGTTT | CAGTCCTTCTTTGGTATTTAAAACGTGGATATTCCTTCTATGTTTACGTGATTCCTGGTTAATCATAGAGGAAAATCCATGTTTTCAGTATCAAATGCTG |
| hsa-miR-6800-5p | GTAGGTGACAGTCAGGGGCGG | ACCTGTAGGTGACAGTCAGGGGCGGGGTGTGGTGGGGCTGGGGCTGGCCCCCTCCTCACACCTCTCCTGGCATCGCCCCCAG |
| hsa-miR-506-5p | TATTCAGGAAGGTGTTACTTAA | GCCACCACCATCAGCCATACTATGTGTAGTGCCTTATTCAGGAAGGTGTTACTTAATAGATTAATATTTGTAAGGCACCCTTCTGAGTAGAGTAATGTGCAACATGGACAACATTTGTGGTGGC |
| hsa-miR-653-3p | TTCACTGGAGTTTGTTTCAATA | TTCATTCCTTCAGTGTTGAAACAATCTCTACTGAACCAGCTTCAAACAAGTTCACTGGAGTTTGTTTCAATATTGCAAGAATGATAAGATGGAAGC |
| hsa-miR-6871-3p | CAGCACCCTGTGGCTCCCACAG | CTCCTCATGGGAGTTCGGGGTGGTTGCTGGAGGTCAGCACCCTGTGGCTCCCACAG |
| hsa-miR-299-5p | TGGTTTACCGTCCCACATACAT | AAGAAATGGTTTACCGTCCCACATACATTTTGAATATGTATGTGGGATGGTAAACCGCTTCTT |
| hsa-miR-548p | TAGCAAAAACTGCAGTTACTTT | ATTAGGTTGGTATAAAATTAATTGCAGTTTTTGTCATTACTTTCAATAGCAAAAACTGCAGTTACTTTTGCACCAATGTAATAC |
| hsa-miR-4470 | TGGCAAACGTGGAAGCCGAGA | CGAGCCTCTTTCGGCTTTCCAGTTTGTCTCGGTCCTTTGGAACGTGGCAAACGTGGAAGCCGAGAGGGCTCT |
| hsa-miR-6788-5p | CTGGGAGAAGAGTGGTGAAGA | GACGGCTGGGAGAAGAGTGGTGAAGAAGAGTATTGATTGTGCTGTTCGCCACTTCCCTCCCTGCAG |
| hsa-miR-3137 | TCTGTAGCCTGGGAGCAATGGGGT | TACAGGTCTGTAGCCTGGGAGCAATGGGGTGTATGGTATAGGGGTAGCCTCGTGCTCCTGGGCTACAAACCTGTA |
| hsa-miR-615-3p | TCCGAGCCTGGGTCTCCCTCTT | CTCGGGAGGGGCGGGAGGGGGGTCCCCGGTGCTCGGATCTCGAGGGTGCTTATTGTTCGGTCCGAGCCTGGGTCTCCCTCTTCCCCCCAACCCCCC |
| hsa-miR-1185-2-3p | ATATACAGGGGGAGACTCTCAT | TTTGGTACTTAAAGAGAGGATACCCTTTGTATGTTCACTTGATTAATGGCGAATATACAGGGGGAGACTCTCATTTGCGTATCAAA |
| hsa-miR-5691 | TTGCTCTGAGCTCCGAGAAAGC | GGACAAGCTTGCTCTGAGCTCCGAGAAAGCTGACAGACAGCTGCTTGGTGTTCAGAGCTTGTCTGTCC |
| hsa-miR-524-5p | CTACAAAGGGAAGCACTTTCTC | TCTCATGCTGTGACCCTACAAAGGGAAGCACTTTCTCTTGTCCAAAGGAAAAGAAGGCGCTTCCCTTTGGAGTGTTACGGTTTGAGA |
| hsa-miR-3685 | TTTCCTACCCTACCTGAAGACT | GTACATTTCCTACCCTACCTGAAGACTTGAGATTATAGTCTTTGGGGGGATGGGCAAAGTAC |
| hsa-miR-7977 | TTCCCAGCCAACGCACCA | TTCCCAGCCAACGCACCAAAAATGATATGGGTCTGTTGTCTGGAGAAAC |
| hsa-miR-519d-3p | CAAAGTGCCTCCCTTTAGAGTG | TCCCATGCTGTGACCCTCCAAAGGGAAGCGCTTTCTGTTTGTTTTCTCTTAAACAAAGTGCCTCCCTTTAGAGTGTTACCGTTTGGGA |
| hsa-miR-5581-3p | TTCCATGCCTCCTAGAAGTTCC | AGCCTTCCAGGAGAAATGGAGACCCTATACATACCTGTTTCCATGCCTCCTAGAAGTTCC |
| hsa-miR-10b-3p | ACAGATTCGATTCTAGGGGAAT | CCAGAGGTTGTAACGTTGTCTATATATACCCTGTAGAACCGAATTTGTGTGGTATCCGTATAGTCACAGATTCGATTCTAGGGGAATATATGGTCGATGCAAAAACTTCA |
| hsa-miR-454-5p | ACCCTATCAATATTGTCTCTGC | TCTGTTTATCACCAGATCCTAGAACCCTATCAATATTGTCTCTGCTGTGTAAATAGTTCTGAGTAGTGCAATATTGCTTATAGGGTTTTGGTGTTTGGAAAGAACAATGGGCAGG |
| hsa-miR-10400-5p | CGGCGGCGGCGGCTCTGGGCG | CGGCGGCGGCGGCTCTGGGCGAGGCGGCGGGGCCTGGGCTCCCGGACGAGGCGGG |
| hsa-miR-548ay-3p | CAAAACCGCGATTACTCTTGCA | AGAAGATGCTTACTACTAGGTTGGTGCAAAAGTAATTGTGGTTTTTGCATTTAAAGTAATGGCCAAAACCGCGATTACTCTTGCACGAACCTAACGGTAACACTTCT |
| hsa-miR-5579-3p | TTAGCTTAAGGAGTACCAGATC | TATGGTACTCCTTAAGCTAACAGGCCCCTGTCACCATTAGCTTAAGGAGTACCAGATC |
| hsa-miR-337-3p | CTCCTATATGATGCCTTTCTTC | GTAGTCAGTAGTTGGGGGGTGGGAACGGCTTCATACAGGAGTTGATGCACAGTTATCCAGCTCCTATATGATGCCTTTCTTCATCCCCTTCAA |
| hsa-miR-92a-3p | TATTGCACTTGTCCCGGCCTGT | CTTTCTACACAGGTTGGGATCGGTTGCAATGCTGTGTTTCTGTATGGTATTGCACTTGTCCCGGCCTGTTGAGTTTGG |
| hsa-miR-6852-5p | CCCTGGGGTTCTGAGGACATG | TGCTGCCCTGGGGTTCTGAGGACATGCTCTGACTCCCCTGATGTCCTCTGTTCCTCAGGTGCTGGG |
| hsa-miR-4763-3p | AGGCAGGGGCTGGTGCTGGGCGGG | CCTGTCCCTCCTGCCCTGCGCCTGCCCAGCCCTCCTGCTCTGGTGACTGAGGACCGCCAGGCAGGGGCTGGTGCTGGGCGGGGGGCGGCGGG |
| hsa-miR-548i | AAAAGTAATTGCGGATTTTGCC | CAGATGGCTCTGAAGTTTGCACCCTATTAGGTTGGTGCAAAAGTAATTGCGGATTTTGCCATTAAAAGTAATGGCAAAAATAGCAATTATTTTTGTACCAGCCTAGTATCTTTTCTCCTTCTACCAAACTTTGTCCCTGAGCCATCTCA |
| hsa-miR-660-5p | TACCCATTGCATATCGGAGTTG | CTGCTCCTTCTCCCATACCCATTGCATATCGGAGTTGTGAATTCTCAAAACACCTCCTGTGTGCATGGATTACAGGAGGGTGAGCCTTGTCATCGTG |
| hsa-miR-520a-5p | CTCCAGAGGGAAGTACTTTCT | CTCAGGCTGTGACCCTCCAGAGGGAAGTACTTTCTGTTGTCTGAGAGAAAAGAAAGTGCTTCCCTTTGGACTGTTTCGGTTTGAG |
| hsa-miR-12136 | GAAAAAGTCATGGAGGCC | GAAAAAGTCATGGAGGCCATGGGGTTGGCTTGAAACCAGCTTTGGGGGGTTCGATTCCTTCCTTTTTTGTC |
| hsa-miR-151a-3p | CTAGACTGAAGCTCCTTGAGG | TTTCCTGCCCTCGAGGAGCTCACAGTCTAGTATGTCTCATCCCCTACTAGACTGAAGCTCCTTGAGGACAGGGATGGTCATACTCACCTC |
| hsa-miR-3941 | TTACACACAACTGAGGATCATA | GAGTCAGAATTCTCATCAGGCTGTGATGCTCAGTTGTGTGTAGATTGAAAGCCCTAATTTTACACACAACTGAGGATCATAGCCTGATGGTTCCTTTTTGTTT |
| hsa-miR-3155b | CCAGGCTCTGCAGTGGGA | CCACTGCAGAGCCTGGGAAGGGAGCTGTCCGGCTCCCCAGGCTCTGCAGTGGGAGG |
| hsa-miR-135a-2-3p | ATGTAGGGATGGAAGCCATGAA | AGATAAATTCACTCTAGTGCTTTATGGCTTTTTATTCCTATGTGATAGTAATAAAGTCTCATGTAGGGATGGAAGCCATGAAATACATTGTGAAAAATCA |
| hsa-miR-200c-5p | CGTCTTACCCAGCAGTGTTTGG | CCCTCGTCTTACCCAGCAGTGTTTGGGTGCGGTTGGGAGTCTCTAATACTGCCGGGTAATGATGGAGG |
| hsa-miR-26b-3p | CCTGTTCTCCATTACTTGGCT | CCGGGACCCAGTTCAAGTAATTCAGGATAGGTTGTGTGCTGTCCAGCCTGTTCTCCATTACTTGGCTCGGGGACCGG |
| hsa-miR-1468-5p | CTCCGTTTGCCTGTTTCGCTG | GGTGGGTGGTTTCTCCGTTTGCCTGTTTCGCTGATGTGCATTCAACTCATTCTCAGCAAAATAAGCAAATGGAAAATTCGTCCATC |
| hsa-miR-6715b-5p | ACAGGCACGACTGGTTTGGCA | GGTTGTGCCCACAGGCACGACTGGTTTGGCAATTCAGTAAAAATGCTCAAACCGGCTGTGCCTGTGGACGCAGCCCA |
| hsa-miR-519c-5p | CTCTAGAGGGAAGCGCTTTCTG | TCTCATGCTGTGACCCTCTAGAGGGAAGCGCTTTCTGTTGTCTGAAAGAAAAGAACGCGCTTCCCTATAGAGGGTTACCCTTTGAGA |
| hsa-miR-4796-3p | TAAAGTGGCAGAGTATAGACAC | TAAATTTGTGTCTATACTCTGTCACTTTACTTTTGGCCTCAAGTCATTGCAGTAAAGTGGCAGAGTATAGACACAAATTTA |
| hsa-miR-582-3p | TAACTGGTTGAACAACTGAACC | ATCTGTGCTCTTTGATTACAGTTGTTCAACCAGTTACTAATCTAACTAATTGTAACTGGTTGAACAACTGAACCCAAAGGGTGCAAAGTAGAAACATT |
| hsa-miR-4433a-3p | ACAGGAGTGGGGGTGGGACAT | CATCCTCCTTACGTCCCACCCCCCACTCCTGTTTCTGGTGAAATATTCAAACAGGAGTGGGGGTGGGACATAAGGAGGATA |
| hsa-miR-106b-3p | CCGCACTGTGGGTACTTGCTGC | CCTGCCGGGGCTAAAGTGCTGACAGTGCAGATAGTGGTCCTCTCCGTGCTACCGCACTGTGGGTACTTGCTGCTCCAGCAGG |
| hsa-miR-6854-3p | TGCGTTTCTCCTCTTGAGCAG | AAAGCAAGCTCAGGTTTGAGAACTGCTGATGTCATCAGTCATAACTTCTGCGTTTCTCCTCTTGAGCAG |
| hsa-miR-5571-5p | CAATTCTCAAAGGAGCCTCCC | ATCTGACACAAAATGTGAACCAAGCAATTCTCAAAGGAGCCTCCCAGGAAATTCACTTTAGGAAGTCCTAGGAGGCTCCTCTGAGAGTTGCTAAAACAAAACATTGAGAGTCC |
| hsa-miR-3148 | TGGAAAAAACTGGTGTGTGCTT | GAGTTAAGATGGAAAAAACTGGTGTGTGCTTATTGATGTAGCCAACAAGCATACATCAGTTTTTTCCAACTTAACTC |
| hsa-miR-433-5p | TACGGTGAGCCTGTCATTATTC | CCGGGGAGAAGTACGGTGAGCCTGTCATTATTCAGAGAGGCTAGATCCTCTGTGTTGAGAAGGATCATGATGGGCTCCTCGGTGTTCTCCAGG |
| hsa-miR-1269b | CTGGACTGAGCCATGCTACTGG | TGAGGTTTCTGGACTGAGCCATGCTACTGGCTTCTCTGGTTCTCCAGCTTACAGATGGCTTATCATGGGACCTCT |
| hsa-miR-4762-3p | CTTCTGATCAAGATTTGTGGTG | CTGATACCCCAAATCTTGATCAGAAGCCTTGATCAGAAGCTAGGAAGGCTTCTGATCAAGATTTGTGGTGTCAAG |
| hsa-miR-942-5p | TCTTCTCTGTTTTGGCCATGTG | ATTAGGAGAGTATCTTCTCTGTTTTGGCCATGTGTGTACTCACAGCCCCTCACACATGGCCGAAACAGAGAAGTTACTTTCCTAAT |
| hsa-miR-129-2-3p | AAGCCCTTACCCCAAAAAGCAT | TGCCCTTCGCGAATCTTTTTGCGGTCTGGGCTTGCTGTACATAACTCAATAGCCGGAAGCCCTTACCCCAAAAAGCATTTGCGGAGGGCG |
| hsa-miR-6739-3p | ATTGTTCTGTCTTTCTCCCAG | GAATGTGGGAAAGAGAAAGAACAAGTAAAAGGAATTTTCATTTTCCAGCCCCTAATTGTTCTGTCTTTCTCCCAG |
| hsa-miR-3177-5p | TGTGTACACACGTGCCAGGCGCT | CCACGTGCCATGTGTACACACGTGCCAGGCGCTGTCTTGAGACATTCGCGCAGTGCACGGCACTGGGGACACGTGGCACTGG |
| hsa-miR-4492 | GGGGCTGGGCGCGCGCC | CTGCAGCGTGCTTCTCCAGGCCCCGCGCGCGGACAGACACACGGACAAGTCCCGCCAGGGGCTGGGCGCGCGCCAGCCGG |
| hsa-miR-4779 | TAGGAGGGAATAGTAAAAGCAG | TAAATGTCTTACTGCTTTTACTGTTCCCTCCTAGAGTCCATTCTTTACTCTAGGAGGGAATAGTAAAAGCAGTAAGACATTTA |
| hsa-miR-3126-3p | CATCTGGCATCCGTCACACAGA | ATGATTATATGAGGGACAGATGCCAGAAGCACTGGTTATGATTTGCATCTGGCATCCGTCACACAGATAATTAT |
| hsa-miR-5001-3p | TTCTGCCTCTGTCCAGGTCCTT | AGCTCAGGGCGGCTGCGCAGAGGGCTGGACTCAGCGGCGGAGCTGGCTGCTGGCCTCAGTTCTGCCTCTGTCCAGGTCCTTGTGACCCGCCCGCTCTCCT |
| hsa-miR-3145-3p | AGATATTTTGAGTGTTTGGAATTG | TATATGAGTTCAACTCCAAACACTCAAAACTCATTGTTGAATGGAATGAGATATTTTGAGTGTTTGGAATTGAACTCGTATA |
| hsa-miR-7109-3p | CAAGCCTCTCCTGCCCTTCCAG | GTCTCCTGGGGGGAGGAGACCCTGCTCTCCCTGGCAGCAAGCCTCTCCTGCCCTTCCAGATTAGC |
| hsa-miR-4670-5p | AAGCGACCATGATGTAACTTCA | CTCTAGGAAGCGACCATGATGTAACTTCACAGACTCTCCAAAAGTCTGAAGTTACATCATGGTCGCTTCCTAGAG |
| hsa-miR-98-5p | TGAGGTAGTAAGTTGTATTGTT | AGGATTCTGCTCATGCCAGGGTGAGGTAGTAAGTTGTATTGTTGTGGGGTAGGGATATTAGGCCCCAATTAGAAGATAACTATACAACTTACTACTTTCCCTGGTGTGTGGCATATTCA |
| hsa-miR-6827-5p | TGGGAGCCATGAGGGTCTGTGC | TCTGGTGGGAGCCATGAGGGTCTGTGCTGTCTCTGAGCACCGTCTCTTCTGTTCCCCAG |
| hsa-miR-29a-3p | TAGCACCATCTGAAATCGGTTA | ATGACTGATTTCTTTTGGTGTTCAGAGTCAATATAATTTTCTAGCACCATCTGAAATCGGTTAT |
| hsa-miR-7113-3p | CCTCCCTGCCCGCCTCTCTGCAG | CTCCAGGGAGACAGTGTGTGAGGCCTCTTGCCATGGCCTCCCTGCCCGCCTCTCTGCAG |
| hsa-miR-362-5p | AATCCTTGGAACCTAGGTGTGAGT | CTTGAATCCTTGGAACCTAGGTGTGAGTGCTATTTCAGTGCAACACACCTATTCAAGGATTCAAA |
| hsa-miR-1286 | TGCAGGACCAAGATGAGCCCT | TGTCCTCTGGGGACTCAGCTTGCTCTGGCTGCTGGATTGAATTAGCTGCAGGACCAAGATGAGCCCTTGGTGGAGACA |
| hsa-miR-549a-5p | AGCTCATCCATAGTTGTCACTG | AGACATGCAACTCAAGAATATATTGAGAGCTCATCCATAGTTGTCACTGTCTCAAATCAGTGACAACTATGGATGAGCTCTTAATATATCCCAGGC |
| hsa-miR-505-3p | CGTCAACACTTGCTGGTTTCCT | GATGCACCCAGTGGGGGAGCCAGGAAGTATTGATGTTTCTGCCAGTTTAGCGTCAACACTTGCTGGTTTCCTCTCTGGAGCATC |
| hsa-miR-3675-5p | TATGGGGCTTCTGTAGAGATTTC | GGATGATAAGTTATGGGGCTTCTGTAGAGATTTCTATGAGAACATCTCTAAGGAACTCCCCCAAACTGAATTC |
| hsa-miR-7855-5p | TTGGTGAGGACCCCAAGCTCGG | GCTTGGTGAGGACCCCAAGCTCGGCACTATTGTGACTGTGCTTTTGGCTTCCTTTCCAGGC |
| hsa-miR-15a-5p | TAGCAGCACATAATGGTTTGTG | CCTTGGAGTAAAGTAGCAGCACATAATGGTTTGTGGATTTTGAAAAGGTGCAGGCCATATTGTGCTGCCTCAAAAATACAAGG |
| hsa-miR-548as-5p | AAAAGTAATTGCGGGTTTTGCC | AAAAGTAATTGCGGGTTTTGCCGTTGCTTTTAATGGTAAAACCCACAATTATGTTTGT |
| hsa-miR-642b-5p | GGTTCCCTCTCCAAATGTGTCT | GAGTTGGGAGGTTCCCTCTCCAAATGTGTCTTGATCCCCCACCCCAAGACACATTTGGAGAGGGACCCTCCCAACTC |
| hsa-miR-550a-3-5p | AGTGCCTGAGGGAGTAAGAG | GATGCTTTGCTGGCTGGTGCAGTGCCTGAGGGAGTAAGAGTCCTGTTGTTGTAAGATAGTGTCTTACTCCCTCAGGCACATCTCCAACAAGTCTC |
| hsa-miR-3622b-5p | AGGCATGGGAGGTCAGGTGA | AGTGATATAATAGAGGGTGCACAGGCATGGGAGGTCAGGTGAGCTCAGCTCCCTGCCTCACCTGAGCTCCCGTGCCTGTGCACCCTCTATTGGCT |
| hsa-miR-6777-5p | ACGGGGAGTCAGGCAGTGGTGGA | TCAAGACGGGGAGTCAGGCAGTGGTGGAGATGGAGAGCCCTGAGCCTCCACTCTCCTGGCCCCCAG |
| hsa-miR-147a | GTGTGTGGAAATGCTTCTGC | AATCTAAAGACAACATTTCTGCACACACACCAGACTATGGAAGCCAGTGTGTGGAAATGCTTCTGCTAGATT |
| hsa-miR-10525-3p | TGACTATGATGTGCACCTGAT | AAGGTGTATGATGGGACTTGGGAGAAAGTACTCCCGGGTGACTATGATGTGCACCTGAT |
| hsa-miR-432-5p | TCTTGGAGTAGGTCATTGGGTGG | TGACTCCTCCAGGTCTTGGAGTAGGTCATTGGGTGGATCCTCTATTTCCTTACGTGGGCCACTGGATGGCTCCTCCATGTCTTGGAGTAGATCA |
| hsa-miR-204-3p | GCTGGGAAGGCAAAGGGACGT | GGCTACAGTCTTTCTTCATGTGACTCGTGGACTTCCCTTTGTCATCCTATGCCTGAGAATATATGAAGGAGGCTGGGAAGGCAAAGGGACGTTCAATTGTCATCACTGGC |
| hsa-miR-3663-3p | TGAGCACCACACAGGCCGGGCGC | CCCGGGACCTTGGTCCAGGCGCTGGTCTGCGTGGTGCTCGGGTGGATAAGTCTGATCTGAGCACCACACAGGCCGGGCGCCGGGACCAAGGGGGCTC |
| hsa-miR-1913 | TCTGCCCCCTCCGCTGCTGCCA | ACCTCTACCTCCCGGCAGAGGAGGCTGCAGAGGCTGGCTTTCCAAAACTCTGCCCCCTCCGCTGCTGCCAAGTGGCTGGT |
| hsa-miR-4529-5p | AGGCCATCAGCAGTCCAATGAA | ATGACAGGCCATCAGCAGTCCAATGAAGACATGAAGACCCAATGTCTTCATTGGACTGCTGATGGCCCGTCACTGGGA |
| hsa-miR-6886-5p | CCCGCAGGTGAGATGAGGGCT | CTTGGCCCGCAGGTGAGATGAGGGCTCCTGGCGCTGATGCCCTTCTCTCCTCCTGCCTCAG |
| hsa-miR-3184-5p | TGAGGGGCCTCAGACCGAGCTTTT | AAGCAAGACTGAGGGGCCTCAGACCGAGCTTTTGGAAAATAGAAAAGTCTCGCTCTCTGCCCCTCAGCCTAACTT |
| hsa-miR-4633-5p | ATATGCCTGGCTAGCTCCTC | TGGCAAGTCTCCGCATATGCCTGGCTAGCTCCTCCACAAATGCGTGTGGAGGAGCTAGCCAGGCATATGCAGAGCGTCA |
| hsa-miR-345-3p | GCCCTGAACGAGGGGTCTGGAG | ACCCAAACCCTAGGTCTGCTGACTCCTAGTCCAGGGCTCGTGATGGCTGGTGGGCCCTGAACGAGGGGTCTGGAGGCCTGGGTTTGAATATCGACAGC |
| hsa-miR-3176 | ACTGGCCTGGGACTACCGG | TGGCCTCTCCAGTCTGCAGCTCCCGGCAGCCTCGGGCCACACTCCCGGGATCCCCAGGGACTGGCCTGGGACTACCGGGGGTGGCGGCCG |
| hsa-miR-6824-3p | TCTCTGGTCTTGCCACCCCAG | GAGGTGTAGGGGAGGTTGGGCCAGGGATGCCTTCACTGTGTCTCTCTGGTCTTGCCACCCCAG |
| hsa-miR-9986 | TGTGAGGTTGTCATGCCTGC | AAGAAGTATTTTGCAAAAAGTGTGAGGTTGTCATGCCTGCTATTACTACAGCTATGCCAGCAGGCATGACAACCTCATACTTTTTGCAGAATAGTTTTTTATATTGTATT |
| hsa-miR-502-5p | ATCCTTGCTATCTGGGTGCTA | TGCTCCCCCTCTCTAATCCTTGCTATCTGGGTGCTAGTGCTGGCTCAATGCAATGCACCTGGGCAAGGATTCAGAGAGGGGGAGCT |
| hsa-miR-1180-3p | TTTCCGGCTCGCGTGGGTGTGT | GCTGCTGGACCCACCCGGCCGGGAATAGTGCTCCTGGTTGTTTCCGGCTCGCGTGGGTGTGTCGGCGGC |
| hsa-miR-5187-3p | ACTGAATCCTCTTTTCCTCAG | GACTAAGGGTGGGATGAGGGATTGAAGTGGAGCAGGAATGCGCTTTTCTCCACTGAATCCTCTTTTCCTCAGGTGG |
| hsa-miR-3944-5p | TGTGCAGCAGGCCAACCGAGA | TCCACCCAGCAGGCGCAGGTCCTGTGCAGCAGGCCAACCGAGAAGCGCCTGCGTCTCCCATTTTCGGGCTGGCCTGCTGCTCCGGACCTGTGCCTGATCTTAATGCTG |
| hsa-miR-664a-3p | TATTCATTTATCCCCAGCCTACA | GAACATTGAAACTGGCTAGGGAAAATGATTGGATAGAAACTATTATTCTATTCATTTATCCCCAGCCTACAAAATGAAAAAA |
| hsa-miR-548at-5p | AAAAGTTATTGCGGTTTTGGCT | AAAAGTTATTGCGGTTTTGGCTGCCAAAAGAAATGGCCAAAACCGCAGTAACTTTTGT |
| hsa-miR-143-5p | GGTGCAGTGCTGCATCTCTGGT | GCGCAGCGCCCTGTCTCCCAGCCTGAGGTGCAGTGCTGCATCTCTGGTCAGTTGGGAGTCTGAGATGAAGCACTGTAGCTCAGGAAGAGAGAAGTTGTTCTGCAGC |
| hsa-miR-383-5p | AGATCAGAAGGTGATTGTGGCT | CTCCTCAGATCAGAAGGTGATTGTGGCTTTGGGTGGATATTAATCAGCCACAGCACTGCCTGGTCAGAAAGAG |
| hsa-miR-503-5p | TAGCAGCGGGAACAGTTCTGCAG | TGCCCTAGCAGCGGGAACAGTTCTGCAGTGAGCGATCGGTGCTCTGGGGTATTGTTTCCGCTGCCAGGGTA |
| hsa-miR-6875-5p | TGAGGGACCCAGGACAGGAGA | GAGTCTGAGGGACCCAGGACAGGAGAAGGCCTATGGTGATTTGCATTCTTCCTGCCCTGGCTCCATCCTCAG |
| hsa-miR-1234-3p | TCGGCCTGACCACCCACCCCAC | GTGAGTGTGGGGTGGCTGGGGCGGGGGGGGCCCGGGGACGGCTTGGGCCTGCCTAGTCGGCCTGACCACCCACCCCACAG |
| hsa-miR-6779-5p | CTGGGAGGGGCTGGGTTTGGC | GAGCTCTGGGAGGGGCTGGGTTTGGCAGGACAGTTTCCAAGCCCTGTCTCCTCCCATCTTCCAG |
| hsa-miR-1233-3p | TGAGCCCTGTCCTCCCGCAG | GTGAGTGGGAGGCCAGGGCACGGCAGGGGGAGCTGCAGGGCTATGGGAGGGGCCCCAGCGTCTGAGCCCTGTCCTCCCGCAG |
| hsa-miR-6769b-5p | TGGTGGGTGGGGAGGAGAAGTGC | CTTCCTGGTGGGTGGGGAGGAGAAGTGCCGTCCTCATGAGCCCCTCTCTGTCCCACCCATAG |
| hsa-miR-133a-5p | AGCTGGTAAAATGGAACCAAAT | ACAATGCTTTGCTAGAGCTGGTAAAATGGAACCAAATCGCCTCTTCAATGGATTTGGTCCCCTTCAACCAGCTGTAGCTATGCATTGA |
| hsa-miR-6501-3p | CCAGAGCAGCCTGCGGTAACAGT | GGAGTTGCCAGGGCTGCCTTTGGTGACAGCAGCAGTAGAGTTGCCAGAGCAGCCTGCGGTAACAGTA |
| hsa-miR-1260b | ATCCCACCACTGCCACCAT | TCTCCGTTTATCCCACCACTGCCACCATTATTGCTACTGTTCAGCAGGTGCTGCTGGTGGTGATGGTGATAGTCTGGTGGGGGCGGTGG |
| hsa-miR-4536-3p | TCGTGCATATATCTACCACAT | ATGTGGTAGATATATGCACGATATATATACTGCCCTGCTTTTATACATACATACATACATACCTATATCGTGCATATATCTACCACAT |
| hsa-miR-185-3p | AGGGGCTGGCTTTCCTCTGGTC | AGGGGGCGAGGGATTGGAGAGAAAGGCAGTTCCTGATGGTCCCCTCCCCAGGGGCTGGCTTTCCTCTGGTCCTTCCCTCCCA |
| hsa-miR-4529-3p | ATTGGACTGCTGATGGCCCGT | ATGACAGGCCATCAGCAGTCCAATGAAGACATGAAGACCCAATGTCTTCATTGGACTGCTGATGGCCCGTCACTGGGA |
| hsa-miR-1243 | AACTGGATCAATTATAGGAGTG | CTAAAACTGGATCAATTATAGGAGTGAAATAAAGGTCCATCTCCTGCCTATTTATTACTTTGCTTTGGTAATAAATCTATTTTTAAAAGAACC |
| hsa-miR-6750-3p | GAACTCACCCTCTGCTCCCAG | GCTGTCAGGGAACAGCTGGGTGAGCTGCTGCCCCAGAGGCCCAGCAGGTGTCCAGAACTCACCCTCTGCTCCCAG |
| hsa-miR-6778-3p | TGCCTCCCTGACATTCCACAG | GTTCAAGTGGGAGGACAGGAGGCAGGTGTGGTTGGAGGAAGCAGCCTGAACCTGCCTCCCTGACATTCCACAG |
| hsa-miR-449b-5p | AGGCAGTGTATTGTTAGCTGGC | TGACCTGAATCAGGTAGGCAGTGTATTGTTAGCTGGCTGCTTGGGTCAAGTCAGCAGCCACAACTACCCTGCCACTTGCTTCTGGATAAATTCTTCT |
| hsa-miR-4636 | AACTCGTGTTCAAAGCCTTTAG | TAGATTCAGAACTCGTGTTCAAAGCCTTTAGCCCAGCAATGGGAGAGTGCTAAAGGCTTCAAGCACGAGTTCTGAATCTA |
| hsa-miR-1262 | ATGGGTGAATTTGTAGAAGGAT | ATCTACAATGGTGATGGGTGAATTTGTAGAAGGATGAAAGTCAAAGAATCCTTCTGGGAACTAATTTTTGGCCTTCAACAAGAATTGTGATAT |
| hsa-miR-4795-3p | ATATTATTAGCCACTTCTGGAT | TGATATGGAAGAAATCCAGAAGTGGCTAATAATATTGACACTATAACAATAATGTCAATATTATTAGCCACTTCTGGATTTATGAATCA |
| hsa-miR-374b-5p | ATATAATACAACCTGCTAAGTG | ACTCGGATGGATATAATACAACCTGCTAAGTGTCCTAGCACTTAGCAGGTTGTATTATCATTGTCCGTGTCT |
| hsa-miR-29c-5p | TGACCGATTTCTCCTGGTGTTC | ATCTCTTACACAGGCTGACCGATTTCTCCTGGTGTTCAGAGTCTGTTTTTGTCTAGCACCATTTGAAATCGGTTATGATGTAGGGGGA |
| hsa-miR-30c-1-3p | CTGGGAGAGGGTTGTTTACTCC | ACCATGCTGTAGTGTGTGTAAACATCCTACACTCTCAGCTGTGAGCTCAAGGTGGCTGGGAGAGGGTTGTTTACTCCTTCTGCCATGGA |
| hsa-miR-616-3p | AGTCATTGGAGGGTTTGAGCAG | TTAGGTAATTCCTCCACTCAAAACCCTTCAGTGACTTCCATGACATGAAATAGGAAGTCATTGGAGGGTTTGAGCAGAGGAATGACCTGTTTTAAAA |
| hsa-miR-548w | AAAAGTAACTGCGGTTTTTGCCT | GGTTGGTGCAAAAGTAACTGCGGTTTTTGCCTTTCAACATAATGGCAAAACCCACAATTACTTTTGCACCAATC |
| hsa-miR-561-5p | ATCAAGGATCTTAAACTTTGCC | CTTCATCCACCAGTCCTCCAGGAACATCAAGGATCTTAAACTTTGCCAGAGCTACAAAGGCAAAGTTTAAGATCCTTGAAGTTCCTGGGGGAACCAT |
| hsa-miR-4445-5p | AGATTGTTTCTTTTGCCGTGCA | TTCCTGCAGATTGTTTCTTTTGCCGTGCAAGTTTAAGTTTTTGCACGGCAAAAGAAACAATCCAGAGGGT |
| hsa-miR-4786-3p | TGAAGCCAGCTCTGGTCTGGGC | GGGCATGGCCTGAGACCAGGACTGGATGCACCACTCTCCCTGTGATGAGGTGAAGCCAGCTCTGGTCTGGGCCATTTCAC |
| hsa-miR-23b-5p | TGGGTTCCTGGCATGCTGATTT | CTCAGGTGCTCTGGCTGCTTGGGTTCCTGGCATGCTGATTTGTGACTTAAGATTAAAATCACATTGCCAGGGATTACCACGCAACCACGACCTTGGC |
| hsa-miR-125b-5p | TCCCTGAGACCCTAACTTGTGA | TGCGCTCCTCTCAGTCCCTGAGACCCTAACTTGTGATGTTTACCGTTTAAATCCACGGGTTAGGCTCTTGGGAGCTGCGAGTCGTGCT |
| hsa-miR-6804-5p | TGAGGGTGTCAGCAGGTGACG | GGATGTGAGGGTGTCAGCAGGTGACGGTGGGGGCCACGCTGACAGCCGCACCTGCCTCTCACCCACAG |
| hsa-miR-3651 | CATAGCCCGGTCGCTGGTACATGA | GATTCGATGGGCCATAGCAATCCTGTGATTTATGCATGGAGGCTGCTTCTCCTCAGCAGCTGCCATAGCCCGGTCGCTGGTACATGATTC |
| hsa-miR-545-5p | TCAGTAAATGTTTATTAGATGA | CCCAGCCTGGCACATTAGTAGGCCTCAGTAAATGTTTATTAGATGAATAAATGAATGACTCATCAGCAAACATTTATTGTGTGCCTGCTAAAGTGAGCTCCACAGG |
| hsa-miR-371b-5p | ACTCAAAAGATGGCGGCACTTT | GGTAACACTCAAAAGATGGCGGCACTTTCACCAGAGAGCAGAAAGTGCCCCCACAGTTTGAGTGCC |
| hsa-miR-4653-5p | TCTCTGAGCAAGGCTTAACACC | TTGTCCAATTCTCTGAGCAAGGCTTAACACCAAAGGGTTAAGGGTTTGCTCTGGAGTTAAGGGTTGCTTGGAGAATTGGAGAA |
| hsa-miR-302c-3p | TAAGTGCTTCCATGTTTCAGTGG | CCTTTGCTTTAACATGGGGGTACCTGCTGTGTGAAACAAAAGTAAGTGCTTCCATGTTTCAGTGGAGG |
| hsa-miR-516b-5p | ATCTGGAGGTAAGAAGCACTTT | TCTCATGATGTGACCATCTGGAGGTAAGAAGCACTTTGTGTTTTGTGAAAGAAAGTGCTTCCTTTCAGAGGGTTACTCTTTGAGA |
| hsa-miR-671-3p | TCCGGTTCTCAGGGCTCCACC | GCAGGTGAACTGGCAGGCCAGGAAGAGGAGGAAGCCCTGGAGGGGCTGGAGGTGATGGATGTTTTCCTCCGGTTCTCAGGGCTCCACCTCTTTCGGGCCGTAGAGCCAGGGCTGGTGC |
| hsa-miR-1261 | ATGGATAAGGCTTTGGCTT | TGCTATGGATAAGGCTTTGGCTTATGGGGATATTGTGGTTGATCTGTTCTATCCAGATGACTGAAACTTTCTCCATAGCAGC |
| hsa-miR-545-3p | TCAGCAAACATTTATTGTGTGC | CCCAGCCTGGCACATTAGTAGGCCTCAGTAAATGTTTATTAGATGAATAAATGAATGACTCATCAGCAAACATTTATTGTGTGCCTGCTAAAGTGAGCTCCACAGG |
| hsa-miR-184 | TGGACGGAGAACTGATAAGGGT | CCAGTCACGTCCCCTTATCACTTTTCCAGCCCAGCTTTGTGACTGTAAGTGTTGGACGGAGAACTGATAAGGGTAGGTGATTGA |
| hsa-miR-7151-3p | CTACAGGCTGGAATGGGCTCA | GATCCATCTCTGCCTGTATTGGCTTGGATTCTGCAAAGCCTACAGGCTGGAATGGGCTCA |
| hsa-miR-93-3p | ACTGCTGAGCTAGCACTTCCCG | CTGGGGGCTCCAAAGTGCTGTTCGTGCAGGTAGTGTGATTACCCAACCTACTGCTGAGCTAGCACTTCCCGAGCCCCCGG |
| hsa-miR-135b-5p | TATGGCTTTTCATTCCTATGTGA | CACTCTGCTGTGGCCTATGGCTTTTCATTCCTATGTGATTGCTGTCCCAAACTCATGTAGGGCTAAAAGCCATGGGCTACAGTGAGGGGCGAGCTCC |
| hsa-miR-195-5p | TAGCAGCACAGAAATATTGGC | AGCTTCCCTGGCTCTAGCAGCACAGAAATATTGGCACAGGGAAGCGAGTCTGCCAATATTGGCTGTGCTGCTCCAGGCAGGGTGGTG |
| hsa-miR-651-3p | AAAGGAAAGTGTATCCTAAAAG | AATCTATCACTGCTTTTTAGGATAAGCTTGACTTTTGTTCAAATAAAAATGCAAAAGGAAAGTGTATCCTAAAAGGCAATGACAGTTTAATGTGTTT |
| hsa-miR-125a-3p | ACAGGTGAGGTTCTTGGGAGCC | TGCCAGTCTCTAGGTCCCTGAGACCCTTTAACCTGTGAGGACATCCAGGGTCACAGGTGAGGTTCTTGGGAGCCTGGCGTCTGGCC |
| hsa-let-7a-2-3p | CTGTACAGCCTCCTAGCTTTCC | AGGTTGAGGTAGTAGGTTGTATAGTTTAGAATTACATCAAGGGAGATAACTGTACAGCCTCCTAGCTTTCCT |
| hsa-miR-6819-3p | AAGCCTCTGTCCCCACCCCAG | GAGGGTTGGGGTGGAGGGCCAAGGAGCTGGGTGGGGTGCCAAGCCTCTGTCCCCACCCCAG |
| hsa-miR-224-3p | AAAATGGTGCCCTAGTGACTACA | GGGCTTTCAAGTCACTAGTGGTTCCGTTTAGTAGATGATTGTGCATTGTTTCAAAATGGTGCCCTAGTGACTACAAAGCCC |
| hsa-miR-520d-3p | AAAGTGCTTCTCTTTGGTGGGT | TCTCAAGCTGTGAGTCTACAAAGGGAAGCCCTTTCTGTTGTCTAAAAGAAAAGAAAGTGCTTCTCTTTGGTGGGTTACGGTTTGAGA |
| hsa-miR-450b-5p | TTTTGCAATATGTTCCTGAATA | GCAGAATTATTTTTGCAATATGTTCCTGAATATGTAATATAAGTGTATTGGGATCATTTTGCATCCATAGTTTTGTAT |
| hsa-miR-3182 | GCTTCTGTAGTGTAGTC | GCTGCTTCTGTAGTGTAGTCCGTGCATCCGCCCTTCGATGCTTGGGTTGGATCATAGAGCAGT |
| hsa-miR-4704-5p | GACACTAGGCATGTGAGTGATT | CTTATCCTAGACACTAGGCATGTGAGTGATTGTCTTCCTCACTCAATCAGTCACATATCTAGTGTCTAGAATGAG |
| hsa-miR-6894-5p | AGGAGGATGGAGAGCTGGGCCAGA | CAAGAAGGAGGATGGAGAGCTGGGCCAGACATGCTCTTGCCTGCCCTCTTCCTCCAG |
| hsa-miR-4683 | TGGAGATCCAGTGCTCGCCCGAT | GACACGCAAGACGAGGCGGGCCTGGAGGTGCACCAGTTCTGGCCGCTGGTGGAGATCCAGTGCTCGCCCGATCTCAAGTTC |
| hsa-miR-1228-5p | GTGGGCGGGGGCAGGTGTGTG | GTGGGCGGGGGCAGGTGTGTGGTGGGTGGTGGCCTGCGGTGAGCAGGGCCCTCACACCTGCCTCGCCCCCCAG |
| hsa-miR-7114-3p | TGACCCACCCCTCTCCACCAG | TCCGCTCTGTGGAGTGGGGTGCCTGTCCCCTGCCACTGGGTGACCCACCCCTCTCCACCAG |
| hsa-miR-299-3p | TATGTGGGATGGTAAACCGCTT | AAGAAATGGTTTACCGTCCCACATACATTTTGAATATGTATGTGGGATGGTAAACCGCTTCTT |
| hsa-miR-636 | TGTGCTTGCTCGTCCCGCCCGCA | TGGCGGCCTGGGCGGGAGCGCGCGGGCGGGGCCGGCCCCGCTGCCTGGAATTAACCCCGCTGTGCTTGCTCGTCCCGCCCGCAGCCCTAGGCGGCGTCG |
| hsa-miR-3150b-5p | CAACCTCGAGGATCTCCCCAGC | GAGGGAAAGCAGGCCAACCTCGAGGATCTCCCCAGCCTTGGCGTTCAGGTGCTGAGGAGATCGTCGAGGTTGGCCTGCTTCCCCTC |
| hsa-miR-6834-5p | GTGAGGGACTGGGATTTGTGG | GTGAGGGACTGGGATTTGTGGGGCGAGGAGGGACCTGTACTAGCCATGGTTCTGATCACATATGTCCCATCCCTCCATCAG |
| hsa-miR-3174 | TAGTGAGTTAGAGATGCAGAGCC | GTTACCTGGTAGTGAGTTAGAGATGCAGAGCCCTGGGCTCCTCAGCAAACCTACTGGATCTGCATTTTAATTCACATGCATGGTAAT |
| hsa-miR-934 | TGTCTACTACTGGAGACACTGG | AGAAATAAGGCTTCTGTCTACTACTGGAGACACTGGTAGTATAAAACCCAGAGTCTCCAGTAATGGACGGGAGCCTTATTTCT |
| hsa-miR-3939 | TACGCGCAGACCACAGGATGTC | CTGGCTTCCAAAGGCCTCTGTGTGTTCCTGTATGTGGGCGTGCACGTACCTGTCACATGTGTACGCGCAGACCACAGGATGTCCACACTGGCTTCCAAACACATCT |
| hsa-miR-548am-3p | CAAAAACTGCAGTTACTTTTGT | AGTTGGTGCAAAAGTAATTGCGGTTTTTGCCGTCGAAAATAATGGCAAAAACTGCAGTTACTTTTGTACCAATG |
| hsa-miR-424-5p | CAGCAGCAATTCATGTTTTGAA | CGAGGGGATACAGCAGCAATTCATGTTTTGAAGTGTTCTAAATGGTTCAAAACGTGAGGCGCTGCTATACCCCCTCGTGGGGAAGGTAGAAGGTGGGG |
| hsa-miR-194-5p | TGTAACAGCAACTCCATGTGGA | ATGGTGTTATCAAGTGTAACAGCAACTCCATGTGGACTGTGTACCAATTTCCAGTGGAGATGCTGTTACTTTTGATGGTTACCAA |
| hsa-miR-3667-3p | ACCTTCCTCTCCATGGGTCTTT | TGAGGATGAAAGACCCATTGAGGAGAAGGTTCTGCTGGCTGAGAACCTTCCTCTCCATGGGTCTTTCATCCTCA |
| hsa-miR-1322 | GATGATGCTGCTGATGCTG | AGTATCATGAATTAGAAACCTACTTATTACATAGTTTACATAAGAAGCGTGATGATGCTGCTGATGCTGTA |
| hsa-miR-6842-3p | TTGGCTGGTCTCTGCTCCGCAG | AGCCCTGGGGGTGGTCTCTAGCCAAGGCTCTGGGGTCTCACCCTTGGCTGGTCTCTGCTCCGCAG |
| hsa-miR-5189-3p | TGCCAACCGTCAGAGCCCAGA | GGCCCGCCTTTTAGGGGCCTCGCTGTCTGGGCACAGGCGGATGGACAGGCTGGCCTCTGGATGACCTGCCAACCGTCAGAGCCCAGACCCACGTGGCCTCAGTTGGGGACCAGG |
| hsa-miR-4742-3p | TCTGTATTCTCCTTTGCCTGCAG | TCAGGCAAAGGGATATTTACAGATACTTTTTAAAATTTGTTTGAGTTGAGGCAGATTAAATATCTGTATTCTCCTTTGCCTGCAG |
| hsa-miR-190b-5p | TGATATGTTTGATATTGGGTTG | TGCTTCTGTGTGATATGTTTGATATTGGGTTGTTTAATTAGGAACCAACTAAATGTCAAACATATTCTTACAGCAGCAG |
| hsa-miR-548bb-5p | AAAAGTAACTATGGTTTTTGCC | TTAGATTGGTGCAAAAGTAACTATGGTTTTTGCCCAAAAACCATAGTTACTTTTGCACCAAGCTAA |
| hsa-miR-4518 | GCTCAGGGATGATAACTGTGCTGAGA | TGGGGGAAAAGTGCTGGGATTGATTAGTGATGTCTGCTGGGGAACCGGGGCTCAGGGATGATAACTGTGCTGAGAAGCCCCCT |
| hsa-miR-609 | AGGGTGTTTCTCTCATCTCT | TGCTCGGCTGTTCCTAGGGTGTTTCTCTCATCTCTGGTCTATAATGGGTTAAATAGTAGAGATGAGGGCAACACCCTAGGAACAGCAGAGGAACC |
| hsa-miR-3609 | CAAAGTGATGAGTAATACTGGCTG | GTAACAGTAACTTTTATTCTCATTTTCCTTTTCTCTACCTTGTAGAGAAGCAAAGTGATGAGTAATACTGGCTGGAGCCC |
| hsa-miR-3944-3p | TTCGGGCTGGCCTGCTGCTCCGG | TCCACCCAGCAGGCGCAGGTCCTGTGCAGCAGGCCAACCGAGAAGCGCCTGCGTCTCCCATTTTCGGGCTGGCCTGCTGCTCCGGACCTGTGCCTGATCTTAATGCTG |
| hsa-miR-548aw | GTGCAAAAGTCATCACGGTT | TAGGTCGGTGCAAAAGTCATCACGGTTTTTACCATTAAAACCGCGATGACTTTTGCATCAACCTA |
| hsa-miR-7975 | ATCCTAGTCACGGCACCA | GTGCAAAGAGCAGGAGGACAGGGGATTTATCTCCCAAGGGAGGTCCCCTGATCCTAGTCACGGCACCA |
| hsa-miR-548a-3p | CAAAACTGGCAATTACTTTTGC | TGCAGGGAGGTATTAAGTTGGTGCAAAAGTAATTGTGATTTTTGCCATTAAAAGTAACGACAAAACTGGCAATTACTTTTGCACCAAACCTGGTATT |
| hsa-miR-4440 | TGTCGTGGGGCTTGCTGGCTTG | CTCTCACCAAGCAAGTGCAGTGGGGCTTGCTGGCTTGCACCGTGACTCCCTCTCACCAAGCAAGTGTCGTGGGGCTTGCTGGCTTGCACTGTGAAGAT |
| hsa-miR-1255a | AGGATGAGCAAAGAAAGTAGATT | ATTGGAAATCCTTTGAGTTGCTTCTCAAGGATGAGCAAAGAAAGTAGATTTTTTAGATTCTAAAGAAACTATCTTCTTTGCTCATCCTTGAGAAGCAACTCCTTATCCATTAA |
| hsa-miR-378h | ACTGGACTTGGTGTCAGATGG | ACAGGAACACTGGACTTGGTGTCAGATGGGATGAGCCCTGGCTCTGTTTCCTAGCAGCAATCTGATCTTGAGCTAGTCACTGG |
| hsa-miR-7976 | TGCCCTGAGACTTTTGCTC | TGCCCTGAGACTTTTGCTCTAATAATTTATTCTAATAATAATTTAGATCAAAAGCCTCAGGGCAGA |
| hsa-miR-4727-5p | ATCTGCCAGCTTCCACAGTGG | AATCTGCCAGCTTCCACAGTGGCAGATTTTCCCATAGTGGGAAGCTGGCAGATTC |
| hsa-miR-451b | TAGCAAGAGAACCATTACCATT | TGGGTATAGCAAGAGAACCATTACCATTACTAAACTCAGTAATGGTAACGGTTTCCTTGCCATTCCCA |
| hsa-miR-6832-3p | ACCCTTTTTCTCTTTCCCAG | GGTGGAGTAGAGAGGAAAAGTTAGGGTCAGTGGCAGAGCCAGGCAGATGCTGACCCTTTTTCTCTTTCCCAG |
| hsa-miR-4660 | TGCAGCTCTGGTGGAAAATGGAG | ACTCCTTCTGCAGCTCTGGTGGAAAATGGAGAAGACTTTTCCTTTCCTCCATCTCCCCCAGGGCCTGGTGGAGT |
| hsa-miR-619-5p | GCTGGGATTACAGGCATGAGCC | CGCCCACCTCAGCCTCCCAAAATGCTGGGATTACAGGCATGAGCCACTGCGGTCGACCATGACCTGGACATGTTTGTGCCCAGTACTGTCAGTTTGCAG |
| hsa-miR-6838-3p | AAGTCCTGCTTCTGTTGCAG | CAGGGAAGCAGCAGTGGCAAGACTCCTAGGTCACGGAAGTCCTGCTTCTGTTGCAG |
| hsa-miR-597-5p | TGTGTCACTCGATGACCACTGT | TACTTACTCTACGTGTGTGTCACTCGATGACCACTGTGAAGACAGTAAAATGTACAGTGGTTCTCTTGTGGCTCAAGCGTAATGTAGAGTACTGGTC |
| hsa-miR-550a-3p | TGTCTTACTCCCTCAGGCACAT | TGATGCTTTGCTGGCTGGTGCAGTGCCTGAGGGAGTAAGAGCCCTGTTGTTGTAAGATAGTGTCTTACTCCCTCAGGCACATCTCCAACAAGTCTCT |
| hsa-miR-3144-3p | ATATACCTGTTCGGTCTCTTTA | AACTACACTTTAAGGGGACCAAAGAGATATATAGATATCAGCTACCTATATACCTGTTCGGTCTCTTTAAAGTGTAGTT |
| hsa-let-7e-5p | TGAGGTAGGAGGTTGTATAGTT | CCCGGGCTGAGGTAGGAGGTTGTATAGTTGAGGAGGACACCCAAGGAGATCACTATACGGCCTCCTAGCTTTCCCCAGG |
| hsa-miR-3127-3p | TCCCCTTCTGCAGGCCTGCTGG | GGCCAGGCCCATCAGGGCTTGTGGAATGGGAAGGAGAAGGGACGCTTCCCCTTCTGCAGGCCTGCTGGGTGTGGCT |
| hsa-miR-4510 | TGAGGGAGTAGGATGTATGGTT | GTGTATGTGAGGGAGTAGGATGTATGGTTGTTAGATAGACAACTACAATCTTTTCTCACAACAGACAG |
| hsa-miR-4708-5p | AGAGATGCCGCCTTGCTCCTT | TTTAGGAGAGAGATGCCGCCTTGCTCCTTGAACAGGAGGAGCAAGGCGGCATCTCTCTGATACTAAA |
| hsa-miR-5189-5p | TCTGGGCACAGGCGGATGGACAGG | GGCCCGCCTTTTAGGGGCCTCGCTGTCTGGGCACAGGCGGATGGACAGGCTGGCCTCTGGATGACCTGCCAACCGTCAGAGCCCAGACCCACGTGGCCTCAGTTGGGGACCAGG |
| hsa-let-7f-1-3p | CTATACAATCTATTGCCTTCCC | TCAGAGTGAGGTAGTAGATTGTATAGTTGTGGGGTAGTGATTTTACCCTGTTCAGGAGATAACTATACAATCTATTGCCTTCCCTGA |
| hsa-miR-760 | CGGCTCTGGGTCTGTGGGGA | GGCGCGTCGCCCCCCTCAGTCCACCAGAGCCCGGATACCTCAGAAATTCGGCTCTGGGTCTGTGGGGAGCGAAATGCAAC |
| hsa-miR-583 | CAAAGAGGAAGGTCCCATTAC | AACTCACACATTAACCAAAGAGGAAGGTCCCATTACTGCAGGGATCTTAGCAGTACTGGGACCTACCTCTTTGGT |
| hsa-let-7c-3p | CTGTACAACCTTCTAGCTTTCC | GCATCCGGGTTGAGGTAGTAGGTTGTATGGTTTAGAGTTACACCCTGGGAGTTAACTGTACAACCTTCTAGCTTTCCTTGGAGC |
| hsa-miR-203b-3p | TTGAACTGTTAAGAACCACTGGA | GCGCCCGCCGGGTCTAGTGGTCCTAAACATTTCACAATTGCGCTACAGAACTGTTGAACTGTTAAGAACCACTGGACCCAGCGCGC |
| hsa-miR-4724-5p | AACTGAACCAGGAGTGAGCTTCG | ACGCAAAATGAACTGAACCAGGAGTGAGCTTCGTGTACATTATCTATTAGAAAATGAAGTACCTTCTGGTTCAGCTAGTCCCTGTGCGT |
| hsa-miR-4700-3p | CACAGGACTGACTCCTCACCCCAGTG | TCAGTGAGGTCTGGGGATGAGGACAGTGTGTCCTGAAATTCACAGGACTGACTCCTCACCCCAGTGCACGAGGA |
| hsa-miR-6843-3p | ATGGTCTCCTGTTCTCTGCAG | CCCCCATTTCCTCAGGAATAAAAGTGCAGCAGTGCCTGCTGTGGGGACAGCTGAGGGCAGTGAGGCCCTGGGGAGCTGCTGCAGGCAGCAGGTGGGCGGGACGCCAGCAGGCTGTCTAGCTGTTCCCATGATGGTCTCCTGTTCTCTGCAG |
| hsa-miR-3929 | GAGGCTGATGTGAGTAGACCACT | AGTGGCTCACACCAGTAATCCCAGCACTTTGGGAGGCTGATGTGAGTAGACCACT |
| hsa-miR-7846-3p | CAGCGGAGCCTGGAGAGAAGG | GCCCCGCCGCCTGGCCTCTGGCCCGCTGGGGCGCGGGCTTTCGCTTTCAGTCGAGGGCTAGCGAGCGCAGCGGAGCCTGGAGAGAAGGCGCTGGGC |
| hsa-miR-3921 | TCTCTGAGTACCATATGCCTTGT | CCTAGCCCAGTACAAGGCATATGGTACTCAAGAGACTTAGAAATCCCTAAGTCTCTGAGTACCATATGCCTTGTACTGGGCTAGG |
| hsa-miR-6737-3p | TCTGTGCTTCACCCCTACCCAG | TTGGGTTGGGGTGGTCGGCCCTGGAGGGGGTTTGTTTGCTTATTCCCCTCTGTGCTTCACCCCTACCCAG |
| hsa-miR-125b-1-3p | ACGGGTTAGGCTCTTGGGAGCT | TGCGCTCCTCTCAGTCCCTGAGACCCTAACTTGTGATGTTTACCGTTTAAATCCACGGGTTAGGCTCTTGGGAGCTGCGAGTCGTGCT |
| hsa-miR-4435 | ATGGCCAGAGCTCACACAGAGG | AGGCAGCAAATGGCCAGAGCTCACACAGAGGGATGAGTGCACTTCACCTGCAGTGTGACTCAGCAGGCCAACAGATGCTA |
| hsa-miR-6798-3p | CTACCCCCCATCCCCCTGTAG | GGCAGCCAGGGGGATGGGCGAGCTTGGGCCCATTCCTTTCCTTACCCTACCCCCCATCCCCCTGTAG |
| hsa-miR-1910-5p | CCAGTCCTGTGCCTGCCGCCT | TGTCCCTTCAGCCAGTCCTGTGCCTGCCGCCTTTGTGCTGTCCTTGGAGGGAGGCAGAAGCAGGATGACAATGAGGGCAA |
| hsa-miR-4769-3p | TCTGCCATCCTCCCTCCCCTAC | GAGGAGAGGTGGGATGGAGAGAAGGTATGAGCTAAAAATCCCCAAGCTCTGCCATCCTCCCTCCCCTACTTCTCCCC |
| hsa-miR-548ab | AAAAGTAATTGTGGATTTTGCT | ATGTTGGTGCAAAAGTAATTGTGGATTTTGCTATTACTTGTATTTATTTGTAATGCAAAACCCGCAATTAGTTTTGCACCAACC |
| hsa-miR-181a-3p | ACCATCGACCGTTGATTGTACC | TGAGTTTTGAGGTTGCTTCAGTGAACATTCAACGCTGTCGGTGAGTTTGGAATTAAAATCAAAACCATCGACCGTTGATTGTACCCTATGGCTAACCATCATCTACTCCA |
| hsa-miR-664a-5p | ACTGGCTAGGGAAAATGATTGGAT | GAACATTGAAACTGGCTAGGGAAAATGATTGGATAGAAACTATTATTCTATTCATTTATCCCCAGCCTACAAAATGAAAAAA |
| hsa-miR-466 | ATACACATACACGCAACACACAT | GTGTGTGTATATGTGTGTTGCATGTGTGTATATGTGTGTATATATGTACACATACACATACACGCAACACACATATATACATGC |
| hsa-miR-4425 | TGTTGGGATTCAGCAGGACCAT | GTGCTTTACATGAATGGTCCCATTGAATCCCAACAGCTTTGCGAAGTGTTGTTGGGATTCAGCAGGACCATTCGTGTAAAGTAA |
| hsa-miR-16-2-3p | CCAATATTACTGTGCTGCTTTA | GTTCCACTCTAGCAGCACGTAAATATTGGCGTAGTGAAATATATATTAAACACCAATATTACTGTGCTGCTTTAGTGTGAC |
| hsa-miR-7843-3p | ATGAAGCCTTCTCTGCCTTACG | GTAGACAGGATGAGGGCAGAGCCAGCTTCCTGATCTGATGGAAGTCATGAAGCCTTCTCTGCCTTACGCTTGGCTCTGC |
| hsa-miR-1225-5p | GTGGGTACGGCCCAGTGGGGGG | GTGGGTACGGCCCAGTGGGGGGGAGAGGGACACGCCCTGGGCTCTGCCCAGGGTGCAGCCGGACTGACTGAGCCCCTGTGCCGCCCCCAG |
| hsa-miR-181a-5p | AACATTCAACGCTGTCGGTGAGT | AGAAGGGCTATCAGGCCAGCCTTCAGAGGACTCCAAGGAACATTCAACGCTGTCGGTGAGTTTGGGATTTGAAAAAACCACTGACCGTTGACTGTACCTTGGGGTCCTTA |
| hsa-miR-4662a-3p | AAAGATAGACAATTGGCTAAAT | TCTATTTAGCCAATTGTCCATCTTTAGCTATTCTGAATGCCTAAAGATAGACAATTGGCTAAATAGA |
| hsa-miR-4766-3p | ATAGCAATTGCTCTTTTGGAA | CTGAAGCTCCTTCTGAAAGAGCAGTTGGTGTTTATTTTTTACTAAATAGCAATTGCTCTTTTGGAAGGAACTTGAG |
| hsa-miR-1245b-3p | TCAGATGATCTAAAGGCCTATA | TTTATATGTAGGCCTTTAGATCACTTAAAGAGTATTCAACATCAGATGATCTAAAGGCCTATACATAAA |
| hsa-miR-6749-3p | CTCCTCCCCTGCCTGGCCCAG | GGCCCTCGGGCCTGGGGTTGGGGGAGCTCTGTCCTGTCTCACTCATTGCTCCTCCCCTGCCTGGCCCAG |
| hsa-miR-1827 | TGAGGCAGTAGATTGAAT | TCAGCAGCACAGCCTTCAGCCTAAAGCAATGAGAAGCCTCTGAAAGGCTGAGGCAGTAGATTGAAT |
| hsa-miR-5695 | ACTCCAAGAAGAATCTAGACAG | CAAGGCCTATCTATCTAGATTCTTCTTGGCCTCTCTGAGCATGCATTCCTGAGACTCCAAGAAGAATCTAGACAGATAGGCCTTG |
| hsa-miR-1299 | TTCTGGAATTCTGTGTGAGGGA | CCTCATGGCAGTGTTCTGGAATCCTACGTGAGGGACAATCATTCAGACCCACGTAGCAGTGTTCTGGAATTCTGTGTGAGGGA |
| hsa-miR-374c-3p | CACTTAGCAGGTTGTATTATAT | ACACGGACAATGATAATACAACCTGCTAAGTGCTAGGACACTTAGCAGGTTGTATTATATCCATCCGAGT |
| hsa-miR-6131 | GGCTGGTCAGATGGGAGTG | TCCCGCATTCCCTCTGCTTTGGTCAGGTGGTGCCCTCCTTCCATGGGTAGAGCCAGAGATGGTGGGTTCTGGCTGGTCAGATGGGAGTGGACAGAGACCCGGGGTCCTC |
| hsa-miR-6845-3p | CCTCTCCTCCCTGTGCCCCAG | AACTGCGGGGCCAGAGCAGAGAGCCCTTGCACACCACCAGCCTCTCCTCCCTGTGCCCCAG |
| hsa-miR-320b | AAAAGCTGGGTTGAGAGGGCAA | ATAAATTAATCCCTCTCTTTCTAGTTCTTCCTAGAGTGAGGAAAAGCTGGGTTGAGAGGGCAAACAAATTAA |
| hsa-miR-505-5p | GGGAGCCAGGAAGTATTGATGT | GATGCACCCAGTGGGGGAGCCAGGAAGTATTGATGTTTCTGCCAGTTTAGCGTCAACACTTGCTGGTTTCCTCTCTGGAGCATC |
| hsa-miR-4729 | TCATTTATCTGTTGGGAAGCTA | TCTGTTTCCTCATTTATCTGTTGGGAAGCTAACTGTGACCTTAGCGTCCCAGCAGATAAATGAGGAAACAGA |
| hsa-miR-3085-5p | AGGTGCCATTCTGAGGGCCAGGAGT | CCCTACTCTGGGAAGGTGCCATTCTGAGGGCCAGGAGTTTGATTATGTGTCACTCTGGCTGCTATGGCCCCCTCCCAGGGTCTGG |
| hsa-miR-192-5p | CTGACCTATGAATTGACAGCC | GCCGAGACCGAGTGCACAGGGCTCTGACCTATGAATTGACAGCCAGTGCTCTCGTCTCCCCTCTGGCTGCCAATTCCATAGGTCACAGGTATGTTCGCCTCAATGCCAGC |
| hsa-miR-3664-5p | AACTCTGTCTTCACTCATGAGT | CTGTAAACTTGAAGGTAGGGAACTCTGTCTTCACTCATGAGTACCTTCCAACACGAGCTCTCAGGAGTAAAGACAGAGTTCCCTACCTTCAATGTGGAT |
| hsa-miR-548l | AAAAGTATTTGCGGGTTTTGTC | TATTAGGTTGGTGCAAAAGTATTTGCGGGTTTTGTCGTAGAAAGTAATGGCAAAAACTGCAGTTACTTGTGCACCAACCAAATGCT |
| hsa-miR-9902 | CCCAGAAATCTGGTATGCCAGC | GCAGGGAAAGGGAACCCAGAAATCTGGTATGCCAGCAAAGAGAGTAAGAACTTCTGACAAGCCAGGCTTCTGGTCTCTCTCTCTCTGTCTCTC |
| hsa-miR-6830-3p | TGTCTTTCTTCTCTCCCTTGCAG | GTGCCCCAAGGAAGGAGGCTGGACATCCCTCATCTGTTTCTCACTGGTGTCTTTCTTCTCTCCCTTGCAG |
| hsa-miR-1283 | TCTACAAAGGAAAGCGCTTTCT | CTCAAGCTATGAGTCTACAAAGGAAAGCGCTTTCTGTTGTCAGAAAGAAGAGAAAGCGCTTCCCTTTTGAGGGTTACGGTTTGAGAA |
| hsa-miR-6729-3p | TCATCCCCCTCGCCCTCTCAG | GAGGGTGGGCGAGGGCGGCTGAGCGGCTCCATCCCCCGGCCTGCTCATCCCCCTCGCCCTCTCAG |
| hsa-miR-5006-3p | TTTCCCTTTCCATCCTGGCAG | AACCATTAGGGGGCTGTGGTTTGCCAGGGCAGGAGGTGGAAGGGAGCCCCATTTACAGTGGTAACTTCCTTTCCCTTTCCATCCTGGCAGGCTTCAGAGAACTTTACCAG |
| hsa-miR-5689 | AGCATACACCTGTAGTCCTAGA | AGCGTGGTAGCATACACCTGTAGTCCTAGATACTCAGGAGGGTGAGTATCTAGGACTACAGGTGTGTGCTACCACGCT |
| hsa-miR-130b-3p | CAGTGCAATGATGAAAGGGCAT | GGCCTGCCCGACACTCTTTCCCTGTTGCACTACTATAGGCCGCTGGGAAGCAGTGCAATGATGAAAGGGCATCGGTCAGGTC |
| hsa-let-7c-5p | TGAGGTAGTAGGTTGTATGGTT | GCATCCGGGTTGAGGTAGTAGGTTGTATGGTTTAGAGTTACACCCTGGGAGTTAACTGTACAACCTTCTAGCTTTCCTTGGAGC |
| hsa-miR-548h-3p | CAAAAACCGCAATTACTTTTGCA | AAGTATTAAGTTGGTGCAAAAGTAATTGAGATTTTTGCTACTGAAAGTAATGGCAAAAACCGCAATTACTTTTGCACCAACCTAATAGATGCCAATG |
| hsa-miR-203a-3p | GTGAAATGTTTAGGACCACTAG | GTGTTGGGGACTCGCGCGCTGGGTCCAGTGGTTCTTAACAGTTCAACAGTTCTGTAGCGCAATTGTGAAATGTTTAGGACCACTAGACCCGGCGGGCGCGGCGACAGCGA |
| hsa-miR-377-3p | ATCACACAAAGGCAACTTTTGT | TTGAGCAGAGGTTGCCCTTGGTGAATTCGCTTTATTTATGTTGAATCACACAAAGGCAACTTTTGTTTG |
| hsa-miR-6783-3p | TTCCTGGGCTTCTCCTCTGTAG | CCTGTTAGGGGAAAAGTCCTGATCCGGGAACCCACAGCCCCGTTCCTGGGCTTCTCCTCTGTAG |
| hsa-miR-498-3p | AAAGCACCTCCAGAGCTTGAAGC | AACCCTCCTTGGGAAGTGAAGCTCAGGCTGTGATTTCAAGCCAGGGGGCGTTTTTCTATAACTGGATGAAAAGCACCTCCAGAGCTTGAAGCTCACAGTTTGAGAGCAATCGTCTAAGGAAGTT |
| hsa-miR-374c-5p | ATAATACAACCTGCTAAGTGCT | ACACGGACAATGATAATACAACCTGCTAAGTGCTAGGACACTTAGCAGGTTGTATTATATCCATCCGAGT |
| hsa-miR-199a-3p | ACAGTAGTCTGCACATTGGTTA | CCAGAGGACACCTCCACTCCGTCTACCCAGTGTTTAGACTATCTGTTCAGGACTCCCAAATTGTACAGTAGTCTGCACATTGGTTAGGCTGGGCTGGGTTAGACCCTCGG |
| hsa-miR-6165 | CAGCAGGAGGTGAGGGGAG | CAGCAGGTCAGCAGGAGGTGAGGGGAGAGGATCCACCTGTCCTGTCCTGTCCTCTCCTGCCCTGTCCTGGCTCCAGCCCCTCCC |
| hsa-miR-454-3p | TAGTGCAATATTGCTTATAGGGT | TCTGTTTATCACCAGATCCTAGAACCCTATCAATATTGTCTCTGCTGTGTAAATAGTTCTGAGTAGTGCAATATTGCTTATAGGGTTTTGGTGTTTGGAAAGAACAATGGGCAGG |
| hsa-miR-4639-3p | TCACTCTCACCTTGCTTTGC | TTGCTAAGTAGGCTGAGATTGATGTCAGGTTATCCCCAAGCATAACCTCACTCTCACCTTGCTTTGCAG |
| hsa-miR-1278 | TAGTACTGTGCATATCATCTAT | ATTTGCTCATAGATGATATGCATAGTACTCCCAGAACTCATTAAGTTGGTAGTACTGTGCATATCATCTATGAGCGAATAG |
| hsa-miR-5699-3p | TCCTGTCTTTCCTTGTTGGAGC | CTGTACCCCTGCCCCAACAAGGAAGGACAAGAGGTGTGAGCCACACACACGCCTGGCCTCCTGTCTTTCCTTGTTGGAGCAGGGATGTAG |
| hsa-miR-6746-3p | CAGCCGCCGCCTGTCTCCACAG | CTTGCCCGGGAGAAGGAGGTGGCCTGGAGAGCTGCTGTCTCCAGCCGCCGCCTGTCTCCACAG |
| hsa-miR-6720-3p | CGCGCCTGCAGGAACTGGTAGA | TTGAGCGAGAGATTGTGGCGCACCGAGTTCTTCCAGCCCTGGTAGGCGCCGCGGAAGAAGGGGAAGCGCGCCTGCAGGAACTGGTAGATCTCGCTGAG |
| hsa-miR-874-5p | CGGCCCCACGCACCAGGGTAAGA | TTAGCCCTGCGGCCCCACGCACCAGGGTAAGAGAGACTCTCGCTTCCTGCCCTGGCCCGAGGGACCGACTGGCTGGGC |
| hsa-miR-6505-3p | TGACTTCTACCTCTTCCAAAG | GCATTGGAATAGGGGATATCTCAGCATGTTGAGCCCTGTCTCTGGGGAGCTGACTTCTACCTCTTCCAAAG |
| hsa-miR-1973 | ACCGTGCAAAGGTAGCATA | TATGTTCAACGGCCATGGTATCCTGACCGTGCAAAGGTAGCATA |
| hsa-miR-4473 | CTAGTGCTCTCCGTTACAAGTA | AAGGAACAGGGGACACTTGTAATGGAGAACACTAAGCTATGGACTGCTATGGACTGCTAGTGCTCTCCGTTACAAGTATCCCCTGTTACCT |
| hsa-miR-3129-3p | AAACTAATCTCTACACTGCTGC | GTACTTGGGCAGTAGTGTAGAGATTGGTTTGCCTGTTAATGAATTCAAACTAATCTCTACACTGCTGCCCAAGAGC |
| hsa-miR-6866-5p | TTAGAGGCTGGAATAGAGATTCT | CCATTTTAGAGGCTGGAATAGAGATTCTTGAGGCTTGGAAGAGTAAGGATCCCTTTATCTGTCCTCTAG |
| hsa-miR-6888-5p | AAGGAGATGCTCAGGCAGAT | GTGGGAAGGAGATGCTCAGGCAGATCTGTCTCTGATTGTTTCCAAGATCTGTCTCGATTGTTTCCAG |
| hsa-miR-107 | AGCAGCATTGTACAGGGCTATCA | CTCTCTGCTTTCAGCTTCTTTACAGTGTTGCCTTGTGGCATGGAGTTCAAGCAGCATTGTACAGGGCTATCAAAGCACAGA |
| hsa-miR-5003-5p | TCACAACAACCTTGCAGGGTAGA | ATGAGTTTGCTTTGTGTCATCCTCACAACAACCTTGCAGGGTAGAGATGATTTTTCCTACTTTTCTAGGTTGTTGGGGGCTGGGGCAGGGGGAACAGAG |
| hsa-miR-4804-5p | TTGGACGGTAAGGTTAAGCAA | TCAGTGTATTTGGACGGTAAGGTTAAGCAAGGTGCGTCGTATCTTGCTTAACCTTGCCCTCGAAATACACTGA |
| hsa-miR-6507-5p | GAAGAATAGGAGGGACTTTGT | GGAGGGAAGAATAGGAGGGACTTTGTATTGTGGTTCAGTACCATGCAAAGTCCTTCCTATTTTTCCCTCC |
| hsa-miR-6873-3p | TTCTCTCTGTCTTTCTCTCTCAG | CCCAGCAGAGGGAATACAGAGGGCAATCAGGACTGGGTCATTCTCTCTGTCTTTCTCTCTCAG |
| hsa-miR-4733-3p | CCACCAGGTCTAGCATTGGGAT | GGTCGCTTAAATCCCAATGCTAGACCCGGTGGCAATCAAGGTCTAGCCACCAGGTCTAGCATTGGGATTTAAGCCC |
| hsa-miR-4775 | TTAATTTTTTGTTTCGGTCACT | ATTAAGCTTTTAATTTTTTGTTTCGGTCACTCTTGATAGCAGACATTGACTGAAACAAAAAATTAAAAGCTTTAT |
| hsa-miR-4671-3p | TTAGTGCATAGTCTTTGGTCT | TATTTTAAGACCGAAGACTGTGCGCTAATCTCTTAGCACTGAAGATTAGTGCATAGTCTTTGGTCTCAAAATA |
| hsa-miR-7845-5p | AAGGGACAGGGAGGGTCGTGG | GCAAGGGACAGGGAGGGTCGTGGCGACACTCGCGCCAGCTCCCGGGACGGCTGGGCTCGGGCTGGTCGCCGACCTCCGACCCTCCACTAGATGCCTGGC |
| hsa-miR-933 | TGTGCGCAGGGAGACCTCTCCC | ACTTGGGTCAGTTCAGAGGTCCTCGGGGCGCGCGTCGAGTCAGCCGTGTGCGCAGGGAGACCTCTCCCACCCACAGT |
| hsa-miR-518c-3p | CAAAGCGCTTCTCTTTAGAGTGT | GCGAGAAGATCTCATGCTGTGACTCTCTGGAGGGAAGCACTTTCTGTTGTCTGAAAGAAAACAAAGCGCTTCTCTTTAGAGTGTTACGGTTTGAGAAAAGC |
| hsa-miR-3940-3p | CAGCCCGGATCCCAGCCCACTT | GCTTATCGAGGAAAAGATCGAGGTGGGTTGGGGCGGGCTCTGGGGATTTGGTCTCACAGCCCGGATCCCAGCCCACTTACCTTGGTTACTCTCCTTCCTTCT |
| hsa-miR-4666a-3p | CATACAATCTGACATGTATTT | ATCACTTAAATACATGTCAGATTGTATGCCTACAAAATCCCTCCAGACTGGCATACAATCTGACATGTATTTAAGAGAT |
| hsa-miR-218-2-3p | CATGGTTCTGTCAAGCACCGCG | GACCAGTCGCTGCGGGGCTTTCCTTTGTGCTTGATCTAACCATGTGGTGGAACGATGGAAACGGAACATGGTTCTGTCAAGCACCGCGGAAAGCACCGTGCTCTCCTGCA |
| hsa-miR-6742-3p | ACCTGGGTTGTCCCCTCTAG | GAGGGAGTGGGGTGGGACCCAGCTGTTGGCCATGGCGACAACACCTGGGTTGTCCCCTCTAG |
| hsa-miR-513c-3p | TAAATTTCACCTTTCTGAGAAGA | GCGTACAGTGCCTTTCTCAAGGAGGTGTCGTTTATGTGAACTAAAATATAAATTTCACCTTTCTGAGAAGAGTAATGTACAGCA |
| hsa-miR-212-5p | ACCTTGGCTCTAGACTGCTTACT | CGGGGCACCCCGCCCGGACAGCGCGCCGGCACCTTGGCTCTAGACTGCTTACTGCCCGGGCCGCCCTCAGTAACAGTCTCCAGTCACGGCCACCGACGCCTGGCCCCGCC |
| hsa-miR-519c-5p | CTCTAGAGGGAAGCGCTTTCTG | CATGCTGTGACCCTCTAGAGGGAAGCGCTTTCTGTTGTCTGAAAGAAAAGAAAGTGCATCCTTTTAGAGGTTTACTGTTTG |
| hsa-miR-6796-3p | GAAGCTCTCCCCTCCCCGCAG | TTACCTTGTGGGGTTGGAGAGCTGGCTGGTCCAGCCCCTCAGAAGCTCTCCCCTCCCCGCAG |
| hsa-miR-889-3p | TTAATATCGGACAACCATTGT | GTGCTTAAAGAATGGCTGTCCGTAGTATGGTCTCTATATTTATGATGATTAATATCGGACAACCATTGTTTTAGTATCC |
| hsa-miR-6732-3p | TAACCCTGTCCTCTCCCTCCCAG | AGGCCTAGGGGGTGGCAGGCTGGCCATCAGTGTGGGCTAACCCTGTCCTCTCCCTCCCAG |
| hsa-miR-15a-3p | CAGGCCATATTGTGCTGCCTCA | CCTTGGAGTAAAGTAGCAGCACATAATGGTTTGTGGATTTTGAAAAGGTGCAGGCCATATTGTGCTGCCTCAAAAATACAAGG |
| hsa-miR-3614-3p | TAGCCTTCAGATCTTGGTGTTTT | GGTTCTGTCTTGGGCCACTTGGATCTGAAGGCTGCCCCTTTGCTCTCTGGGGTAGCCTTCAGATCTTGGTGTTTTGAATTCTTACT |
| hsa-miR-574-5p | TGAGTGTGTGTGTGTGAGTGTGT | GGGACCTGCGTGGGTGCGGGCGTGTGAGTGTGTGTGTGTGAGTGTGTGTCGCTCCGGGTCCACGCTCATGCACACACCCACACGCCCACACTCAGG |
| hsa-miR-33a-3p | CAATGTTTCCACAGTGCATCAC | CTGTGGTGCATTGTAGTTGCATTGCATGTTCTGGTGGTACCCATGCAATGTTTCCACAGTGCATCACAG |
| hsa-miR-1226-5p | GTGAGGGCATGCAGGCCTGGATGGGG | GTGAGGGCATGCAGGCCTGGATGGGGCAGCTGGGATGGTCCAAAAGGGTGGCCTCACCAGCCCTGTGTTCCCTAG |
| hsa-miR-5580-3p | CACATATGAAGTGAGCCAGCAC | TGCTGGCTCATTTCATATGTGTGCTGAGAAAATTCACACATATGAAGTGAGCCAGCAC |
| hsa-miR-4657 | AATGTGGAAGTGGTCTGAGGCAT | AATGTGGAAGTGGTCTGAGGCATATAGAGTATATGCCAAGAACACTACCATAT |
| hsa-miR-3610 | GAATCGGAAAGGAGGCGCCG | AAGAGCCGCGGCGTAACGGCAGCCATCTTGTTTGTTTGAGTGAATCGGAAAGGAGGCGCCGGCTGTGGCGGCG |
| hsa-miR-4767 | CGCGGGCGCTCCTGGCCGCCGCC | ACATGGGCCCGCGGGCGCTCCTGGCCGCCGCCCGACTTCGGGGCCAGCCGGGGGCAGAGCGCGCGGGAGCCCGAGCGT |
| hsa-miR-296-3p | GAGGGTTGGGTGGAGGCTCTCC | AGGACCCTTCCAGAGGGCCCCCCCTCAATCCTGTTGTGCCTAATTCAGAGGGTTGGGTGGAGGCTCTCCTGAAGGGCTCT |
| hsa-miR-655-5p | AGAGGTTATCCGTGTTATGTTC | AACTATGCAAGGATATTTGAGGAGAGGTTATCCGTGTTATGTTCGCTTCATTCATCATGAATAATACATGGTTAACCTCTTTTTGAATATCAGACTC |
| hsa-miR-6720-5p | TTCCAGCCCTGGTAGGCGCCGCG | TTGAGCGAGAGATTGTGGCGCACCGAGTTCTTCCAGCCCTGGTAGGCGCCGCGGAAGAAGGGGAAGCGCGCCTGCAGGAACTGGTAGATCTCGCTGAG |
| hsa-miR-4423-3p | ATAGGCACCAAAAAGCAACAA | ATCATGTACTGCAGTTGCCTTTTTGTTCCCATGCTGTTTAAGCCTAGCATAGGCACCAAAAAGCAACAACAGTATGTGAA |
| hsa-miR-877-3p | TCCTCTTCTCCCTCCTCCCAG | GTAGAGGAGATGGCGCAGGGGACACGGGCAAAGACTTGGGGGTTCCTGGGACCCTCAGACGTGTGTCCTCTTCTCCCTCCTCCCAG |
| hsa-miR-519c-5p | CTCTAGAGGGAAGCGCTTTCTG | TCTCAGCCTGTGACCCTCTAGAGGGAAGCGCTTTCTGTTGTCTGAAAGAAAAGAAAGTGCATCTTTTTAGAGGATTACAGTTTGAGA |
| hsa-miR-6752-3p | TCCCTGCCCCCATACTCCCAG | ATGGAGGGGGGTGTGGAGCCAGGGGGCCCAGGTCTACAGCTTCTCCCCGCTCCCTGCCCCCATACTCCCAG |
| hsa-miR-5008-3p | CCTGTGCTCCCAGGGCCTCGC | GGGCTGACCCCTAGGGTCAGGTGAGGCCCTTGGGGCACAGTGGTGCCATCTCCCCTGTGCTCCCAGGGCCTCGCCTGTCCCTTGAGGTCGGCCC |
| hsa-miR-27b-3p | TTCACAGTGGCTAAGTTCTGC | ACCTCTCTAACAAGGTGCAGAGCTTAGCTGATTGGTGAACAGTGATTGGTTTCCGCTTTGTTCACAGTGGCTAAGTTCTGCACCTGAAGAGAAGGTG |
| hsa-miR-208b-3p | ATAAGACGAACAAAAGGTTTGT | CCTCTCAGGGAAGCTTTTTGCTCGAATTATGTTTCTGATCCGAATATAAGACGAACAAAAGGTTTGTCTGAGGGCAG |
| hsa-miR-487b-5p | GTGGTTATCCCTGTCCTGTTCG | TTGGTACTTGGAGAGTGGTTATCCCTGTCCTGTTCGTTTTGCTCATGTCGAATCGTACAGGGTCATCCACTTTTTCAGTATCAA |
| hsa-miR-649 | AAACCTGTGTTGTTCAAGAGTC | GGCCTAGCCAAATACTGTATTTTTGATCGACATTTGGTTGAAAAATATCTATGTATTAGTAAACCTGTGTTGTTCAAGAGTCCACTGTGTTTTGCTG |
| hsa-miR-326 | CCTCTGGGCCCTTCCTCCAG | CTCATCTGTCTGTTGGGCTGGAGGCAGGGCCTTTGTGAAGGCGGGTGGTGCTCAGATCGCCTCTGGGCCCTTCCTCCAGCCCCGAGGCGGATTCA |
| hsa-miR-24-2-5p | TGCCTACTGAGCTGAAACACAG | CTCTGCCTCCCGTGCCTACTGAGCTGAAACACAGTTGGTTTGTGTACACTGGCTCAGTTCAGCAGGAACAGGG |
| hsa-miR-548g-5p | TGCAAAAGTAATTGCAGTTTTTG | AGTTATTAGATTAGTGCAAAAGTAATTGCAGTTTTTGCATTACGTTCTATGGCAAAACTGTAATTACTTTTGTACCAACATAATACTTC |
| hsa-miR-1185-1-3p | ATATACAGGGGGAGACTCTTAT | TTTGGTACTTGAAGAGAGGATACCCTTTGTATGTTCACTTGATTAATGGCGAATATACAGGGGGAGACTCTTATTTGCGTATCAAA |
| hsa-miR-4800-5p | AGTGGACCGAGGAAGGAAGGA | GGAGAAAGGAGTGGACCGAGGAAGGAAGGAAGGCAAGGCTGTCTGTCCATCCGTCCGTCTGTCCACCTACCTGTCAGTCC |
| hsa-miR-6846-3p | TGACCCCTTCTGTCTCCCTAG | CAGGCTGGGGGCTGGATGGGGTAGAGTAGGAGAGCCCACTGACCCCTTCTGTCTCCCTAG |
| hsa-miR-3529-3p | AACAACAAAATCACTAGTCTTCCA | GGCACCATTAGGTAGACTGGGATTTGTTGTTGAGCGCAGTAAGACAACAACAAAATCACTAGTCTTCCAGATGGGGCC |
| hsa-miR-4661-5p | AACTAGCTCTGTGGATCCTGAC | TTTACTCTGAACTAGCTCTGTGGATCCTGACAGACAGCCTGATAGACAGGATCCACAGAGCTAGTCCAGAGTAAA |
| hsa-miR-6817-5p | TCTGCCATAGGAAGCTTGGAGTGG | AGGATTCTGCCATAGGAAGCTTGGAGTGGAACTGACCTGCCCCCTTTCTCTCTGACTCCATGGCAG |
| hsa-miR-6754-3p | TCTTCACCTGCCTCTGCCTGCA | GGCTGCCAGGGAGGCTGGTTTGGAGGAGTCTGGTGGCCTGTTCTCTTCACCTGCCTCTGCCTGCAG |
| hsa-miR-16-5p | TAGCAGCACGTAAATATTGGCG | GTCAGCAGTGCCTTAGCAGCACGTAAATATTGGCGTTAAGATTCTAAAATTATCTCCAGTATTAACTGTGCTGCTGAAGTAAGGTTGAC |
| hsa-miR-3159 | TAGGATTACAAGTGTCGGCCAC | CCAAAGTCCTAGGATTACAAGTGTCGGCCACGGGCTGGGCACAGTGGCTCACGCCTGTAATCCCAGCATTTTGG |
| hsa-miR-1302 | TTGGGACATACTTATGCTAAA | CAGAAAGCCCAGTTAAATTTGAATTTCAAGTAAACAATGAATAATTGTGTATGTAAGAATATCCCATACAATATTTGGGACATACTTATGCTAAAAATTATTCCTTGCTTATCTGAAATTCAAATGTAACTAGGATTCCTGTA |
| hsa-miR-558 | TGAGCTGCTGTACCAAAAT | GTGTGTGTGTGTGTGTGTGGTTATTTTGGTATAGTAGCTCTAGACTCTATTATAGTTTCCTGAGCTGCTGTACCAAAATACCACAAACGGGCTG |
| hsa-miR-217-5p | TACTGCATCAGGAACTGATTGGA | AGTATAATTATTACATAGTTTTTGATGTCGCAGATACTGCATCAGGAACTGATTGGATAAGAATCAGTCACCATCAGTTCCTAATGCATTGCCTTCAGCATCTAAACAAG |
| hsa-miR-514b-3p | ATTGACACCTCTGTGAGTGGA | CATGTGGTACTCTTCTCAAGAGGGAGGCAATCATGTGTAATTAGATATGATTGACACCTCTGTGAGTGGAGTAACACATG |
| hsa-miR-4723-5p | TGGGGGAGCCATGAGATAAGAGCA | AGTTGGTGGGGGAGCCATGAGATAAGAGCACCTCCTAGAGAATGTTGAACTAAAGGTGCCCTCTCTGGCTCCTCCCCAAAG |
| hsa-miR-4785 | AGAGTCGGCGACGCCGCCAGC | GTAGGTGGGGACGCGGCGGCGCTGCTCCTCCGCTGCCGCCGGGAGAGTCGGCGACGCCGCCAGCTCCGCGCGC |
| hsa-miR-7706 | TGAAGCGCCTGTGCTCTGCCGAGA | TGGAGCTGTGTGCAGGGCCAGCGCGGAGCCCGAGCAGCCGCGGTGAAGCGCCTGTGCTCTGCCGAGA |
| hsa-miR-512-5p | CACTCAGCCTTGAGGGCACTTTC | TCTCAGTCTGTGGCACTCAGCCTTGAGGGCACTTTCTGGTGCCAGAATGAAAGTGCTGTCATAGCTGAGGTCCAATGACTGAGG |
| hsa-miR-1976 | CCTCCTGCCCTCCTTGCTGT | GCAGCAAGGAAGGCAGGGGTCCTAAGGTGTGTCCTCCTGCCCTCCTTGCTGT |
| hsa-miR-191-5p | CAACGGAATCCCAAAAGCAGCTG | CGGCTGGACAGCGGGCAACGGAATCCCAAAAGCAGCTGTTGTCTCCAGAGCATTCCAGCTGCGCTTGGATTTCGTCCCCTGCTCTCCTGCCT |
