# Supplementary material for: Genome-Wide Expression Difference of MicroRNAs in Basal Cell Carcinoma
Source: J Immunol Res. 2021 Aug 4;2021:7223500. doi: 10.1155/2021/7223500 (PMC8357504; doi:10.1155/2021/7223500)
Supplement: Supplementary Materials — Supplementary Figure 1: length distribution of sRNA tags in six sequencing libraries. Supplementary Figure 2: KEGG pathway of basal cell carcinoma and 24 miRNA-regulated gene members marked in a red box. Supplementary Table 1: information of TaqMan probes used in quantitative PCR. Supplementary Table 2: list of miRNAs identified in this study. Supplementary Table 3: differentially expressed miRNAs (DEMs) in the BCCs when compared with control. Supplementary Table 4: list of the enriched “biological process” GO terms of targeted genes of DEMs between the control and BCC groups. Supplementary Table 5: miRNA-targeted gene analysis in the basal cell carcinoma (ko05217) pathway. u: upregulation; d: downregulation. [file 7223500.f1.zip › 7223500.f5.docx]

| **Table S3. Differentially expressed miRNAs (DEMs)** | | | | | |
| --- | --- | --- | --- | --- | --- |
| **miRNA id** | **TPM(CK)** | **TPM(SC)** | **log2Ratio(BCC/Control)** | **Up / down regulation** | **FDR** |
| hsa-miR-2113 | 0.01 | 4.43 | 7.920806471 | UP | 6.31E-61 |
| hsa-miR-1269b | 0.05 | 16.55 | 7.881966729 | UP | 6.94E-233 |
| hsa-miR-135a-3p | 0 | 0.82 | 6.505768972 | UP | 6.28E-14 |
| hsa-miR-551a | 0.05 | 3.65 | 5.664967567 | UP | 3.71E-57 |
| hsa-miR-548y | 0.04 | 1.39 | 5.257089859 | UP | 1.91E-22 |
| hsa-miR-548i | 0.01 | 0.56 | 4.920806471 | UP | 9.51E-10 |
| hsa-miR-9983-3p | 0 | 0.26 | 4.84680589 | UP | 1.87E-05 |
| hsa-miR-3180-3p | 0.23 | 4.48 | 3.851265538 | UP | 8.58E-60 |
| hsa-miR-675-5p | 0.48 | 8.02 | 3.621246189 | UP | 4.43E-102 |
| hsa-miR-542-3p | 0.07 | 0.9 | 3.276950281 | UP | 8.15E-12 |
| hsa-miR-550a-3p | 0.04 | 0.5 | 3.223369241 | UP | 2.01E-07 |
| hsa-miR-503-3p | 0.09 | 0.79 | 3.109840296 | UP | 2.76E-10 |
| hsa-miR-130b-5p | 1.23 | 12.37 | 2.939915294 | UP | 2.74E-132 |
| hsa-miR-96-5p | 541.37 | 12773.57 | 2.78979494 | UP | 0 |
| hsa-miR-6815-5p | 0.25 | 2.07 | 2.676880888 | UP | 1.68E-21 |
| hsa-miR-5695 | 0.04 | 0.33 | 2.537477832 | UP | 0.000192403 |
| hsa-miR-4662a-3p | 0.05 | 0.4 | 2.505768972 | UP | 2.92E-05 |
| hsa-miR-4708-3p | 0.05 | 0.41 | 2.456859372 | UP | 4.64E-05 |
| hsa-miR-1284 | 0.63 | 4.35 | 2.447785788 | UP | 4.56E-39 |
| hsa-miR-431-3p | 0.08 | 0.57 | 2.335843971 | UP | 4.19E-06 |
| hsa-miR-584-5p | 4.77 | 29.65 | 2.19819617 | UP | 3.62E-221 |
| hsa-miR-941 | 4.85 | 27.47 | 2.127969615 | UP | 5.25E-203 |
| hsa-miR-320d | 0.86 | 4.88 | 2.090731473 | UP | 9.63E-37 |
| hsa-miR-615-3p | 4.06 | 22.06 | 1.995708455 | UP | 3.24E-148 |
| hsa-miR-509-5p | 0.08 | 0.42 | 1.871896871 | UP | 0.000470075 |
| hsa-miR-30c-1-3p | 11.05 | 51.4 | 1.811416897 | UP | 1.49E-302 |
| hsa-miR-320b | 7.59 | 34.22 | 1.775954652 | UP | 2.28E-199 |
| hsa-miR-125a-3p | 2.03 | 8.78 | 1.720341889 | UP | 6.10E-50 |
| hsa-miR-483-3p | 0.09 | 0.41 | 1.69841405 | UP | 0.000815254 |
| hsa-miR-6766-3p | 0.3 | 1.21 | 1.682294385 | UP | 4.16E-08 |
| hsa-miR-25-5p | 0.61 | 2.42 | 1.648347053 | UP | 3.09E-14 |
| hsa-miR-335-5p | 13.39 | 54.84 | 1.639905645 | UP | 7.93E-286 |
| hsa-miR-3912-3p | 0.22 | 0.88 | 1.598878376 | UP | 5.82E-06 |
| hsa-miR-32-5p | 10.81 | 42.15 | 1.550246155 | UP | 8.79E-203 |
| hsa-miR-3913-3p | 0.25 | 0.98 | 1.520875864 | UP | 3.69E-06 |
| hsa-miR-1247-3p | 0.13 | 0.47 | 1.516416216 | UP | 0.000996478 |
| hsa-miR-328-3p | 14.46 | 54.23 | 1.507448803 | UP | 8.64E-241 |
| hsa-miR-320c | 6.25 | 22.49 | 1.442357626 | UP | 9.42E-98 |
| hsa-miR-4636 | 1.1 | 3.8 | 1.429523948 | UP | 8.83E-18 |
| hsa-miR-3690 | 0.16 | 0.54 | 1.424848977 | UP | 0.000790325 |
| hsa-miR-1910-5p | 0.17 | 0.57 | 1.37146788 | UP | 0.000822693 |
| hsa-miR-508-5p | 2.93 | 9.89 | 1.322794034 | UP | 3.86E-37 |
| hsa-miR-30b-3p | 7.85 | 25.39 | 1.292003548 | UP | 3.15E-93 |
| hsa-miR-142-5p | 6.77 | 22.17 | 1.282296903 | UP | 3.15E-80 |
| hsa-miR-935 | 0.74 | 2.4 | 1.276950281 | UP | 3.89E-10 |
| hsa-miR-130a-3p | 22.08 | 71.2 | 1.25361867 | UP | 5.34E-244 |
| hsa-miR-151b | 3.04 | 9.27 | 1.207369413 | UP | 1.10E-31 |
| hsa-miR-130a-5p | 0.36 | 1.07 | 1.129393093 | UP | 0.000102996 |
| hsa-miR-509-3-5p | 25.92 | 76.05 | 1.105584437 | UP | 1.80E-207 |
| hsa-miR-513b-5p | 2.68 | 7.66 | 1.083773922 | UP | 7.74E-22 |
| hsa-miR-340-3p | 1.89 | 5.04 | 1.021908666 | UP | 7.05E-14 |
| hsa-miR-585-3p | 0.71 | 1.93 | 1.017379731 | UP | 2.76E-06 |
| hsa-miR-1843 | 3.45 | 9.02 | 1.016869193 | UP | 1.74E-23 |
| hsa-miR-133a-3p | 9.78 | 6.64 | -1.007014782 | DOWN | 2.37E-33 |
| hsa-miR-561-5p | 0.74 | 0.48 | -1.012556336 | DOWN | 0.000505097 |
| hsa-miR-3529-3p | 94.56 | 60.57 | -1.017647441 | DOWN | 4.13E-293 |
| hsa-miR-223-5p | 1.93 | 1.2 | -1.050214462 | DOWN | 4.59E-08 |
| hsa-miR-21-3p | 36.38 | 23.25 | -1.07897997 | DOWN | 7.03E-133 |
| hsa-miR-338-5p | 3.45 | 2.2 | -1.084818078 | DOWN | 2.76E-14 |
| hsa-miR-27b-5p | 16.83 | 10.5 | -1.101122633 | DOWN | 1.35E-63 |
| hsa-miR-598-3p | 12.48 | 7.61 | -1.129591381 | DOWN | 5.81E-49 |
| hsa-miR-6516-5p | 0.94 | 0.59 | -1.138087218 | DOWN | 2.44E-05 |
| hsa-miR-29c-5p | 32.25 | 19.37 | -1.154446798 | DOWN | 9.50E-130 |
| hsa-miR-24-2-5p | 1.72 | 1.04 | -1.16098762 | DOWN | 1.34E-08 |
| hsa-miR-27a-5p | 8.41 | 4.84 | -1.188544643 | DOWN | 2.12E-36 |
| hsa-miR-18a-5p | 12.12 | 7.08 | -1.199628096 | DOWN | 3.39E-53 |
| hsa-miR-942-5p | 1.94 | 1.12 | -1.203440956 | DOWN | 6.04E-10 |
| hsa-miR-3934-5p | 1.23 | 0.67 | -1.232998865 | DOWN | 5.36E-07 |
| hsa-miR-1303 | 1.19 | 0.67 | -1.242423878 | DOWN | 7.02E-07 |
| hsa-miR-205-3p | 13.29 | 7.46 | -1.243833927 | DOWN | 1.65E-61 |
| hsa-miR-592 | 2 | 1.11 | -1.254900883 | DOWN | 6.00E-11 |
| hsa-miR-629-3p | 1.02 | 0.57 | -1.27238831 | DOWN | 2.23E-06 |
| hsa-miR-382-5p | 9.76 | 5.4 | -1.27793686 | DOWN | 2.19E-47 |
| hsa-miR-370-3p | 5.36 | 2.83 | -1.332666321 | DOWN | 2.98E-28 |
| hsa-miR-3651 | 0.42 | 0.22 | -1.355317934 | DOWN | 0.000912603 |
| hsa-miR-511-3p | 0.62 | 0.33 | -1.370747975 | DOWN | 5.87E-05 |
| hsa-miR-454-3p | 21.16 | 10.84 | -1.392932226 | DOWN | 1.99E-116 |
| hsa-miR-299-5p | 3.14 | 1.63 | -1.419980651 | DOWN | 5.51E-19 |
| hsa-miR-7976 | 0.65 | 0.33 | -1.430868967 | DOWN | 2.45E-05 |
| hsa-miR-652-5p | 1.57 | 0.76 | -1.439940873 | DOWN | 2.70E-10 |
| hsa-miR-135b-3p | 0.97 | 0.48 | -1.441763608 | DOWN | 3.75E-07 |
| hsa-miR-624-5p | 6.59 | 3.17 | -1.453033712 | DOWN | 6.03E-39 |
| hsa-miR-188-5p | 8.89 | 4.4 | -1.460373702 | DOWN | 5.03E-54 |
| hsa-miR-641 | 0.42 | 0.2 | -1.500657297 | DOWN | 0.000473575 |
| hsa-miR-134-5p | 9.3 | 4.47 | -1.503980437 | DOWN | 2.45E-57 |
| hsa-miR-4677-5p | 0.72 | 0.34 | -1.512152936 | DOWN | 4.47E-06 |
| hsa-miR-548k | 8.43 | 3.84 | -1.534912839 | DOWN | 7.83E-54 |
| hsa-miR-34c-3p | 2.43 | 1.13 | -1.535422715 | DOWN | 7.97E-17 |
| hsa-miR-1268b | 0.89 | 0.41 | -1.538625147 | DOWN | 3.34E-07 |
| hsa-miR-766-3p | 4.64 | 2.1 | -1.559321665 | DOWN | 1.14E-30 |
| hsa-miR-103a-2-5p | 6.24 | 2.77 | -1.57661842 | DOWN | 2.38E-41 |
| hsa-miR-148a-5p | 6.01 | 2.64 | -1.599827475 | DOWN | 3.22E-41 |
| hsa-miR-3176 | 0.78 | 0.34 | -1.623514045 | DOWN | 9.58E-07 |
| hsa-miR-944 | 29.02 | 12.45 | -1.626011433 | DOWN | 5.40E-195 |
| hsa-miR-203b-3p | 0.36 | 0.15 | -1.642129723 | DOWN | 0.00050549 |
| hsa-miR-6501-5p | 0.48 | 0.2 | -1.664156029 | DOWN | 5.03E-05 |
| hsa-miR-184 | 0.85 | 0.36 | -1.677961829 | DOWN | 1.17E-07 |
| hsa-miR-4484 | 0.66 | 0.27 | -1.693903373 | DOWN | 2.22E-06 |
| hsa-miR-486-5p | 15.73 | 6.29 | -1.737690032 | DOWN | 7.60E-116 |
| hsa-miR-329-3p | 0.64 | 0.26 | -1.738156611 | DOWN | 1.86E-06 |
| hsa-miR-539-5p | 0.37 | 0.15 | -1.749044927 | DOWN | 0.000187721 |
| hsa-miR-362-5p | 37.52 | 14.97 | -1.749283669 | DOWN | 2.13E-279 |
| hsa-miR-4488 | 0.52 | 0.19 | -1.763691703 | DOWN | 2.56E-05 |
| hsa-miR-6734-5p | 0.35 | 0.14 | -1.779633247 | DOWN | 0.000259254 |
| hsa-miR-3200-3p | 0.92 | 0.37 | -1.788144747 | DOWN | 9.40E-09 |
| hsa-miR-502-3p | 19.54 | 7.61 | -1.791068142 | DOWN | 1.41E-152 |
| hsa-miR-548o-3p | 0.74 | 0.29 | -1.816159123 | DOWN | 1.29E-07 |
| hsa-miR-10b-3p | 1.31 | 0.49 | -1.856801107 | DOWN | 3.15E-12 |
| hsa-miR-3194-3p | 0.54 | 0.19 | -1.87916892 | DOWN | 5.34E-06 |
| hsa-miR-494-3p | 1.18 | 0.42 | -1.906356932 | DOWN | 1.08E-11 |
| hsa-miR-618 | 0.53 | 0.21 | -1.915694796 | DOWN | 3.15E-06 |
| hsa-miR-501-3p | 3.85 | 1.35 | -1.956336781 | DOWN | 1.16E-35 |
| hsa-miR-3065-5p | 1.6 | 0.56 | -1.961836578 | DOWN | 1.18E-15 |
| hsa-miR-1908-5p | 0.66 | 0.22 | -1.986084124 | DOWN | 2.04E-07 |
| hsa-miR-2355-3p | 0.24 | 0.08 | -1.986084124 | DOWN | 0.000904061 |
| hsa-miR-107 | 15.04 | 4.77 | -2.069546847 | DOWN | 1.96E-141 |
| hsa-miR-193b-5p | 4.4 | 1.37 | -2.100462145 | DOWN | 4.23E-44 |
| hsa-miR-1294 | 0.5 | 0.15 | -2.111615006 | DOWN | 2.52E-06 |
| hsa-miR-1307-5p | 15.19 | 4.68 | -2.14184587 | DOWN | 2.96E-154 |
| hsa-miR-4701-5p | 0.32 | 0.1 | -2.178729202 | DOWN | 7.16E-05 |
| hsa-miR-891a-5p | 0.29 | 0.08 | -2.208476546 | DOWN | 0.000164777 |
| hsa-miR-500a-3p | 10.56 | 3.07 | -2.209400462 | DOWN | 3.75E-109 |
| hsa-miR-642a-5p | 0.81 | 0.23 | -2.220549378 | DOWN | 5.59E-10 |
| hsa-miR-1-3p | 23.87 | 6.87 | -2.231286429 | DOWN | 4.00E-250 |
| hsa-miR-22-5p | 18.24 | 5.1 | -2.264202858 | DOWN | 1.95E-192 |
| hsa-miR-548j-5p | 0.84 | 0.23 | -2.267855093 | DOWN | 1.75E-10 |
| hsa-miR-362-3p | 1.87 | 0.52 | -2.292745463 | DOWN | 5.04E-22 |
| hsa-miR-3659 | 0.7 | 0.21 | -2.29420642 | DOWN | 3.72E-09 |
| hsa-miR-4732-3p | 0.29 | 0.07 | -2.327121042 | DOWN | 0.000209981 |
| hsa-miR-513a-3p | 0.35 | 0.1 | -2.348654204 | DOWN | 1.22E-05 |
| hsa-miR-548av-3p | 1.16 | 0.28 | -2.348654204 | DOWN | 1.06E-13 |
| hsa-miR-18a-3p | 11.11 | 2.84 | -2.355540589 | DOWN | 4.21E-122 |
| hsa-miR-378e | 0.52 | 0.14 | -2.364595748 | DOWN | 1.79E-07 |
| hsa-miR-378g | 0.82 | 0.21 | -2.401121624 | DOWN | 8.40E-11 |
| hsa-miR-4284 | 0.21 | 0.06 | -2.401121624 | DOWN | 0.000474582 |
| hsa-miR-4664-5p | 1.38 | 0.35 | -2.401121624 | DOWN | 1.56E-17 |
| hsa-miR-627-5p | 0.33 | 0.08 | -2.460015313 | DOWN | 1.51E-05 |
| hsa-miR-378d | 4.69 | 1.08 | -2.536132728 | DOWN | 6.68E-58 |
| hsa-miR-193a-3p | 5.9 | 1.32 | -2.598870307 | DOWN | 9.63E-76 |
| hsa-miR-378c | 0.66 | 0.14 | -2.664156029 | DOWN | 7.11E-10 |
| hsa-miR-3617-5p | 0.21 | 0.04 | -2.723049719 | DOWN | 0.000304457 |
| hsa-miR-6499-5p | 3.21 | 0.6 | -2.818206952 | DOWN | 2.43E-44 |
| hsa-miR-885-5p | 5.98 | 0.97 | -2.992841491 | DOWN | 4.45E-84 |
| hsa-miR-5187-5p | 0.34 | 0.05 | -3.044977813 | DOWN | 1.55E-06 |
| hsa-miR-3944-5p | 0.86 | 0.14 | -3.056473452 | DOWN | 4.55E-14 |
| hsa-miR-526a-5p | 0.79 | 0.12 | -3.089177617 | DOWN | 3.35E-13 |
| hsa-miR-1299 | 1.97 | 0.3 | -3.091437124 | DOWN | 6.55E-30 |
| hsa-miR-10a-3p | 0.18 | 0.03 | -3.101561342 | DOWN | 0.000330877 |
| hsa-miR-629-5p | 1.31 | 0.2 | -3.108940872 | DOWN | 2.82E-21 |
| hsa-miR-6887-3p | 0.29 | 0.04 | -3.138087218 | DOWN | 1.15E-05 |
| hsa-miR-452-5p | 4.33 | 0.63 | -3.176767686 | DOWN | 8.47E-66 |
| hsa-miR-3150b-3p | 0.2 | 0.03 | -3.208476546 | DOWN | 0.000170472 |
| hsa-miR-582-3p | 0.27 | 0.04 | -3.208476546 | DOWN | 5.88E-06 |
| hsa-miR-548ar-3p | 0.31 | 0.04 | -3.275590742 | DOWN | 3.03E-06 |
| hsa-miR-143-5p | 1.64 | 0.22 | -3.319984861 | DOWN | 2.59E-27 |
| hsa-miR-512-5p | 0.63 | 0.08 | -3.339721079 | DOWN | 1.87E-11 |
| hsa-miR-520d-5p | 0.54 | 0.07 | -3.401121624 | DOWN | 2.66E-10 |
| hsa-miR-3150b-5p | 0.35 | 0.04 | -3.516598841 | DOWN | 2.06E-07 |
| hsa-miR-145-3p | 6.81 | 0.8 | -3.523280206 | DOWN | 9.41E-114 |
| hsa-miR-1973 | 0.23 | 0.04 | -3.571046625 | DOWN | 1.12E-05 |
| hsa-miR-520a-5p | 1.43 | 0.15 | -3.642129723 | DOWN | 2.14E-25 |
| hsa-miR-517-5p | 2.03 | 0.2 | -3.713399548 | DOWN | 3.94E-36 |
| hsa-miR-548d-3p | 0.15 | 0.01 | -3.860553242 | DOWN | 0.00031597 |
| hsa-miR-383-5p | 0.92 | 0.08 | -3.882248313 | DOWN | 1.16E-17 |
| hsa-miR-152-5p | 0.34 | 0.03 | -4.101561342 | DOWN | 4.72E-08 |
| hsa-miR-4517 | 0.17 | 0.01 | -4.101561342 | DOWN | 7.95E-05 |
| hsa-miR-520g-3p | 0.39 | 0.03 | -4.259102619 | DOWN | 5.95E-09 |
| hsa-miR-1972 | 0.2 | 0.01 | -4.308012219 | DOWN | 1.99E-05 |
| hsa-miR-525-5p | 1.14 | 0.07 | -4.436745533 | DOWN | 9.45E-23 |
| hsa-miR-3622a-5p | 0.12 | 0 | -4.571046625 | DOWN | 0.000594821 |
| hsa-miR-548e-3p | 0.28 | 0.01 | -4.723049719 | DOWN | 6.54E-07 |
| hsa-miR-526b-5p | 1.7 | 0.1 | -4.74689646 | DOWN | 6.72E-34 |
| hsa-miR-520h | 1.86 | 0.08 | -4.89297472 | DOWN | 1.10E-37 |
| hsa-miR-203a-5p | 0.75 | 0.04 | -5.156009126 | DOWN | 2.77E-16 |
| hsa-miR-517a-3p | 11.12 | 0.4 | -5.204918726 | DOWN | 1.43E-220 |
| hsa-miR-1323 | 1.21 | 0.04 | -5.308012219 | DOWN | 3.75E-26 |
| hsa-miR-3619-3p | 0.24 | 0 | -5.571046625 | DOWN | 1.48E-06 |
| hsa-miR-516a-5p | 4.13 | 0.1 | -5.832171441 | DOWN | 3.31E-84 |
| hsa-miR-550b-2-5p | 0.9 | 0.01 | -6.445515743 | DOWN | 1.58E-19 |
| hsa-miR-523-3p | 0.56 | 0 | -6.758673628 | DOWN | 1.33E-12 |
| hsa-miR-378i | 1.02 | 0 | -7.667908164 | DOWN | 5.64E-21 |
