# Supplementary material for: Genome-Wide Expression Difference of MicroRNAs in Basal Cell Carcinoma
Source: J Immunol Res. 2021 Aug 4;2021:7223500. doi: 10.1155/2021/7223500 (PMC8357504; doi:10.1155/2021/7223500)
Supplement: Supplementary Materials — Supplementary Figure 1: length distribution of sRNA tags in six sequencing libraries. Supplementary Figure 2: KEGG pathway of basal cell carcinoma and 24 miRNA-regulated gene members marked in a red box. Supplementary Table 1: information of TaqMan probes used in quantitative PCR. Supplementary Table 2: list of miRNAs identified in this study. Supplementary Table 3: differentially expressed miRNAs (DEMs) in the BCCs when compared with control. Supplementary Table 4: list of the enriched “biological process” GO terms of targeted genes of DEMs between the control and BCC groups. Supplementary Table 5: miRNA-targeted gene analysis in the basal cell carcinoma (ko05217) pathway. u: upregulation; d: downregulation. [file 7223500.f1.zip › 7223500.f6.docx]

Table S4. List of the enriched "Biological process" GO terms of targeted genes of DEMs between control and BCC groups.

| **Gene ontology** | **GO term id** | **GO term description** | **FDR** |
| --- | --- | --- | --- |
| Biological process | GO:0009812 | Flavonoid metabolic process | 2.13E-07 |
| Biological process | GO:0001504 | Neurotransmitter uptake | 4.27E-06 |
| Biological process | GO:0098657 | Import into cell | 9.78E-06 |
| Biological process | GO:0098609 | Cell-cell adhesion | 3.72E-05 |
| Biological process | GO:0001570 | Vasculogenesis | 5.39E-05 |
| Biological process | GO:0002011 | Morphogenesis of an epithelial sheet | 8.85E-05 |
| Biological process | GO:0007064 | Mitotic sister chromatid cohesion | 1.17E-04 |
| Biological process | GO:0006023 | Aminoglycan biosynthetic process | 1.29E-04 |
| Biological process | GO:0002252 | Immune effector process | 2.26E-04 |
| Biological process | GO:0032410 | Negative regulation of transporter activity | 2.91E-04 |
| Biological process | GO:0045741 | Positive regulation of epidermal growth factor-activated receptor activity | 3.52E-04 |
| Biological process | GO:0045742 | Positive regulation of epidermal growth factor receptor signaling pathway | 3.99E-04 |
| Biological process | GO:0050731 | Positive regulation of peptidyl-tyrosine phosphorylation | 4.28E-04 |
| Biological process | GO:0061098 | Positive regulation of protein tyrosine kinase activity | 4.47E-04 |
| Biological process | GO:1901186 | Positive regulation of erbb signaling pathway | 4.84E-04 |
| Biological process | GO:0002285 | Lymphocyte activation involved in immune response | 5.26E-04 |
| Biological process | GO:0010669 | Epithelial structure maintenance | 5.37E-04 |
| Biological process | GO:0006940 | Regulation of smooth muscle contraction | 5.63E-04 |
| Biological process | GO:0002263 | Cell activation involved in immune response | 5.71E-04 |
| Biological process | GO:1901137 | Carbohydrate derivative biosynthetic process | 5.79E-04 |
| Biological process | GO:0030334 | Regulation of cell migration | 5.88E-04 |
| Biological process | GO:0007159 | Leukocyte cell-cell adhesion | 5.92E-04 |
| Biological process | GO:0043624 | Cellular protein complex disassembly | 5.96E-04 |
| Biological process | GO:0016192 | Vesicle-mediated transport | 1.17E-03 |
| Biological process | GO:0140014 | Mitotic nuclear division | 1.93E-03 |
| Biological process | GO:0043551 | Regulation of phosphatidylinositol 3-kinase activity | 2.41E-03 |
| Biological process | GO:0002366 | Leukocyte activation involved in immune response | 2.79E-03 |
| Biological process | GO:0043588 | Skin development | 3.88E-03 |
| Biological process | GO:0010830 | Regulation of myotube differentiation | 4.27E-03 |
| Biological process | GO:0051270 | Regulation of cellular component movement | 4.69E-03 |
| Biological process | GO:2000145 | Regulation of cell motility | 5.24E-03 |
| Biological process | GO:0030216 | Keratinocyte differentiation | 5.71E-03 |
| Biological process | GO:0000070 | Mitotic sister chromatid segregation | 6.13E-03 |
| Biological process | GO:0030041 | Actin filament polymerization | 6.47E-03 |
| Biological process | GO:0002407 | Dendritic cell chemotaxis | 7.06E-03 |
| Biological process | GO:0036336 | Dendritic cell migration | 7.64E-03 |
| Biological process | GO:0045063 | T-helper 1 cell differentiation | 8.27E-03 |
| Biological process | GO:0010594 | Regulation of endothelial cell migration | 9.73E-03 |
| Biological process | GO:0010632 | Regulation of epithelial cell migration | 9.96E-03 |
