# Supplementary material for: Genome-Wide Expression Difference of MicroRNAs in Basal Cell Carcinoma
Source: J Immunol Res. 2021 Aug 4;2021:7223500. doi: 10.1155/2021/7223500 (PMC8357504; doi:10.1155/2021/7223500)
Supplement: Supplementary Materials — Supplementary Figure 1: length distribution of sRNA tags in six sequencing libraries. Supplementary Figure 2: KEGG pathway of basal cell carcinoma and 24 miRNA-regulated gene members marked in a red box. Supplementary Table 1: information of TaqMan probes used in quantitative PCR. Supplementary Table 2: list of miRNAs identified in this study. Supplementary Table 3: differentially expressed miRNAs (DEMs) in the BCCs when compared with control. Supplementary Table 4: list of the enriched “biological process” GO terms of targeted genes of DEMs between the control and BCC groups. Supplementary Table 5: miRNA-targeted gene analysis in the basal cell carcinoma (ko05217) pathway. u: upregulation; d: downregulation. [file 7223500.f1.zip › 7223500.f7.docx]

Table S5. MiRNA-targeted gene analysis in the "basal cell carcinoma" (ko05217) pathway. u: up-regulation; d: down-regulation.

| **Target gene description (gene symbles, gene ID)** | **miRNA** | **Fold changes** |
| --- | --- | --- |
| Patched 1 (*PTCH1*, NM_001083606) | hsa-miR-320b | 1.78, u |
|  | hsa-miR-30c-1-3p | 1.81, u |
| Smoothened homolog precursor (*SMO*, NM_005631) | hsa-miR-125a-3p | 1.72, u |
|  | hsa-miR-30c-1-3p | 1.81, u |
|  | hsa-miR-629-3p | -1.27, d |
|  | hsa-miR-3176 | -1.62, d |
|  | hsa-miR-4701-5p | -2.18, d |
|  | hsa-miR-3194-3p | -1.88, d |
| Kinesin family member 7 (*KIF7*, NM_198525) | hsa-miR-18a-3p | -2.36, d |
| Suppressor of fused homolog (*SUFU*, NM_016169) | hsa-miR-30b-3p | 1.29, u |
|  | hsa-miR-370-3p | -1.33, d |
|  | hsa-miR-3944-5p | -3.06, d |
|  | hsa-miR-1908-5p | -1.99, d |
| Zinc finger protein GLI3 (*GLI3*, NM_000168) | hsa-miR-1269b | 7.88, u |
|  | hsa-miR-509-3-5p | 1.11, u |
|  | hsa-miR-3180-3p | 3.85, u |
|  | hsa-miR-6815-5p | 2.68, u |
| Bone morphogenetic protein 2 (*BMP2*, NM_001200) | hsa-miR-18a-3p | -2.36, d |
|  | hsa-miR-370-3p | -1.33, d |
|  | hsa-miR-135b-3p | -1.44, d |
|  | hsa-miR-766-3p | -1.56, d |
| Hedgehog interacting protein 1 (*HIP1*, NM_032425) | hsa-miR-30c-1-3p | 1.81, u |
|  | hsa-miR-328-3p | 1.51, u |
|  | hsa-miR-3180-3p | 3.85, u |
|  | hsa-miR-766-3p | -1.56, d |
|  | hsa-miR-18a-3p | -2.36, d |
|  | hsa-miR-370-3p | -1.33, d |
| Zinc finger protein GLI1 (*GLI1*, NM_001167609) | hsa-miR-3622a-5p | -4.57, d |
|  | hsa-miR-18a-3p | -2.36, d |
|  | hsa-miR-134-5p | -1.50, d |
|  | hsa-miR-6815-5p | 2.68, u |
|  | hsa-miR-4664-5p | -2.40, d |
|  | hsa-miR-512-5p | -3.34, d |
|  | hsa-miR-6501-5p | -1.66, d |
| Wingless-type MMTV integration site family, member 9 (*WNT*, NM_001043318) | hsa-miR-3622a-5p | -4.57, d |
|  | hsa-miR-6734-5p | -1.78, d |
|  | hsa-miR-942-5p | -1.20, d |
|  | hsa-miR-1908-5p | -1.99, d |
| Tumor protein p53 (*TP53*, NM_001126113) | hsa-miR-30b-3p | 1.29, u |
|  | hsa-miR-6815-5p | 2.68, u |
|  | hsa-miR-1247-3p | 1.53, u |
| Cyclin-dependent kinase inhibitor 1A (*CDKN1A/P21*, NM_000389) | hsa-miR-6734-5p | -1.78, d |
|  | hsa-miR-3180-3p | 3.85, u |
|  | hsa-miR-3194-3p | -1.88, d |
|  | hsa-miR-520a-5p | -3.64, d |
|  | hsa-miR-525-5p | -4.44, d |
|  | hsa-miR-5187-5p | -3.04, d |
|  | hsa-miR-1972 | -4.31, d |
|  | hsa-miR-4488 | -1.76, d |
| Apoptosis regulator BAX (*BAX*, NM_138761) | hsa-miR-10b-3p | -1.86, d |
|  | hsa-miR-193b-5p | -2.10, d |
|  | hsa-miR-548j-5p | -2.27, d |
|  | hsa-miR-3622a-5p | -4.57, d |
| DNA damage-binding protein 2 (*DDB2/P48*, NM_000107) | hsa-miR-675-5p | 3.62, u |
|  | hsa-miR-1299 | -3.09, d |
|  | hsa-miR-766-3p | -1.56, d |
| Growth arrest and DNA-damage-inducible protein (*GADD45*, NM_006705) | hsa-miR-328-3p | 1.51, u |
|  | hsa-miR-6887-3p | -3.14, d |
|  | hsa-miR-4732-3p | -2.33, d |
| Bcl-2 homologous antagonist/killer 1 (*BAK1*, NM_001188) | hsa-miR-193a-3p | -2.60, d |
|  | hsa-miR-125a-3p | 1.72, d |
|  | hsa-miR-1973 | -3.57, d |
|  | hsa-miR-526b-5p | -4.75, d |
|  | hsa-miR-370-3p | -1.33, d |
|  | hsa-miR-3176 | -1.62, d |
|  | hsa-miR-629-3p | -1.27, d |
| DNA polymerase kappa (*POLK*, NM_032027) | hsa-miR-3150b-3p | -3.21, d |
| Frizzled 4 (*FZD4*, NM_012193) | hsa-miR-30c-1-3p | 1.81, u |
|  | hsa-miR-125a-3p | 1.72, u |
|  | hsa-miR-525-5p | -4.44, d |
|  | hsa-miR-942-5p | -1.20, d |
|  | hsa-miR-378e | -2.36, d |
|  | hsa-miR-1268b | -1.54, d |
|  | hsa-miR-1294 | -2.11, d |
|  | hsa-miR-1972 | -4.31, d |
|  | hsa-miR-6734-5p | -1.78, d |
| Segment polarity protein dishevelled 3 (*DVL3*, NM_004423) | hsa-miR-675-5p | 3.62, u |
|  | hsa-miR-3622a-5p | -4.57, d |
|  | hsa-miR-378g | -1.40, d |
|  | hsa-miR-3200-3p | -1.79, d |
|  | hsa-miR-370-3p | -1.33, d |
|  | hsa-miR-3176 | -1.62, d |
|  | hsa-miR-1294 | -2.11, d |
| Glycogen synthase kinase 3 beta (*GSK3B*, NM_001146156) | hsa-miR-508-5p | 1.32, u |
|  | hsa-miR-143-5p | -3.32, d |
|  | hsa-miR-520d-5p | -3.40, d |
|  | hsa-miR-561-5p | -1.01, d |
|  | hsa-miR-3200-3p | -1.79, d |
| Axin 1 (*AXIN1*, NM_003502) | hsa-miR-501-3p | -1.96, d |
|  | hsa-miR-143-5p | -3.32, d |
|  | hsa-miR-378i | -7.67, d |
|  | hsa-miR-4488 | -1.76, d |
|  | hsa-miR-3150b-3p | -3.21, d |
| Adenomatosis polyposis coli protein (*APC2*, NM_005883) | hsa-miR-642a-5p | -2.22, d |
|  | hsa-miR-18a-3p | -2.36,d |
|  | hsa-miR-618 | -1.92, d |
|  | hsa-miR-30b-3p | 1.29, u |
|  | hsa-miR-6499-5p | -2.82, d |
|  | hsa-miR-6501-5p | -1.66, d |
|  | hsa-miR-382-5p | -1.28, d |
|  | hsa-miR-6499-5p | -2.82, d |
|  | hsa-miR-370-3p | -1.33, d |
|  | hsa-miR-766-3p | -1.56, d |
|  | hsa-miR-4664-5p | -2.40, d |
| Catenin beta 1 (*CTNNB1/b-catenin*, NM_001098209) | hsa-miR-1972 | -4.31, d |
|  | hsa-miR-9983-3p | 4,85, u |
| Transcription factor 7 (*TCF7*, NM_001206844) | hsa-miR-130a-3p | 1.25, u |
|  | hsa-miR-320b | 1.78, u |
|  | hsa-miR-508-5p | 1.32, u |
|  | hsa-miR-18a-3p | -2.36, d |
|  | hsa-miR-320c | 1.44, u |
|  | hsa-miR-320d | 2.09, u |
|  | hsa-miR-1299 | -3.09, d |
|  | hsa-miR-370-3p | -1.33, d |
|  | hsa-miR-3176 | -1.62, d |
|  | hsa-miR-6887-3p | -3.13, d |
|  | hsa-miR-3150b-3p | -3.21, d |
